# Influence of Poly(vinylpyrrolidone) concentration on properties of silver nanoparticles manufactured by modified thermal treatment method

**DOI:** 10.1371/journal.pone.0186094

**Published:** 2017-10-18

**Authors:** Leila Gharibshahi, Elias Saion, Elham Gharibshahi, Abdul Halim Shaari, Khamirul Amin Matori

**Affiliations:** Department of Physics, Faculty of Science, University of Putra Malaysia (UPM), Serdang, Selangor, Malaysia; Institute of Materials Science, GERMANY

## Abstract

Very narrow and pure silver nanoparticles were synthesized by modified thermal treatment method via oxygen and nitrogen flow in succession. The structural and optical properties of the calcined silver nanoparticles at 600°C with diverse Poly(vinylpyrrolidone) concentrations varied from 2% to 4% were studied by means of different techniques. Fourier transform infrared spectroscopy was used to monitor the production of pure Ag nanoparticles at a given Poly(vinylpyrrolidone) concentration. The X-ray powder diffraction spectra are evidence for the transformation of the amorphous sample at 30°C to the cubic crystalline nanostructures at the calcination temperatures for all Poly(vinylpyrrolidone) concentrations. The transmission electron microscopy images showed the creation of spherical silver nanoparticles with the average particle size decreased by increasing Poly(vinylpyrrolidone) concentrations from 4.61 nm at 2% to 2.49 nm at 4% Poly(vinylpyrrolidone). The optical properties were investigated by means of UV–vis absorption spectrophotometer, which showed an increase in the conduction band of Ag nanoparticles with increasing Poly(vinylpyrrolidone) concentrations from 2.83 eV at 2% Poly(vinylpyrrolidone) to 2.94 eV at 4% Poly(vinylpyrrolidone) due to decreasing particle size. This was due to less attraction between conduction electrons and metal ions for smaller particle size corresponding to fewer atoms that made up the metal nanoparticles.

## Introduction

Noble metal nanoparticles, especially silver nanoparticles with narrow size distributions, have very wide applications in the various fields of nanotechnology including being used as catalysis and biocatalysis [1–6], in surface enhanced resonance Raman scattering [7, 8], in function improvement of the Remie Fibers [9] in environmental application [10], in therapeutics, diagnostic assays and medical diagnostics [11–21], in thermal ablation, and radiotherapy enhancement [22–24] in drug and gene delivery [25, 26], sensors and optical biosensors [27–31], oil refining processes, and fuel cell technology [32], in plastic industry [33] and optical applications [34]. Also noble metal nanoparticles especially silver nanoparticles have biomedical and antimicrobial applications in human health care such as coating contact lenses, cardiovascular implants, wound dressing, bone cement and other implants, medical catheters, bandages, endodontic filling materials, dental instruments [35]. These applications are related to the size-dependent properties of the metal nanoparticles including the particle size-dependence of absorption energy, a blue shift of absorption wavelength, and an improvement of photocatalytic acting with a particle size reduction. The metal nanoparticles show these interesting physical and chemical properties due to many surface atoms and the quantum confinement of electrons [36]. The optical properties of the metal nanoparticles were described classically as the resonance oscillation of the surface plasmon due to interaction with an electromagnetic field [37], Gustav Mie first acquired the absorption spectrum of metal nanoparticles in 1908, which is based on the classical electrodynamics method. This approach delineated the oscillation of conduction electrons originated from the incident electromagnetic waves, which polarized metal nanoparticles and known as the localized surface Plasmon resonance (LSPR) [38].

Mie proposed a solution to Maxwell’s equations [37] which describes the extinction spectra of spherical particles; however, the classical methods consider a continuous system and free electrons, hence ignoring the discrete nature of electronic structures of metal nanoparticles [39]. Therefore, it needs to apply quantum-mechanical method on optical properties of metal nanoparticles [36, 40, 41].

Recently the optical properties of the metal nanoparticles are ascribed to intra-band quantum excitations of the conduction electrons [36, 41] mimicking the interactions of light on the metal surface via the photoelectric absorption and Compton scattering. In quantum approach, the intra-band excitations of conduction electrons from the lowest energy state to higher energy states considered interpreting the absorption spectrum of metal nanoparticles [36, 42]. Researchers have recently expended much effort to develop approaches of producing monodispersed nanoparticles and controlling the particle size of metal nanoparticles.[43]. The main aim of controlling nanoparticle size and shape is to get the best dimension for their applications. Usually, the shape, size, and size distribution of nanoparticles can be controlled by changing the production approaches, reducing agents and stabilizers [44–50].

Different organic materials can be used as reducing agents and stabilizers in producing nanoparticles. The most popular and important material, which can be used in the synthesis of nanoparticles is poly(vinylpyrrolidone) (PVP). PVP can be used in various roles in the synthesis of nanoparticles, for example, to stabilize the surface of particles, to control the rate of nanoparticles growth and dispersion, and as a reducing agent. In producing metal nanoparticles via polyol synthesis, the PVP is broadly applied as a stabilizer and shape controlling agent in which the PVP and metal interact via the carbonyl group and nitrogen atom of the pyrrolidine ring [51].

Different methods for synthesizing Ag nanoparticles by using inorganic salts as metal precursors have been reported; such as by chemical reduction [52–56], photochemical method [57–61], electrochemical method [62–66], microwave processing [67–69], ultrasound processing [70–72], and gamma irradiation [73–75]. All of these methods were used to control the silver nanoparticles’ size by changing the different parameters of the synthesis procedures, such as using the surfactant, capping, stabilizer and reduction agents or by changing the dose of the radiation in the gamma radiation, microwave assistant and photochemical synthesis. These techniques have produced particles in the appropriate and necessary size and shape, but have some disadvantages such as involving complicated preparation procedures, difficulty in attaining pure particles and producing poisonous products that may damage the environment.

To overcome some of these shortcomings, the modified thermal treatment method is proposed here for the synthesis of metal nanoparticles for the first time. The motivation of this study is to find a simple method for synthesizing pure metal nanoparticles and controlling their size simultaneously. The oxide nanomaterials; including ZnFe_2_O_4_, MnFe_2_O_4_, and CoFe_2_O_4_ [76–78]; ZnCr_2_O_4_ [79]; ZnO [80], CdO [81], TiO_2_ and ZrO_2_ [82, 83] were already synthesized using the thermal treatment method. In the above-mentioned studies, oxide nano materials were synthesized from a water-based solution consisting of a metal precursor and poly(vinylpyrrolidone)vinyl pyrrolidone (PVP) which is calcined at the specific temperatures and the size of the nanoparticles is controlled by the calcination temperature. This study is the first attempt to synthesise pure Ag nanoparticle without any impurity and control its size by changing the PVP concentrations via a modified thermal treatment method using the oxygen and nitrogen gases’ flow. The differences between the modified thermal treatment method and thermal treatment method are in the synthesis process and the final product. In the thermal treatment method, the nanoparticles are calcined in the box furnace without using any gases to produce oxide nanoparticles such as ferrite nanoparticles and semiconductor nanoparticles, which they are not very pure and contain some impurities such as carbon. The modified thermal treatment method is used for the first time to produce very pure and narrow noble metal nanoparticles by using oxygen and nitrogen gases and PVP as a capping agent. In this method, the size of the particles can be controlled by changing the synthesis parameters such as PVP concentration and calcination temperature, which we published in our previous work [84].

This research investigates the influence of PVP concentration on the size and optical properties of silver nanoparticles produced via a modified thermal treatment method. PVP plays important roles as a capping agent and in controlling the nanoparticles’ size, decreases the speed of agglomeration and improves the crystallinity of nanoparticles[81, 85]. The metal nanoparticles can be manufactured using the modified thermal treatment method by removing oxygen via nitrogen flow during calcination at a suitable temperature in a water-based solution comprising metal precursor and PVP as a capping agent. Because no other chemicals were added into the reactant, the modified thermal treatment method has the benefits of easiness, low expense, and is not harmful to the environment as no toxic and unwanted products are discharged into the drainage system. Therefore, the novelty of this simple bottom–up method is the synthesis of pure silver nanoparticles with narrow size, which their size can be controlled by changing PVP concentration.

One of the advantages of modified thermal treatment method in comparison with other methods of synthesizing nanoparticles such as synthesizing ferrite nanoparticles [86] and synthesizing of silver and silver oxide nanoparticles by thermal decomposition [87] is the purity of the products. In modified thermal treatment method, the synthesized nanoparticles are very pure silver nanoparticles without any oxygen or carbon, but in the other methods, the product is not completely pure. Furthermore, in comparison with the thermal decomposition method [87], the modified thermal treatment method is simpler in preparing pure silver nanoparticles and the metal precursor in these methods is different. Moreover, in modified thermal treatment method, the PVP is used as a capping agent and therefore in this method by changing the PVP concentration the size of the nanoparticles are controllable, but in the thermal decomposition method in synthesizing the silver nanoparticles [87] the PVP was not used, therefore, it might be impossible to control the size of the nanoparticles. The size of nanoparticles in modified thermal treatment method in comparison with other works is smaller due to use the PVP as a capping agent to prevent the agglomeration of particles. Moreover, in synthesizing silver nanoparticles by thermal decomposition [87], the heat treatment, which was used, is annealing that is a process in metallurgy to remove defects on the crystalline planes but the heat treatment in modified thermal treatment method is calcination, which is used to do a thermal decomposition mostly of minerals and the functional group such as PVP and it is not used to process metallic alloys during its production chain.

## Materials and methods

The materials, which were used in this method, are silver nitrate, PVP and deionized water as the metal precursor, a capping agent, and a solvent, respectively. Silver nitrate, AgNO_3_ (M = 169.88 g/mol), and PVP (MW = 58,000) were both bought from Sigma-Aldrich (St. Louis, USA). All chemicals are analytical grade products and used without further purification. An aqueous solution of PVP was prepared by dissolving different concentration of PVP in 100 ml of deionized water at room temperature (30°C); then, 50 mg of silver nitrate was added to the polymer solution, which was stirred for 3 hours using a magnetic stirrer. At the end of the 3 hour period, an achromatic solution with no sediment of materials was achieved. To observe the PVP concentration influence, the rate of PVP to water volume was changed from 2% to 4% during synthesis.

**Fig 1:**
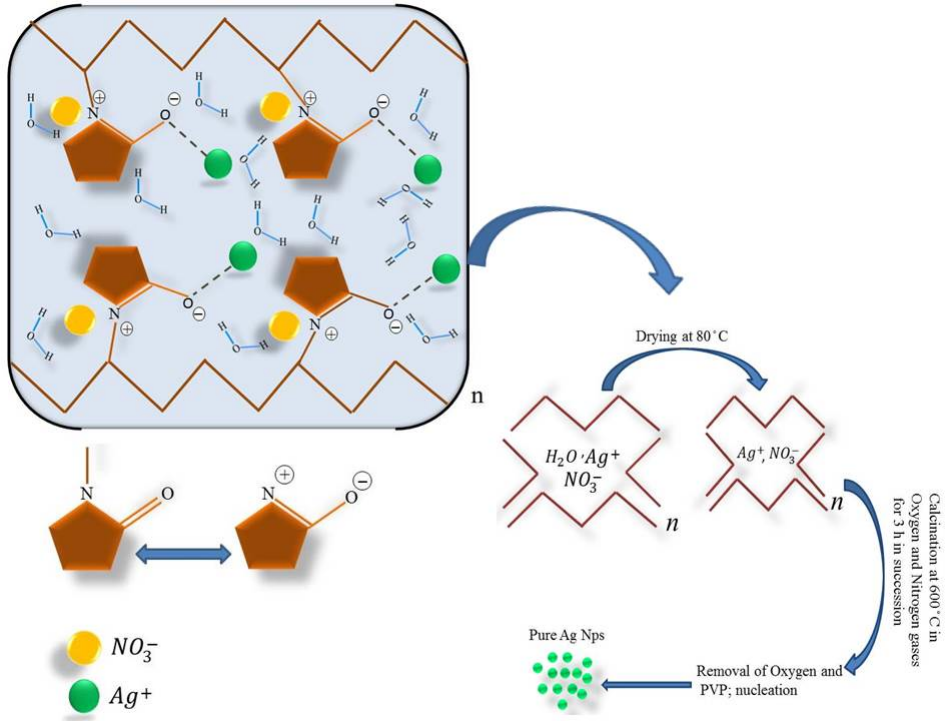
A schematic diagram for the synthesis of the nanoparticles by modified thermal treatment method.

The solution was poured into a glass Petri dish and heated in an oven at 80°C for 24 hours to evaporate the water. After drying, the solid that remained was crushed and was ground in a mortar to form a uniform powder. The calcination of the powder was conducted at 600°C for 3 hours in oxygen and nitrogen flow in succession to break down the organic combinations and oxygen respectively and to produce the silver nanocrystals. Both gases were flowed to the furnace chamber at the same flow rate of 50 mL/minute. Fig 1 shows a schematic diagram for the synthesis of the silver nanoparticles by modified thermal treatment method.

Several characterization techniques were used to study the influence of PVP concentration on the properties of synthesized silver nanoparticles. The crystal structure of Ag nanoparticles was examined by X-ray diffractometry (XRD Shimadzu model 6000, Shimadzu, Tokyo, Japan) using Cu Kα (0.154 nm) as an X-ray source to generate diffraction patterns from the crystalline samples at ambient temperature in the 2θ range of 10°–80°. The infrared spectra (280–4000 cm^-1^) were recorded using a Fourier transform infrared spectrometer (FTIR Perkin Elmer model 1650, Perkin Elmer, Waltham, USA) to monitor the removal of the capping agent for the samples calcined at 600°C according to the PVP concentrations. The XRD and FTIR results were applied to prove the creation of pure crystalline Ag nanoparticles with various concentration take us of PVP, which calcined at 600°C. The transmission electron microscopy (TEM model Hitachi H-7100 TEM, Hitachi, Tokyo, Japan) with an accelerating voltage of 500 kV was applied to investigate the morphology and particle size distribution of samples in which the images were achieved with the unified scale of 50 nm. The sample was prepared on the copper grids by drying a drop of Ag nanoparticles powder dispersion in deionized water. The sizes of the nanoparticles were measured by using the Image Tool software and the graph of the size distribution were drawn using the origin 9 software and fitted by a Gaussian function. Furthermore, the optical properties of the synthesized silver nanoparticles were investigated by means of UV–vis spectrophotometer (Shimadzu model UV-3600, Shimadzu, Tokyo, Japan) at room temperature between the wavelengths of 200–800 nm.

## Results and discussion

### Phase composition analysis (FTIR spectroscopy)

Fourier transform infrared spectroscopy (FTIR) is a technique used to identify the component of a sample and offers essential information relating the phase structure of the sample and type of the chemical bonds present between diverse compounds and polymers. In this work, FTIR spectroscopy was applied to monitor the removal of PVP and to investigate the ideal PVP concentration in which the purity of Ag nanoparticles formed. The FTIR spectra were applied between the wave numbers of 280–4000 cm^-1^, to determine the specimen compound consisting the organic and inorganic combinations of samples before calcination at 30°C and after calcination at 600°C with different PVP concentrations.

**Fig 2:**
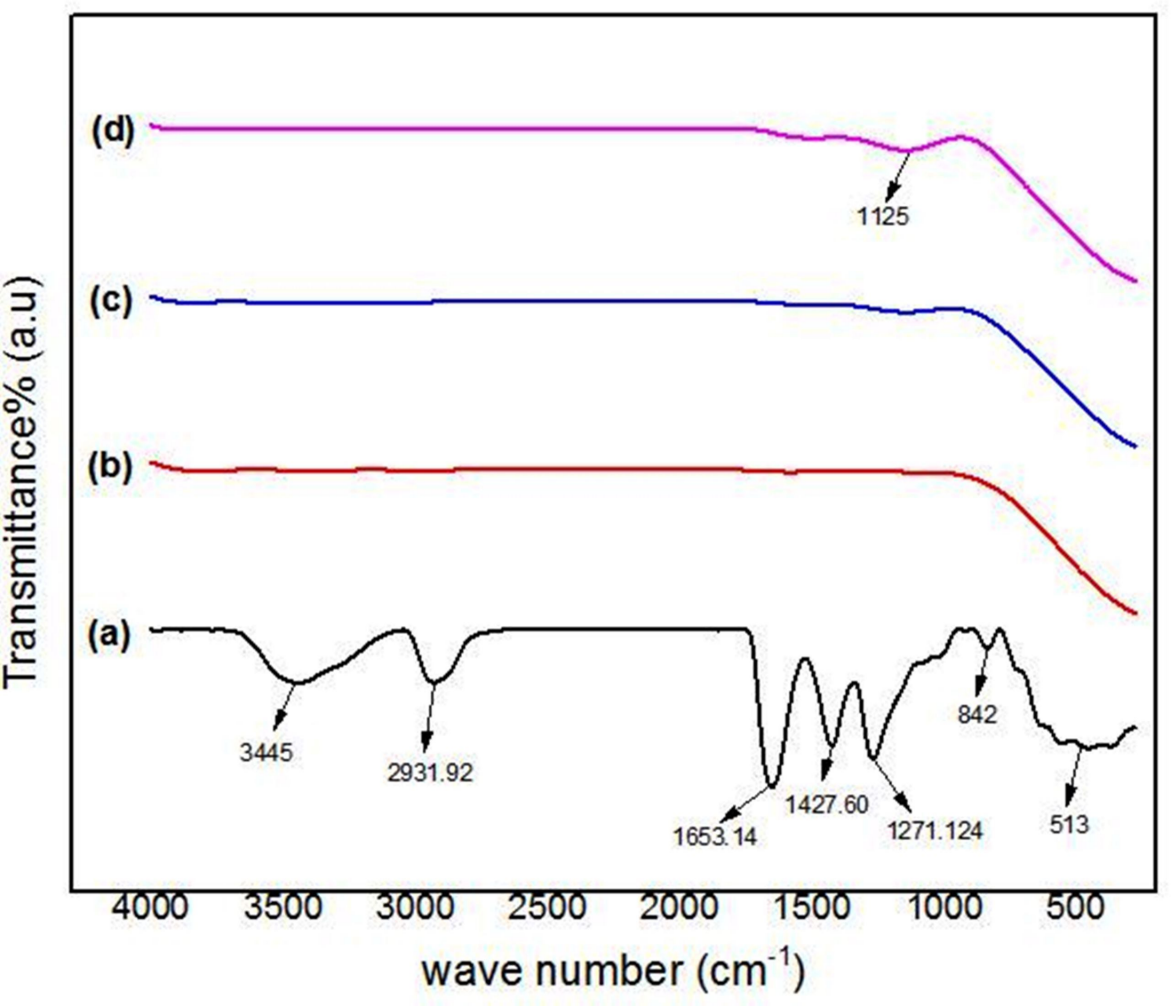
The FTIR spectra of samples in the range of 280–4500 cm^-1^. (a) before calcination (T = 30°C) and synthesized with (b) 2% PVP, (c) 3% PVP, (d) 4% PVP calcined at 600°C.

Fig 2 shows the FTIR spectra of the samples before calcination at room temperature (30°C) and after calcination at 600°C. For the sample before calcination at the room temperature, which contained PVP and silver precursor, as illustrated in Fig 2(A), the peaks with the absorption wave number of 3445, 2931, 1653, 1427, 1271, and 842 cm^-1^ is the representative of the covalent bonds vibration of the N-H, C-H, — C = C — stretching, C — C in the ring, C — N stretching, and C — C in ring, respectively. The peak at the 513 cm^-1^ corresponds to the ionic bond groups vibration of Ag — O [88, 89]. Fig 2(B)–2(D) present the FTIR spectra of the samples after calcination at 600°C synthesized with different PVP concentrations, 2, 3, and 4%, respectively. The Ag-Ag bonds could already form below 400 cm^-1^ but since the FTIR instrument uses the mid-inferred ray with the wave number of 4000–400 cm^-1^, it cannot produce enough energy to vibrate the metallic bonds. Therefore the spectra cannot display any absorption peaks of Ag-Ag bonds. Fig 2(B) refers to the sample, which was synthesized with 2% PVP and calcined at 600°C. As can be seen in the figure, there are no peaks in this spectrum, which shows the complete removal of PVP from the sample and pure silver nanoparticles were produced. Fig 2(C) is the spectrum for the sample with 3% PVP concentrations. It is clear that this spectrum in comparison with the Fig 2(B) is not so smooth after the wave number of 1000 cm^-1^, which can be the reason of the PVP trace in the sample. Fig 2(D) is the FTIR spectrum for the sample with 4% PVP concentration. The spectrum has a wide peak at the wave number of 1125 cm^-1^, which belongs to the C-N stretching bonds. The peak in this spectrum confirms the incomplete removal of the organic contents from the sample. The FTIR analysis determined that the best PVP concentration in the selected range of quantities for synthesizing pure Ag nanoparticles is the 2% PVP at this calcination temperature.

### Structural analysis

The effect of PVP concentrations on the structural properties of the Ag nanoparticles that were produced with different PVP concentrations via thermal treatment method was studied by means of X-ray Diffraction patterns.

**Fig 3:**
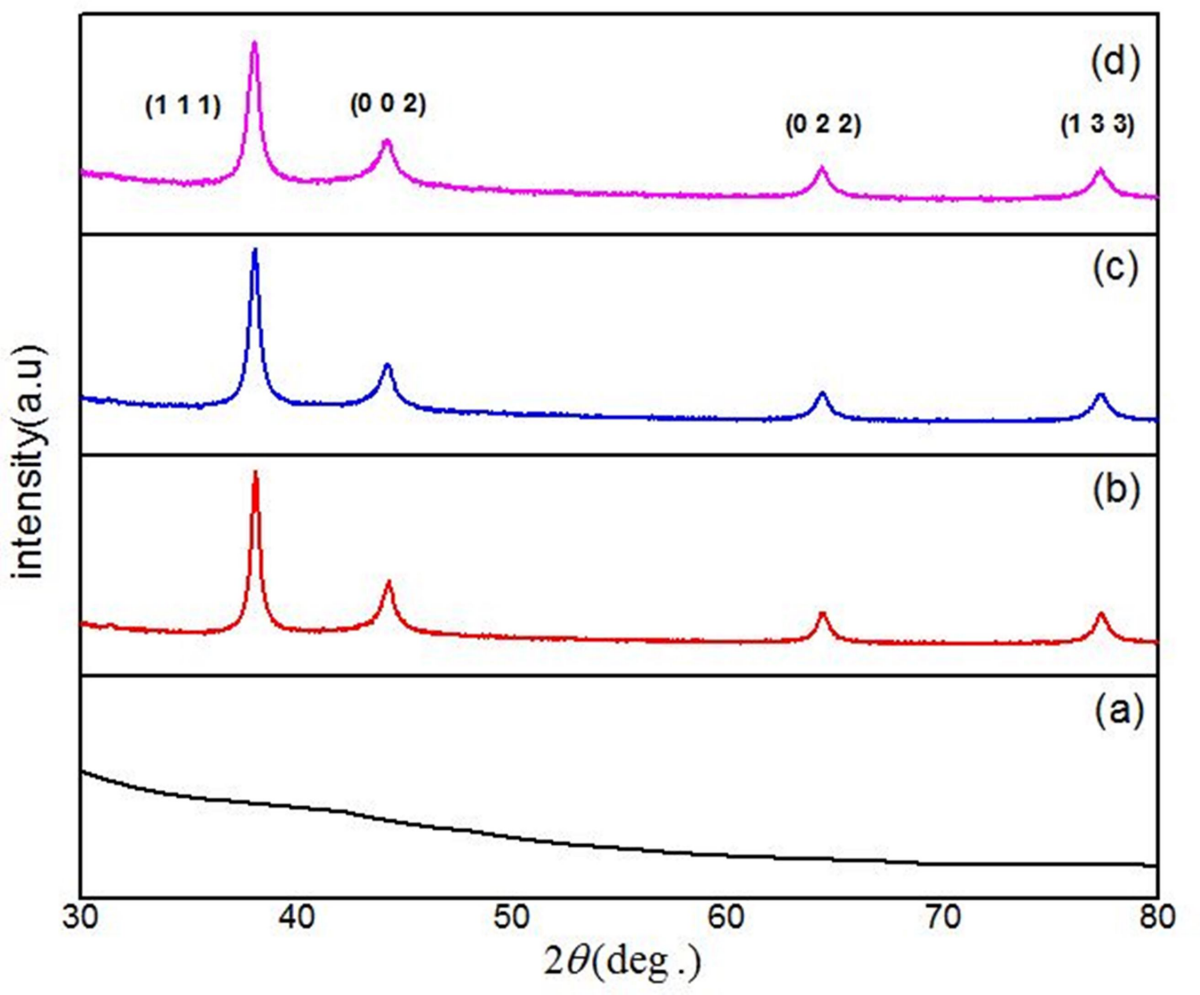
XRD patterns for Ag nanoparticles powder (a) before calcination (T = 30°C), (b) 2% PVP, (c) 3% PVP, and (d) 4% PVP after calcined at 600°C.

Fig 3 displays the typical XRD analysis of samples with different PVP concentrations (2%, 3%, and 4%) before and after calcination at 600°C. The XRD analysis for the sample before calcination displays no diffraction peaks, which is evidence that the sample was amorphous, including PVP, Ag^0^ and Ag^+^ ions at the room temperature. For the calcined samples at 600°C, the diffraction peaks imply the creation of crystalline Ag nanoparticles with the reflection planes of (1 1 1), (0 0 2), (0 2 2), and (1 3 3). The most prominent peak in all samples was found at 2θ = 38.0°, which relates to the reflection plane of (1 1 1). These reflection planes in the diffraction patterns demonstrate the existence of a cubic structure of Ag nanoparticles in the samples, which were produced with different PVP concentration and calcined at 600°C, referring to the reference code 98-006-2693 of cubical silver crystals in the standard phase reported of the XRD database with a = 4.0880 A°and volume = 68.32 A°.

Fig 3(B)–3(D) displays the XRD patterns of the Ag nanoparticles produced with different PVP concentrations, 2%, 3%, and 4% which calcined at 600°C. From XRD spectra, it is apparent that the diffraction peaks become broad by growing the PVP concentrations because the size of particles decreased by increasing the PVP concentrations (Table 1). The crystalline size of the synthesized Ag nanoparticles, which ranged from 4.30 to 3.37 nm, were calculated by using the Scherer equation, which is offered below and considers the broadening of the prominent peak, (1 1 1).

$$D(nm)=\frac{0.94\lambda }{\beta \cos\theta }$$ (1)

In which, *β* is the full width of the diffraction line at half of the maximum intensity (FWHM) measured in radians, *λ* is the X-ray wavelength of Cu Kα = 0.154 nm and *θ* is the Bragg angle.

**Table 1:** Structural properties of synthesized Ag nanoparticles with different PVP concentrations calcined at 600°C.

PVP (%)	2θ (deg.)±0.01	FWHM (deg.)±0.01	D_XRD_ (nm)	D_TEM_ (nm)
2	38.08	2.04	4.30	4.61±1.96
3	38.08	2.42	3.60	2.92±1.08
4	38.05	2.60	3.37	2.49±1.61

### Morphology and size distribution

**Fig 4:**
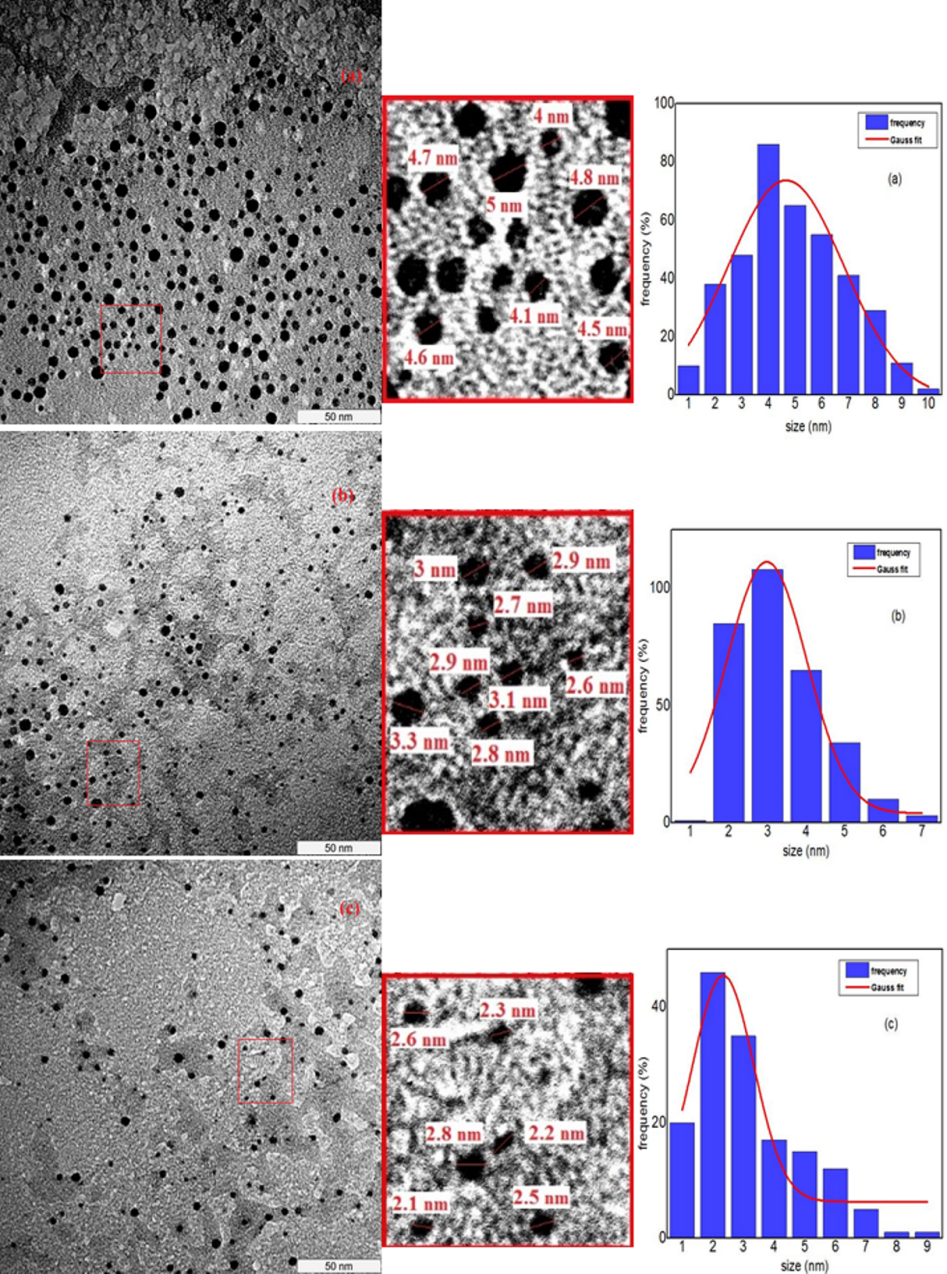
TEM images and particle size distributions of samples calcined at 600°C for different PVP concentrations (a) 2%, (b) 3%, and (c) 4%.

The morphological structure and average size distribution of the silver nanoparticles, which were prepared with different PVP concentration (2%, 3%, and 4%) are studied by using Transmission Electron Microscopy (TEM) images. TEM images are shown in Fig 4(A), 4(B) and 4(C) demonstrate that Ag nanoparticles have a spherical shape and are uniform in the morphology and size distribution for all samples with different PVP concentrations that calcined at 600°C. The average size and the standard division of the nanoparticles were obtained by means of the Image Tool software, which gives the diameters of the particles in nanometres according to the scale bar indicated in the enlarged images. Furthermore, the graph of the size distribution of the nanoparticles in each image was drawn using origin 9 software and fitted with the Gaussian function, which clearly indicated the size was decreasing due to the increase in PVP concentration. The unified scale of all images is 50 nm, which is shown in the bottom right of the TEM images. The average size of the Ag nanoparticles that calcined at 600°C and synthesized with different PVP concentrations, 2%, 3%, and 4% are about 4.61, 2.92, and 2.49 nm, respectively, which is well-matched with the XRD results (Table 1). These results express the reduction in particle size due to the increase in the PVP concentration used in the synthesis process. The researcher denominate the metal nanoparticles with this range of sizes as metal nanoclusters, which contain several to a hundred of atoms and can bridge between a single atom of metal and large metal nanoparticles [10, 90]. These silver nanoclusters have different applications in varied fields such as determination of nitrate ion in industrial applications, environmental application and health monitoring [91, 92], biosensor, bio imaging analysis, and biomedical studies [93].

In the synthesis of the samples via modified thermal treatment method, the silver ions (Ag^+^) interact with PVP in the form of the Ag (PVP)^+^. Due to these interactions, the metal particles will cap upon nucleation and Ag^+^ ions will stabilize with the complex compound. This stabilization of silver ions reduces the nucleation process and larger particles production. Furthermore, by increasing the concentrations of PVP from 2% to 4%, in the same concentration of metal nitrate and calcination temperature, more Ag^+^ ions interact with PVP and the growth of the particles is considerably controlled [85]. Therefore, it is reasonable to conclude that when the interaction between the PVP and metal particles increases, the ability of PVP for stabilizing metal particles increases too, which results in the smaller size of the metal nanoparticles.

### Optical properties

The localized plasmon resonance of a single metallic nanoparticle can be shifted in frequency via alterations in particle shape and size. In closely packed colloidal noble metallic nanoparticles, additional shifts are expected to occur due to electromagnetic interactions between the localized modes. For small particles, these interactions are essential to a dipolar nature, and the particle ensemble can in a first approximation be treated as an ensemble of interacting dipoles [38, 39, 94].

**Fig 5:**
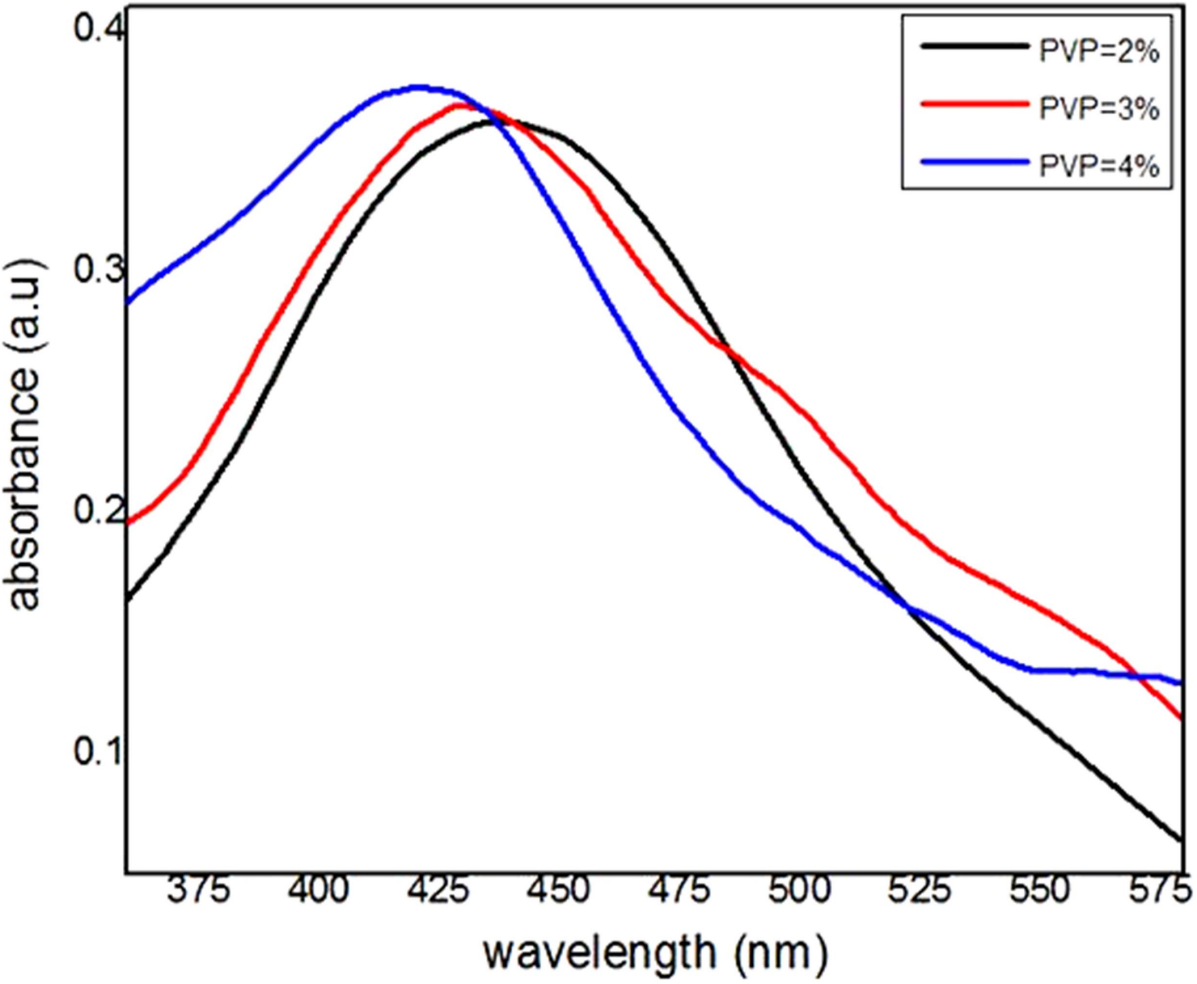
The UV-visible absorption spectrum of Ag nanoparticles synthesized with the same concentration of AgNO_3_ (50 mg) but the different concentration of PVP (2%, 3%, 4%) calcined at 600°C.

The effect of different PVP concentrations on the optical properties of silver nanoparticles synthesized by the thermal treatment method is determined by measuring the optical absorption via UV-vis spectrometer and calculating the conduction band energy of the samples. This spectrometer works based on comparing the intensities of two beams, which are transmitted from the sample and reference cavity. Therefore, the samples were prepared from the dispersion of the synthesized Ag nanoparticles powder, which calcined at 600°C with different PVP concentrations in a 2% PVP solution and the absorption spectra was measured between the wavelengths of 200–800 nm at ambient temperature, which are shown in Fig 5. It is clear from the absorption spectra that the maximum absorbance wavelengths (λ_max_) blue shifted from 438 to 421 nm by increasing the PVP concentrations from 2% to 4%. This blue shifting is due to the decreasing in size of the nanoparticles by increasing the PVP concentrations. The maximum absorbance wavelength is associated with the conduction band energy according to quantum theory of metal nanoparticles [40, 41, 85].

The conduction band energy, E_*cb*_ (eV) can be calculated directly from the UV-visible absorption spectra by using Einstein’s photon energy equation:

$$E_{cb}=\frac{hc}{\lambda_{max}}$$ (2)

Where *λ*_*max*_ is the maximum absorbance wavelength, *h* is the Planck constant and *c* is the speed of light. The conduction band energy illustrates the amount of energy needed to excite the conduction electrons from the lowest energy state to higher energy states affected by the UV-visible electromagnetic radiation [36, 41, 42]. The results are listed in Table 2.

**Table 2:** Optical properties of synthesized Ag nanoparticles with different PVP concentrations.

PVP concentration (%)	D_TEM (nm)_	Absorbance Wavelength (nm)	Conduction band (eV) by Eq (2)	Conduction band (eV) by Eq (3)
2	4.61±1.96	438	2.83	2.83
3	2.92±1.08	430	2.88	2.88
4	2.49±1.61	421	2.94	2.94

Because some of the absorption spectra have a wide peak, calculating the conduction band energy of produced silver nanoparticles indirectly from the absorption spectra by the following Tauc’s equation was also tried:

$$\left({\alpha h\nu )}^{2}=B(h\nu -E_{cb}\right)$$ (3)

**Fig 6:**
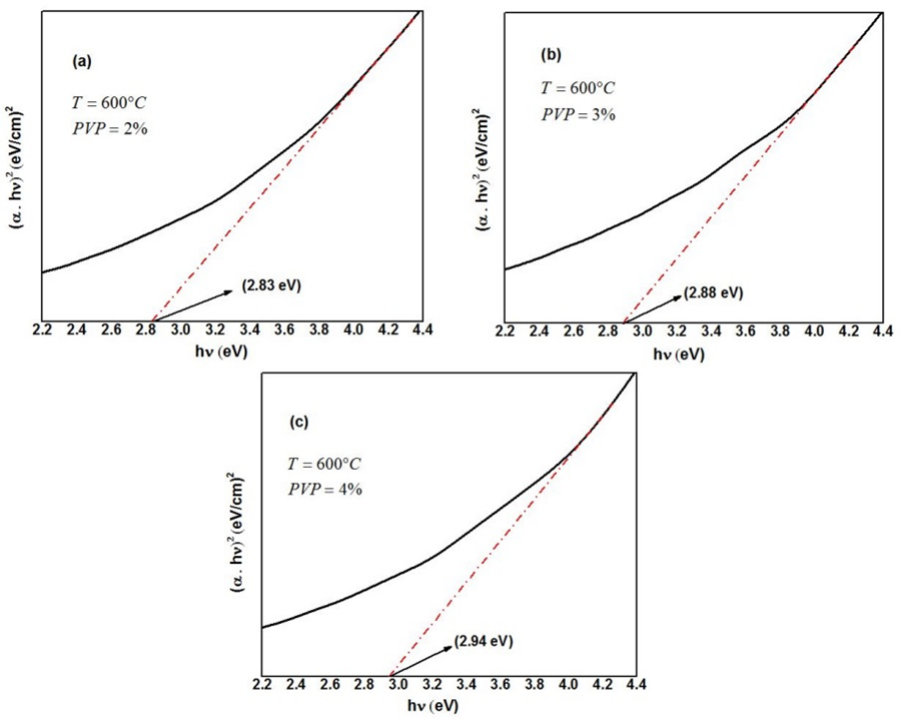
Tauc plots of Ag nanoparticles synthesized with 50 mg AgNO_3_ and different PVP concentrations (a) 2%, (b) 3%, and (c) 4%, calcined at 600°C.

Where *α* is the absorption coefficient, *hv* is the photon energy, *E*_cb_ is the conduction band energy, and *B* is a constant. According to this equation, by plotting the (*αhν*)^2^ versus (*hv*) and extrapolation of the linear part of the curve to the energy axis, the conduction band energy of Ag nanoparticles can be achieved, as shown in Fig 6 [85, 95]. It is obvious from Fig 6 that by increasing the PVP concentration from 2% to 4%, the conduction band energy increased from 2.83 to 2.94 eV. These results as listed in Table 2 are in agreement with the direct calculations from Einstein’s photon energy Eq (2).

Mie theory classically can describe the optical absorption of the metal nanoparticles system according to the localized surface plasmon resonance that relates to the interaction of the electromagnetic waves[37]. Recently the optical absorption of metal nanoparticles was easily described by the quantum mechanical principle due to intra-band excitations of conduction electrons by photon [40, 41, 96, 97], mimicking the interactions of light on the metal surface via the photoelectric absorption and Compton scattering [42]. In metal nanoparticles, the conduction electrons are not entirely free like in the bulk structure, but instead, some are held by the individual atoms and some are free and moved between atoms to form a metallic bond that cement the metal nanoparticles. Upon receiving photon energy of UV light having the maximum absorbance wavelengths (*λ*_*max*_), the conduction electrons experience intra-band quantum excitation beyond the Fermi energy, of which the conduction band of metal nanoparticle is defined. For smaller particle size, fewer numbers of atoms from the particle and thereby reduce the potential attraction between the conduction electrons and metal ions of the particle. In this way, the conduction band energy increases for the smaller particle. On the other hand, for larger particle sizes, large numbers of atoms forming the particle and increase the potential attraction between conduction electrons and metal ions and therefore reduce the conduction band energy of the metal nanoparticles [41, 96–98].

## Conclusions

In this study, the effect of PVP concentration on the size and optical properties of Ag nanoparticles produced by modified thermal treatment method was investigated. The presence of PVP in producing Ag nanoparticles was essential. The PVP behaves as a capping agent in the synthesis of Ag nanoparticles, which capped the Ag atoms and prevented them from agglomeration. Therefore, by increasing the PVP concentration, more Ag atoms will cap and at the end, the size of the silver nanoparticles will be decreased.

The optimum PVP concentration that can be used to produce pure Ag nanoparticles by modified thermal treatment method was 2%. The absorbance wavelength of Ag nanoparticles has blue-shifted from 438 to 421 nm corresponding to the average particle size decrease of 4.61 to 2.49 nm by increasing the PVP concentration from 2% to 4%. The conduction band energy increased from 2.83 eV at 2% PVP to 2.94 eV at 4% PVP due to less attraction between conduction electrons and metal ions for the smaller particle size.

The minimal data set is available in the paper and S1 File.

## Supporting information

S1 File Minimal dataset. (PDF)
