# Supplementary material for: Influence of Poly(vinylpyrrolidone) concentration on properties of silver nanoparticles manufactured by modified thermal treatment method
Source: PLoS One. 2017 Oct 18;12(10):e0186094. doi: 10.1371/journal.pone.0186094 (PMC5646761; doi:10.1371/journal.pone.0186094)
Supplement: S1 File — (PDF) [file pone.0186094.s001.pdf]

Fig 2 data:

wave number	Transmittance	Transmittance	Transmittance	Transmittance
(cm^{-1})				
		2%	3%	4%
4000	99.61892	104.21043	102.38465	101.20301
3999	99.59793	104.14657	102.32413	101.15716
3998	99.57733	104.08317	102.26424	101.11191
3997	99.55735	104.02024	102.20499	101.06727
3996	99.53823	103.95777	102.14637	101.02323
3995	99.52021	103.89577	102.08839	100.9798
3994	99.50351	103.83422	102.03104	100.93695
3993	99.48836	103.77313	101.97432	100.89471
3992	99.47494	103.71251	101.91823	100.85305
3991	99.4634	103.65234	101.86277	100.81198
3990	99.45387	103.59263	101.80793	100.7715
3989	99.4464	103.53338	101.75372	100.7316
3988	99.44095	103.47459	101.70013	100.69229
3987	99.43742	103.41626	101.64716	100.65355
3986	99.43562	103.35838	101.59481	100.61539
3985	99.43531	103.30095	101.54307	100.5778
3984	99.43619	103.24398	101.49195	100.54078
3983	99.43798	103.18747	101.44145	100.50432
3982	99.44038	103.1314	101.39155	100.46843
3981	99.44316	103.07579	101.34226	100.4331
3980	99.44612	103.02063	101.29358	100.39833
3979	99.44911	102.96592	101.2455	100.36411
3978	99.45204	102.91166	101.19803	100.33045
3977	99.45483	102.85785	101.15115	100.29733
3976	99.45745	102.80449	101.10487	100.26475
3975	99.45993	102.75157	101.05919	100.23272
3974	99.46232	102.6991	101.01409	100.20123
3973	99.46473	102.64707	100.96959	100.17027
3972	99.46729	102.59549	100.92568	100.13984
3971	99.47014	102.54435	100.88235	100.10994
3970	99.47341	102.49366	100.8396	100.08057
3969	99.4772	102.4434	100.79743	100.05172
3968	99.48156	102.39359	100.75584	100.02338
3967	99.48655	102.34421	100.71482	99.99556
3966	99.49222	102.29528	100.67437	99.96825
3965	99.49862	102.24678	100.63449	99.94145
3964	99.50583	102.19871	100.59518	99.91516

3963	99.51394	102.15108	100.55643	99.88936
3962	99.523	102.10389	100.51824	99.86406
3961	99.53303	102.05713	100.48061	99.83925
3960	99.544	102.0108	100.44353	99.81493
3959	99.55579	101.9649	100.407	99.79109
3958	99.56824	101.91943	100.37101	99.76774
3957	99.58119	101.87439	100.33557	99.74486
3956	99.59447	101.82978	100.30068	99.72246
3955	99.60795	101.78559	100.26632	99.70053
3954	99.62122	101.74182	100.23249	99.67906
3953	99.63477	101.69849	100.1992	99.65806
3952	99.64829	101.65557	100.16644	99.63751
3951	99.6616	101.61307	100.1342	99.61742
3950	99.67448	101.571	100.10248	99.59778
3949	99.68672	101.52934	100.07128	99.57859
3948	99.69816	101.4881	100.04059	99.55983
3947	99.70863	101.44728	100.01042	99.54152
3946	99.71805	101.40687	99.98075	99.52364
3945	99.72638	101.36688	99.95159	99.50619
3944	99.7336	101.32729	99.92292	99.48916
3943	99.73977	101.28812	99.89476	99.47256
3942	99.74492	101.24936	99.86709	99.45637
3941	99.74913	101.21101	99.8399	99.4406
3940	99.7525	101.17306	99.81321	99.42524
3939	99.75514	101.13552	99.78699	99.41028
3938	99.75716	101.09838	99.76126	99.39572
3937	99.75871	101.06165	99.736	99.38156
3936	99.75993	101.02531	99.71121	99.36779
3935	99.76095	100.98938	99.68689	99.35441
3934	99.76189	100.95384	99.66303	99.34142
3933	99.7628	100.9187	99.63964	99.3288
3932	99.76369	100.88396	99.61669	99.31656
3931	99.76448	100.84961	99.59421	99.30469
3930	99.76503	100.81565	99.57216	99.29318
3929	99.76516	100.78208	99.55057	99.28204
3928	99.76474	100.74891	99.52941	99.27126
3927	99.76363	100.71612	99.50869	99.26083
3926	99.76182	100.68371	99.48841	99.25075
3925	99.75932	100.65169	99.46855	99.24101
3924	99.75618	100.62006	99.44912	99.23162
3923	99.75247	100.5888	99.4301	99.22256

3922	99.74821	100.55793	99.41151	99.21383
3921	99.7434	100.52743	99.39333	99.20543
3920	99.73798	100.49731	99.37555	99.19736
3919	99.73195	100.46757	99.35818	99.1896
3918	99.7253	100.4382	99.34122	99.18215
3917	99.71815	100.4092	99.32464	99.17502
3916	99.71063	100.38057	99.30846	99.16819
3915	99.70294	100.3523	99.29267	99.16167
3914	99.69522	100.32441	99.27727	99.15544
3913	99.68755	100.29688	99.26224	99.1495
3912	99.67991	100.26971	99.24759	99.14385
3911	99.67222	100.24291	99.23331	99.13848
3910	99.66444	100.21646	99.2194	99.1334
3909	99.65662	100.19037	99.20585	99.12859
3908	99.64888	100.16464	99.19267	99.12404
3907	99.64144	100.13927	99.17983	99.11977
3906	99.63449	100.11424	99.16735	99.11576
3905	99.62814	100.08957	99.15522	99.112
3904	99.62231	100.06524	99.14343	99.1085
3903	99.61652	100.04127	99.13198	99.10524
3902	99.61105	100.01764	99.12086	99.10223
3901	99.60532	99.99435	99.11008	99.09946
3900	99.59915	99.9714	99.09962	99.09693
3899	99.59251	99.9488	99.08948	99.09462
3898	99.58549	99.92653	99.07966	99.09255
3897	99.57825	99.9046	99.07016	99.09069
3896	99.57101	99.883	99.06097	99.08906
3895	99.56393	99.86174	99.05208	99.08764
3894	99.55717	99.8408	99.04349	99.08643
3893	99.55087	99.8202	99.0352	99.08542
3892	99.54509	99.79992	99.0272	99.08462
3891	99.53989	99.77997	99.0195	99.08401
3890	99.53527	99.76034	99.01208	99.0836
3889	99.53124	99.74103	99.00494	99.08337
3888	99.52779	99.72204	98.99807	99.08334
3887	99.52491	99.70336	98.99148	99.08348
3886	99.52266	99.68501	98.98516	99.08379
3885	99.52107	99.66696	98.97911	99.08428
3884	99.52024	99.64923	98.97331	99.08494
3883	99.52025	99.63181	98.96777	99.08576
3882	99.52117	99.61469	98.96248	99.08675

3881	99.52303	99.59788	98.95745	99.08789
3880	99.52586	99.58137	98.95265	99.08918
3879	99.52963	99.56517	98.9481	99.09061
3878	99.53433	99.54926	98.94379	99.0922
3877	99.53997	99.53365	98.9397	99.09392
3876	99.54656	99.51833	98.93585	99.09578
3875	99.55407	99.50331	98.93222	99.09777
3874	99.56238	99.48858	98.92881	99.09989
3873	99.5713	99.47414	98.92562	99.10214
3872	99.58055	99.45999	98.92264	99.1045
3871	99.58982	99.44612	98.91987	99.10699
3870	99.59887	99.43254	98.91731	99.10958
3869	99.60759	99.41923	98.91494	99.11229
3868	99.61594	99.40621	98.91278	99.1151
3867	99.62352	99.39346	98.91081	99.11802
3866	99.63113	99.38098	98.90903	99.12103
3865	99.63821	99.36878	98.90743	99.12414
3864	99.64459	99.35685	98.90602	99.12733
3863	99.64999	99.34519	98.90479	99.13062
3862	99.65419	99.33379	98.90373	99.13399
3861	99.65708	99.32266	98.90285	99.13744
3860	99.65876	99.31179	98.90213	99.14097
3859	99.65954	99.30118	98.90157	99.14458
3858	99.65985	99.29083	98.90118	99.14825
3857	99.66009	99.28073	98.90094	99.15199
3856	99.66055	99.27089	98.90086	99.15579
3855	99.66133	99.2613	98.90093	99.15966
3854	99.66235	99.25195	98.90114	99.16358
3853	99.66347	99.24286	98.9015	99.16756
3852	99.66454	99.23401	98.90199	99.17158
3851	99.66542	99.2254	98.90262	99.17566
3850	99.66602	99.21703	98.90339	99.17977
3849	99.66624	99.20891	98.90428	99.18393
3848	99.66599	99.20102	98.9053	99.18813
3847	99.66517	99.19336	98.90644	99.19237
3846	99.66374	99.18593	98.9077	99.19663
3845	99.6617	99.17874	98.90907	99.20093
3844	99.65917	99.17177	98.91056	99.20525
3843	99.65631	99.16503	98.91216	99.20959
3842	99.65333	99.15851	98.91387	99.21396
3841	99.65043	99.15222	98.91567	99.21834

3840	99.64776	99.14614	98.91758	99.22274
3839	99.64537	99.14028	98.91958	99.22715
3838	99.64324	99.13463	98.92168	99.23158
3837	99.64134	99.1292	98.92387	99.23601
3836	99.63958	99.12398	98.92615	99.24044
3835	99.63794	99.11896	98.92851	99.24488
3834	99.63637	99.11415	98.93095	99.24931
3833	99.63484	99.10955	98.93347	99.25374
3832	99.63333	99.10515	98.93606	99.25817
3831	99.63179	99.10094	98.93873	99.26259
3830	99.63023	99.09694	98.94147	99.267
3829	99.62868	99.09313	98.94428	99.2714
3828	99.62718	99.08951	98.94715	99.27578
3827	99.6258	99.08609	98.95008	99.28015
3826	99.62457	99.08285	98.95308	99.28449
3825	99.62348	99.0798	98.95613	99.28882
3824	99.62252	99.07693	98.95923	99.29312
3823	99.62162	99.07425	98.96238	99.2974
3822	99.62074	99.07174	98.96558	99.30165
3821	99.61986	99.06941	98.96883	99.30587
3820	99.61897	99.06726	98.97212	99.31006
3819	99.61811	99.06529	98.97546	99.31422
3818	99.61731	99.06348	98.97883	99.31834
3817	99.61659	99.06184	98.98224	99.32243
3816	99.61551	99.06038	98.98568	99.32647
3815	99.6151	99.05907	98.98915	99.33048
3814	99.61515	99.05793	98.99265	99.33445
3813	99.61554	99.05695	98.99618	99.33837
3812	99.61617	99.05613	98.99973	99.34225
3811	99.61697	99.05546	99.00331	99.34608
3810	99.61785	99.05495	99.00691	99.34986
3809	99.61873	99.05459	99.01052	99.35359
3808	99.61947	99.05438	99.01415	99.35728
3807	99.61992	99.05432	99.0178	99.36091
3806	99.62001	99.0544	99.02145	99.36448
3805	99.6197	99.05463	99.02512	99.36801
3804	99.61907	99.055	99.02879	99.37147
3803	99.61827	99.0555	99.03247	99.37488
3802	99.61748	99.05615	99.03616	99.37824
3801	99.61687	99.05693	99.03984	99.38153
3800	99.61653	99.05784	99.04353	99.38476

3799	99.61652	99.05889	99.04721	99.38793
3798	99.61683	99.06006	99.0509	99.39104
3797	99.61739	99.06136	99.05457	99.39408
3796	99.61811	99.06279	99.05825	99.39706
3795	99.61887	99.06434	99.06191	99.39998
3794	99.61949	99.06601	99.06556	99.40283
3793	99.61979	99.06779	99.0692	99.40561
3792	99.61952	99.0697	99.07283	99.40833
3791	99.61838	99.07171	99.07644	99.41097
3790	99.61598	99.07384	99.08004	99.41355
3789	99.61266	99.07609	99.08362	99.41606
3788	99.60839	99.07844	99.08718	99.4185
3787	99.60315	99.08089	99.09072	99.42087
3786	99.5969	99.08345	99.09424	99.42317
3785	99.58965	99.08611	99.09774	99.4254
3784	99.58139	99.08888	99.10121	99.42755
3783	99.57219	99.09174	99.10466	99.42963
3782	99.56221	99.09469	99.10808	99.43164
3781	99.5517	99.09775	99.11147	99.43358
3780	99.541	99.10089	99.11483	99.43545
3779	99.53052	99.10413	99.11817	99.43724
3778	99.52066	99.10745	99.12147	99.43896
3777	99.51177	99.11086	99.12474	99.4406
3776	99.50417	99.11435	99.12797	99.44217
3775	99.49805	99.11793	99.13117	99.44367
3774	99.49355	99.12159	99.13434	99.44509
3773	99.49071	99.12533	99.13747	99.44644
3772	99.48947	99.12914	99.14056	99.44772
3771	99.48968	99.13303	99.14361	99.44892
3770	99.4911	99.13699	99.14663	99.45005
3769	99.49353	99.14102	99.1496	99.45111
3768	99.49682	99.14513	99.15254	99.45209
3767	99.50095	99.1493	99.15543	99.453
3766	99.50603	99.15353	99.15828	99.45384
3765	99.51224	99.15783	99.16109	99.4546
3764	99.51976	99.1622	99.16385	99.4553
3763	99.5287	99.16662	99.16657	99.45592
3762	99.53901	99.1711	99.16925	99.45647
3761	99.55047	99.17564	99.17188	99.45694
3760	99.56271	99.18023	99.17446	99.45735
3759	99.57529	99.18488	99.177	99.45769

3758	99.58774	99.18958	99.17949	99.45795
3757	99.59963	99.19432	99.18194	99.45815
3756	99.6106	99.19912	99.18434	99.45828
3755	99.62033	99.20396	99.18668	99.45834
3754	99.62855	99.20885	99.18898	99.45833
3753	99.635	99.21377	99.19123	99.45826
3752	99.63952	99.21874	99.19344	99.45812
3751	99.64207	99.22375	99.19559	99.45792
3750	99.64274	99.2288	99.19769	99.45764
3749	99.64178	99.23388	99.19974	99.45731
3748	99.63956	99.239	99.20175	99.45691
3747	99.63646	99.24415	99.2037	99.45645
3746	99.63286	99.24933	99.2056	99.45593
3745	99.62904	99.25454	99.20745	99.45534
3744	99.62525	99.25978	99.20925	99.4547
3743	99.62167	99.26504	99.211	99.45399
3742	99.61849	99.27033	99.21269	99.45323
3741	99.61594	99.27564	99.21434	99.45241
3740	99.6142	99.28098	99.21593	99.45153
3739	99.61344	99.28633	99.21747	99.4506
3738	99.61367	99.2917	99.21897	99.44961
3737	99.61471	99.29709	99.22041	99.44857
3736	99.61623	99.3025	99.2218	99.44747
3735	99.61768	99.30792	99.22313	99.44632
3734	99.61836	99.31335	99.22442	99.44512
3733	99.61736	99.31879	99.22566	99.44387
3732	99.61446	99.32424	99.22684	99.44258
3731	99.60963	99.3297	99.22797	99.44123
3730	99.60293	99.33517	99.22906	99.43984
3729	99.59453	99.34064	99.23009	99.4384
3728	99.58463	99.34612	99.23107	99.43691
3727	99.57342	99.3516	99.23201	99.43539
3726	99.56109	99.35708	99.23289	99.43382
3725	99.54776	99.36256	99.23372	99.4322
3724	99.53348	99.36803	99.23451	99.43055
3723	99.51817	99.37351	99.23524	99.42886
3722	99.5017	99.37898	99.23593	99.42713
3721	99.48391	99.38445	99.23657	99.42537
3720	99.46468	99.38991	99.23716	99.42356
3719	99.44402	99.39536	99.2377	99.42173
3718	99.42212	99.4008	99.2382	99.41986

3717	99.39934	99.40623	99.23865	99.41796
3716	99.37612	99.41165	99.23905	99.41602
3715	99.35298	99.41706	99.23941	99.41406
3714	99.33035	99.42245	99.23972	99.41207
3713	99.30854	99.42783	99.23998	99.41005
3712	99.28773	99.43319	99.24021	99.40801
3711	99.26802	99.43853	99.24038	99.40594
3710	99.24944	99.44385	99.24052	99.40384
3709	99.23204	99.44916	99.24061	99.40173
3708	99.21585	99.45444	99.24065	99.39959
3707	99.20088	99.4597	99.24066	99.39743
3706	99.18709	99.46494	99.24062	99.39525
3705	99.17436	99.47015	99.24054	99.39306
3704	99.16251	99.47534	99.24042	99.39085
3703	99.15126	99.4805	99.24027	99.38862
3702	99.1403	99.48564	99.24007	99.38638
3701	99.12926	99.49074	99.23983	99.38412
3700	99.11779	99.49582	99.23955	99.38186
3699	99.10552	99.50086	99.23924	99.37958
3698	99.09209	99.50588	99.23889	99.3773
3697	99.07718	99.51086	99.2385	99.375
3696	99.06053	99.51581	99.23808	99.3727
3695	99.04198	99.52072	99.23762	99.37039
3694	99.0215	99.5256	99.23713	99.36808
3693	98.99924	99.53044	99.2366	99.36577
3692	98.97544	99.53525	99.23604	99.36345
3691	98.95036	99.54002	99.23545	99.36113
3690	98.9242	99.54475	99.23482	99.35881
3689	98.89707	99.54944	99.23416	99.35649
3688	98.86887	99.55409	99.23348	99.35417
3687	98.83941	99.5587	99.23276	99.35186
3686	98.80843	99.56327	99.23201	99.34955
3685	98.77567	99.56779	99.23124	99.34725
3684	98.74099	99.57227	99.23043	99.34495
3683	98.70439	99.57671	99.2296	99.34266
3682	98.66595	99.5811	99.22874	99.34038
3681	98.62588	99.58545	99.22786	99.33811
3680	98.58442	99.58975	99.22695	99.33584
3679	98.54178	99.59401	99.22601	99.3336
3678	98.49813	99.59821	99.22505	99.33136
3677	98.45356	99.60237	99.22407	99.32913

3676	98.40807	99.60648	99.22307	99.32693
3675	98.36154	99.61054	99.22204	99.32473
3674	98.31372	99.61455	99.22099	99.32255
3673	98.26428	99.61851	99.21992	99.32039
3672	98.21284	99.62242	99.21884	99.31825
3671	98.159	99.62627	99.21773	99.31613
3670	98.10239	99.63008	99.2166	99.31403
3669	98.04273	99.63383	99.21546	99.31195
3668	97.97982	99.63753	99.2143	99.30989
3667	97.91354	99.64117	99.21313	99.30785
3666	97.8438	99.64476	99.21193	99.30583
3665	97.77059	99.64829	99.21073	99.30384
3664	97.69389	99.65177	99.20951	99.30188
3663	97.61376	99.6552	99.20827	99.29994
3662	97.53026	99.65857	99.20703	99.29803
3661	97.44349	99.66188	99.20577	99.29614
3660	97.35357	99.66513	99.2045	99.29428
3659	97.26071	99.66833	99.20321	99.29245
3658	97.16518	99.67147	99.20192	99.29065
3657	97.06731	99.67455	99.20062	99.28888
3656	96.96747	99.67757	99.19931	99.28714
3655	96.86603	99.68054	99.198	99.28544
3654	96.76336	99.68344	99.19667	99.28376
3653	96.65973	99.68629	99.19534	99.28211
3652	96.55537	99.68907	99.194	99.2805
3651	96.45041	99.6918	99.19266	99.27892
3650	96.34486	99.69447	99.19131	99.27738
3649	96.23867	99.69707	99.18996	99.27587
3648	96.13169	99.69962	99.1886	99.27439
3647	96.02379	99.7021	99.18725	99.27295
3646	95.91482	99.70453	99.18589	99.27155
3645	95.80469	99.70689	99.18452	99.27018
3644	95.69338	99.70919	99.18316	99.26885
3643	95.58091	99.71143	99.1818	99.26755
3642	95.46735	99.71361	99.18043	99.2663
3641	95.35268	99.71573	99.17907	99.26507
3640	95.23682	99.71778	99.17771	99.26389
3639	95.11957	99.71978	99.17635	99.26275
3638	95.00059	99.72171	99.17499	99.26164
3637	94.87951	99.72358	99.17364	99.26057
3636	94.75595	99.72539	99.17229	99.25955

3635	94.62959	99.72713	99.17094	99.25856
3634	94.50023	99.72881	99.1696	99.25761
3633	94.36777	99.73043	99.16826	99.2567
3632	94.23224	99.73199	99.16693	99.25583
3631	94.09374	99.73349	99.16561	99.25499
3630	93.95242	99.73492	99.16429	99.2542
3629	93.80848	99.73629	99.16298	99.25345
3628	93.66212	99.7376	99.16168	99.25274
3627	93.51352	99.73885	99.16038	99.25207
3626	93.36288	99.74004	99.1591	99.25144
3625	93.21036	99.74116	99.15782	99.25084
3624	93.05617	99.74222	99.15656	99.25029
3623	92.90055	99.74322	99.1553	99.24978
3622	92.7437	99.74416	99.15405	99.24931
3621	92.58581	99.74504	99.15282	99.24888
3620	92.42698	99.74586	99.15159	99.24848
3619	92.26724	99.74661	99.15038	99.24813
3618	92.10656	99.7473	99.14918	99.24782
3617	91.94483	99.74794	99.148	99.24754
3616	91.78191	99.74851	99.14682	99.24731
3615	91.61765	99.74902	99.14566	99.24711
3614	91.45185	99.74947	99.14451	99.24696
3613	91.28435	99.74986	99.14338	99.24684
3612	91.11498	99.7502	99.14226	99.24676
3611	90.9436	99.75047	99.14116	99.24672
3610	90.77006	99.75068	99.14007	99.24671
3609	90.59423	99.75084	99.139	99.24675
3608	90.41596	99.75093	99.13794	99.24682
3607	90.23511	99.75097	99.1369	99.24692
3606	90.05156	99.75095	99.13587	99.24707
3605	89.86522	99.75087	99.13486	99.24725
3604	89.67606	99.75073	99.13387	99.24746
3603	89.48409	99.75054	99.1329	99.24771
3602	89.28943	99.75029	99.13194	99.248
3601	89.09221	99.74998	99.131	99.24832
3600	88.89259	99.74962	99.13008	99.24868
3599	88.69074	99.7492	99.12917	99.24907
3598	88.4868	99.74872	99.12829	99.24949
3597	88.28088	99.74819	99.12742	99.24994
3596	88.0731	99.74761	99.12657	99.25043
3595	87.86356	99.74697	99.12574	99.25095

3594	87.65242	99.74628	99.12493	99.2515
3593	87.43983	99.74553	99.12414	99.25208
3592	87.22599	99.74474	99.12337	99.25269
3591	87.01112	99.74388	99.12262	99.25334
3590	86.79538	99.74298	99.12188	99.25401
3589	86.57894	99.74203	99.12117	99.25471
3588	86.36193	99.74102	99.12048	99.25543
3587	86.14441	99.73996	99.1198	99.25619
3586	85.92644	99.73886	99.11915	99.25697
3585	85.70801	99.7377	99.11851	99.25778
3584	85.48913	99.73649	99.1179	99.25861
3583	85.26978	99.73524	99.11731	99.25947
3582	85.04996	99.73393	99.11674	99.26035
3581	84.82966	99.73258	99.11618	99.26126
3580	84.60886	99.73119	99.11565	99.26219
3579	84.38753	99.72974	99.11514	99.26314
3578	84.16565	99.72825	99.11465	99.26412
3577	83.94322	99.72671	99.11418	99.26511
3576	83.72028	99.72513	99.11373	99.26613
3575	83.4969	99.7235	99.1133	99.26716
3574	83.2732	99.72183	99.11289	99.26822
3573	83.0493	99.72012	99.1125	99.26929
3572	82.82532	99.71836	99.11213	99.27038
3571	82.60138	99.71656	99.11179	99.27149
3570	82.37759	99.71472	99.11146	99.27261
3569	82.15405	99.71284	99.11115	99.27375
3568	81.93087	99.71092	99.11087	99.2749
3567	81.70818	99.70896	99.1106	99.27607
3566	81.48617	99.70696	99.11035	99.27726
3565	81.26507	99.70492	99.11013	99.27845
3564	81.04512	99.70284	99.10992	99.27966
3563	80.82658	99.70072	99.10973	99.28087
3562	80.60967	99.69857	99.10956	99.2821
3561	80.39458	99.69638	99.10942	99.28334
3560	80.18148	99.69416	99.10929	99.28459
3559	79.97045	99.6919	99.10918	99.28584
3558	79.76155	99.68961	99.10909	99.28711
3557	79.55479	99.68728	99.10902	99.28838
3556	79.35014	99.68492	99.10897	99.28965
3555	79.14758	99.68253	99.10894	99.29094
3554	78.9471	99.6801	99.10892	99.29222

3553	78.74871	99.67765	99.10892	99.29352
3552	78.55245	99.67516	99.10895	99.29481
3551	78.35841	99.67265	99.10899	99.29611
3550	78.1667	99.6701	99.10905	99.29741
3549	77.97742	99.66753	99.10912	99.29871
3548	77.7907	99.66493	99.10921	99.30001
3547	77.6066	99.6623	99.10933	99.30131
3546	77.42515	99.65965	99.10945	99.30261
3545	77.24634	99.65697	99.1096	99.30391
3544	77.07009	99.65427	99.10976	99.3052
3543	76.89633	99.65154	99.10994	99.3065
3542	76.725	99.64879	99.11013	99.30779
3541	76.55603	99.64601	99.11034	99.30907
3540	76.38944	99.64321	99.11056	99.31035
3539	76.22524	99.6404	99.1108	99.31163
3538	76.06351	99.63756	99.11106	99.3129
3537	75.90434	99.6347	99.11133	99.31416
3536	75.74783	99.63182	99.11162	99.31542
3535	75.59407	99.62892	99.11192	99.31666
3534	75.44312	99.626	99.11223	99.3179
3533	75.29503	99.62307	99.11256	99.31913
3532	75.14985	99.62012	99.1129	99.32035
3531	75.00763	99.61715	99.11325	99.32156
3530	74.86841	99.61417	99.11362	99.32276
3529	74.73227	99.61117	99.114	99.32394
3528	74.59929	99.60816	99.1144	99.32512
3527	74.46953	99.60514	99.1148	99.32628
3526	74.34301	99.6021	99.11522	99.32743
3525	74.21969	99.59905	99.11565	99.32857
3524	74.09947	99.596	99.11609	99.32969
3523	73.98218	99.59293	99.11654	99.33079
3522	73.86762	99.58985	99.117	99.33188
3521	73.75555	99.58676	99.11747	99.33296
3520	73.64577	99.58366	99.11795	99.33402
3519	73.53808	99.58056	99.11845	99.33506
3518	73.43234	99.57744	99.11895	99.33609
3517	73.32845	99.57433	99.11946	99.33709
3516	73.22629	99.5712	99.11998	99.33809
3515	73.1258	99.56807	99.12051	99.33906
3514	73.0269	99.56494	99.12104	99.34001
3513	72.92956	99.56181	99.12159	99.34094

3512	72.83372	99.55867	99.12214	99.34186
3511	72.73937	99.55552	99.1227	99.34275
3510	72.64652	99.55238	99.12326	99.34363
3509	72.55517	99.54924	99.12384	99.34448
3508	72.4653	99.54609	99.12442	99.34532
3507	72.37689	99.54295	99.125	99.34613
3506	72.2899	99.53981	99.12559	99.34692
3505	72.20427	99.53666	99.12619	99.34769
3504	72.11993	99.53353	99.12679	99.34844
3503	72.03686	99.53039	99.1274	99.34916
3502	71.95504	99.52726	99.12801	99.34986
3501	71.87455	99.52413	99.12862	99.35054
3500	71.79549	99.52101	99.12924	99.3512
3499	71.71799	99.51789	99.12986	99.35183
3498	71.64222	99.51478	99.13049	99.35244
3497	71.56831	99.51167	99.13111	99.35303
3496	71.49633	99.50858	99.13174	99.35359
3495	71.4263	99.50549	99.13238	99.35413
3494	71.35814	99.50241	99.13301	99.35465
3493	71.29172	99.49934	99.13365	99.35514
3492	71.22684	99.49628	99.13429	99.35561
3491	71.16329	99.49323	99.13493	99.35605
3490	71.10089	99.49019	99.13556	99.35647
3489	71.03945	99.48716	99.1362	99.35686
3488	70.97882	99.48415	99.13684	99.35723
3487	70.91888	99.48114	99.13748	99.35758
3486	70.85949	99.47815	99.13812	99.3579
3485	70.80056	99.47518	99.13876	99.3582
3484	70.74198	99.47222	99.1394	99.35847
3483	70.68369	99.46928	99.14004	99.35872
3482	70.62568	99.46635	99.14067	99.35894
3481	70.56801	99.46343	99.1413	99.35914
3480	70.51079	99.46054	99.14193	99.35931
3479	70.45418	99.45766	99.14256	99.35946
3478	70.39836	99.4548	99.14319	99.35959
3477	70.34348	99.45196	99.14381	99.35969
3476	70.28968	99.44914	99.14443	99.35977
3475	70.23707	99.44633	99.14505	99.35983
3474	70.18574	99.44355	99.14566	99.35986
3473	70.1358	99.44079	99.14627	99.35986
3472	70.08736	99.43805	99.14687	99.35985

3471	70.04056	99.43533	99.14747	99.35981
3470	69.99554	99.43263	99.14806	99.35975
3469	69.95242	99.42995	99.14865	99.35966
3468	69.91129	99.4273	99.14924	99.35955
3467	69.87217	99.42467	99.14982	99.35942
3466	69.83509	99.42206	99.15039	99.35927
3465	69.8	99.41948	99.15096	99.3591
3464	69.76687	99.41693	99.15152	99.3589
3463	69.73567	99.4144	99.15208	99.35868
3462	69.70634	99.41189	99.15263	99.35845
3461	69.67883	99.40941	99.15317	99.35819
3460	69.65303	99.40696	99.1537	99.35791
3459	69.62882	99.40454	99.15423	99.35761
3458	69.60603	99.40214	99.15475	99.35729
3457	69.58449	99.39977	99.15527	99.35695
3456	69.564	99.39743	99.15577	99.35659
3455	69.54442	99.39511	99.15627	99.35621
3454	69.5256	99.39283	99.15676	99.35581
3453	69.50749	99.39058	99.15724	99.3554
3452	69.49007	99.38835	99.15771	99.35497
3451	69.47339	99.38616	99.15818	99.35452
3450	69.45759	99.38399	99.15863	99.35405
3449	69.44284	99.38186	99.15908	99.35357
3448	69.42934	99.37975	99.15952	99.35306
3447	69.41734	99.37768	99.15995	99.35255
3446	69.40705	99.37564	99.16037	99.35202
3445	69.39866	99.37364	99.16078	99.35147
3444	69.39232	99.37166	99.16118	99.35091
3443	69.3881	99.36972	99.16157	99.35033
3442	69.38607	99.36781	99.16195	99.34974
3441	69.38623	99.36593	99.16233	99.34914
3440	69.38855	99.36409	99.16269	99.34852
3439	69.39302	99.36228	99.16304	99.3479
3438	69.39959	99.36051	99.16338	99.34725
3437	69.4082	99.35877	99.16371	99.3466
3436	69.41881	99.35706	99.16404	99.34594
3435	69.43136	99.35539	99.16435	99.34527
3434	69.4458	99.35376	99.16465	99.34458
3433	69.46213	99.35216	99.16494	99.34389
3432	69.48033	99.35059	99.16522	99.34319
3431	69.5004	99.34906	99.16549	99.34247

3430	69.52234	99.34757	99.16575	99.34175
3429	69.54608	99.34611	99.16599	99.34103
3428	69.57156	99.34469	99.16623	99.34029
3427	69.59864	99.3433	99.16646	99.33955
3426	69.6272	99.34196	99.16667	99.3388
3425	69.65713	99.34064	99.16687	99.33805
3424	69.68833	99.33937	99.16707	99.33729
3423	69.72076	99.33813	99.16725	99.33653
3422	69.75444	99.33693	99.16742	99.33576
3421	69.78941	99.33577	99.16758	99.33499
3420	69.82574	99.33464	99.16773	99.33421
3419	69.8635	99.33355	99.16787	99.33343
3418	69.9028	99.3325	99.168	99.33265
3417	69.94375	99.33149	99.16812	99.33187
3416	69.98647	99.33052	99.16822	99.33109
3415	70.03113	99.32958	99.16832	99.3303
3414	70.07785	99.32868	99.1684	99.32952
3413	70.12676	99.32782	99.16847	99.32874
3412	70.17793	99.327	99.16854	99.32795
3411	70.23138	99.32621	99.16859	99.32717
3410	70.28707	99.32547	99.16863	99.32639
3409	70.34491	99.32476	99.16866	99.32561
3408	70.40478	99.32409	99.16868	99.32483
3407	70.46659	99.32346	99.16869	99.32406
3406	70.53024	99.32286	99.16869	99.32329
3405	70.59563	99.32231	99.16868	99.32252
3404	70.66268	99.32179	99.16865	99.32176
3403	70.73127	99.32131	99.16862	99.321
3402	70.80129	99.32087	99.16858	99.32025
3401	70.87261	99.32047	99.16853	99.31951
3400	70.94507	99.3201	99.16846	99.31877
3399	71.01855	99.31978	99.16839	99.31803
3398	71.09296	99.31949	99.16831	99.31731
3397	71.16823	99.31924	99.16822	99.31659
3396	71.24433	99.31903	99.16811	99.31588
3395	71.32129	99.31885	99.168	99.31517
3394	71.39915	99.31872	99.16788	99.31448
3393	71.47799	99.31862	99.16775	99.31379
3392	71.55788	99.31856	99.16761	99.31312
3391	71.63887	99.31854	99.16746	99.31245
3390	71.72101	99.31855	99.1673	99.3118

3389	71.80429	99.3186	99.16714	99.31115
3388	71.88864	99.31869	99.16696	99.31052
3387	71.974	99.31881	99.16678	99.30989
3386	72.06024	99.31898	99.16658	99.30928
3385	72.14727	99.31917	99.16638	99.30868
3384	72.23499	99.31941	99.16617	99.30809
3383	72.32332	99.31968	99.16596	99.30752
3382	72.41222	99.31999	99.16573	99.30696
3381	72.50161	99.32033	99.1655	99.30641
3380	72.59142	99.32071	99.16526	99.30587
3379	72.68155	99.32113	99.16501	99.30535
3378	72.77186	99.32158	99.16476	99.30484
3377	72.86221	99.32207	99.16449	99.30435
3376	72.95248	99.32259	99.16423	99.30387
3375	73.04253	99.32314	99.16395	99.3034
3374	73.1323	99.32374	99.16367	99.30296
3373	73.22171	99.32436	99.16338	99.30252
3372	73.31072	99.32502	99.16308	99.3021
3371	73.39929	99.32571	99.16278	99.3017
3370	73.4874	99.32644	99.16247	99.30131
3369	73.57504	99.3272	99.16216	99.30094
3368	73.66222	99.328	99.16184	99.30059
3367	73.74895	99.32882	99.16152	99.30025
3366	73.83527	99.32968	99.16119	99.29992
3365	73.92125	99.33058	99.16085	99.29962
3364	74.0069	99.3315	99.16051	99.29933
3363	74.09224	99.33246	99.16017	99.29906
3362	74.17724	99.33344	99.15982	99.2988
3361	74.26186	99.33446	99.15947	99.29856
3360	74.34604	99.33551	99.15911	99.29834
3359	74.42974	99.33659	99.15875	99.29814
3358	74.51298	99.3377	99.15838	99.29795
3357	74.59581	99.33885	99.15801	99.29778
3356	74.67833	99.34002	99.15764	99.29763
3355	74.76067	99.34122	99.15727	99.2975
3354	74.84299	99.34245	99.15689	99.29738
3353	74.92543	99.3437	99.15651	99.29728
3352	75.00807	99.34499	99.15612	99.2972
3351	75.09092	99.3463	99.15574	99.29714
3350	75.17393	99.34765	99.15535	99.29709
3349	75.25693	99.34902	99.15496	99.29706

3348	75.33968	99.35041	99.15457	99.29705
3347	75.4219	99.35183	99.15417	99.29706
3346	75.50334	99.35328	99.15378	99.29708
3345	75.58376	99.35476	99.15338	99.29712
3344	75.66301	99.35626	99.15298	99.29718
3343	75.74106	99.35779	99.15258	99.29726
3342	75.81795	99.35934	99.15218	99.29735
3341	75.89378	99.36091	99.15179	99.29746
3340	75.96869	99.36251	99.15138	99.29758
3339	76.04285	99.36413	99.15098	99.29773
3338	76.1164	99.36578	99.15058	99.29788
3337	76.18949	99.36744	99.15018	99.29806
3336	76.26225	99.36913	99.14979	99.29825
3335	76.33484	99.37085	99.14939	99.29846
3334	76.40738	99.37258	99.14899	99.29868
3333	76.48001	99.37434	99.14859	99.29892
3332	76.55277	99.37611	99.1482	99.29918
3331	76.62567	99.37791	99.1478	99.29945
3330	76.69864	99.37972	99.14741	99.29974
3329	76.77153	99.38156	99.14702	99.30004
3328	76.84417	99.38341	99.14663	99.30035
3327	76.91638	99.38528	99.14624	99.30068
3326	76.98802	99.38718	99.14585	99.30103
3325	77.059	99.38908	99.14547	99.30138
3324	77.12929	99.39101	99.14509	99.30176
3323	77.1989	99.39295	99.14471	99.30214
3322	77.26791	99.39491	99.14434	99.30254
3321	77.33642	99.39689	99.14397	99.30295
3320	77.40453	99.39888	99.1436	99.30337
3319	77.47237	99.40088	99.14324	99.30381
3318	77.54003	99.4029	99.14287	99.30426
3317	77.60758	99.40494	99.14252	99.30472
3316	77.67506	99.40698	99.14216	99.30519
3315	77.74245	99.40904	99.14181	99.30568
3314	77.80964	99.41112	99.14147	99.30617
3313	77.8765	99.4132	99.14113	99.30667
3312	77.9428	99.4153	99.14079	99.30719
3311	78.00832	99.41741	99.14046	99.30771
3310	78.07278	99.41953	99.14013	99.30825
3309	78.13596	99.42166	99.13981	99.30879
3308	78.19767	99.4238	99.13949	99.30934

3307	78.25783	99.42595	99.13918	99.3099
3306	78.31651	99.42811	99.13888	99.31047
3305	78.37389	99.43028	99.13857	99.31104
3304	78.43028	99.43246	99.13828	99.31163
3303	78.4861	99.43464	99.13799	99.31222
3302	78.54177	99.43683	99.1377	99.31281
3301	78.59769	99.43903	99.13743	99.31342
3300	78.65418	99.44123	99.13715	99.31403
3299	78.71141	99.44344	99.13689	99.31464
3298	78.76946	99.44566	99.13663	99.31526
3297	78.82832	99.44788	99.13637	99.31588
3296	78.88792	99.4501	99.13613	99.31651
3295	78.94818	99.45233	99.13589	99.31714
3294	79.00906	99.45456	99.13565	99.31778
3293	79.07052	99.4568	99.13542	99.31842
3292	79.13256	99.45904	99.1352	99.31906
3291	79.19518	99.46128	99.13499	99.3197
3290	79.25838	99.46352	99.13478	99.32035
3289	79.32216	99.46576	99.13458	99.32099
3288	79.38654	99.468	99.13439	99.32164
3287	79.45159	99.47025	99.13421	99.32229
3286	79.51739	99.47249	99.13403	99.32294
3285	79.5841	99.47473	99.13386	99.32359
3284	79.65189	99.47698	99.13369	99.32423
3283	79.72096	99.47922	99.13354	99.32488
3282	79.79148	99.48145	99.13339	99.32553
3281	79.8636	99.48369	99.13325	99.32617
3280	79.93743	99.48592	99.13312	99.32681
3279	80.01302	99.48815	99.13299	99.32745
3278	80.09034	99.49038	99.13287	99.32809
3277	80.1693	99.4926	99.13276	99.32872
3276	80.24977	99.49482	99.13266	99.32935
3275	80.33153	99.49703	99.13257	99.32998
3274	80.41439	99.49924	99.13248	99.3306
3273	80.49817	99.50144	99.1324	99.33122
3272	80.58274	99.50363	99.13233	99.33183
3271	80.66807	99.50582	99.13227	99.33244
3270	80.75427	99.508	99.13221	99.33304
3269	80.84151	99.51018	99.13217	99.33363
3268	80.93006	99.51234	99.13213	99.33422
3267	81.02021	99.5145	99.1321	99.3348

3266	81.11226	99.51665	99.13207	99.33537
3265	81.20643	99.51879	99.13206	99.33594
3264	81.3029	99.52092	99.13205	99.3365
3263	81.40171	99.52303	99.13205	99.33705
3262	81.50283	99.52514	99.13206	99.33759
3261	81.60607	99.52724	99.13208	99.33812
3260	81.71121	99.52933	99.1321	99.33865
3259	81.81792	99.5314	99.13213	99.33916
3258	81.92589	99.53347	99.13218	99.33966
3257	82.03484	99.53552	99.13222	99.34016
3256	82.14455	99.53756	99.13228	99.34064
3255	82.2549	99.53958	99.13234	99.34111
3254	82.36584	99.54159	99.13241	99.34158
3253	82.47736	99.54359	99.13249	99.34203
3252	82.58947	99.54557	99.13258	99.34247
3251	82.70213	99.54754	99.13267	99.34289
3250	82.81524	99.54949	99.13278	99.34331
3249	82.92866	99.55143	99.13289	99.34371
3248	83.04222	99.55336	99.133	99.3441
3247	83.15578	99.55526	99.13313	99.34448
3246	83.26925	99.55715	99.13326	99.34485
3245	83.38266	99.55903	99.1334	99.3452
3244	83.49608	99.56089	99.13354	99.34554
3243	83.60969	99.56273	99.1337	99.34587
3242	83.72369	99.56455	99.13386	99.34618
3241	83.83827	99.56635	99.13402	99.34648
3240	83.95358	99.56814	99.1342	99.34676
3239	84.06969	99.56991	99.13438	99.34703
3238	84.18655	99.57165	99.13457	99.34729
3237	84.30408	99.57338	99.13476	99.34753
3236	84.42209	99.57509	99.13496	99.34776
3235	84.54038	99.57678	99.13517	99.34797
3234	84.65879	99.57846	99.13538	99.34817
3233	84.77723	99.58011	99.1356	99.34835
3232	84.89569	99.58174	99.13583	99.34852
3231	85.0143	99.58334	99.13606	99.34868
3230	85.13326	99.58493	99.1363	99.34881
3229	85.25277	99.5865	99.13654	99.34894
3228	85.37302	99.58804	99.13679	99.34905
3227	85.49408	99.58957	99.13705	99.34914
3226	85.61592	99.59107	99.13731	99.34922

3225	85.73841	99.59255	99.13758	99.34928
3224	85.86133	99.594	99.13785	99.34933
3223	85.98447	99.59544	99.13813	99.34936
3222	86.10762	99.59685	99.13841	99.34938
3221	86.23067	99.59824	99.1387	99.34938
3220	86.35358	99.5996	99.13899	99.34936
3219	86.47637	99.60094	99.13929	99.34933
3218	86.59912	99.60226	99.13959	99.34929
3217	86.72196	99.60355	99.1399	99.34923
3216	86.84501	99.60482	99.14021	99.34916
3215	86.96836	99.60607	99.14053	99.34907
3214	87.09208	99.60729	99.14085	99.34896
3213	87.21618	99.60848	99.14117	99.34885
3212	87.3406	99.60965	99.1415	99.34871
3211	87.46522	99.6108	99.14183	99.34856
3210	87.58989	99.61192	99.14216	99.3484
3209	87.71445	99.61302	99.1425	99.34822
3208	87.83884	99.61409	99.14284	99.34803
3207	87.96304	99.61513	99.14319	99.34783
3206	88.08718	99.61615	99.14354	99.34761
3205	88.21145	99.61714	99.14389	99.34738
3204	88.3361	99.61811	99.14424	99.34713
3203	88.46134	99.61905	99.1446	99.34687
3202	88.58731	99.61997	99.14496	99.3466
3201	88.71402	99.62085	99.14532	99.34631
3200	88.84132	99.62172	99.14568	99.34601
3199	88.96895	99.62255	99.14605	99.3457
3198	89.09652	99.62336	99.14642	99.34538
3197	89.22363	99.62415	99.14679	99.34504
3196	89.3499	99.6249	99.14716	99.3447
3195	89.475	99.62563	99.14753	99.34434
3194	89.59875	99.62634	99.14791	99.34397
3193	89.72106	99.62701	99.14828	99.34359
3192	89.84198	99.62766	99.14866	99.34319
3191	89.9616	99.62829	99.14904	99.34279
3190	90.08008	99.62888	99.14942	99.34238
3189	90.19755	99.62945	99.1498	99.34196
3188	90.31406	99.62999	99.15018	99.34152
3187	90.4296	99.63051	99.15056	99.34108
3186	90.54412	99.631	99.15094	99.34063
3185	90.65753	99.63146	99.15132	99.34017

3184	90.76981	99.63189	99.1517	99.3397
3183	90.88098	99.6323	99.15208	99.33923
3182	90.99119	99.63268	99.15247	99.33874
3181	91.1006	99.63303	99.15285	99.33825
3180	91.20945	99.63336	99.15323	99.33775
3179	91.31791	99.63366	99.15361	99.33725
3178	91.42609	99.63393	99.15398	99.33674
3177	91.53397	99.63418	99.15436	99.33622
3176	91.64148	99.6344	99.15474	99.3357
3175	91.74846	99.63459	99.15512	99.33517
3174	91.85473	99.63475	99.15549	99.33464
3173	91.96013	99.63489	99.15586	99.3341
3172	92.06454	99.63501	99.15623	99.33356
3171	92.16793	99.63509	99.1566	99.33301
3170	92.2703	99.63515	99.15697	99.33247
3169	92.37176	99.63519	99.15734	99.33191
3168	92.47248	99.63519	99.1577	99.33136
3167	92.57266	99.63518	99.15806	99.3308
3166	92.6725	99.63513	99.15842	99.33025
3165	92.77219	99.63506	99.15878	99.32969
3164	92.87182	99.63497	99.15913	99.32913
3163	92.97135	99.63484	99.15948	99.32857
3162	93.07062	99.6347	99.15983	99.32801
3161	93.16938	99.63452	99.16018	99.32745
3160	93.2673	99.63433	99.16052	99.32689
3159	93.36406	99.6341	99.16086	99.32633
3158	93.4594	99.63386	99.1612	99.32578
3157	93.55318	99.63358	99.16153	99.32522
3156	93.64534	99.63329	99.16186	99.32467
3155	93.73599	99.63296	99.16219	99.32412
3154	93.82531	99.63262	99.16251	99.32358
3153	93.91355	99.63225	99.16283	99.32304
3152	94.00104	99.63185	99.16315	99.3225
3151	94.08804	99.63144	99.16346	99.32197
3150	94.17483	99.63099	99.16377	99.32144
3149	94.26159	99.63053	99.16407	99.32092
3148	94.34841	99.63004	99.16437	99.3204
3147	94.43528	99.62953	99.16466	99.31989
3146	94.52212	99.629	99.16495	99.31939
3145	94.60878	99.62844	99.16524	99.31889
3144	94.69512	99.62786	99.16552	99.31841

3143	94.78102	99.62726	99.1658	99.31793
3142	94.86637	99.62664	99.16607	99.31745
3141	94.95111	99.62599	99.16634	99.31699
3140	95.03518	99.62532	99.1666	99.31654
3139	95.1185	99.62464	99.16686	99.31609
3138	95.20098	99.62393	99.16711	99.31566
3137	95.28248	99.6232	99.16736	99.31523
3136	95.3629	99.62245	99.16761	99.31482
3135	95.44214	99.62168	99.16785	99.31441
3134	95.52019	99.62089	99.16808	99.31402
3133	95.59711	99.62008	99.16831	99.31364
3132	95.673	99.61925	99.16853	99.31328
3131	95.74799	99.6184	99.16875	99.31292
3130	95.82225	99.61753	99.16896	99.31258
3129	95.89586	99.61664	99.16917	99.31225
3128	95.96891	99.61574	99.16937	99.31194
3127	96.04139	99.61481	99.16956	99.31164
3126	96.11325	99.61387	99.16975	99.31135
3125	96.18437	99.61291	99.16994	99.31108
3124	96.25456	99.61194	99.17012	99.31082
3123	96.32361	99.61095	99.17029	99.31058
3122	96.39128	99.60994	99.17046	99.31036
3121	96.45738	99.60891	99.17063	99.31015
3120	96.5218	99.60787	99.17078	99.30995
3119	96.58458	99.60681	99.17093	99.30978
3118	96.6459	99.60574	99.17108	99.30962
3117	96.70607	99.60465	99.17122	99.30948
3116	96.76547	99.60354	99.17136	99.30935
3115	96.82451	99.60243	99.17149	99.30924
3114	96.88353	99.60129	99.17161	99.30915
3113	96.94276	99.60015	99.17173	99.30908
3112	97.00231	99.59899	99.17184	99.30903
3111	97.06216	99.59781	99.17195	99.30899
3110	97.12217	99.59663	99.17205	99.30898
3109	97.18215	99.59543	99.17215	99.30898
3108	97.24183	99.59422	99.17224	99.309
3107	97.30095	99.593	99.17232	99.30905
3106	97.35926	99.59176	99.1724	99.30911
3105	97.41657	99.59052	99.17247	99.30919
3104	97.47276	99.58926	99.17254	99.30929
3103	97.52779	99.58799	99.17261	99.30941

3102	97.58173	99.58671	99.17266	99.30956
3101	97.63472	99.58543	99.17272	99.30972
3100	97.68696	99.58413	99.17276	99.3099
3099	97.73861	99.58282	99.17281	99.31011
3098	97.78984	99.58151	99.17284	99.31033
3097	97.84075	99.58019	99.17287	99.31058
3096	97.89138	99.57886	99.1729	99.31085
3095	97.94174	99.57752	99.17292	99.31113
3094	97.99179	99.57617	99.17294	99.31144
3093	98.04147	99.57482	99.17295	99.31178
3092	98.0907	99.57346	99.17296	99.31213
3091	98.13935	99.57209	99.17296	99.3125
3090	98.1873	99.57072	99.17295	99.3129
3089	98.23441	99.56934	99.17295	99.31331
3088	98.28056	99.56796	99.17293	99.31375
3087	98.32569	99.56657	99.17292	99.31421
3086	98.36977	99.56518	99.1729	99.31469
3085	98.41286	99.56378	99.17287	99.3152
3084	98.45506	99.56238	99.17284	99.31572
3083	98.49648	99.56097	99.17281	99.31627
3082	98.53728	99.55957	99.17277	99.31684
3081	98.57761	99.55816	99.17272	99.31743
3080	98.61766	99.55675	99.17268	99.31804
3079	98.65766	99.55533	99.17263	99.31867
3078	98.69785	99.55392	99.17257	99.31932
3077	98.73847	99.5525	99.17252	99.31999
3076	98.77969	99.55108	99.17245	99.32069
3075	98.82156	99.54966	99.17239	99.3214
3074	98.86397	99.54824	99.17232	99.32214
3073	98.90666	99.54683	99.17225	99.3229
3072	98.94922	99.54541	99.17217	99.32368
3071	98.99119	99.54399	99.17209	99.32447
3070	99.03214	99.54258	99.17201	99.32529
3069	99.07171	99.54116	99.17193	99.32613
3068	99.10968	99.53975	99.17184	99.32699
3067	99.14598	99.53834	99.17175	99.32787
3066	99.18067	99.53693	99.17166	99.32876
3065	99.2139	99.53553	99.17156	99.32968
3064	99.24591	99.53413	99.17147	99.33062
3063	99.27692	99.53273	99.17137	99.33157
3062	99.30718	99.53134	99.17127	99.33254

3061	99.33687	99.52995	99.17116	99.33354
3060	99.36612	99.52857	99.17106	99.33455
3059	99.39495	99.52719	99.17095	99.33557
3058	99.4233	99.52582	99.17084	99.33662
3057	99.45097	99.52446	99.17073	99.33768
3056	99.4777	99.5231	99.17062	99.33876
3055	99.50317	99.52174	99.1705	99.33986
3054	99.52703	99.5204	99.17039	99.34097
3053	99.54893	99.51906	99.17028	99.3421
3052	99.56852	99.51772	99.17016	99.34324
3051	99.58548	99.5164	99.17004	99.3444
3050	99.59951	99.51508	99.16992	99.34558
3049	99.61033	99.51378	99.16981	99.34677
3048	99.6179	99.51248	99.16969	99.34797
3047	99.62166	99.51119	99.16957	99.34919
3046	99.6216	99.50991	99.16945	99.35042
3045	99.61759	99.50864	99.16933	99.35167
3044	99.60941	99.50738	99.16921	99.35292
3043	99.59681	99.50613	99.16909	99.35419
3042	99.57937	99.5049	99.16898	99.35548
3041	99.55663	99.50367	99.16886	99.35677
3040	99.52798	99.50245	99.16874	99.35808
3039	99.49282	99.50125	99.16862	99.3594
3038	99.45053	99.50006	99.16851	99.36073
3037	99.40051	99.49888	99.1684	99.36206
3036	99.34223	99.49771	99.16828	99.36341
3035	99.27515	99.49656	99.16817	99.36477
3034	99.19877	99.49542	99.16806	99.36614
3033	99.11259	99.49429	99.16795	99.36751
3032	99.01615	99.49318	99.16785	99.36889
3031	98.90908	99.49208	99.16774	99.37028
3030	98.7911	99.49099	99.16764	99.37168
3029	98.66206	99.48992	99.16754	99.37309
3028	98.52193	99.48887	99.16744	99.3745
3027	98.37079	99.48783	99.16734	99.37591
3026	98.20872	99.4868	99.16725	99.37733
3025	98.0358	99.48579	99.16716	99.37876
3024	97.85202	99.4848	99.16707	99.38019
3023	97.65732	99.48382	99.16698	99.38163
3022	97.45154	99.48286	99.1669	99.38306
3021	97.23444	99.48192	99.16682	99.38451

3020	97.00575	99.48099	99.16675	99.38595
3019	96.76516	99.48008	99.16667	99.38739
3018	96.5124	99.47918	99.16661	99.38884
3017	96.24719	99.47831	99.16654	99.39029
3016	95.96934	99.47745	99.16648	99.39174
3015	95.67874	99.47661	99.16642	99.39319
3014	95.37539	99.47579	99.16637	99.39464
3013	95.0594	99.47499	99.16632	99.39608
3012	94.73099	99.4742	99.16627	99.39753
3011	94.39042	99.47344	99.16623	99.39897
3010	94.03803	99.47269	99.1662	99.40042
3009	93.67412	99.47197	99.16616	99.40185
3008	93.29901	99.47126	99.16614	99.40329
3007	92.91302	99.47057	99.16611	99.40472
3006	92.51647	99.4699	99.1661	99.40615
3005	92.10971	99.46925	99.16608	99.40757
3004	91.69314	99.46863	99.16608	99.40899
3003	91.26715	99.46802	99.16607	99.4104
3002	90.83222	99.46743	99.16608	99.41181
3001	90.38883	99.46686	99.16609	99.41321
3000	89.93753	99.46632	99.1661	99.4146
2999	89.47892	99.46579	99.16612	99.41598
2998	89.01366	99.46529	99.16615	99.41736
2997	88.54249	99.46481	99.16618	99.41873
2996	88.06618	99.46435	99.16622	99.42009
2995	87.58555	99.46391	99.16626	99.42144
2994	87.10143	99.46349	99.16631	99.42277
2993	86.61463	99.46309	99.16636	99.4241
2992	86.12595	99.46272	99.16643	99.42542
2991	85.63618	99.46236	99.16649	99.42673
2990	85.1461	99.46203	99.16657	99.42802
2989	84.6565	99.46173	99.16665	99.42931
2988	84.16815	99.46144	99.16674	99.43058
2987	83.68182	99.46118	99.16683	99.43184
2986	83.19825	99.46094	99.16693	99.43308
2985	82.71809	99.46072	99.16704	99.43431
2984	82.24196	99.46052	99.16715	99.43553
2983	81.7704	99.46035	99.16727	99.43673
2982	81.30392	99.4602	99.1674	99.43792
2981	80.84299	99.46007	99.16754	99.43909
2980	80.38804	99.45996	99.16768	99.44024

2979	79.93951	99.45988	99.16783	99.44138
2978	79.49784	99.45982	99.16799	99.44251
2977	79.06346	99.45979	99.16815	99.44361
2976	78.63676	99.45977	99.16832	99.4447
2975	78.21814	99.45978	99.1685	99.44577
2974	77.80794	99.45982	99.16869	99.44683
2973	77.40647	99.45987	99.16888	99.44786
2972	77.01397	99.45995	99.16908	99.44888
2971	76.63063	99.46006	99.16929	99.44988
2970	76.25656	99.46018	99.1695	99.45086
2969	75.89182	99.46033	99.16973	99.45181
2968	75.53645	99.46051	99.16996	99.45275
2967	75.1905	99.4607	99.17019	99.45367
2966	74.85408	99.46092	99.17044	99.45457
2965	74.52736	99.46116	99.17069	99.45545
2964	74.21059	99.46143	99.17095	99.45631
2963	73.90411	99.46171	99.17122	99.45715
2962	73.60826	99.46203	99.1715	99.45796
2961	73.32341	99.46236	99.17178	99.45876
2960	73.04988	99.46272	99.17207	99.45953
2959	72.78793	99.4631	99.17237	99.46028
2958	72.53776	99.4635	99.17268	99.461
2957	72.29945	99.46393	99.17299	99.46171
2956	72.07305	99.46438	99.17331	99.46239
2955	71.85857	99.46485	99.17364	99.46305
2954	71.65597	99.46534	99.17398	99.46369
2953	71.4652	99.46586	99.17432	99.4643
2952	71.28623	99.4664	99.17467	99.46489
2951	71.11902	99.46696	99.17503	99.46546
2950	70.96349	99.46754	99.1754	99.46601
2949	70.81956	99.46815	99.17577	99.46653
2948	70.68708	99.46878	99.17616	99.46702
2947	70.56584	99.46943	99.17655	99.46749
2946	70.45555	99.4701	99.17694	99.46794
2945	70.35587	99.4708	99.17735	99.46837
2944	70.26638	99.47151	99.17776	99.46877
2943	70.18663	99.47225	99.17818	99.46914
2942	70.11615	99.47301	99.1786	99.4695
2941	70.05445	99.47379	99.17903	99.46982
2940	70.00106	99.47459	99.17947	99.47013
2939	69.95551	99.47541	99.17992	99.47041

2938	69.91733	99.47626	99.18037	99.47066
2937	69.88608	99.47712	99.18084	99.47089
2936	69.86131	99.478	99.1813	99.4711
2935	69.8426	99.47891	99.18178	99.47128
2934	69.82955	99.47984	99.18226	99.47144
2933	69.8218	99.48078	99.18275	99.47157
2932	69.81906	99.48175	99.18324	99.47168
2931	69.82107	99.48273	99.18374	99.47176
2930	69.82766	99.48374	99.18425	99.47182
2929	69.83874	99.48476	99.18477	99.47186
2928	69.85428	99.48581	99.18529	99.47187
2927	69.87433	99.48687	99.18581	99.47186
2926	69.89897	99.48795	99.18635	99.47183
2925	69.92831	99.48905	99.18688	99.47177
2924	69.96246	99.49017	99.18743	99.47169
2923	70.00144	99.49131	99.18798	99.47159
2922	70.04524	99.49246	99.18854	99.47146
2921	70.09376	99.49363	99.1891	99.47131
2920	70.14679	99.49483	99.18967	99.47113
2919	70.20407	99.49603	99.19024	99.47094
2918	70.26526	99.49726	99.19082	99.47072
2917	70.32998	99.4985	99.1914	99.47048
2916	70.39782	99.49976	99.19199	99.47021
2915	70.46836	99.50104	99.19258	99.46993
2914	70.54117	99.50233	99.19318	99.46962
2913	70.61588	99.50364	99.19379	99.4693
2912	70.6921	99.50496	99.1944	99.46895
2911	70.76949	99.5063	99.19501	99.46858
2910	70.84773	99.50765	99.19563	99.46818
2909	70.92649	99.50902	99.19625	99.46777
2908	71.00545	99.51041	99.19687	99.46734
2907	71.0843	99.51181	99.1975	99.46689
2906	71.16275	99.51322	99.19814	99.46642
2905	71.24054	99.51465	99.19878	99.46593
2904	71.31749	99.51609	99.19942	99.46542
2903	71.39352	99.51754	99.20007	99.46489
2902	71.46864	99.51901	99.20071	99.46434
2901	71.54297	99.52049	99.20137	99.46378
2900	71.61671	99.52199	99.20202	99.4632
2899	71.69012	99.52349	99.20268	99.4626
2898	71.76349	99.52501	99.20334	99.46198

2897	71.83715	99.52654	99.20401	99.46135
2896	71.91148	99.52808	99.20468	99.4607
2895	71.98687	99.52963	99.20535	99.46003
2894	72.06379	99.5312	99.20602	99.45935
2893	72.1427	99.53277	99.20669	99.45865
2892	72.22412	99.53436	99.20737	99.45794
2891	72.30855	99.53595	99.20805	99.45721
2890	72.39646	99.53756	99.20873	99.45647
2889	72.48828	99.53917	99.20941	99.45571
2888	72.58436	99.54079	99.2101	99.45495
2887	72.68499	99.54243	99.21078	99.45417
2886	72.79038	99.54407	99.21147	99.45337
2885	72.90068	99.54572	99.21216	99.45257
2884	73.01601	99.54737	99.21285	99.45175
2883	73.13641	99.54904	99.21354	99.45092
2882	73.26194	99.55071	99.21423	99.45008
2881	73.39262	99.55239	99.21492	99.44923
2880	73.52845	99.55408	99.21561	99.44837
2879	73.66949	99.55577	99.2163	99.4475
2878	73.81576	99.55747	99.21699	99.44662
2877	73.96731	99.55917	99.21769	99.44573
2876	74.12421	99.56088	99.21838	99.44483
2875	74.28652	99.5626	99.21907	99.44393
2874	74.4543	99.56432	99.21976	99.44301
2873	74.62763	99.56604	99.22045	99.4421
2872	74.80661	99.56777	99.22114	99.44117
2871	74.99137	99.5695	99.22183	99.44024
2870	75.18204	99.57124	99.22252	99.4393
2869	75.37882	99.57298	99.2232	99.43836
2868	75.58191	99.57472	99.22389	99.43741
2867	75.79154	99.57647	99.22457	99.43646
2866	76.00794	99.57821	99.22525	99.4355
2865	76.23133	99.57996	99.22593	99.43454
2864	76.46193	99.58171	99.22661	99.43358
2863	76.69993	99.58347	99.22729	99.43261
2862	76.94551	99.58522	99.22796	99.43165
2861	77.19876	99.58697	99.22863	99.43068
2860	77.45971	99.58873	99.2293	99.42971
2859	77.72832	99.59048	99.22997	99.42874
2858	78.00444	99.59224	99.23063	99.42777
2857	78.28786	99.59399	99.2313	99.42679

2856	78.5783	99.59574	99.23195	99.42582
2855	78.87548	99.59749	99.23261	99.42485
2854	79.17905	99.59924	99.23326	99.42389
2853	79.48868	99.60099	99.23391	99.42292
2852	79.80398	99.60274	99.23455	99.42196
2851	80.12454	99.60448	99.23519	99.421
2850	80.44987	99.60622	99.23583	99.42004
2849	80.77944	99.60796	99.23646	99.41908
2848	81.11268	99.60969	99.23709	99.41813
2847	81.449	99.61142	99.23771	99.41719
2846	81.78781	99.61315	99.23833	99.41625
2845	82.12856	99.61487	99.23895	99.41531
2844	82.47072	99.61659	99.23956	99.41438
2843	82.8138	99.6183	99.24017	99.41346
2842	83.15734	99.62	99.24077	99.41254
2841	83.50088	99.6217	99.24137	99.41163
2840	83.84402	99.6234	99.24196	99.41073
2839	84.18633	99.62509	99.24254	99.40983
2838	84.52745	99.62677	99.24312	99.40894
2837	84.86702	99.62844	99.2437	99.40807
2836	85.20471	99.63011	99.24427	99.4072
2835	85.54021	99.63177	99.24483	99.40633
2834	85.87324	99.63343	99.24539	99.40548
2833	86.20351	99.63507	99.24594	99.40464
2832	86.53078	99.63671	99.24649	99.40381
2831	86.85478	99.63833	99.24703	99.40299
2830	87.17531	99.63995	99.24757	99.40218
2829	87.49215	99.64156	99.2481	99.40138
2828	87.80513	99.64316	99.24862	99.4006
2827	88.11404	99.64475	99.24913	99.39982
2826	88.4187	99.64633	99.24964	99.39906
2825	88.71888	99.6479	99.25015	99.39831
2824	89.01436	99.64946	99.25064	99.39757
2823	89.30486	99.65101	99.25113	99.39685
2822	89.59011	99.65255	99.25161	99.39614
2821	89.86981	99.65408	99.25209	99.39544
2820	90.14368	99.65559	99.25256	99.39476
2819	90.4114	99.65709	99.25302	99.39409
2818	90.67273	99.65858	99.25347	99.39343
2817	90.92742	99.66006	99.25392	99.3928
2816	91.17531	99.66153	99.25436	99.39217

2815	91.41631	99.66298	99.25479	99.39156
2814	91.65039	99.66442	99.25522	99.39097
2813	91.8776	99.66585	99.25564	99.39039
2812	92.09798	99.66726	99.25605	99.38983
2811	92.31162	99.66866	99.25645	99.38928
2810	92.51859	99.67004	99.25685	99.38875
2809	92.71892	99.67141	99.25724	99.38824
2808	92.91266	99.67277	99.25762	99.38774
2807	93.09986	99.67411	99.25799	99.38726
2806	93.2806	99.67544	99.25836	99.3868
2805	93.45502	99.67675	99.25871	99.38635
2804	93.62328	99.67804	99.25906	99.38592
2803	93.78557	99.67932	99.2594	99.38551
2802	93.94214	99.68059	99.25974	99.38512
2801	94.0932	99.68183	99.26006	99.38474
2800	94.23899	99.68306	99.26038	99.38438
2799	94.37972	99.68428	99.26069	99.38404
2798	94.51559	99.68548	99.261	99.38372
2797	94.64677	99.68666	99.26129	99.38342
2796	94.77341	99.68783	99.26158	99.38313
2795	94.89562	99.68897	99.26186	99.38286
2794	95.01349	99.6901	99.26213	99.38261
2793	95.12707	99.69122	99.26239	99.38238
2792	95.23644	99.69231	99.26265	99.38216
2791	95.34168	99.69339	99.26289	99.38197
2790	95.4429	99.69445	99.26313	99.38179
2789	95.54026	99.69549	99.26337	99.38163
2788	95.63389	99.69651	99.26359	99.38149
2787	95.72398	99.69752	99.2638	99.38137
2786	95.81066	99.6985	99.26401	99.38127
2785	95.89405	99.69947	99.26421	99.38118
2784	95.97427	99.70042	99.26441	99.38111
2783	96.05139	99.70135	99.26459	99.38106
2782	96.12552	99.70226	99.26477	99.38103
2781	96.19676	99.70315	99.26494	99.38102
2780	96.26524	99.70402	99.2651	99.38103
2779	96.33108	99.70487	99.26525	99.38105
2778	96.39443	99.70571	99.2654	99.38109
2777	96.45547	99.70652	99.26554	99.38115
2776	96.51436	99.70731	99.26567	99.38123
2775	96.57131	99.70809	99.2658	99.38132

2774	96.62655	99.70884	99.26591	99.38143
2773	96.68031	99.70957	99.26602	99.38156
2772	96.7328	99.71029	99.26612	99.38171
2771	96.7842	99.71098	99.26622	99.38187
2770	96.83464	99.71166	99.26631	99.38205
2769	96.88416	99.71231	99.26639	99.38225
2768	96.93276	99.71294	99.26646	99.38247
2767	96.98041	99.71355	99.26653	99.3827
2766	97.02705	99.71414	99.26659	99.38294
2765	97.07265	99.71471	99.26665	99.38321
2764	97.11721	99.71527	99.26669	99.38349
2763	97.16075	99.7158	99.26673	99.38378
2762	97.2033	99.7163	99.26677	99.38409
2761	97.2449	99.71679	99.2668	99.38442
2760	97.28558	99.71726	99.26682	99.38476
2759	97.32536	99.71771	99.26683	99.38511
2758	97.36423	99.71813	99.26684	99.38548
2757	97.40219	99.71854	99.26684	99.38587
2756	97.43923	99.71892	99.26684	99.38627
2755	97.47535	99.71929	99.26683	99.38668
2754	97.51057	99.71963	99.26682	99.38711
2753	97.54495	99.71995	99.2668	99.38755
2752	97.57855	99.72026	99.26677	99.388
2751	97.6115	99.72054	99.26674	99.38847
2750	97.64392	99.7208	99.26671	99.38894
2749	97.67593	99.72104	99.26667	99.38944
2748	97.70764	99.72126	99.26662	99.38994
2747	97.73911	99.72146	99.26657	99.39045
2746	97.77036	99.72163	99.26651	99.39098
2745	97.80139	99.72179	99.26645	99.39152
2744	97.83217	99.72193	99.26639	99.39206
2743	97.86268	99.72204	99.26632	99.39262
2742	97.89293	99.72214	99.26624	99.39319
2741	97.92296	99.72222	99.26617	99.39377
2740	97.95281	99.72227	99.26608	99.39436
2739	97.98253	99.72231	99.266	99.39496
2738	98.01218	99.72232	99.26591	99.39556
2737	98.04176	99.72232	99.26581	99.39618
2736	98.07132	99.72229	99.26572	99.3968
2735	98.10084	99.72225	99.26562	99.39743
2734	98.13035	99.72219	99.26551	99.39807

2733	98.15986	99.7221	99.26541	99.39872
2732	98.18937	99.722	99.2653	99.39937
2731	98.21887	99.72188	99.26518	99.40003
2730	98.2483	99.72174	99.26507	99.4007
2729	98.27756	99.72157	99.26495	99.40137
2728	98.30654	99.72139	99.26483	99.40205
2727	98.33512	99.7212	99.26471	99.40273
2726	98.36321	99.72098	99.26458	99.40342
2725	98.39079	99.72074	99.26446	99.40411
2724	98.41789	99.72049	99.26433	99.40481
2723	98.44458	99.72021	99.2642	99.40551
2722	98.47099	99.71992	99.26407	99.40621
2721	98.49721	99.71961	99.26394	99.40692
2720	98.52334	99.71928	99.2638	99.40762
2719	98.54943	99.71894	99.26367	99.40834
2718	98.57551	99.71857	99.26353	99.40905
2717	98.60158	99.71819	99.2634	99.40976
2716	98.62764	99.7178	99.26326	99.41048
2715	98.65368	99.71738	99.26312	99.4112
2714	98.67973	99.71695	99.26299	99.41191
2713	98.70584	99.7165	99.26285	99.41263
2712	98.73209	99.71604	99.26271	99.41335
2711	98.75858	99.71555	99.26258	99.41406
2710	98.7854	99.71506	99.26244	99.41478
2709	98.81262	99.71454	99.2623	99.41549
2708	98.84028	99.71401	99.26217	99.41621
2707	98.86836	99.71347	99.26203	99.41692
2706	98.89677	99.71291	99.2619	99.41763
2705	98.92541	99.71233	99.26177	99.41833
2704	98.95416	99.71174	99.26164	99.41903
2703	98.9829	99.71114	99.26151	99.41973
2702	99.01155	99.71052	99.26138	99.42043
2701	99.04005	99.70988	99.26125	99.42112
2700	99.06837	99.70924	99.26113	99.42181
2699	99.09643	99.70858	99.26101	99.42249
2698	99.12417	99.7079	99.26089	99.42317
2697	99.15148	99.70721	99.26077	99.42384
2696	99.17827	99.70651	99.26065	99.42451
2695	99.2044	99.7058	99.26054	99.42517
2694	99.22978	99.70507	99.26043	99.42583
2693	99.25432	99.70433	99.26033	99.42648

2692	99.27795	99.70358	99.26022	99.42712
2691	99.30061	99.70281	99.26012	99.42775
2690	99.32224	99.70204	99.26002	99.42838
2689	99.34278	99.70125	99.25993	99.429
2688	99.36218	99.70046	99.25984	99.42961
2687	99.38043	99.69965	99.25976	99.43021
2686	99.39757	99.69883	99.25968	99.43081
2685	99.41366	99.698	99.2596	99.4314
2684	99.42882	99.69716	99.25952	99.43197
2683	99.44318	99.69631	99.25946	99.43254
2682	99.45685	99.69546	99.25939	99.4331
2681	99.46992	99.69459	99.25933	99.43365
2680	99.48247	99.69371	99.25928	99.43419
2679	99.49454	99.69283	99.25923	99.43471
2678	99.50617	99.69194	99.25918	99.43523
2677	99.5174	99.69104	99.25914	99.43574
2676	99.5283	99.69013	99.25911	99.43624
2675	99.5389	99.68921	99.25908	99.43672
2674	99.54926	99.68829	99.25906	99.43719
2673	99.55943	99.68736	99.25904	99.43766
2672	99.56939	99.68642	99.25903	99.43811
2671	99.57913	99.68548	99.25902	99.43855
2670	99.58858	99.68453	99.25902	99.43897
2669	99.59764	99.68358	99.25903	99.43939
2668	99.60619	99.68262	99.25904	99.43979
2667	99.61406	99.68166	99.25906	99.44018
2666	99.61886	99.68069	99.25909	99.44056
2665	99.62497	99.67971	99.25912	99.44092
2664	99.63014	99.67874	99.25916	99.44128
2663	99.6343	99.67776	99.2592	99.44161
2662	99.63738	99.67677	99.25925	99.44194
2661	99.63941	99.67579	99.25931	99.44225
2660	99.64043	99.6748	99.25938	99.44255
2659	99.64049	99.6738	99.25945	99.44284
2658	99.63966	99.67281	99.25953	99.44311
2657	99.63802	99.67181	99.25962	99.44337
2656	99.63564	99.67081	99.25972	99.44361
2655	99.63261	99.66981	99.25982	99.44384
2654	99.62904	99.66881	99.25993	99.44406
2653	99.62508	99.66781	99.26005	99.44426
2652	99.62088	99.66681	99.26017	99.44445

2651	99.61658	99.66581	99.26031	99.44463
2650	99.61233	99.66481	99.26045	99.44479
2649	99.60825	99.66381	99.2606	99.44493
2648	99.60446	99.66281	99.26075	99.44507
2647	99.60105	99.66182	99.26092	99.44519
2646	99.59814	99.66082	99.26109	99.44529
2645	99.59585	99.65983	99.26127	99.44538
2644	99.59426	99.65884	99.26146	99.44546
2643	99.59343	99.65785	99.26166	99.44552
2642	99.59337	99.65686	99.26187	99.44557
2641	99.59399	99.65588	99.26208	99.44561
2640	99.59519	99.6549	99.2623	99.44563
2639	99.59683	99.65392	99.26253	99.44564
2638	99.59875	99.65295	99.26277	99.44563
2637	99.60084	99.65199	99.26302	99.44561
2636	99.603	99.65103	99.26327	99.44558
2635	99.60514	99.65007	99.26354	99.44553
2634	99.60718	99.64912	99.26381	99.44547
2633	99.60905	99.64818	99.26409	99.4454
2632	99.61065	99.64724	99.26438	99.44531
2631	99.61185	99.6463	99.26468	99.44521
2630	99.61256	99.64538	99.26499	99.4451
2629	99.61268	99.64446	99.2653	99.44498
2628	99.61214	99.64355	99.26562	99.44484
2627	99.61089	99.64265	99.26596	99.44469
2626	99.60888	99.64175	99.2663	99.44452
2625	99.60162	99.64087	99.26665	99.44435
2624	99.59836	99.63999	99.267	99.44416
2623	99.59496	99.63912	99.26737	99.44396
2622	99.59148	99.63826	99.26774	99.44375
2621	99.58799	99.63741	99.26813	99.44353
2620	99.58461	99.63657	99.26852	99.44329
2619	99.58149	99.63574	99.26892	99.44305
2618	99.57878	99.63491	99.26933	99.44279
2617	99.57658	99.63411	99.26974	99.44252
2616	99.575	99.63331	99.27017	99.44225
2615	99.57407	99.63252	99.2706	99.44196
2614	99.57377	99.63174	99.27104	99.44166
2613	99.57407	99.63098	99.27149	99.44135
2612	99.57488	99.63023	99.27194	99.44103
2611	99.57611	99.62949	99.27241	99.4407

2610	99.57768	99.62876	99.27288	99.44037
2609	99.57951	99.62805	99.27336	99.44002
2608	99.58155	99.62735	99.27385	99.43966
2607	99.58379	99.62666	99.27435	99.4393
2606	99.58623	99.62598	99.27485	99.43893
2605	99.58888	99.62532	99.27536	99.43855
2604	99.59172	99.62468	99.27588	99.43816
2603	99.5947	99.62405	99.27641	99.43777
2602	99.59773	99.62343	99.27694	99.43736
2601	99.60068	99.62283	99.27749	99.43695
2600	99.6034	99.62225	99.27804	99.43654
2599	99.60578	99.62168	99.27859	99.43611
2598	99.60775	99.62112	99.27915	99.43569
2597	99.60929	99.62058	99.27972	99.43525
2596	99.61043	99.62006	99.2803	99.43481
2595	99.61122	99.61955	99.28088	99.43437
2594	99.61171	99.61907	99.28147	99.43392
2593	99.61195	99.61859	99.28207	99.43346
2592	99.61194	99.61814	99.28267	99.433
2591	99.61166	99.6177	99.28328	99.43254
2590	99.61109	99.61728	99.2839	99.43207
2589	99.61022	99.61688	99.28452	99.4316
2588	99.60905	99.61649	99.28515	99.43113
2587	99.60761	99.61613	99.28578	99.43065
2586	99.606	99.61578	99.28642	99.43017
2585	99.6043	99.61545	99.28707	99.42969
2584	99.60268	99.61514	99.28772	99.42921
2583	99.60129	99.61485	99.28838	99.42873
2582	99.60032	99.61458	99.28904	99.42824
2581	99.59995	99.61433	99.28971	99.42775
2580	99.60029	99.61409	99.29038	99.42727
2579	99.6014	99.61388	99.29105	99.42678
2578	99.60325	99.61369	99.29173	99.42629
2577	99.60574	99.61351	99.29242	99.42581
2576	99.60871	99.61336	99.29311	99.42532
2575	99.61863	99.61323	99.29381	99.42484
2574	99.62085	99.61311	99.2945	99.42435
2573	99.62276	99.61302	99.29521	99.42387
2572	99.62431	99.61295	99.29591	99.42339
2571	99.6255	99.6129	99.29662	99.42292
2570	99.62636	99.61287	99.29734	99.42244

2569	99.62697	99.61287	99.29806	99.42197
2568	99.62741	99.61288	99.29878	99.4215
2567	99.62778	99.61292	99.2995	99.42104
2566	99.62819	99.61297	99.30023	99.42058
2565	99.62869	99.61305	99.30096	99.42012
2564	99.62931	99.61315	99.30169	99.41967
2563	99.63001	99.61327	99.30242	99.41922
2562	99.63072	99.61342	99.30316	99.41878
2561	99.63134	99.61359	99.3039	99.41834
2560	99.63177	99.61377	99.30464	99.41791
2559	99.63195	99.61399	99.30538	99.41748
2558	99.63184	99.61422	99.30613	99.41707
2557	99.63143	99.61447	99.30687	99.41665
2556	99.63075	99.61475	99.30762	99.41625
2555	99.62979	99.61505	99.30837	99.41585
2554	99.62857	99.61538	99.30912	99.41546
2553	99.6271	99.61573	99.30987	99.41508
2552	99.62543	99.61609	99.31062	99.4147
2551	99.62363	99.61649	99.31137	99.41433
2550	99.62184	99.6169	99.31212	99.41398
2549	99.62023	99.61734	99.31287	99.41363
2548	99.61898	99.6178	99.31362	99.41329
2547	99.61826	99.61829	99.31437	99.41296
2546	99.61818	99.61879	99.31512	99.41263
2545	99.61884	99.61932	99.31587	99.41232
2544	99.6241	99.61988	99.31662	99.41202
2543	99.62567	99.62045	99.31737	99.41173
2542	99.62707	99.62105	99.31812	99.41145
2541	99.62826	99.62168	99.31886	99.41118
2540	99.6292	99.62232	99.31961	99.41092
2539	99.62982	99.62299	99.32035	99.41067
2538	99.63001	99.62368	99.32109	99.41043
2537	99.6297	99.6244	99.32183	99.41021
2536	99.62882	99.62513	99.32257	99.40999
2535	99.62738	99.62589	99.3233	99.40979
2534	99.62547	99.62668	99.32403	99.4096
2533	99.62325	99.62749	99.32476	99.40942
2532	99.62091	99.62831	99.32549	99.40926
2531	99.61868	99.62917	99.32621	99.4091
2530	99.61675	99.63004	99.32693	99.40896
2529	99.61526	99.63094	99.32765	99.40884

2528	99.61432	99.63186	99.32836	99.40872
2527	99.61399	99.6328	99.32907	99.40862
2526	99.61427	99.63377	99.32978	99.40853
2525	99.61513	99.63476	99.33048	99.40846
2524	99.61648	99.63577	99.33118	99.4084
2523	99.61817	99.6368	99.33187	99.40835
2522	99.62003	99.63786	99.33256	99.40832
2521	99.62187	99.63893	99.33324	99.4083
2520	99.62352	99.64003	99.33392	99.40829
2519	99.61993	99.64115	99.33459	99.4083
2518	99.62093	99.6423	99.33526	99.40833
2517	99.62117	99.64346	99.33593	99.40836
2516	99.62077	99.64465	99.33658	99.40841
2515	99.61989	99.64585	99.33724	99.40848
2514	99.61867	99.64708	99.33788	99.40856
2513	99.61724	99.64833	99.33853	99.40865
2512	99.61572	99.6496	99.33916	99.40876
2511	99.61424	99.65089	99.33979	99.40888
2510	99.61292	99.65221	99.34041	99.40902
2509	99.61188	99.65354	99.34103	99.40917
2508	99.61119	99.65489	99.34164	99.40934
2507	99.61086	99.65627	99.34224	99.40952
2506	99.61081	99.65766	99.34284	99.40972
2505	99.61092	99.65907	99.34342	99.40993
2504	99.61102	99.66051	99.34401	99.41015
2503	99.61099	99.66196	99.34458	99.41039
2502	99.61077	99.66343	99.34515	99.41064
2501	99.61039	99.66492	99.34571	99.41091
2500	99.60991	99.66643	99.34626	99.41119
2499	99.60945	99.66796	99.3468	99.41149
2498	99.60911	99.66951	99.34734	99.4118
2497	99.60896	99.67108	99.34787	99.41212
2496	99.60906	99.67266	99.34839	99.41246
2495	99.60945	99.67426	99.3489	99.41281
2494	99.61016	99.67589	99.3494	99.41318
2493	99.61123	99.67752	99.3499	99.41356
2492	99.61264	99.67918	99.35039	99.41395
2491	99.61435	99.68085	99.35087	99.41435
2490	99.61624	99.68254	99.35134	99.41477
2489	99.61812	99.68425	99.3518	99.41521
2488	99.61978	99.68597	99.35225	99.41565

2487	99.62099	99.68771	99.35269	99.41611
2486	99.62156	99.68946	99.35313	99.41658
2485	99.62135	99.69123	99.35355	99.41707
2484	99.62029	99.69302	99.35397	99.41756
2483	99.62031	99.69482	99.35438	99.41807
2482	99.61809	99.69663	99.35477	99.41859
2481	99.61663	99.69846	99.35516	99.41912
2480	99.61595	99.70031	99.35554	99.41967
2479	99.61615	99.70216	99.35591	99.42022
2478	99.61781	99.70404	99.35627	99.42079
2477	99.61982	99.70592	99.35662	99.42137
2476	99.62209	99.70782	99.35696	99.42196
2475	99.62481	99.70973	99.35729	99.42256
2474	99.6281	99.71166	99.35762	99.42317
2473	99.63198	99.71359	99.35793	99.42379
2472	99.63641	99.71554	99.35823	99.42442
2471	99.6413	99.7175	99.35852	99.42506
2470	99.64656	99.71948	99.3588	99.42571
2469	99.65207	99.72146	99.35908	99.42637
2468	99.65773	99.72345	99.35934	99.42703
2467	99.6634	99.72546	99.35959	99.42771
2466	99.6689	99.72747	99.35984	99.42839
2465	99.67405	99.7295	99.36007	99.42909
2464	99.67868	99.73153	99.36029	99.42979
2463	99.68263	99.73358	99.36051	99.4305
2462	99.68586	99.73563	99.36071	99.43121
2461	99.68837	99.73769	99.3609	99.43193
2460	99.69024	99.73976	99.36109	99.43266
2459	99.69157	99.74184	99.36126	99.4334
2458	99.69245	99.74393	99.36142	99.43414
2457	99.69293	99.74602	99.36158	99.43489
2456	99.69301	99.74812	99.36172	99.43564
2455	99.69266	99.75023	99.36186	99.4364
2454	99.69184	99.75234	99.36198	99.43716
2453	99.69051	99.75446	99.36209	99.43793
2452	99.68862	99.75659	99.3622	99.4387
2451	99.68612	99.75872	99.3623	99.43947
2450	99.68296	99.76085	99.36238	99.44025
2449	99.67912	99.76299	99.36246	99.44104
2448	99.67458	99.76514	99.36252	99.44182
2447	99.66942	99.76729	99.36258	99.44261

2446	99.66375	99.76944	99.36263	99.4434
2445	99.65775	99.77159	99.36267	99.44419
2444	99.65164	99.77375	99.3627	99.44498
2443	99.64558	99.77591	99.36272	99.44578
2442	99.63969	99.77808	99.36273	99.44657
2441	99.63403	99.78024	99.36273	99.44737
2440	99.62857	99.78241	99.36272	99.44817
2439	99.62327	99.78457	99.36271	99.44896
2438	99.61808	99.78674	99.36268	99.44976
2437	99.61101	99.78891	99.36265	99.45055
2436	99.60615	99.79108	99.3626	99.45134
2435	99.60149	99.79325	99.36255	99.45214
2434	99.59699	99.79542	99.36249	99.45293
2433	99.59259	99.79759	99.36243	99.45371
2432	99.58819	99.79975	99.36235	99.4545
2431	99.58369	99.80192	99.36227	99.45528
2430	99.57901	99.80408	99.36218	99.45606
2429	99.5741	99.80624	99.36208	99.45683
2428	99.56894	99.80839	99.36197	99.45761
2427	99.56358	99.81055	99.36185	99.45837
2426	99.55807	99.8127	99.36173	99.45913
2425	99.55247	99.81485	99.3616	99.45989
2424	99.54692	99.81699	99.36146	99.46064
2423	99.54154	99.81913	99.36132	99.46139
2422	99.53655	99.82126	99.36117	99.46213
2421	99.53217	99.82339	99.36101	99.46286
2420	99.52863	99.82551	99.36084	99.46359
2419	99.52615	99.82762	99.36067	99.46431
2418	99.52485	99.82973	99.36049	99.46502
2417	99.52479	99.83184	99.36031	99.46573
2416	99.52592	99.83393	99.36012	99.46642
2415	99.52813	99.83602	99.35992	99.46711
2414	99.53126	99.8381	99.35972	99.46779
2413	99.53514	99.84017	99.35951	99.46846
2412	99.53961	99.84224	99.3593	99.46913
2411	99.54451	99.84429	99.35908	99.46978
2410	99.54969	99.84634	99.35885	99.47042
2409	99.55503	99.84838	99.35862	99.47105
2408	99.56038	99.8504	99.35839	99.47168
2407	99.56568	99.85242	99.35815	99.47229
2406	99.57093	99.85443	99.35791	99.47289

2405	99.57619	99.85642	99.35766	99.47348
2404	99.58157	99.85841	99.35741	99.47405
2403	99.58712	99.86038	99.35715	99.47462
2402	99.59276	99.86234	99.3569	99.47517
2401	99.59828	99.86429	99.35663	99.47572
2400	99.60338	99.86623	99.35637	99.47624
2399	99.60775	99.86815	99.3561	99.47676
2398	99.61117	99.87006	99.35583	99.47726
2397	99.61352	99.87196	99.35555	99.47775
2396	99.61486	99.87384	99.35527	99.47823
2395	99.61535	99.87571	99.35499	99.47869
2394	99.61522	99.87757	99.35471	99.47914
2393	99.61476	99.87941	99.35443	99.47958
2392	99.61428	99.88123	99.35414	99.48
2391	99.61258	99.88304	99.35385	99.4804
2390	99.61277	99.88484	99.35357	99.48079
2389	99.61359	99.88662	99.35328	99.48117
2388	99.61505	99.88838	99.35298	99.48153
2387	99.61708	99.89012	99.35269	99.48188
2386	99.61956	99.89185	99.3524	99.48221
2385	99.62236	99.89356	99.35211	99.48252
2384	99.62537	99.89526	99.35181	99.48282
2383	99.62854	99.89694	99.35152	99.4831
2382	99.63187	99.89859	99.35123	99.48337
2381	99.63536	99.90024	99.35094	99.48362
2380	99.63896	99.90186	99.35064	99.48385
2379	99.64262	99.90346	99.35035	99.48407
2378	99.6462	99.90505	99.35006	99.48427
2377	99.6495	99.90661	99.34978	99.48446
2376	99.65231	99.90816	99.34949	99.48463
2375	99.65442	99.90968	99.3492	99.48478
2374	99.65565	99.91119	99.34892	99.48491
2373	99.65588	99.91267	99.34864	99.48503
2372	99.65506	99.91414	99.34836	99.48513
2371	99.65321	99.91558	99.34808	99.48522
2370	99.65041	99.91701	99.34781	99.48529
2369	99.64673	99.91841	99.34754	99.48534
2368	99.64223	99.91979	99.34727	99.48537
2367	99.63698	99.92115	99.34701	99.48538
2366	99.63108	99.92249	99.34675	99.48538
2365	99.62467	99.9238	99.34649	99.48536

2364	99.61799	99.9251	99.34624	99.48533
2363	99.61126	99.92637	99.34599	99.48528
2362	99.60463	99.92761	99.34574	99.48521
2361	99.59814	99.92884	99.34551	99.48512
2360	99.59178	99.93004	99.34527	99.48502
2359	99.58556	99.93122	99.34504	99.4849
2358	99.57962	99.93238	99.34482	99.48476
2357	99.57428	99.93351	99.3446	99.48461
2356	99.57	99.93462	99.34438	99.48443
2355	99.56723	99.9357	99.34417	99.48425
2354	99.56636	99.93676	99.34397	99.48404
2353	99.56759	99.9378	99.34378	99.48382
2352	99.57094	99.93881	99.34359	99.48359
2351	99.57618	99.93979	99.3434	99.48333
2350	99.58291	99.94076	99.34323	99.48306
2349	99.59058	99.94169	99.34306	99.48278
2348	99.59854	99.94261	99.3429	99.48248
2347	99.60612	99.94349	99.34274	99.48216
2346	99.61883	99.94435	99.34259	99.48183
2345	99.6222	99.94519	99.34245	99.48148
2344	99.62208	99.946	99.34232	99.48112
2343	99.62018	99.94679	99.3422	99.48074
2342	99.61835	99.94755	99.34208	99.48035
2341	99.61846	99.94828	99.34197	99.47994
2340	99.61322	99.94899	99.34187	99.47952
2339	99.61484	99.94967	99.34178	99.47908
2338	99.61603	99.95033	99.3417	99.47863
2337	99.61676	99.95096	99.34163	99.47817
2336	99.61701	99.95157	99.34156	99.47769
2335	99.61679	99.95214	99.34151	99.4772
2334	99.61618	99.9527	99.34146	99.4767
2333	99.61529	99.95322	99.34143	99.47618
2332	99.61425	99.95372	99.3414	99.47565
2331	99.6132	99.9542	99.34139	99.47511
2330	99.61224	99.95464	99.34138	99.47456
2329	99.61146	99.95506	99.34138	99.474
2328	99.61093	99.95546	99.3414	99.47342
2327	99.61069	99.95582	99.34142	99.47283
2326	99.61083	99.95616	99.34146	99.47224
2325	99.61146	99.95648	99.3415	99.47163
2324	99.61273	99.95677	99.34156	99.47101

2323	99.61482	99.95703	99.34162	99.47038
2322	99.61784	99.95726	99.3417	99.46974
2321	99.62178	99.95747	99.34179	99.4691
2320	99.62654	99.95765	99.34189	99.46844
2319	99.63188	99.95781	99.342	99.46778
2318	99.63754	99.95794	99.34212	99.4671
2317	99.64322	99.95804	99.34226	99.46642
2316	99.64867	99.95811	99.3424	99.46574
2315	99.65362	99.95816	99.34256	99.46504
2314	99.65778	99.95819	99.34273	99.46434
2313	99.66088	99.95818	99.34291	99.46363
2312	99.66267	99.95816	99.3431	99.46292
2311	99.66294	99.9581	99.3433	99.4622
2310	99.6616	99.95802	99.34352	99.46148
2309	99.65869	99.95791	99.34374	99.46075
2308	99.6543	99.95778	99.34398	99.46001
2307	99.64859	99.95762	99.34423	99.45928
2306	99.64179	99.95744	99.3445	99.45854
2305	99.63417	99.95723	99.34477	99.45779
2304	99.62605	99.957	99.34506	99.45705
2303	99.61776	99.95674	99.34536	99.4563
2302	99.60965	99.95645	99.34567	99.45554
2301	99.60196	99.95614	99.346	99.45479
2300	99.61145	99.95581	99.34634	99.45404
2299	99.60575	99.95545	99.34668	99.45328
2298	99.6008	99.95506	99.34705	99.45253
2297	99.59635	99.95466	99.34742	99.45177
2296	99.59224	99.95422	99.34781	99.45102
2295	99.58836	99.95377	99.3482	99.45027
2294	99.58475	99.95328	99.34861	99.44952
2293	99.58149	99.95278	99.34904	99.44877
2292	99.57867	99.95225	99.34947	99.44802
2291	99.57636	99.9517	99.34992	99.44727
2290	99.57455	99.95112	99.35038	99.44653
2289	99.57318	99.95053	99.35085	99.44579
2288	99.57217	99.94991	99.35133	99.44506
2287	99.5714	99.94926	99.35183	99.44433
2286	99.57084	99.9486	99.35234	99.44361
2285	99.57045	99.94791	99.35286	99.44289
2284	99.57024	99.9472	99.35339	99.44217
2283	99.5702	99.94646	99.35394	99.44147

2282	99.57031	99.94571	99.35449	99.44076
2281	99.57047	99.94494	99.35506	99.44007
2280	99.57058	99.94414	99.35564	99.43938
2279	99.57054	99.94332	99.35623	99.43871
2278	99.57026	99.94248	99.35683	99.43804
2277	99.56973	99.94163	99.35745	99.43737
2276	99.56897	99.94075	99.35807	99.43672
2275	99.56805	99.93985	99.35871	99.43608
2274	99.56703	99.93893	99.35936	99.43545
2273	99.566	99.938	99.36002	99.43482
2272	99.56504	99.93704	99.36069	99.43421
2271	99.56427	99.93607	99.36137	99.43361
2270	99.56383	99.93507	99.36206	99.43302
2269	99.56385	99.93406	99.36276	99.43244
2268	99.56443	99.93303	99.36347	99.43188
2267	99.5656	99.93199	99.3642	99.43133
2266	99.5673	99.93092	99.36493	99.43079
2265	99.56942	99.92984	99.36568	99.43026
2264	99.57186	99.92874	99.36643	99.42975
2263	99.57452	99.92763	99.36719	99.42925
2262	99.57742	99.9265	99.36797	99.42877
2261	99.58059	99.92535	99.36875	99.4283
2260	99.58414	99.92419	99.36954	99.42785
2259	99.58813	99.92302	99.37034	99.42741
2258	99.5926	99.92183	99.37115	99.42699
2257	99.59753	99.92062	99.37197	99.42658
2256	99.60289	99.9194	99.3728	99.4262
2255	99.60863	99.91817	99.37364	99.42582
2254	99.60918	99.91692	99.37449	99.42547
2253	99.61582	99.91567	99.37534	99.42513
2252	99.62274	99.91439	99.3762	99.42481
2251	99.62982	99.91311	99.37707	99.42451
2250	99.63683	99.91181	99.37795	99.42423
2249	99.64346	99.91051	99.37883	99.42397
2248	99.64944	99.90919	99.37972	99.42372
2247	99.65461	99.90786	99.38062	99.4235
2246	99.65894	99.90652	99.38153	99.42329
2245	99.6626	99.90517	99.38244	99.4231
2244	99.66583	99.90381	99.38336	99.42294
2243	99.66891	99.90244	99.38428	99.42279
2242	99.67203	99.90107	99.38522	99.42267

2241	99.67527	99.89968	99.38615	99.42256
2240	99.67861	99.89829	99.38709	99.42248
2239	99.68195	99.89689	99.38804	99.42241
2238	99.68516	99.89548	99.38899	99.42237
2237	99.68809	99.89406	99.38995	99.42235
2236	99.69059	99.89264	99.39091	99.42235
2235	99.6925	99.89121	99.39188	99.42237
2234	99.69364	99.88978	99.39285	99.42242
2233	99.69385	99.88834	99.39383	99.42248
2232	99.69302	99.8869	99.3948	99.42257
2231	99.69119	99.88545	99.39579	99.42268
2230	99.6885	99.884	99.39677	99.42281
2229	99.68517	99.88254	99.39776	99.42297
2228	99.68145	99.88108	99.39875	99.42315
2227	99.67748	99.87962	99.39974	99.42335
2226	99.6733	99.87816	99.40074	99.42357
2225	99.66885	99.87669	99.40173	99.42382
2224	99.66403	99.87522	99.40273	99.42409
2223	99.65877	99.87375	99.40373	99.42438
2222	99.65313	99.87229	99.40473	99.42469
2221	99.64726	99.87082	99.40573	99.42503
2220	99.64138	99.86935	99.40674	99.42539
2219	99.63562	99.86788	99.40774	99.42577
2218	99.63006	99.86641	99.40874	99.42618
2217	99.62463	99.86495	99.40974	99.42661
2216	99.61918	99.86348	99.41075	99.42706
2215	99.6136	99.86202	99.41175	99.42753
2214	99.60784	99.86057	99.41275	99.42803
2213	99.59372	99.85911	99.41375	99.42855
2212	99.58803	99.85766	99.41474	99.42909
2211	99.5834	99.85621	99.41574	99.42965
2210	99.57976	99.85477	99.41673	99.43024
2209	99.57702	99.85333	99.41772	99.43085
2208	99.57515	99.8519	99.41871	99.43148
2207	99.57419	99.85047	99.4197	99.43213
2206	99.5743	99.84905	99.42068	99.43281
2205	99.57571	99.84764	99.42166	99.4335
2204	99.5786	99.84624	99.42263	99.43422
2203	99.58303	99.84484	99.4236	99.43496
2202	99.58878	99.84345	99.42457	99.43572
2201	99.59541	99.84207	99.42553	99.4365

2200	99.60229	99.84069	99.42649	99.4373
2199	99.60878	99.83933	99.42744	99.43813
2198	99.61434	99.83798	99.42838	99.43897
2197	99.61859	99.83663	99.42933	99.43983
2196	99.62135	99.8353	99.43026	99.44071
2195	99.62258	99.83398	99.43119	99.44161
2194	99.62232	99.83267	99.43211	99.44254
2193	99.62069	99.83137	99.43303	99.44348
2192	99.61783	99.83009	99.43393	99.44443
2191	99.61397	99.82881	99.43484	99.44541
2190	99.60936	99.82755	99.43573	99.44641
2189	99.60423	99.82631	99.43661	99.44742
2188	99.60985	99.82508	99.43749	99.44845
2187	99.60384	99.82386	99.43836	99.4495
2186	99.59945	99.82266	99.43922	99.45056
2185	99.5965	99.82147	99.44007	99.45164
2184	99.59475	99.8203	99.44092	99.45274
2183	99.59405	99.81914	99.44175	99.45385
2182	99.59431	99.818	99.44258	99.45498
2181	99.59554	99.81688	99.44339	99.45612
2180	99.59775	99.81577	99.4442	99.45727
2179	99.6008	99.81469	99.44499	99.45844
2178	99.60444	99.81362	99.44577	99.45963
2177	99.60827	99.81257	99.44655	99.46082
2176	99.61187	99.81153	99.44731	99.46203
2175	99.61494	99.81052	99.44806	99.46325
2174	99.61733	99.80953	99.4488	99.46449
2173	99.61911	99.80855	99.44953	99.46573
2172	99.62042	99.8076	99.45024	99.46699
2171	99.62137	99.80667	99.45095	99.46825
2170	99.62197	99.80576	99.45164	99.46953
2169	99.62207	99.80487	99.45232	99.47081
2168	99.62141	99.804	99.45299	99.47211
2167	99.62588	99.80315	99.45364	99.47341
2166	99.62504	99.80233	99.45428	99.47472
2165	99.62365	99.80153	99.45491	99.47604
2164	99.62187	99.80075	99.45552	99.47736
2163	99.61983	99.8	99.45613	99.47869
2162	99.61755	99.79927	99.45671	99.48003
2161	99.61492	99.79856	99.45729	99.48137
2160	99.61177	99.79788	99.45785	99.48272

2159	99.608	99.79723	99.45839	99.48407
2158	99.6037	99.7966	99.45892	99.48542
2157	99.59918	99.79599	99.45944	99.48678
2156	99.59488	99.79541	99.45994	99.48814
2155	99.5913	99.79486	99.46043	99.4895
2154	99.58883	99.79433	99.4609	99.49086
2153	99.58771	99.79383	99.46135	99.49222
2152	99.588	99.79336	99.4618	99.49359
2151	99.58964	99.79291	99.46222	99.49495
2150	99.59252	99.7925	99.46263	99.49631
2149	99.59644	99.7921	99.46303	99.49767
2148	99.60117	99.79174	99.46341	99.49903
2147	99.60642	99.79141	99.46377	99.50039
2146	99.6118	99.7911	99.46412	99.50174
2145	99.61691	99.79083	99.46445	99.50309
2144	99.62143	99.79058	99.46476	99.50443
2143	99.62517	99.79036	99.46506	99.50577
2142	99.62811	99.79017	99.46534	99.50711
2141	99.6304	99.79002	99.46561	99.50843
2140	99.63224	99.78989	99.46586	99.50975
2139	99.63386	99.78979	99.46609	99.51107
2138	99.63536	99.78972	99.46631	99.51237
2137	99.63682	99.78969	99.46651	99.51367
2136	99.63828	99.78968	99.4667	99.51495
2135	99.63985	99.78971	99.46686	99.51623
2134	99.64176	99.78977	99.46701	99.5175
2133	99.64427	99.78985	99.46715	99.51875
2132	99.62937	99.78997	99.46727	99.52
2131	99.6336	99.79013	99.46737	99.52123
2130	99.63721	99.79031	99.46745	99.52245
2129	99.64012	99.79053	99.46752	99.52365
2128	99.64225	99.79078	99.46757	99.52484
2127	99.64354	99.79106	99.4676	99.52602
2126	99.64405	99.79137	99.46762	99.52718
2125	99.64397	99.79172	99.46762	99.52833
2124	99.64354	99.7921	99.4676	99.52946
2123	99.64293	99.79251	99.46757	99.53058
2122	99.64221	99.79296	99.46752	99.53167
2121	99.64131	99.79344	99.46745	99.53275
2120	99.6401	99.79395	99.46737	99.53382
2119	99.63847	99.7945	99.46727	99.53486

2118	99.63643	99.79508	99.46716	99.53588
2117	99.63414	99.79569	99.46703	99.53689
2116	99.63186	99.79634	99.46688	99.53787
2115	99.62982	99.79702	99.46672	99.53883
2114	99.6282	99.79773	99.46654	99.53977
2113	99.627	99.79848	99.46634	99.54069
2112	99.62619	99.79927	99.46613	99.54159
2111	99.64021	99.80008	99.4659	99.54246
2110	99.63852	99.80093	99.46566	99.54332
2109	99.63604	99.80182	99.4654	99.54414
2108	99.63296	99.80274	99.46513	99.54495
2107	99.62956	99.80369	99.46484	99.54573
2106	99.62608	99.80468	99.46454	99.54648
2105	99.6227	99.8057	99.46422	99.54721
2104	99.61957	99.80675	99.46389	99.54792
2103	99.61684	99.80784	99.46354	99.54859
2102	99.61467	99.80897	99.46318	99.54925
2101	99.61322	99.81012	99.4628	99.54987
2100	99.61264	99.81131	99.46242	99.55047
2099	99.61297	99.81254	99.46201	99.55104
2098	99.61413	99.8138	99.46159	99.55158
2097	99.61589	99.81509	99.46116	99.55209
2096	99.61794	99.81642	99.46072	99.55258
2095	99.61998	99.81778	99.46026	99.55304
2094	99.62178	99.81917	99.4598	99.55346
2093	99.62323	99.8206	99.45931	99.55386
2092	99.6243	99.82206	99.45882	99.55423
2091	99.625	99.82355	99.45831	99.55457
2090	99.62537	99.82508	99.4578	99.55488
2089	99.62545	99.82664	99.45727	99.55516
2088	99.6253	99.82823	99.45672	99.55541
2087	99.62503	99.82985	99.45617	99.55563
2086	99.62219	99.83151	99.45561	99.55581
2085	99.62273	99.8332	99.45504	99.55597
2084	99.62362	99.83492	99.45445	99.55609
2083	99.62491	99.83668	99.45386	99.55619
2082	99.62654	99.83846	99.45326	99.55625
2081	99.62831	99.84028	99.45264	99.55628
2080	99.62999	99.84213	99.45202	99.55628
2079	99.63139	99.84401	99.45139	99.55625
2078	99.63244	99.84592	99.45075	99.55618

2077	99.63317	99.84787	99.4501	99.55609
2076	99.63363	99.84984	99.44945	99.55596
2075	99.63386	99.85184	99.44879	99.5558
2074	99.63377	99.85388	99.44812	99.55561
2073	99.63317	99.85594	99.44744	99.55539
2072	99.63185	99.85804	99.44676	99.55513
2071	99.62967	99.86016	99.44607	99.55484
2070	99.62664	99.86231	99.44537	99.55453
2069	99.62294	99.86449	99.44467	99.55418
2068	99.61883	99.8667	99.44396	99.5538
2067	99.61456	99.86894	99.44325	99.55338
2066	99.61594	99.87121	99.44254	99.55294
2065	99.61314	99.8735	99.44182	99.55247
2064	99.61213	99.87583	99.44109	99.55196
2063	99.6124	99.87817	99.44036	99.55142
2062	99.61352	99.88055	99.43963	99.55086
2061	99.61517	99.88295	99.4389	99.55026
2060	99.61715	99.88538	99.43817	99.54963
2059	99.61931	99.88784	99.43743	99.54898
2058	99.62144	99.89032	99.43669	99.54829
2057	99.62329	99.89282	99.43595	99.54758
2056	99.62457	99.89535	99.43521	99.54683
2055	99.62502	99.8979	99.43447	99.54606
2054	99.62446	99.90048	99.43373	99.54526
2053	99.6228	99.90308	99.43299	99.54443
2052	99.62001	99.90571	99.43225	99.54357
2051	99.61601	99.90836	99.43151	99.54269
2050	99.59462	99.91103	99.43078	99.54178
2049	99.59148	99.91372	99.43004	99.54084
2048	99.59014	99.91643	99.42931	99.53988
2047	99.59035	99.91917	99.42858	99.53889
2046	99.59187	99.92192	99.42785	99.53787
2045	99.59444	99.9247	99.42713	99.53684
2044	99.59779	99.92749	99.42641	99.53577
2043	99.60163	99.93031	99.4257	99.53469
2042	99.60557	99.93314	99.42499	99.53358
2041	99.60926	99.93599	99.42429	99.53245
2040	99.6124	99.93886	99.42359	99.53129
2039	99.61483	99.94175	99.4229	99.53012
2038	99.61659	99.94466	99.42221	99.52892
2037	99.6178	99.94758	99.42154	99.52771

2036	99.61866	99.95051	99.42087	99.52647
2035	99.61925	99.95347	99.4202	99.52522
2034	99.61952	99.95644	99.41955	99.52394
2033	99.61934	99.95942	99.4189	99.52265
2032	99.61856	99.96241	99.41827	99.52135
2031	99.61716	99.96542	99.41764	99.52002
2030	99.61611	99.96845	99.41702	99.51868
2029	99.61335	99.97148	99.41642	99.51733
2028	99.61136	99.97453	99.41582	99.51596
2027	99.61055	99.97759	99.41523	99.51458
2026	99.61103	99.98066	99.41466	99.51318
2025	99.6126	99.98374	99.4141	99.51178
2024	99.61483	99.98683	99.41355	99.51036
2023	99.61728	99.98993	99.41301	99.50893
2022	99.6196	99.99303	99.41249	99.50749
2021	99.62166	99.99615	99.41198	99.50604
2020	99.62347	99.99927	99.41148	99.50459
2019	99.62512	100.0024	99.411	99.50313
2018	99.62663	100.00554	99.41054	99.50166
2017	99.62795	100.00868	99.41008	99.50019
2016	99.62897	100.01183	99.40965	99.49871
2015	99.62964	100.01498	99.40923	99.49723
2014	99.62998	100.01813	99.40882	99.49574
2013	99.63015	100.02129	99.40844	99.49426
2012	99.63034	100.02445	99.40807	99.49277
2011	99.63071	100.02762	99.40771	99.49128
2010	99.62297	100.03078	99.40738	99.48979
2009	99.62319	100.03395	99.40706	99.48831
2008	99.62241	100.03711	99.40676	99.48683
2007	99.62065	100.04028	99.40648	99.48535
2006	99.61804	100.04344	99.40622	99.48387
2005	99.6149	100.04661	99.40598	99.4824
2004	99.61158	100.04977	99.40576	99.48094
2003	99.60837	100.05292	99.40556	99.47948
2002	99.60546	100.05608	99.40537	99.47804
2001	99.60292	100.05923	99.40521	99.4766
2000	99.60076	100.06237	99.40508	99.47517
1999	99.59905	100.06551	99.40496	99.47375
1998	99.5979	100.06864	99.40486	99.47235
1997	99.59747	100.07177	99.40479	99.47096
1996	99.59784	100.07489	99.40474	99.46958

1995	99.59897	100.078	99.40471	99.46822
1994	99.60066	100.0811	99.4047	99.46687
1993	99.60258	100.0842	99.40472	99.46554
1992	99.60446	100.08728	99.40476	99.46423
1991	99.60616	100.09035	99.40482	99.46294
1990	99.6078	100.09341	99.40491	99.46166
1989	99.6096	100.09646	99.40502	99.46041
1988	99.61182	100.09949	99.40516	99.45918
1987	99.6145	100.10252	99.40532	99.45798
1986	99.61745	100.10552	99.40551	99.45679
1985	99.62027	100.10852	99.40572	99.45564
1984	99.62251	100.11149	99.40596	99.4545
1983	99.6239	100.11445	99.40622	99.4534
1982	99.62577	100.1174	99.40651	99.45232
1981	99.62566	100.12033	99.40682	99.45127
1980	99.6252	100.12324	99.40716	99.45025
1979	99.62503	100.12613	99.40752	99.44926
1978	99.62563	100.129	99.40792	99.4483
1977	99.62721	100.13185	99.40834	99.44738
1976	99.62976	100.13467	99.40878	99.44649
1975	99.6331	100.13748	99.40925	99.44563
1974	99.63703	100.14027	99.40975	99.44481
1973	99.64141	100.14303	99.41028	99.44402
1972	99.64611	100.14577	99.41083	99.44327
1971	99.65102	100.14848	99.41141	99.44256
1970	99.6559	100.15117	99.41202	99.44188
1969	99.6605	100.15384	99.41266	99.44125
1968	99.6646	100.15648	99.41332	99.44066
1967	99.66806	100.15909	99.41401	99.44011
1966	99.67097	100.16167	99.41473	99.43959
1965	99.67351	100.16422	99.41547	99.43913
1964	99.67591	100.16675	99.41625	99.4387
1963	99.67825	100.16925	99.41705	99.43832
1962	99.6805	100.17171	99.41788	99.43799
1961	99.68241	100.17415	99.41873	99.4377
1960	99.68373	100.17655	99.41962	99.43746
1959	99.68426	100.17892	99.42053	99.43727
1958	99.68394	100.18126	99.42147	99.43712
1957	99.68286	100.18356	99.42244	99.43702
1956	99.68112	100.18583	99.42343	99.43697
1955	99.67879	100.18807	99.42445	99.43697

1954	99.6758	100.19027	99.4255	99.43703
1953	99.67205	100.19243	99.42658	99.43713
1952	99.66744	100.19456	99.42768	99.43729
1951	99.66201	100.19665	99.42881	99.43749
1950	99.65603	100.1987	99.42997	99.43776
1949	99.64987	100.20071	99.43116	99.43807
1948	99.64396	100.20268	99.43237	99.43844
1947	99.63859	100.20462	99.43361	99.43886
1946	99.63383	100.20651	99.43487	99.43934
1945	99.62955	100.20836	99.43616	99.43987
1944	99.62186	100.21017	99.43748	99.44046
1943	99.61798	100.21193	99.43882	99.44111
1942	99.61421	100.21366	99.44019	99.44181
1941	99.61065	100.21534	99.44158	99.44257
1940	99.60742	100.21697	99.443	99.44339
1939	99.60453	100.21856	99.44445	99.44426
1938	99.60182	100.22011	99.44591	99.4452
1937	99.59891	100.22161	99.44741	99.44619
1936	99.59538	100.22306	99.44892	99.44724
1935	99.5909	100.22446	99.45047	99.44835
1934	99.58536	100.22582	99.45203	99.44951
1933	99.57895	100.22713	99.45362	99.45074
1932	99.57205	100.22839	99.45523	99.45202
1931	99.56518	100.2296	99.45686	99.45337
1930	99.55878	100.23076	99.45852	99.45477
1929	99.55324	100.23187	99.4602	99.45623
1928	99.54881	100.23292	99.4619	99.45776
1927	99.5457	100.23393	99.46362	99.45934
1926	99.5441	100.23488	99.46536	99.46098
1925	99.54416	100.23579	99.46712	99.46268
1924	99.54594	100.23663	99.4689	99.46444
1923	99.54931	100.23743	99.4707	99.46625
1922	99.55396	100.23817	99.47252	99.46813
1921	99.55944	100.23885	99.47436	99.47007
1920	99.56522	100.23948	99.47622	99.47206
1919	99.57089	100.24006	99.4781	99.47411
1918	99.57623	100.24058	99.47999	99.47622
1917	99.58118	100.24104	99.4819	99.47838
1916	99.58581	100.24144	99.48382	99.48061
1915	99.5902	100.24179	99.48576	99.48289
1914	99.59435	100.24208	99.48772	99.48522

1913	99.5982	100.24231	99.48969	99.48762
1912	99.60167	100.24248	99.49168	99.49006
1911	99.60479	100.24259	99.49368	99.49256
1910	99.60772	100.24264	99.49569	99.49512
1909	99.61073	100.24263	99.49772	99.49773
1908	99.61411	100.24256	99.49975	99.50039
1907	99.61803	100.24243	99.5018	99.50311
1906	99.62244	100.24224	99.50386	99.50587
1905	99.65896	100.24198	99.50593	99.50869
1904	99.66328	100.24167	99.50801	99.51156
1903	99.66645	100.24129	99.51009	99.51448
1902	99.66839	100.24085	99.51219	99.51744
1901	99.66918	100.24034	99.51429	99.52046
1900	99.66898	100.23977	99.5164	99.52352
1899	99.66797	100.23914	99.51852	99.52662
1898	99.66628	100.23844	99.52064	99.52977
1897	99.664	100.23768	99.52277	99.53297
1896	99.66121	100.23685	99.5249	99.5362
1895	99.6581	100.23596	99.52703	99.53948
1894	99.65495	100.235	99.52917	99.5428
1893	99.65214	100.23398	99.53131	99.54616
1892	99.65002	100.23288	99.53345	99.54956
1891	99.64886	100.23173	99.53559	99.55299
1890	99.64871	100.2305	99.53773	99.55646
1889	99.64947	100.22921	99.53987	99.55997
1888	99.6261	100.22786	99.542	99.56351
1887	99.62871	100.22643	99.54414	99.56708
1886	99.63051	100.22494	99.54627	99.57068
1885	99.63132	100.22338	99.5484	99.57431
1884	99.631	100.22175	99.55052	99.57797
1883	99.62948	100.22005	99.55263	99.58165
1882	99.62665	100.21828	99.55474	99.58536
1881	99.62241	100.21645	99.55685	99.58909
1880	99.61674	100.21454	99.55894	99.59284
1879	99.60977	100.21257	99.56103	99.59662
1878	99.60182	100.21053	99.5631	99.60041
1877	99.59333	100.20842	99.56517	99.60422
1876	99.5848	100.20624	99.56722	99.60804
1875	99.57665	100.20398	99.56926	99.61188
1874	99.56914	100.20166	99.57129	99.61573
1873	99.56236	100.19927	99.5733	99.61959

1872	99.55629	100.19681	99.5753	99.62345
1871	99.55089	100.19428	99.57728	99.62733
1870	99.54621	100.19168	99.57925	99.6312
1869	99.54237	100.18901	99.5812	99.63508
1868	99.53946	100.18627	99.58313	99.63896
1867	99.53749	100.18346	99.58503	99.64284
1866	99.53629	100.18058	99.58692	99.64671
1865	99.53554	100.17763	99.58879	99.65058
1864	99.53491	100.17461	99.59064	99.65444
1863	99.5341	100.17151	99.59246	99.65829
1862	99.53299	100.16835	99.59426	99.66213
1861	99.53161	100.16512	99.59603	99.66595
1860	99.53011	100.16182	99.59778	99.66976
1859	99.52866	100.15844	99.5995	99.67355
1858	99.52737	100.155	99.6012	99.67732
1857	99.52627	100.15148	99.60286	99.68106
1856	99.52539	100.1479	99.6045	99.68478
1855	99.52475	100.14425	99.6061	99.68848
1854	99.52446	100.14052	99.60767	99.69214
1853	99.52467	100.13673	99.60921	99.69577
1852	99.52555	100.13286	99.61072	99.69937
1851	99.5272	100.12893	99.6122	99.70293
1850	99.5296	100.12493	99.61363	99.70645
1849	99.53263	100.12086	99.61504	99.70993
1848	99.5361	100.11671	99.6164	99.71336
1847	99.53983	100.1125	99.61773	99.71675
1846	99.54376	100.10822	99.61902	99.72009
1845	99.54788	100.10388	99.62026	99.72337
1844	99.55226	100.09946	99.62147	99.72661
1843	99.55693	100.09497	99.62264	99.72978
1842	99.56185	100.09042	99.62376	99.7329
1841	99.56693	100.0858	99.62484	99.73596
1840	99.5721	100.08111	99.62588	99.73895
1839	99.57735	100.07635	99.62687	99.74187
1838	99.58281	100.07153	99.62781	99.74473
1837	99.58868	100.06664	99.6287	99.74751
1836	99.59516	100.06168	99.62955	99.75022
1835	99.60237	100.05666	99.63035	99.75285
1834	99.61025	100.05157	99.6311	99.7554
1833	99.61861	100.04641	99.6318	99.75787
1832	99.62984	100.04119	99.63245	99.76025

1831	99.63806	100.03591	99.63304	99.76255
1830	99.6455	100.03056	99.63358	99.76475
1829	99.65215	100.02514	99.63407	99.76686
1828	99.65801	100.01966	99.6345	99.76888
1827	99.66308	100.01412	99.63487	99.77079
1826	99.66724	100.00851	99.63519	99.77261
1825	99.67033	100.00284	99.63545	99.77432
1824	99.67222	99.99711	99.63565	99.77592
1823	99.67291	99.99131	99.63579	99.77741
1822	99.6726	99.98546	99.63587	99.77879
1821	99.67159	99.97954	99.63589	99.78006
1820	99.6702	99.97356	99.63584	99.78121
1819	99.66867	99.96752	99.63574	99.78224
1818	99.66704	99.96142	99.63557	99.78314
1817	99.66518	99.95526	99.63533	99.78392
1816	99.6629	99.94905	99.63503	99.78457
1815	99.66005	99.94277	99.63467	99.78509
1814	99.65664	99.93643	99.63424	99.78547
1813	99.6528	99.93004	99.63373	99.78572
1812	99.64879	99.92359	99.63317	99.78583
1811	99.64481	99.91708	99.63253	99.7858
1810	99.64103	99.91052	99.63182	99.78562
1809	99.63748	99.9039	99.63104	99.78529
1808	99.63414	99.89723	99.63019	99.78482
1807	99.63099	99.8905	99.62927	99.78419
1806	99.62814	99.88372	99.62827	99.78341
1805	99.62578	99.87688	99.6272	99.78247
1804	99.62524	99.86999	99.62606	99.78136
1803	99.62399	99.86305	99.62484	99.7801
1802	99.6228	99.85606	99.62355	99.77867
1801	99.62167	99.84902	99.62218	99.77707
1800	99.62054	99.84192	99.62073	99.7753
1799	99.61938	99.83478	99.61921	99.77336
1798	99.61824	99.82759	99.6176	99.77124
1797	99.61725	99.82035	99.61592	99.76894
1796	99.61654	99.81306	99.61416	99.76646
1795	99.61619	99.80572	99.61232	99.7638
1794	99.61614	99.79834	99.6104	99.76096
1793	99.61621	99.79091	99.6084	99.75792
1792	99.61622	99.78343	99.60632	99.7547
1791	99.61602	99.77591	99.60416	99.75128

1790	99.61563	99.76835	99.60191	99.74767
1789	99.61517	99.76075	99.59958	99.74386
1788	99.61479	99.7531	99.59716	99.73986
1787	99.61462	99.74541	99.59467	99.73565
1786	99.61468	99.73768	99.59209	99.73123
1785	99.6149	99.7299	99.58942	99.72661
1784	99.61522	99.72209	99.58667	99.72178
1783	99.61568	99.71424	99.58383	99.71675
1782	99.61645	99.70635	99.58091	99.7115
1781	99.61706	99.69843	99.5779	99.70603
1780	99.6184	99.69047	99.5748	99.70035
1779	99.61949	99.68247	99.57162	99.69445
1778	99.62032	99.67444	99.56835	99.68833
1777	99.62069	99.66637	99.56499	99.68199
1776	99.62041	99.65827	99.56155	99.67542
1775	99.61944	99.65013	99.55801	99.66863
1774	99.61797	99.64197	99.55439	99.66161
1773	99.61638	99.63377	99.55068	99.65436
1772	99.61505	99.62555	99.54688	99.64688
1771	99.61424	99.61729	99.54299	99.63917
1770	99.61392	99.60901	99.53901	99.63122
1769	99.61381	99.60069	99.53494	99.62304
1768	99.61351	99.59235	99.53079	99.61462
1767	99.61271	99.58399	99.52654	99.60596
1766	99.61126	99.5756	99.5222	99.59706
1765	99.60925	99.56718	99.51778	99.58792
1764	99.60684	99.55874	99.51326	99.57853
1763	99.60413	99.55028	99.50865	99.5689
1762	99.601	99.5418	99.50396	99.55903
1761	99.61066	99.53329	99.49917	99.54891
1760	99.61111	99.52477	99.4943	99.53853
1759	99.61692	99.51622	99.48933	99.52792
1758	99.62492	99.50766	99.48427	99.51705
1757	99.63209	99.49908	99.47913	99.50592
1756	99.63544	99.49049	99.47389	99.49455
1755	99.63183	99.48187	99.46857	99.48292
1754	99.61786	99.47325	99.46315	99.47104
1753	99.56657	99.46461	99.45764	99.45891
1752	99.52843	99.45595	99.45205	99.44652
1751	99.47984	99.44729	99.44637	99.43387
1750	99.41966	99.43861	99.4406	99.42097

1749	99.34683	99.42992	99.43473	99.4078
1748	99.26034	99.42122	99.42878	99.39438
1747	99.15901	99.41252	99.42275	99.3807
1746	99.04136	99.40381	99.41662	99.36677
1745	98.90548	99.39509	99.41041	99.35257
1744	98.7491	99.38636	99.40411	99.33811
1743	98.56969	99.37763	99.39772	99.3234
1742	98.36465	99.3689	99.39124	99.30842
1741	98.13139	99.36016	99.38468	99.29318
1740	97.86737	99.35142	99.37804	99.27769
1739	97.57001	99.34268	99.3713	99.26193
1738	97.23676	99.33394	99.36449	99.24591
1737	96.86512	99.3252	99.35758	99.22963
1736	96.45283	99.31646	99.3506	99.2131
1735	95.99801	99.30773	99.34353	99.1963
1734	95.49919	99.299	99.33637	99.17924
1733	94.95519	99.29027	99.32914	99.16192
1732	94.36481	99.28155	99.32182	99.14435
1731	93.7265	99.27283	99.31442	99.12651
1730	93.03816	99.26413	99.30694	99.10842
1729	92.29711	99.25543	99.29938	99.09007
1728	91.50048	99.24674	99.29174	99.07147
1727	90.64575	99.23806	99.28402	99.05261
1726	89.73118	99.22939	99.27622	99.03349
1725	88.75601	99.22073	99.26834	99.01412
1724	87.72026	99.21209	99.26039	98.9945
1723	86.62438	99.20345	99.25236	98.97462
1722	85.46895	99.19484	99.24425	98.95449
1721	84.25459	99.18624	99.23607	98.93411
1720	82.98203	99.17766	99.22782	98.91348
1719	81.65233	99.16909	99.21949	98.89261
1718	80.267	99.16054	99.21109	98.87149
1717	78.82806	99.15202	99.20262	98.85012
1716	77.33786	99.14351	99.19408	98.82851
1715	75.79893	99.13502	99.18547	98.80665
1714	74.21382	99.12656	99.17679	98.78456
1713	72.58514	99.11812	99.16804	98.76222
1712	70.91572	99.10971	99.15922	98.73965
1711	69.20881	99.10131	99.15034	98.71684
1710	67.46813	99.09295	99.14139	98.69379
1709	65.6979	99.08461	99.13238	98.67052

1708	63.90259	99.0763	99.1233	98.64701
1707	62.08678	99.06802	99.11416	98.62327
1706	60.25494	99.05977	99.10496	98.59931
1705	58.41145	99.05154	99.0957	98.57512
1704	56.56063	99.04335	99.08638	98.55071
1703	54.70689	99.03519	99.07701	98.52608
1702	52.85492	99.02707	99.06757	98.50123
1701	51.0097	99.01898	99.05808	98.47617
1700	49.17645	99.01092	99.04854	98.45089
1699	47.36041	99.0029	99.03894	98.4254
1698	45.5666	98.99492	99.02928	98.3997
1697	43.79967	98.98697	99.01958	98.3738
1696	42.06381	98.97906	99.00983	98.34769
1695	40.36283	98.97119	99.00002	98.32138
1694	38.70021	98.96336	98.99017	98.29487
1693	37.07903	98.95557	98.98027	98.26817
1692	35.50196	98.94783	98.97033	98.24128
1691	33.97118	98.94012	98.96034	98.21419
1690	32.48833	98.93246	98.95031	98.18692
1689	31.05472	98.92485	98.94024	98.15946
1688	29.67138	98.91728	98.93013	98.13182
1687	28.33932	98.90975	98.91998	98.10401
1686	27.05961	98.90227	98.90979	98.07602
1685	25.83342	98.89484	98.89956	98.04786
1684	24.66176	98.88746	98.8893	98.01953
1683	23.54532	98.88012	98.879	97.99103
1682	22.48421	98.87284	98.86868	97.96237
1681	21.47795	98.86561	98.85832	97.93356
1680	20.52562	98.85842	98.84793	97.90459
1679	19.626	98.85129	98.83751	97.87547
1678	18.77773	98.84422	98.82707	97.8462
1677	17.97936	98.83719	98.8166	97.81678
1676	17.22937	98.83022	98.8061	97.78723
1675	16.52618	98.82331	98.79559	97.75754
1674	15.86814	98.81645	98.78505	97.72771
1673	15.25357	98.80965	98.77449	97.69776
1672	14.68082	98.80291	98.76391	97.66767
1671	14.14828	98.79622	98.75332	97.63747
1670	13.65445	98.78959	98.74271	97.60715
1669	13.19791	98.78303	98.73208	97.57671
1668	12.77728	98.77652	98.72145	97.54616

1667	12.39121	98.77007	98.7108	97.51551
1666	12.03837	98.76368	98.70014	97.48475
1665	11.71744	98.75736	98.68947	97.4539
1664	11.42716	98.7511	98.6788	97.42295
1663	11.16635	98.7449	98.66812	97.39191
1662	10.93401	98.73877	98.65744	97.36078
1661	10.72921	98.7327	98.64676	97.32958
1660	10.55117	98.72669	98.63607	97.29829
1659	10.39918	98.72076	98.62539	97.26693
1658	10.27258	98.71488	98.6147	97.2355
1657	10.17073	98.70908	98.60402	97.20401
1656	10.09303	98.70334	98.59335	97.17245
1655	10.03893	98.69767	98.58268	97.14084
1654	10.00801	98.69207	98.57202	97.10918
1653	10	98.68654	98.56137	97.07747
1652	10.01473	98.68108	98.55074	97.04571
1651	10.05214	98.67569	98.54011	97.01392
1650	10.11221	98.67037	98.5295	96.98209
1649	10.19495	98.66512	98.51891	96.95023
1648	10.30043	98.65994	98.50833	96.91835
1647	10.42874	98.65484	98.49777	96.88644
1646	10.58011	98.6498	98.48723	96.85452
1645	10.75482	98.64484	98.47671	96.82259
1644	10.95321	98.63996	98.46622	96.79065
1643	11.17567	98.63515	98.45575	96.7587
1642	11.42253	98.63041	98.44531	96.72676
1641	11.69409	98.62575	98.43489	96.69482
1640	11.99056	98.62116	98.4245	96.6629
1639	12.3121	98.61665	98.41415	96.63099
1638	12.65891	98.61222	98.40383	96.5991
1637	13.03119	98.60786	98.39354	96.56723
1636	13.42913	98.60358	98.38328	96.53539
1635	13.85291	98.59938	98.37307	96.50359
1634	14.30271	98.59525	98.36289	96.47183
1633	14.77869	98.59121	98.35275	96.4401
1632	15.28106	98.58724	98.34265	96.40843
1631	15.81005	98.58335	98.33259	96.37681
1630	16.36588	98.57954	98.32258	96.34524
1629	16.94878	98.5758	98.31261	96.31373
1628	17.5589	98.57215	98.30269	96.2823
1627	18.19633	98.56858	98.29281	96.25093

1626	18.86102	98.56509	98.28299	96.21963
1625	19.55281	98.56167	98.27322	96.18842
1624	20.2714	98.55834	98.2635	96.15729
1623	21.0164	98.55509	98.25383	96.12625
1622	21.78733	98.55192	98.24422	96.0953
1621	22.58367	98.54883	98.23466	96.06445
1620	23.40484	98.54582	98.22516	96.0337
1619	24.25021	98.5429	98.21572	96.00306
1618	25.11904	98.54005	98.20634	95.97253
1617	26.01046	98.53729	98.19702	95.94211
1616	26.92348	98.53461	98.18776	95.91181
1615	27.85695	98.53201	98.17857	95.88164
1614	28.80962	98.5295	98.16944	95.8516
1613	29.78017	98.52707	98.16038	95.82169
1612	30.76724	98.52472	98.15139	95.79191
1611	31.7694	98.52245	98.14246	95.76228
1610	32.78513	98.52026	98.13361	95.73279
1609	33.81278	98.51816	98.12482	95.70346
1608	34.85055	98.51614	98.11611	95.67428
1607	35.8966	98.5142	98.10747	95.64525
1606	36.94902	98.51235	98.0989	95.61639
1605	38.00595	98.51058	98.09042	95.58769
1604	39.06563	98.50889	98.082	95.55917
1603	40.12641	98.50728	98.07367	95.53082
1602	41.18679	98.50576	98.06541	95.50265
1601	42.24538	98.50432	98.05723	95.47466
1600	43.30081	98.50296	98.04913	95.44686
1599	44.35167	98.50168	98.04112	95.41925
1598	45.39646	98.50049	98.03319	95.39183
1597	46.43368	98.49938	98.02534	95.36461
1596	47.46187	98.49835	98.01757	95.33759
1595	48.47968	98.49741	98.00989	95.31078
1594	49.48599	98.49654	98.0023	95.28417
1593	50.47988	98.49576	97.99479	95.25778
1592	51.46068	98.49506	97.98737	95.23161
1591	52.42795	98.49445	97.98004	95.20565
1590	53.38137	98.49391	97.97279	95.17992
1589	54.32067	98.49345	97.96564	95.15441
1588	55.24555	98.49308	97.95858	95.12913
1587	56.15569	98.49279	97.95161	95.10409
1586	57.05078	98.49258	97.94473	95.07928

1585	57.93055	98.49244	97.93795	95.05472
1584	58.7947	98.49239	97.93126	95.03039
1583	59.64296	98.49242	97.92466	95.00631
1582	60.47499	98.49253	97.91816	94.98248
1581	61.29052	98.49272	97.91175	94.9589
1580	62.08937	98.49299	97.90544	94.93557
1579	62.87151	98.49333	97.89922	94.91251
1578	63.63706	98.49376	97.8931	94.8897
1577	64.38622	98.49426	97.88708	94.86715
1576	65.11913	98.49484	97.88115	94.84487
1575	65.83587	98.4955	97.87533	94.82286
1574	66.53643	98.49624	97.8696	94.80112
1573	67.2208	98.49705	97.86397	94.77965
1572	67.88906	98.49794	97.85843	94.75846
1571	68.54148	98.4989	97.853	94.73754
1570	69.17849	98.49994	97.84767	94.71691
1569	69.80064	98.50106	97.84244	94.69655
1568	70.4085	98.50225	97.8373	94.67648
1567	71.00256	98.50352	97.83227	94.6567
1566	71.58318	98.50486	97.82734	94.6372
1565	72.15067	98.50627	97.82251	94.61799
1564	72.70533	98.50775	97.81777	94.59908
1563	73.2475	98.50931	97.81314	94.58045
1562	73.7776	98.51094	97.80861	94.56213
1561	74.29615	98.51265	97.80418	94.54409
1560	74.80359	98.51442	97.79985	94.52636
1559	75.30013	98.51626	97.79562	94.50893
1558	75.78567	98.51818	97.79149	94.49179
1557	76.25986	98.52016	97.78747	94.47496
1556	76.72238	98.52222	97.78354	94.45843
1555	77.17313	98.52434	97.77971	94.44221
1554	77.6123	98.52653	97.77598	94.42629
1553	78.04023	98.52878	97.77235	94.41068
1552	78.45717	98.5311	97.76882	94.39537
1551	78.86306	98.53349	97.76539	94.38037
1550	79.25753	98.53595	97.76205	94.36569
1549	79.63998	98.53847	97.75882	94.35131
1548	80.00968	98.54105	97.75568	94.33723
1547	80.36586	98.5437	97.75264	94.32347
1546	80.70774	98.54641	97.7497	94.31002
1545	81.0344	98.54918	97.74685	94.29689

1544	81.3448	98.55202	97.7441	94.28406
1543	81.63763	98.55491	97.74145	94.27154
1542	81.91133	98.55787	97.73889	94.25933
1541	82.16417	98.56088	97.73642	94.24744
1540	82.39449	98.56396	97.73405	94.23586
1539	82.60096	98.56709	97.73177	94.22458
1538	82.78281	98.57028	97.72959	94.21362
1537	82.93983	98.57352	97.72749	94.20297
1536	83.07222	98.57683	97.72549	94.19263
1535	83.18043	98.58019	97.72357	94.18259
1534	83.26511	98.5836	97.72175	94.17287
1533	83.32716	98.58706	97.72001	94.16345
1532	83.36771	98.59058	97.71836	94.15434
1531	83.38812	98.59416	97.7168	94.14554
1530	83.38989	98.59778	97.71532	94.13704
1529	83.37452	98.60145	97.71393	94.12884
1528	83.34333	98.60518	97.71262	94.12095
1527	83.29736	98.60895	97.71139	94.11336
1526	83.23736	98.61277	97.71025	94.10607
1525	83.16385	98.61664	97.70918	94.09908
1524	83.07725	98.62056	97.7082	94.09239
1523	82.97795	98.62452	97.70729	94.08599
1522	82.86639	98.62853	97.70646	94.07989
1521	82.74298	98.63258	97.70571	94.07408
1520	82.60796	98.63667	97.70503	94.06856
1519	82.4612	98.64081	97.70443	94.06333
1518	82.30212	98.64499	97.70389	94.05839
1517	82.12971	98.64921	97.70343	94.05373
1516	81.94269	98.65347	97.70304	94.04936
1515	81.73976	98.65777	97.70271	94.04527
1514	81.51967	98.6621	97.70246	94.04145
1513	81.28119	98.66648	97.70226	94.03791
1512	81.02289	98.67089	97.70214	94.03464
1511	80.74299	98.67533	97.70207	94.03165
1510	80.43944	98.67982	97.70206	94.02892
1509	80.11019	98.68433	97.70212	94.02646
1508	79.75363	98.68888	97.70223	94.02426
1507	79.36896	98.69346	97.7024	94.02233
1506	78.95626	98.69807	97.70262	94.02065
1505	78.51643	98.70271	97.7029	94.01922
1504	78.05093	98.70738	97.70323	94.01804

1503	77.56163	98.71208	97.7036	94.01712
1502	77.0509	98.71681	97.70403	94.01644
1501	76.52168	98.72156	97.7045	94.01599
1500	75.97754	98.72634	97.70502	94.01579
1499	75.42258	98.73114	97.70557	94.01582
1498	74.86118	98.73597	97.70618	94.01609
1497	74.29769	98.74082	97.70681	94.01658
1496	73.73602	98.74569	97.70749	94.01729
1495	73.17932	98.75058	97.7082	94.01823
1494	72.62968	98.75549	97.70895	94.01938
1493	72.0881	98.76042	97.70973	94.02075
1492	71.55452	98.76537	97.71054	94.02232
1491	71.02797	98.77034	97.71137	94.0241
1490	70.50668	98.77532	97.71223	94.02607
1489	69.98813	98.78032	97.71312	94.02825
1488	69.46931	98.78533	97.71403	94.03062
1487	68.94687	98.79036	97.71495	94.03317
1486	68.41741	98.79539	97.7159	94.03591
1485	67.87767	98.80044	97.71686	94.03883
1484	67.32467	98.8055	97.71784	94.04193
1483	66.75582	98.81057	97.71883	94.04519
1482	66.16891	98.81565	97.71982	94.04862
1481	65.56212	98.82073	97.72083	94.05221
1480	64.93398	98.82582	97.72184	94.05596
1479	64.28333	98.83092	97.72285	94.05987
1478	63.60936	98.83602	97.72387	94.06391
1477	62.9116	98.84112	97.72488	94.06811
1476	62.19009	98.84623	97.72589	94.07243
1475	61.44544	98.85134	97.7269	94.0769
1474	60.67894	98.85645	97.72789	94.08149
1473	59.89243	98.86156	97.72888	94.0862
1472	59.08814	98.86667	97.72986	94.09103
1471	58.26845	98.87178	97.73082	94.09597
1470	57.43573	98.87688	97.73177	94.10102
1469	56.5924	98.88198	97.73269	94.10618
1468	55.74106	98.88707	97.7336	94.11143
1467	54.88455	98.89216	97.73448	94.11677
1466	54.02606	98.89724	97.73534	94.1222
1465	53.16902	98.90232	97.73617	94.12771
1464	52.3169	98.90738	97.73697	94.1333
1463	51.47299	98.91244	97.73773	94.13895

1462	50.64022	98.91749	97.73846	94.14468
1461	49.82102	98.92252	97.73916	94.15046
1460	49.01728	98.92754	97.73981	94.15629
1459	48.23027	98.93255	97.74043	94.16218
1458	47.46055	98.93754	97.741	94.16811
1457	46.70789	98.94252	97.74152	94.17407
1456	45.9714	98.94749	97.74199	94.18007
1455	45.24978	98.95243	97.74242	94.18609
1454	44.5416	98.95736	97.74279	94.19214
1453	43.84565	98.96227	97.7431	94.1982
1452	43.16108	98.96716	97.74336	94.20427
1451	42.48753	98.97203	97.74355	94.21034
1450	41.82513	98.97687	97.74369	94.21641
1449	41.17451	98.9817	97.74375	94.22247
1448	40.53673	98.9865	97.74375	94.22852
1447	39.91331	98.99128	97.74369	94.23454
1446	39.30609	98.99603	97.74354	94.24055
1445	38.71716	99.00076	97.74333	94.24652
1444	38.1488	99.00546	97.74304	94.25246
1443	37.6033	99.01013	97.74267	94.25835
1442	37.08292	99.01477	97.74221	94.26419
1441	36.58982	99.01939	97.74168	94.26998
1440	36.12597	99.02397	97.74105	94.27571
1439	35.69317	99.02852	97.74034	94.28137
1438	35.29301	99.03305	97.73954	94.28697
1437	34.92686	99.03754	97.73865	94.29248
1436	34.5958	99.04199	97.73766	94.29791
1435	34.30073	99.04641	97.73657	94.30325
1434	34.04227	99.0508	97.73539	94.3085
1433	33.82086	99.05515	97.7341	94.31364
1432	33.63681	99.05947	97.73271	94.31868
1431	33.49024	99.06374	97.73121	94.32361
1430	33.38121	99.06798	97.72961	94.32842
1429	33.30973	99.07219	97.72789	94.3331
1428	33.27583	99.07635	97.72606	94.33766
1427	33.27968	99.08047	97.72412	94.34207
1426	33.3216	99.08455	97.72206	94.34635
1425	33.402	99.08859	97.71988	94.35048
1424	33.52135	99.09258	97.71758	94.35446
1423	33.68	99.09653	97.71515	94.35828
1422	33.87805	99.10044	97.7126	94.36194

1421	34.11526	99.10431	97.70993	94.36543
1420	34.39094	99.10812	97.70712	94.36874
1419	34.70397	99.1119	97.70418	94.37187
1418	35.0528	99.11562	97.70111	94.37482
1417	35.4356	99.1193	97.6979	94.37758
1416	35.85042	99.12293	97.69456	94.38013
1415	36.29531	99.12652	97.69108	94.38249
1414	36.76848	99.13005	97.68745	94.38464
1413	37.26836	99.13353	97.68368	94.38658
1412	37.79362	99.13697	97.67977	94.38829
1411	38.3431	99.14035	97.67571	94.38979
1410	38.91569	99.14368	97.67151	94.39105
1409	39.51026	99.14696	97.66715	94.39209
1408	40.12563	99.15018	97.66264	94.39288
1407	40.76056	99.15336	97.65797	94.39343
1406	41.41374	99.15647	97.65315	94.39373
1405	42.08381	99.15954	97.64818	94.39377
1404	42.76933	99.16255	97.64304	94.39356
1403	43.46877	99.1655	97.63774	94.39309
1402	44.18049	99.1684	97.63229	94.39234
1401	44.90278	99.17124	97.62666	94.39132
1400	45.6338	99.17402	97.62088	94.39003
1399	46.37165	99.17675	97.61492	94.38845
1398	47.11431	99.17942	97.6088	94.38658
1397	47.8597	99.18203	97.60251	94.38442
1396	48.60565	99.18458	97.59605	94.38197
1395	49.35002	99.18707	97.58941	94.37921
1394	50.09074	99.18951	97.5826	94.37615
1393	50.82584	99.19188	97.57562	94.37279
1392	51.55356	99.19419	97.56846	94.3691
1391	52.2723	99.19644	97.56112	94.3651
1390	52.98069	99.19863	97.55361	94.36078
1389	53.67748	99.20076	97.54591	94.35613
1388	54.36161	99.20283	97.53803	94.35115
1387	55.03211	99.20483	97.52998	94.34584
1386	55.68812	99.20677	97.52173	94.34019
1385	56.32885	99.20865	97.51331	94.3342
1384	56.95355	99.21047	97.5047	94.32787
1383	57.56146	99.21222	97.4959	94.32119
1382	58.1518	99.2139	97.48691	94.31415
1381	58.72375	99.21553	97.47774	94.30676

1380	59.27654	99.21709	97.46838	94.29901
1379	59.80947	99.21858	97.45883	94.2909
1378	60.32204	99.22001	97.44909	94.28243
1377	60.81404	99.22137	97.43915	94.27359
1376	61.28565	99.22267	97.42903	94.26437
1375	61.73741	99.2239	97.41871	94.25478
1374	62.17026	99.22507	97.40821	94.24482
1373	62.5854	99.22617	97.3975	94.23448
1372	62.98416	99.22721	97.38661	94.22375
1371	63.36792	99.22818	97.37552	94.21264
1370	63.73801	99.22908	97.36423	94.20114
1369	64.09564	99.22991	97.35275	94.18925
1368	64.44191	99.23068	97.34107	94.17697
1367	64.7777	99.23138	97.3292	94.1643
1366	65.10367	99.23202	97.31713	94.15123
1365	65.42023	99.23259	97.30487	94.13776
1364	65.72749	99.23309	97.29241	94.1239
1363	66.02535	99.23352	97.27975	94.10963
1362	66.31354	99.23389	97.2669	94.09496
1361	66.59172	99.23419	97.25385	94.07988
1360	66.85956	99.23442	97.2406	94.06439
1359	67.11678	99.23458	97.22716	94.0485
1358	67.36323	99.23468	97.21352	94.0322
1357	67.59887	99.23471	97.19968	94.01549
1356	67.82376	99.23467	97.18565	93.99836
1355	68.03807	99.23457	97.17142	93.98082
1354	68.24205	99.2344	97.157	93.96287
1353	68.43596	99.23416	97.14238	93.94451
1352	68.62008	99.23386	97.12756	93.92573
1351	68.79456	99.23348	97.11256	93.90653
1350	68.9594	99.23304	97.09735	93.88692
1349	69.11438	99.23254	97.08196	93.86689
1348	69.25906	99.23196	97.06637	93.84644
1347	69.39272	99.23133	97.05059	93.82557
1346	69.51442	99.23062	97.03462	93.80429
1345	69.62288	99.22985	97.01846	93.78259
1344	69.71652	99.22901	97.00211	93.76048
1343	69.79331	99.22811	96.98557	93.73795
1342	69.85077	99.22714	96.96884	93.715
1341	69.88596	99.2261	96.95192	93.69163
1340	69.89549	99.225	96.93482	93.66785

1339	69.87565	99.22384	96.91753	93.64365
1338	69.82252	99.22261	96.90006	93.61904
1337	69.73216	99.22131	96.8824	93.59402
1336	69.60071	99.21995	96.86456	93.56858
1335	69.42457	99.21853	96.84654	93.54274
1334	69.20049	99.21704	96.82834	93.51648
1333	68.92571	99.21549	96.80997	93.48981
1332	68.59813	99.21388	96.79141	93.46274
1331	68.21636	99.2122	96.77268	93.43526
1330	67.77983	99.21047	96.75378	93.40738
1329	67.28886	99.20867	96.7347	93.37909
1328	66.74458	99.2068	96.71545	93.35041
1327	66.14885	99.20488	96.69604	93.32132
1326	65.50415	99.20289	96.67645	93.29184
1325	64.81344	99.20085	96.6567	93.26197
1324	64.08	99.19874	96.63678	93.2317
1323	63.30733	99.19658	96.6167	93.20104
1322	62.49899	99.19435	96.59646	93.17
1321	61.65851	99.19206	96.57606	93.13857
1320	60.78927	99.18972	96.5555	93.10676
1319	59.8944	99.18732	96.53479	93.07457
1318	58.9767	99.18486	96.51392	93.04201
1317	58.03863	99.18234	96.4929	93.00907
1316	57.08231	99.17977	96.47174	92.97576
1315	56.10952	99.17714	96.45042	92.94209
1314	55.12183	99.17445	96.42896	92.90805
1313	54.12063	99.17171	96.40736	92.87366
1312	53.10723	99.16892	96.38561	92.83891
1311	52.08294	99.16607	96.36373	92.8038
1310	51.04909	99.16316	96.34171	92.76835
1309	50.00716	99.1602	96.31956	92.73255
1308	48.95875	99.15719	96.29727	92.69641
1307	47.90569	99.15413	96.27486	92.65994
1306	46.84995	99.15102	96.25232	92.62313
1305	45.79375	99.14785	96.22966	92.58599
1304	44.73946	99.14463	96.20687	92.54853
1303	43.68964	99.14137	96.18397	92.51076
1302	42.64698	99.13805	96.16095	92.47266
1301	41.61436	99.13469	96.13782	92.43426
1300	40.59483	99.13128	96.11457	92.39555
1299	39.59157	99.12782	96.09122	92.35654

1298	38.60794	99.12431	96.06777	92.31724
1297	37.64741	99.12076	96.04421	92.27765
1296	36.71344	99.11716	96.02055	92.23777
1295	35.80945	99.11352	95.99679	92.19761
1294	34.93868	99.10983	95.97295	92.15718
1293	34.10409	99.1061	95.94901	92.11647
1292	33.30832	99.10232	95.92498	92.07551
1291	32.55362	99.09851	95.90087	92.03429
1290	31.84177	99.09465	95.87668	91.99281
1289	31.17412	99.09075	95.8524	91.95109
1288	30.55145	99.08681	95.82806	91.90913
1287	29.97407	99.08283	95.80364	91.86693
1286	29.44175	99.07881	95.77915	91.82451
1285	28.95379	99.07476	95.7546	91.78186
1284	28.50911	99.07067	95.72998	91.739
1283	28.1063	99.06654	95.70531	91.69593
1282	27.74374	99.06237	95.68058	91.65265
1281	27.41969	99.05817	95.6558	91.60918
1280	27.13239	99.05393	95.63097	91.56552
1279	26.88013	99.04966	95.60609	91.52167
1278	26.66136	99.04536	95.58117	91.47765
1277	26.47472	99.04103	95.55621	91.43346
1276	26.31913	99.03666	95.53122	91.38911
1275	26.19378	99.03227	95.5062	91.34459
1274	26.09814	99.02784	95.48114	91.29993
1273	26.03193	99.02338	95.45606	91.25513
1272	25.99505	99.0189	95.43097	91.21019
1271	25.98753	99.01439	95.40585	91.16513
1270	26.00947	99.00985	95.38072	91.11994
1269	26.06099	99.00528	95.35558	91.07464
1268	26.14218	99.00069	95.33043	91.02924
1267	26.25307	98.99608	95.30528	90.98373
1266	26.39362	98.99144	95.28014	90.93814
1265	26.56366	98.98678	95.25499	90.89246
1264	26.76294	98.9821	95.22986	90.84671
1263	26.99106	98.97739	95.20473	90.80089
1262	27.24748	98.97266	95.17962	90.755
1261	27.53156	98.96792	95.15453	90.70907
1260	27.84256	98.96316	95.12947	90.66309
1259	28.1796	98.95837	95.10443	90.61708
1258	28.54174	98.95357	95.07942	90.57103

1257	28.9279	98.94876	95.05445	90.52497
1256	29.33687	98.94392	95.02952	90.47889
1255	29.76729	98.93908	95.00462	90.4328
1254	30.21761	98.93422	94.97978	90.38672
1253	30.6861	98.92934	94.95498	90.34066
1252	31.17087	98.92445	94.93024	90.29461
1251	31.66989	98.91956	94.90556	90.24859
1250	32.18101	98.91465	94.88093	90.2026
1249	32.70201	98.90973	94.85637	90.15666
1248	33.23063	98.9048	94.83189	90.11077
1247	33.76461	98.89986	94.80747	90.06495
1246	34.30173	98.89491	94.78313	90.01919
1245	34.83989	98.88996	94.75888	89.97351
1244	35.37716	98.885	94.7347	89.92792
1243	35.91184	98.88004	94.71062	89.88242
1242	36.44255	98.87507	94.68663	89.83702
1241	36.96821	98.87009	94.66274	89.79174
1240	37.48806	98.86512	94.63894	89.74657
1239	38.00165	98.86014	94.61525	89.70154
1238	38.50878	98.85516	94.59167	89.65664
1237	39.0095	98.85018	94.5682	89.61189
1236	39.50401	98.8452	94.54484	89.56729
1235	39.99263	98.84022	94.52161	89.52285
1234	40.47574	98.83524	94.4985	89.47859
1233	40.95367	98.83027	94.47551	89.4345
1232	41.42671	98.8253	94.45266	89.3906
1231	41.895	98.82033	94.42994	89.3469
1230	42.35859	98.81537	94.40736	89.3034
1229	42.81736	98.81041	94.38492	89.26012
1228	43.27113	98.80546	94.36263	89.21706
1227	43.71967	98.80051	94.34049	89.17423
1226	44.16273	98.79557	94.3185	89.13164
1225	44.60011	98.79064	94.29666	89.0893
1224	45.03166	98.78572	94.27499	89.04722
1223	45.45727	98.78081	94.25349	89.0054
1222	45.87693	98.77591	94.23215	88.96385
1221	46.2907	98.77103	94.21098	88.92258
1220	46.69875	98.76615	94.18999	88.88161
1219	47.10131	98.76129	94.16918	88.84093
1218	47.49871	98.75643	94.14855	88.80056
1217	47.89129	98.7516	94.1281	88.7605

1216	48.27943	98.74677	94.10785	88.72077
1215	48.66348	98.74197	94.08779	88.68137
1214	49.04373	98.73718	94.06792	88.64231
1213	49.42048	98.7324	94.04826	88.60359
1212	49.79396	98.72764	94.02879	88.56524
1211	50.16443	98.7229	94.00954	88.52724
1210	50.53213	98.71818	93.99049	88.48962
1209	50.89732	98.71348	93.97166	88.45238
1208	51.26024	98.7088	93.95305	88.41553
1207	51.62113	98.70413	93.93465	88.37907
1206	51.9802	98.69949	93.91648	88.34302
1205	52.33763	98.69487	93.89853	88.30738
1204	52.69359	98.69027	93.88081	88.27216
1203	53.04823	98.68569	93.86333	88.23737
1202	53.40167	98.68114	93.84608	88.20301
1201	53.75399	98.67661	93.82907	88.16909
1200	54.10523	98.67211	93.8123	88.13563
1199	54.45537	98.66762	93.79577	88.10262
1198	54.8043	98.66317	93.77949	88.07008
1197	55.15185	98.65874	93.76347	88.03801
1196	55.49778	98.65433	93.74769	88.00641
1195	55.84182	98.64996	93.73217	87.97531
1194	56.18365	98.6456	93.71691	87.9447
1193	56.52292	98.64128	93.70191	87.91459
1192	56.85926	98.63699	93.68718	87.88499
1191	57.19228	98.63272	93.67271	87.8559
1190	57.52157	98.62848	93.65851	87.82734
1189	57.84671	98.62427	93.64458	87.7993
1188	58.16734	98.62009	93.63093	87.7718
1187	58.48315	98.61594	93.61755	87.74484
1186	58.79391	98.61182	93.60445	87.71843
1185	59.09945	98.60773	93.59163	87.69257
1184	59.39974	98.60367	93.5791	87.66727
1183	59.69478	98.59964	93.56685	87.64254
1182	59.98469	98.59565	93.55489	87.61838
1181	60.26966	98.59168	93.54322	87.59481
1180	60.54997	98.58775	93.53184	87.57181
1179	60.82606	98.58385	93.52075	87.54941
1178	61.09842	98.57998	93.50996	87.5276
1177	61.3677	98.57615	93.49947	87.5064
1176	61.6346	98.57235	93.48928	87.4858

1175	61.89989	98.56858	93.47939	87.46582
1174	62.16438	98.56485	93.4698	87.44645
1173	62.42893	98.56114	93.46052	87.42771
1172	62.69441	98.55748	93.45154	87.4096
1171	62.96175	98.55384	93.44287	87.39212
1170	63.23185	98.55025	93.43451	87.37528
1169	63.50561	98.54668	93.42646	87.35908
1168	63.78385	98.54315	93.41872	87.34353
1167	64.06728	98.53965	93.4113	87.32863
1166	64.35645	98.53619	93.40419	87.31439
1165	64.65176	98.53276	93.3974	87.30081
1164	64.95343	98.52937	93.39092	87.28789
1163	65.26154	98.52601	93.38476	87.27564
1162	65.57604	98.52269	93.37892	87.26407
1161	65.89677	98.5194	93.3734	87.25317
1160	66.2235	98.51615	93.3682	87.24295
1159	66.55592	98.51293	93.36332	87.23342
1158	66.89363	98.50974	93.35877	87.22457
1157	67.23623	98.50659	93.35454	87.21641
1156	67.58327	98.50347	93.35063	87.20895
1155	67.93431	98.50039	93.34704	87.20218
1154	68.28896	98.49734	93.34378	87.19611
1153	68.64679	98.49433	93.34085	87.19074
1152	69.00745	98.49134	93.33823	87.18608
1151	69.37055	98.4884	93.33595	87.18213
1150	69.73569	98.48548	93.33399	87.17888
1149	70.10248	98.4826	93.33236	87.17635
1148	70.47049	98.47975	93.33105	87.17452
1147	70.83927	98.47694	93.33006	87.17342
1146	71.20839	98.47415	93.32941	87.17303
1145	71.57733	98.4714	93.32907	87.17336
1144	71.94557	98.46868	93.32907	87.1744
1143	72.31251	98.46599	93.32939	87.17617
1142	72.67751	98.46334	93.33003	87.17867
1141	73.03987	98.46071	93.33099	87.18188
1140	73.39889	98.45811	93.33228	87.18582
1139	73.75387	98.45555	93.3339	87.19049
1138	74.10415	98.45301	93.33583	87.19588
1137	74.44907	98.4505	93.33809	87.20199
1136	74.788	98.44802	93.34067	87.20883
1135	75.12032	98.44557	93.34356	87.2164

1134	75.4454	98.44315	93.34678	87.22469
1133	75.7626	98.44075	93.35031	87.23371
1132	76.07133	98.43838	93.35416	87.24345
1131	76.37102	98.43604	93.35833	87.25392
1130	76.66113	98.43372	93.36281	87.26512
1129	76.94119	98.43142	93.3676	87.27703
1128	77.21074	98.42915	93.3727	87.28967
1127	77.46934	98.42691	93.37812	87.30303
1126	77.71654	98.42468	93.38384	87.31711
1125	77.95194	98.42248	93.38987	87.33191
1124	78.17516	98.4203	93.3962	87.34743
1123	78.3859	98.41814	93.40284	87.36366
1122	78.58398	98.41601	93.40978	87.3806
1121	78.76935	98.41389	93.41702	87.39825
1120	78.94207	98.41179	93.42456	87.41662
1119	79.1023	98.4097	93.43239	87.43568
1118	79.25029	98.40764	93.44051	87.45546
1117	79.38637	98.40559	93.44893	87.47593
1116	79.51092	98.40355	93.45764	87.4971
1115	79.62443	98.40153	93.46663	87.51897
1114	79.72741	98.39953	93.47591	87.54153
1113	79.82049	98.39753	93.48547	87.56478
1112	79.9043	98.39555	93.4953	87.58871
1111	79.97951	98.39358	93.50542	87.61333
1110	80.04677	98.39162	93.5158	87.63862
1109	80.10671	98.38966	93.52646	87.66459
1108	80.15993	98.38772	93.53739	87.69122
1107	80.20702	98.38578	93.54858	87.71852
1106	80.24855	98.38384	93.56004	87.74648
1105	80.28508	98.38192	93.57175	87.7751
1104	80.31718	98.37999	93.58372	87.80437
1103	80.34539	98.37807	93.59594	87.83429
1102	80.37027	98.37615	93.60842	87.86484
1101	80.39236	98.37423	93.62113	87.89604
1100	80.41221	98.3723	93.6341	87.92786
1099	80.43038	98.37038	93.6473	87.96031
1098	80.44748	98.36845	93.66073	87.99338
1097	80.46407	98.36652	93.6744	88.02706
1096	80.48073	98.36458	93.6883	88.06136
1095	80.49796	98.36263	93.70242	88.09625
1094	80.51622	98.36068	93.71676	88.13174

1093	80.53588	98.35871	93.73132	88.16781
1092	80.55727	98.35674	93.7461	88.20448
1091	80.58067	98.35475	93.76108	88.24171
1090	80.60631	98.35275	93.77627	88.27952
1089	80.63441	98.35073	93.79166	88.31788
1088	80.66513	98.34869	93.80724	88.35681
1087	80.69861	98.34664	93.82302	88.39628
1086	80.73491	98.34457	93.83899	88.43629
1085	80.77409	98.34247	93.85514	88.47683
1084	80.81621	98.34036	93.87147	88.51789
1083	80.86133	98.33822	93.88797	88.55948
1082	80.90961	98.33605	93.90464	88.60157
1081	80.96122	98.33386	93.92149	88.64417
1080	81.01641	98.33164	93.93849	88.68725
1079	81.07542	98.32939	93.95564	88.73082
1078	81.13851	98.3271	93.97295	88.77487
1077	81.20589	98.32479	93.99041	88.81938
1076	81.27774	98.32243	94.00801	88.86436
1075	81.3542	98.32005	94.02574	88.90978
1074	81.43535	98.31762	94.04361	88.95564
1073	81.52121	98.31515	94.0616	89.00193
1072	81.6117	98.31264	94.07971	89.04865
1071	81.70657	98.31009	94.09794	89.09577
1070	81.80545	98.30749	94.11629	89.14331
1069	81.9078	98.30484	94.13473	89.19123
1068	82.01294	98.30215	94.15328	89.23954
1067	82.1201	98.2994	94.17193	89.28822
1066	82.22845	98.2966	94.19066	89.33726
1065	82.33716	98.29375	94.20948	89.38666
1064	82.44546	98.29084	94.22837	89.4364
1063	82.5526	98.28788	94.24734	89.48648
1062	82.65796	98.28485	94.26638	89.53688
1061	82.761	98.28176	94.28548	89.58758
1060	82.86133	98.27861	94.30463	89.6386
1059	82.9587	98.27539	94.32384	89.6899
1058	83.05302	98.2721	94.34309	89.74148
1057	83.14429	98.26875	94.36238	89.79333
1056	83.23264	98.26532	94.3817	89.84544
1055	83.31822	98.26182	94.40106	89.89779
1054	83.40116	98.25824	94.42043	89.95038
1053	83.48155	98.25458	94.43981	90.0032

1052	83.55942	98.25085	94.45921	90.05623
1051	83.63469	98.24703	94.47861	90.10946
1050	83.70723	98.24313	94.49801	90.16288
1049	83.77683	98.23914	94.51739	90.21647
1048	83.84326	98.23507	94.53676	90.27024
1047	83.90622	98.2309	94.55612	90.32415
1046	83.96544	98.22664	94.57544	90.37821
1045	84.02068	98.22229	94.59473	90.4324
1044	84.07175	98.21784	94.61398	90.48671
1043	84.11857	98.21329	94.63318	90.54113
1042	84.16116	98.20864	94.65233	90.59564
1041	84.19965	98.20389	94.67143	90.65023
1040	84.23424	98.19903	94.69045	90.7049
1039	84.26516	98.19406	94.70941	90.75962
1038	84.29266	98.18898	94.72829	90.81438
1037	84.31697	98.18379	94.74708	90.86918
1036	84.3383	98.17849	94.76578	90.924
1035	84.35688	98.17306	94.78438	90.97882
1034	84.37292	98.16752	94.80288	91.03364
1033	84.3867	98.16186	94.82127	91.08844
1032	84.39849	98.15607	94.83955	91.14322
1031	84.40863	98.15016	94.85769	91.19794
1030	84.41752	98.14411	94.87571	91.25262
1029	84.42564	98.13794	94.8936	91.30722
1028	84.43356	98.13163	94.91133	91.36174
1027	84.442	98.12519	94.92892	91.41617
1026	84.45184	98.1186	94.94636	91.47049
1025	84.46411	98.11188	94.96363	91.52469
1024	84.47995	98.10501	94.98072	91.57875
1023	84.50061	98.098	94.99765	91.63267
1022	84.52734	98.09084	95.01439	91.68643
1021	84.56138	98.08353	95.03094	91.74001
1020	84.60389	98.07607	95.04729	91.79341
1019	84.65586	98.06845	95.06345	91.84661
1018	84.71811	98.06067	95.07939	91.8996
1017	84.79124	98.05273	95.09511	91.95237
1016	84.87557	98.04463	95.11062	92.00489
1015	84.97115	98.03636	95.12589	92.05717
1014	85.07774	98.02793	95.14093	92.10918
1013	85.19493	98.01932	95.15572	92.16091
1012	85.32211	98.01054	95.17027	92.21236

1011	85.45865	98.00158	95.18456	92.2635
1010	85.60395	97.99245	95.19858	92.31432
1009	85.75752	97.98313	95.21234	92.36481
1008	85.91904	97.97364	95.22582	92.41496
1007	86.0884	97.96395	95.23902	92.46476
1006	86.26571	97.95408	95.25193	92.51419
1005	86.45126	97.94401	95.26454	92.56323
1004	86.64548	97.93375	95.27686	92.61188
1003	86.84893	97.9233	95.28886	92.66012
1002	87.06216	97.91264	95.30055	92.70795
1001	87.28569	97.90179	95.31191	92.75534
1000	87.51991	97.89072	95.32295	92.80228
999	87.76502	97.87946	95.33366	92.84876
998	88.02104	97.86798	95.34402	92.89478
997	88.28775	97.85629	95.35403	92.9403
996	88.5648	97.84438	95.36369	92.98533
995	88.85169	97.83226	95.37299	93.02986
994	89.14786	97.81991	95.38193	93.07386
993	89.45268	97.80735	95.39049	93.11732
992	89.76552	97.79455	95.39867	93.16024
991	90.0857	97.78153	95.40646	93.2026
990	90.41251	97.76828	95.41387	93.24438
989	90.7452	97.75479	95.42087	93.28558
988	91.08299	97.74107	95.42747	93.32619
987	91.42501	97.72711	95.43366	93.36619
986	91.77036	97.7129	95.43943	93.40556
985	92.11805	97.69846	95.44478	93.44431
984	92.467	97.68376	95.44971	93.48241
983	92.816	97.66881	95.45419	93.51985
982	93.16376	97.65361	95.45824	93.55663
981	93.50885	97.63816	95.46184	93.59272
980	93.84985	97.62245	95.46498	93.62813
979	94.1853	97.60648	95.46767	93.66283
978	94.51387	97.59024	95.4699	93.69682
977	94.83433	97.57374	95.47165	93.73008
976	95.14567	97.55697	95.47293	93.7626
975	95.44708	97.53992	95.47372	93.79438
974	95.73798	97.52261	95.47403	93.8254
973	96.01803	97.50501	95.47384	93.85565
972	96.28713	97.48714	95.47316	93.88511
971	96.54538	97.46898	95.47197	93.91379

970	96.79306	97.45054	95.47027	93.94166
969	97.0306	97.43181	95.46806	93.96872
968	97.25847	97.41279	95.46533	93.99496
967	97.47715	97.39348	95.46207	94.02036
966	97.68706	97.37387	95.45828	94.04492
965	97.88852	97.35396	95.45395	94.06863
964	98.08168	97.33375	95.44908	94.09147
963	98.26662	97.31324	95.44366	94.11344
962	98.44322	97.29242	95.4377	94.13452
961	98.61133	97.27129	95.43117	94.15471
960	98.77066	97.24985	95.42409	94.174
959	98.92092	97.2281	95.41644	94.19238
958	99.06182	97.20603	95.40821	94.20983
957	99.19312	97.18364	95.39941	94.22636
956	99.31466	97.16092	95.39003	94.24195
955	99.42639	97.13788	95.38006	94.25658
954	99.52835	97.11451	95.3695	94.27027
953	99.63192	97.09082	95.35835	94.28298
952	99.71409	97.06679	95.3466	94.29472
951	99.78567	97.04242	95.33424	94.30548
950	99.84679	97.01772	95.32127	94.31525
949	99.89756	96.99267	95.3077	94.32403
948	99.93806	96.96728	95.2935	94.33179
947	99.96838	96.94155	95.27868	94.33854
946	99.98865	96.91546	95.26324	94.34427
945	99.99908	96.88903	95.24717	94.34897
944	100	96.86224	95.23047	94.35264
943	99.99183	96.83509	95.21313	94.35526
942	99.97514	96.80759	95.19514	94.35683
941	99.95068	96.77973	95.17652	94.35734
940	99.91935	96.7515	95.15724	94.35679
939	99.88227	96.7229	95.13731	94.35517
938	99.84071	96.69394	95.11673	94.35247
937	99.79607	96.6646	95.09548	94.34869
936	99.7498	96.63489	95.07357	94.34382
935	99.70334	96.6048	95.051	94.33786
934	99.65797	96.57434	95.02776	94.33079
933	99.61479	96.54349	95.00384	94.32262
932	99.57467	96.51226	94.97925	94.31334
931	99.53822	96.48064	94.95398	94.30294
930	99.50582	96.44864	94.92802	94.29141

929	99.47765	96.41624	94.90139	94.27876
928	99.45373	96.38345	94.87406	94.26498
927	99.43391	96.35027	94.84604	94.25007
926	99.41801	96.31668	94.81733	94.23401
925	99.40577	96.2827	94.78792	94.2168
924	99.39695	96.24831	94.75782	94.19845
923	99.39134	96.21352	94.72701	94.17894
922	99.38877	96.17832	94.6955	94.15828
921	99.38911	96.14271	94.66328	94.13646
920	99.39227	96.10668	94.63036	94.11347
919	99.39815	96.07025	94.59672	94.08931
918	99.40662	96.03339	94.56237	94.06399
917	99.41752	95.99612	94.52731	94.03749
916	99.43067	95.95842	94.49153	94.00981
915	99.44583	95.92031	94.45503	93.98096
914	99.46273	95.88176	94.41781	93.95092
913	99.48109	95.84279	94.37987	93.9197
912	99.5006	95.80339	94.34121	93.8873
911	99.52094	95.76356	94.30181	93.85371
910	99.54179	95.72329	94.2617	93.81893
909	99.56288	95.68259	94.22085	93.78296
908	99.58399	95.64144	94.17928	93.7458
907	99.60499	95.59986	94.13697	93.70744
906	99.62583	95.55783	94.09393	93.66789
905	99.64657	95.51536	94.05016	93.62714
904	99.66727	95.47244	94.00565	93.5852
903	99.688	95.42908	93.96041	93.54206
902	99.70876	95.38526	93.91443	93.49773
901	99.72945	95.34099	93.86771	93.45219
900	99.74984	95.29626	93.82026	93.40546
899	99.76955	95.25108	93.77207	93.35754
898	99.78803	95.20544	93.72313	93.30841
897	99.80454	95.15934	93.67346	93.25809
896	99.81818	95.11277	93.62305	93.20657
895	99.82783	95.06575	93.5719	93.15386
894	99.83223	95.01825	93.52001	93.09996
893	99.82998	94.97029	93.46737	93.04486
892	99.81962	94.92186	93.414	92.98857
891	99.79971	94.87295	93.35988	92.93109
890	99.76888	94.82357	93.30503	92.87242
889	99.72591	94.77372	93.24943	92.81256

888	99.66976	94.72339	93.19309	92.75152
887	99.60056	94.67258	93.13601	92.68929
886	99.51549	94.62129	93.07819	92.62589
885	99.41474	94.56952	93.01963	92.56131
884	99.29813	94.51726	92.96033	92.49555
883	99.1657	94.46452	92.90029	92.42861
882	99.01763	94.41129	92.83952	92.36051
881	98.85424	94.35758	92.778	92.29124
880	98.67591	94.30337	92.71575	92.2208
879	98.48304	94.24867	92.65277	92.14921
878	98.27607	94.19348	92.58904	92.07645
877	98.05544	94.13779	92.52459	92.00255
876	97.82163	94.08161	92.4594	91.92749
875	97.57522	94.02493	92.39348	91.85129
874	97.31685	93.96775	92.32683	91.77394
873	97.04728	93.91007	92.25945	91.69546
872	96.76735	93.85189	92.19134	91.61584
871	96.47798	93.7932	92.1225	91.53509
870	96.18012	93.73401	92.05294	91.45322
869	95.87476	93.67432	91.98265	91.37023
868	95.56291	93.61412	91.91165	91.28612
867	95.24558	93.5534	91.83992	91.20091
866	94.9238	93.49218	91.76747	91.11459
865	94.59862	93.43045	91.69431	91.02717
864	94.27106	93.3682	91.62043	90.93865
863	93.94214	93.30545	91.54584	90.84905
862	93.61287	93.24217	91.47054	90.75836
861	93.28424	93.17838	91.39453	90.6666
860	92.95729	93.11408	91.31781	90.57376
859	92.63309	93.04926	91.24039	90.47986
858	92.31276	92.98391	91.16226	90.3849
857	91.99751	92.91805	91.08344	90.28889
856	91.68861	92.85167	91.00391	90.19183
855	91.38741	92.78476	90.92369	90.09373
854	91.09529	92.71733	90.84278	89.99459
853	90.81374	92.64938	90.76118	89.89443
852	90.54426	92.5809	90.67889	89.79325
851	90.28841	92.5119	90.59592	89.69106
850	90.04777	92.44237	90.51226	89.58786
849	89.82393	92.37231	90.42792	89.48366
848	89.61842	92.30173	90.34291	89.37847

847	89.43268	92.23061	90.25723	89.27229
846	89.26801	92.15897	90.17087	89.16514
845	89.12551	92.0868	90.08384	89.05702
844	89.0061	92.01409	89.99615	88.94794
843	88.91047	91.94085	89.9078	88.8379
842	88.83905	91.86708	89.81879	88.72692
841	88.79204	91.79278	89.72913	88.61499
840	88.76942	91.71794	89.63881	88.50214
839	88.77095	91.64257	89.54784	88.38836
838	88.79623	91.56666	89.45623	88.27367
837	88.84474	91.49022	89.36398	88.15808
836	88.91592	91.41324	89.27108	88.04158
835	89.00917	91.33573	89.17756	87.9242
834	89.12389	91.25768	89.0834	87.80593
833	89.25949	91.17909	88.98861	87.68679
832	89.41535	91.09997	88.8932	87.56679
831	89.59082	91.0203	88.79717	87.44593
830	89.78516	90.9401	88.70052	87.32423
829	89.99753	90.85936	88.60326	87.20169
828	90.22703	90.77808	88.50538	87.07831
827	90.47267	90.69626	88.40691	86.95412
826	90.73338	90.6139	88.30783	86.82911
825	91.00807	90.531	88.20815	86.70331
824	91.29559	90.44756	88.10788	86.57671
823	91.59476	90.36358	88.00702	86.44932
822	91.90439	90.27906	87.90557	86.32116
821	92.2233	90.19401	87.80354	86.19223
820	92.55028	90.10841	87.70093	86.06254
819	92.88419	90.02227	87.59775	85.93211
818	93.22384	89.93559	87.494	85.80094
817	93.56811	89.84837	87.38969	85.66903
816	93.91579	89.7606	87.28481	85.53641
815	94.26567	89.6723	87.17938	85.40308
814	94.61649	89.58346	87.07339	85.26904
813	94.96689	89.49408	86.96685	85.13431
812	95.3155	89.40416	86.85977	84.9989
811	95.66087	89.3137	86.75215	84.86282
810	96.00151	89.22269	86.644	84.72607
809	96.3359	89.13115	86.53531	84.58867
808	96.66247	89.03907	86.42609	84.45062
807	96.97962	88.94645	86.31636	84.31194

806	97.28571	88.8533	86.2061	84.17263
805	97.57909	88.7596	86.09533	84.03271
804	97.85815	88.66537	85.98405	83.89218
803	98.12131	88.57059	85.87227	83.75105
802	98.36708	88.47529	85.75999	83.60933
801	98.59409	88.37944	85.64721	83.46704
800	98.80112	88.28306	85.53394	83.32417
799	98.9871	88.18614	85.42018	83.18075
798	99.15113	88.08869	85.30594	83.03677
797	99.2925	87.9907	85.19123	82.89226
796	99.41069	87.89218	85.07604	82.74721
795	99.50533	87.79312	84.96038	82.60165
794	99.57619	87.69353	84.84426	82.45557
793	99.62313	87.59341	84.72768	82.30898
792	99.62976	87.49276	84.61064	82.16191
791	99.6123	87.39158	84.49316	82.01435
790	99.57066	87.28986	84.37523	81.86632
789	99.50476	87.18762	84.25685	81.71782
788	99.41452	87.08485	84.13805	81.56886
787	99.29987	86.98155	84.01881	81.41946
786	99.16079	86.87772	83.89914	81.26962
785	98.9973	86.77337	83.77905	81.11935
784	98.80946	86.66849	83.65854	80.96867
783	98.59737	86.56308	83.53762	80.81757
782	98.3612	86.45716	83.41629	80.66607
781	98.1012	86.35071	83.29456	80.51418
780	97.8177	86.24373	83.17243	80.36191
779	97.51113	86.13624	83.0499	80.20927
778	97.18205	86.02823	82.92699	80.05625
777	96.83115	85.9197	82.80369	79.90289
776	96.45921	85.81065	82.68	79.74917
775	96.06712	85.70108	82.55594	79.59512
774	95.65586	85.591	82.43151	79.44074
773	95.22652	85.48041	82.30672	79.28603
772	94.7803	85.3693	82.18155	79.13101
771	94.31849	85.25768	82.05604	78.97569
770	93.84251	85.14555	81.93016	78.82008
769	93.35382	85.03291	81.80394	78.66417
768	92.85397	84.91977	81.67738	78.50799
767	92.34453	84.80611	81.55047	78.35154
766	91.82704	84.69195	81.42323	78.19482

765	91.30303	84.57729	81.29566	78.03785
764	90.77396	84.46212	81.16776	77.88063
763	90.24128	84.34645	81.03954	77.72318
762	89.70634	84.23028	80.911	77.5655
761	89.17049	84.11362	80.78215	77.40759
760	88.63501	83.99645	80.65298	77.24947
759	88.10112	83.87879	80.52352	77.09114
758	87.57002	83.76064	80.39375	76.93261
757	87.04286	83.642	80.26369	76.77389
756	86.52073	83.52286	80.13333	76.61499
755	86.0047	83.40323	80.00269	76.45591
754	85.49578	83.28312	79.87176	76.29666
753	84.9949	83.16252	79.74056	76.13725
752	84.50294	83.04143	79.60908	75.97768
751	84.02074	82.91986	79.47733	75.81797
750	83.54906	82.79781	79.34531	75.65812
749	83.08864	82.67529	79.21303	75.49813
748	82.64023	82.55228	79.08049	75.33801
747	82.20454	82.4288	78.9477	75.17777
746	81.78231	82.30484	78.81465	75.01742
745	81.37426	82.18041	78.68136	74.85697
744	80.98108	82.05551	78.54783	74.69641
743	80.60341	81.93014	78.41406	74.53576
742	80.24188	81.8043	78.28005	74.37501
741	79.89705	81.678	78.14581	74.21419
740	79.5695	81.55124	78.01135	74.05329
739	79.25974	81.42401	77.87666	73.89232
738	78.96829	81.29633	77.74176	73.73129
737	78.6956	81.16819	77.60663	73.5702
736	78.44204	81.03959	77.4713	73.40906
735	78.20783	80.91054	77.33576	73.24787
734	77.99306	80.78103	77.20001	73.08664
733	77.79757	80.65108	77.06406	72.92537
732	77.62101	80.52068	76.92791	72.76407
731	77.46278	80.38984	76.79157	72.60275
730	77.32207	80.25855	76.65504	72.44141
729	77.19785	80.12682	76.51832	72.28005
728	77.08889	79.99466	76.38142	72.11868
727	76.99379	79.86205	76.24434	71.95731
726	76.91102	79.72901	76.10708	71.79594
725	76.83894	79.59554	75.96964	71.63457

724	76.77587	79.46164	75.83204	71.4732
723	76.72009	79.32731	75.69427	71.31186
722	76.66992	79.19256	75.55633	71.15053
721	76.62372	79.05738	75.41824	70.98922
720	76.57994	78.92178	75.27998	70.82793
719	76.5371	78.78576	75.14157	70.66668
718	76.49379	78.64933	75.00301	70.50546
717	76.44867	78.51248	74.8643	70.34428
716	76.40049	78.37522	74.72545	70.18314
715	76.34799	78.23755	74.58645	70.02204
714	76.28993	78.09947	74.44731	69.861
713	76.22506	77.96099	74.30803	69.7
712	76.15213	77.8221	74.16862	69.53906
711	76.06992	77.68282	74.02908	69.37818
710	75.97728	77.54314	73.88941	69.21736
709	75.87313	77.40306	73.74961	69.05661
708	75.75653	77.26259	73.60969	68.89592
707	75.62655	77.12173	73.46964	68.73531
706	75.48227	76.98049	73.32948	68.57476
705	75.32274	76.83886	73.1892	68.4143
704	75.14693	76.69684	73.0488	68.25391
703	74.95377	76.55445	72.9083	68.0936
702	74.74213	76.41168	72.76768	67.93338
701	74.51092	76.26853	72.62696	67.77324
700	74.2591	76.12501	72.48614	67.6132
699	73.98575	75.98113	72.34521	67.45324
698	73.69008	75.83687	72.20418	67.29337
697	73.37144	75.69226	72.06305	67.1336
696	73.02928	75.54728	71.92182	66.97392
695	72.66319	75.40194	71.7805	66.81434
694	72.2729	75.25624	71.63909	66.65486
693	71.85828	75.11019	71.49759	66.49548
692	71.41938	74.96379	71.356	66.33621
691	70.95647	74.81705	71.21432	66.17704
690	70.47005	74.66995	71.07256	66.01797
689	69.96087	74.52252	70.93072	65.85901
688	69.42992	74.37474	70.78879	65.70015
687	68.87841	74.22663	70.64678	65.54141
686	68.30777	74.07818	70.5047	65.38277
685	67.71961	73.9294	70.36254	65.22424
684	67.11562	73.78029	70.22031	65.06583

683	66.49763	73.63086	70.078	64.90753
682	65.86745	73.4811	69.93562	64.74933
681	65.22689	73.33102	69.79317	64.59126
680	64.57776	73.18063	69.65065	64.43329
679	63.9218	73.02992	69.50806	64.27544
678	63.26069	72.8789	69.36541	64.1177
677	62.59606	72.72757	69.22269	63.96008
676	61.92946	72.57593	69.07991	63.80257
675	61.26236	72.42399	68.93706	63.64518
674	60.59613	72.27175	68.79415	63.4879
673	59.93205	72.11921	68.65119	63.33074
672	59.27134	71.96638	68.50816	63.17369
671	58.61515	71.81326	68.36508	63.01675
670	57.96464	71.65985	68.22193	62.85993
669	57.32097	71.50615	68.07874	62.70323
668	56.68533	71.35217	67.93548	62.54663
667	56.05886	71.19791	67.79217	62.39015
666	55.44264	71.04337	67.64881	62.23378
665	54.83761	70.88856	67.50539	62.07753
664	54.24457	70.73348	67.36193	61.92138
663	53.66429	70.57813	67.21841	61.76535
662	53.0975	70.42252	67.07484	61.60943
661	52.54499	70.26664	66.93122	61.45361
660	52.00761	70.1105	66.78755	61.2979
659	51.48628	69.95411	66.64383	61.14231
658	50.98197	69.79747	66.50006	60.98681
657	50.49565	69.64058	66.35625	60.83143
656	50.02824	69.48344	66.21239	60.67614
655	49.58054	69.32605	66.06848	60.52097
654	49.15326	69.16843	65.92452	60.36589
653	48.74701	69.01057	65.78052	60.21091
652	48.36225	68.85247	65.63647	60.05604
651	47.99938	68.69414	65.49238	59.90126
650	47.65865	68.53559	65.34824	59.74658
649	47.3402	68.37681	65.20406	59.592
648	47.04402	68.2178	65.05983	59.43751
647	46.76996	68.05858	64.91556	59.28311
646	46.51769	67.89914	64.77124	59.12881
645	46.28677	67.73949	64.62688	58.9746
644	46.07663	67.57963	64.48248	58.82047
643	45.88658	67.41956	64.33803	58.66643

642	45.71581	67.25929	64.19354	58.51248
641	45.56347	67.09882	64.04901	58.35861
640	45.4286	66.93815	63.90443	58.20483
639	45.31021	66.77728	63.75981	58.05112
638	45.20729	66.61623	63.61514	57.8975
637	45.1188	66.45499	63.47043	57.74395
636	45.04371	66.29356	63.32568	57.59048
635	44.98097	66.13195	63.18088	57.43708
634	44.92952	65.97016	63.03604	57.28375
633	44.88828	65.8082	62.89116	57.1305
632	44.85613	65.64606	62.74623	56.97731
631	44.83196	65.48375	62.60125	56.82419
630	44.81462	65.32128	62.45624	56.67113
629	44.80299	65.15865	62.31117	56.51814
628	44.79594	64.99585	62.16607	56.36521
627	44.79236	64.8329	62.02091	56.21234
626	44.79113	64.6698	61.87572	56.05952
625	44.79111	64.50654	61.73047	55.90676
624	44.79112	64.34314	61.58518	55.75406
623	44.79	64.1796	61.43985	55.60141
622	44.78659	64.01591	61.29446	55.4488
621	44.77982	63.85209	61.14903	55.29625
620	44.76866	63.68813	61.00356	55.14374
619	44.75219	63.52404	60.85803	54.99128
618	44.72954	63.35982	60.71246	54.83886
617	44.69987	63.19547	60.56684	54.68648
616	44.6624	63.03101	60.42117	54.53414
615	44.61634	62.86642	60.27546	54.38183
614	44.56096	62.70172	60.12969	54.22957
613	44.49557	62.53691	59.98387	54.07733
612	44.41954	62.37199	59.83801	53.92513
611	44.33235	62.20696	59.69209	53.77295
610	44.23359	62.04183	59.54612	53.62081
609	44.12295	61.87659	59.4001	53.46869
608	44.00023	61.71127	59.25403	53.3166
607	43.86532	61.54584	59.10791	53.16452
606	43.71823	61.38033	58.96173	53.01247
605	43.55907	61.21473	58.8155	52.86044
604	43.38805	61.04905	58.66922	52.70843
603	43.20549	60.88328	58.52288	52.55643
602	43.01177	60.71744	58.37649	52.40445

601	42.80736	60.55152	58.23005	52.25248
600	42.59276	60.38553	58.08355	52.10052
599	42.3685	60.21947	57.93699	51.94857
598	42.13517	60.05335	57.79038	51.79663
597	41.89338	59.88717	57.64372	51.64469
596	41.6438	59.72092	57.49699	51.49276
595	41.38718	59.55462	57.35021	51.34083
594	41.1243	59.38827	57.20337	51.18891
593	40.85598	59.22186	57.05648	51.03699
592	40.5831	59.05541	56.90952	50.88506
591	40.30653	58.88892	56.76251	50.73314
590	40.02719	58.72239	56.61544	50.58121
589	39.74602	58.55581	56.46831	50.42928
588	39.464	58.38921	56.32112	50.27734
587	39.18214	58.22257	56.17387	50.12539
586	38.90149	58.05591	56.02656	49.97344
585	38.62313	57.88921	55.87919	49.82148
584	38.34813	57.7225	55.73176	49.6695
583	38.07756	57.55577	55.58427	49.51752
582	37.81246	57.38902	55.43671	49.36553
581	37.55383	57.22227	55.2891	49.21352
580	37.30266	57.0555	55.14142	49.0615
579	37.05985	56.88872	54.99369	48.90946
578	36.82628	56.72194	54.84589	48.75741
577	36.60271	56.55516	54.69803	48.60534
576	36.38981	56.38838	54.5501	48.45326
575	36.18813	56.22161	54.40212	48.30115
574	35.99809	56.05485	54.25407	48.14903
573	35.82	55.8881	54.10595	47.99689
572	35.65407	55.72136	53.95778	47.84474
571	35.50041	55.55464	53.80954	47.69256
570	35.35907	55.38794	53.66124	47.54036
569	35.23002	55.22127	53.51288	47.38814
568	35.11317	55.05462	53.36446	47.2359
567	35.00837	54.888	53.21597	47.08364
566	34.91542	54.72141	53.06742	46.93136
565	34.83405	54.55486	52.91881	46.77906
564	34.76394	54.38835	52.77013	46.62674
563	34.70474	54.22188	52.62139	46.47439
562	34.65602	54.05546	52.47259	46.32202
561	34.61733	53.88908	52.32373	46.16963

560	34.58818	53.72275	52.17481	46.01723
559	34.56807	53.55648	52.02582	45.86479
558	34.55651	53.39026	51.87678	45.71234
557	34.55303	53.2241	51.72767	45.55987
556	34.55725	53.058	51.57851	45.40738
555	34.56878	52.89197	51.42928	45.25487
554	34.58731	52.72601	51.27999	45.10233
553	34.61253	52.56012	51.13065	44.94978
552	34.6441	52.3943	50.98125	44.79721
551	34.68166	52.22856	50.83178	44.64463
550	34.72482	52.06289	50.68226	44.49202
549	34.77313	51.89731	50.53269	44.3394
548	34.82615	51.73182	50.38305	44.18677
547	34.88342	51.56641	50.23336	44.03411
546	34.9445	51.4011	50.08362	43.88145
545	35.00892	51.23588	49.93382	43.72877
544	35.07623	51.07075	49.78397	43.57608
543	35.14594	50.90573	49.63406	43.42338
542	35.21753	50.7408	49.4841	43.27067
541	35.29044	50.57599	49.33409	43.11796
540	35.36408	50.41128	49.18403	42.96523
539	35.43786	50.24668	49.03392	42.8125
538	35.51116	50.08219	48.88377	42.65976
537	35.58336	49.91782	48.73356	42.50702
536	35.65385	49.75357	48.58331	42.35428
535	35.72203	49.58945	48.43301	42.20154
534	35.78736	49.42544	48.28267	42.0488
533	35.84929	49.26157	48.13228	41.89607
532	35.90738	49.09782	47.98185	41.74334
531	35.96122	48.93421	47.83139	41.59061
530	36.0105	48.77073	47.68088	41.4379
529	36.05494	48.60739	47.53033	41.28519
528	36.09432	48.44419	47.37975	41.1325
527	36.12846	48.28114	47.22913	40.97982
526	36.15719	48.11823	47.07847	40.82716
525	36.18036	47.95547	46.92779	40.67451
524	36.19786	47.79286	46.77707	40.52189
523	36.20957	47.63041	46.62632	40.36929
522	36.21543	47.46812	46.47554	40.21671
521	36.21535	47.30598	46.32474	40.06416
520	36.20929	47.14401	46.17391	39.91164

519	36.19723	46.9822	46.02306	39.75915
518	36.17919	46.82056	45.87219	39.6067
517	36.15524	46.65909	45.7213	39.45429
516	36.1255	46.49779	45.57039	39.30191
515	36.09019	46.33666	45.41946	39.14958
514	36.04955	46.17572	45.26852	38.99729
513	36.00382	46.01495	45.11757	38.84505
512	35.95327	45.85437	44.96661	38.69286
511	35.89812	45.69398	44.81563	38.54072
510	35.83859	45.53377	44.66466	38.38864
509	35.77488	45.37375	44.51368	38.23662
508	35.70719	45.21393	44.36269	38.08466
507	35.63576	45.0543	44.21171	37.93276
506	35.56086	44.89487	44.06073	37.78093
505	35.4828	44.73564	43.90975	37.62918
504	35.40187	44.57662	43.75878	37.4775
503	35.31838	44.4178	43.60782	37.32589
502	35.2326	44.25918	43.45687	37.17437
501	35.14478	44.10078	43.30594	37.02293
500	35.05512	43.94259	43.15502	36.87158
499	34.96382	43.78462	43.00412	36.72032
498	34.87107	43.62687	42.85325	36.56916
497	34.77701	43.46933	42.70239	36.41809
496	34.6818	43.31202	42.55157	36.26712
495	34.58558	43.15493	42.40077	36.11626
494	34.48847	42.99807	42.25001	35.96551
493	34.3906	42.84145	42.09928	35.81486
492	34.29211	42.68505	41.94859	35.66434
491	34.19315	42.52889	41.79794	35.51393
490	34.0939	42.37296	41.64733	35.36365
489	33.99456	42.21727	41.49677	35.21349
488	33.89534	42.06183	41.34626	35.06347
487	33.79643	41.90663	41.1958	34.91357
486	33.69804	41.75168	41.04539	34.76382
485	33.60037	41.59697	40.89505	34.61421
484	33.50362	41.44252	40.74476	34.46474
483	33.40798	41.28831	40.59454	34.31543
482	33.31369	41.13437	40.44439	34.16627
481	33.22096	40.98068	40.29431	34.01727
480	33.13006	40.82725	40.1443	33.86843
479	33.04129	40.67408	39.99437	33.71975

478	32.95498	40.52118	39.84452	33.57125
477	32.8715	40.36855	39.69475	33.42292
476	32.79123	40.21618	39.54508	33.27477
475	32.7146	40.06408	39.39549	33.1268
474	32.64195	39.91226	39.24599	32.97902
473	32.57361	39.76071	39.0966	32.83143
472	32.50976	39.60944	38.9473	32.68403
471	32.4505	39.45845	38.79811	32.53684
470	32.3958	39.30774	38.64903	32.38985
469	32.34553	39.15732	38.50006	32.24307
468	32.29952	39.00718	38.35121	32.0965
467	32.25756	38.85733	38.20247	31.95015
466	32.21946	38.70777	38.05386	31.80402
465	32.18507	38.5585	37.90537	31.65811
464	32.15425	38.40952	37.75702	31.51244
463	32.12695	38.26085	37.6088	31.367
462	32.10314	38.11247	37.46071	31.2218
461	32.08285	37.96439	37.31277	31.07684
460	32.06613	37.81661	37.16497	30.93213
459	32.05303	37.66914	37.01733	30.78768
458	32.04359	37.52197	36.86983	30.64348
457	32.0378	37.37512	36.7225	30.49954
456	32.03564	37.22857	36.57532	30.35587
455	32.03701	37.08234	36.42831	30.21246
454	32.04183	36.93642	36.28147	30.06934
453	32.05	36.79082	36.1348	29.92649
452	32.06142	36.64553	35.98831	29.78393
451	32.07609	36.50057	35.842	29.64165
450	32.09403	36.35593	35.69588	29.49967
449	32.11527	36.21161	35.54995	29.35799
448	32.1398	36.06762	35.4042	29.21661
447	32.16763	35.92396	35.25866	29.07553
446	32.19871	35.78062	35.11332	28.93477
445	32.23294	35.63762	34.96819	28.79432
444	32.27007	35.49495	34.82326	28.65419
443	32.3099	35.35262	34.67855	28.51439
442	32.35227	35.21063	34.53406	28.37492
441	32.39704	35.06897	34.3898	28.23578
440	32.44398	34.92766	34.24576	28.09698
439	32.49268	34.78668	34.10195	27.95852
438	32.54276	34.64605	33.95838	27.82041

437	32.59408	34.50577	33.81504	27.68265
436	32.64671	34.36584	33.67196	27.54525
435	32.70069	34.22625	33.52912	27.40821
434	32.75584	34.08702	33.38653	27.27153
433	32.81181	33.94814	33.24421	27.13523
432	32.86825	33.80962	33.10214	26.9993
431	32.92481	33.67145	32.96034	26.86374
430	32.98113	33.53364	32.81882	26.72857
429	33.03686	33.39619	32.67756	26.59379
428	33.09174	33.25911	32.53659	26.4594
427	33.14566	33.12238	32.3959	26.32541
426	33.19859	32.98602	32.2555	26.19182
425	33.25055	32.85003	32.11539	26.05864
424	33.3016	32.71441	31.97558	25.92586
423	33.35182	32.57915	31.83607	25.7935
422	33.40126	32.44427	31.69687	25.66155
421	33.44994	32.30976	31.55798	25.53003
420	33.49783	32.17563	31.4194	25.39894
419	33.54486	32.04187	31.28114	25.26827
418	33.59088	31.90849	31.14321	25.13804
417	33.63564	31.77549	31.0056	25.00826
416	33.67881	31.64287	30.86833	24.87891
415	33.72002	31.51063	30.7314	24.75001
414	33.75887	31.37878	30.5948	24.62157
413	33.79506	31.24731	30.45855	24.49358
412	33.82834	31.11623	30.32265	24.36605
411	33.8586	30.98554	30.18711	24.23899
410	33.88579	30.85524	30.05192	24.11239
409	33.90992	30.72533	29.9171	23.98627
408	33.931	30.59581	29.78265	23.86062
407	33.94891	30.46669	29.64857	23.73546
406	33.96343	30.33796	29.51486	23.61077
405	33.97427	30.20963	29.38154	23.48658
404	33.98109	30.0817	29.2486	23.36288
403	33.98353	29.95417	29.11605	23.23967
402	33.98116	29.82704	28.9839	23.11696
401	33.97375	29.70031	28.85214	22.99476
400	33.96132	29.57398	28.72079	22.87306
399	33.944	29.44806	28.58984	22.75188
398	33.92195	29.32255	28.4593	22.63121
397	33.89547	29.19745	28.32918	22.51105

396	33.86528	29.07275	28.19948	22.39142
395	33.83249	28.94847	28.0702	22.27232
394	33.79814	28.8246	27.94135	22.15374
393	33.76271	28.70114	27.81293	22.03569
392	33.72608	28.5781	27.68495	21.91818
391	33.68805	28.45547	27.55741	21.80121
390	33.64873	28.33325	27.43031	21.68479
389	33.60848	28.21146	27.30366	21.5689
388	33.56762	28.09009	27.17747	21.45357
387	33.52647	27.96913	27.05173	21.33879
386	33.48547	27.8486	26.92645	21.22457
385	33.44517	27.72849	26.80163	21.1109
384	33.40603	27.6088	26.67729	20.9978
383	33.36841	27.48954	26.55342	20.88526
382	33.33262	27.37071	26.43002	20.77328
381	33.29897	27.2523	26.3071	20.66188
380	33.2677	27.13432	26.18467	20.55106
379	33.23906	27.01677	26.06272	20.44081
378	33.2133	26.89965	25.94127	20.33114
377	33.19069	26.78297	25.82031	20.22205
376	33.17149	26.66671	25.69985	20.11355
375	33.15597	26.55089	25.5799	20.00564
374	33.1444	26.43551	25.46045	19.89832
373	33.13711	26.32056	25.34151	19.79159
372	33.13454	26.20605	25.22309	19.68545
371	33.13722	26.09197	25.10518	19.57992
370	33.14572	25.97834	24.98779	19.47499
369	33.16057	25.86514	24.87093	19.37066
368	33.18215	25.75238	24.7546	19.26693
367	33.21065	25.64007	24.63879	19.16382
366	33.24613	25.5282	24.52353	19.06131
365	33.28857	25.41677	24.4088	18.95942
364	33.33796	25.30579	24.29461	18.85814
363	33.39442	25.19525	24.18096	18.75748
362	33.4582	25.08516	24.06787	18.65744
361	33.52973	24.97552	23.95532	18.55802
360	33.60952	24.86632	23.84333	18.45923
359	33.69822	24.75757	23.7319	18.36106
358	33.79649	24.64927	23.62102	18.26352
357	33.90489	24.54143	23.51071	18.1666
356	34.02374	24.43403	23.40097	18.07032

355	34.1531	24.32709	23.2918	17.97467
354	34.29285	24.22059	23.18319	17.87966
353	34.4425	24.11456	23.07517	17.78528
352	34.60119	24.00897	22.96772	17.69154
351	34.76777	23.90384	22.86085	17.59844
350	34.9411	23.79917	22.75457	17.50598
349	35.12043	23.69495	22.64887	17.41416
348	35.30546	23.59119	22.54376	17.32298
347	35.49601	23.48789	22.43924	17.23245
346	35.69176	23.38505	22.33531	17.14257
345	35.89221	23.28266	22.23198	17.05334
344	36.09678	23.18074	22.12925	16.96475
343	36.30481	23.07927	22.02712	16.87681
342	36.51559	22.97827	21.9256	16.78953
341	36.72835	22.87773	21.82468	16.70289
340	36.94246	22.77765	21.72436	16.61691
339	37.15746	22.67803	21.62466	16.53158
338	37.37299	22.57888	21.52557	16.44691
337	37.58882	22.48019	21.4271	16.36289
336	37.80482	22.38196	21.32924	16.27953
335	38.02091	22.2842	21.232	16.19683
334	38.23699	22.18691	21.13537	16.11478
333	38.45289	22.09008	21.03938	16.0334
332	38.66835	21.99371	20.944	15.95267
331	38.88304	21.89782	20.84925	15.8726
330	39.09657	21.80239	20.75513	15.79319
329	39.30844	21.70743	20.66164	15.71444
328	39.51802	21.61293	20.56877	15.63635
327	39.72457	21.51891	20.47654	15.55892
326	39.92733	21.42535	20.38495	15.48215
325	40.12567	21.33227	20.29398	15.40604
324	40.31906	21.23965	20.20366	15.33059
323	40.50685	21.1475	20.11397	15.25581
322	40.68858	21.05583	20.02492	15.18168
321	40.86427	20.96462	19.93652	15.10822
320	41.03407	20.87389	19.84875	15.03542
319	41.19805	20.78362	19.76163	14.96328
318	41.35612	20.69383	19.67515	14.8918
317	41.50811	20.60451	19.58931	14.82098
316	41.65372	20.51566	19.50412	14.75082
315	41.79261	20.42728	19.41958	14.68131

314	41.92443	20.33938	19.33568	14.61247
313	42.04886	20.25195	19.25243	14.54429
312	42.16576	20.16499	19.16983	14.47676
311	42.27514	20.0785	19.08788	14.4099
310	42.37723	19.99249	19.00659	14.34368
309	42.47245	19.90695	18.92594	14.27813
308	42.56134	19.82189	18.84594	14.21323
307	42.64451	19.7373	18.76659	14.14898
306	42.7225	19.65318	18.6879	14.08539
305	42.79565	19.56953	18.60985	14.02245
304	42.86414	19.48636	18.53246	13.96017
303	42.928	19.40367	18.45572	13.89853
302	42.9873	19.32144	18.37964	13.83754
301	43.0423	19.2397	18.30421	13.77721
300	43.09354	19.15842	18.22943	13.71752
299	43.14173	19.07762	18.1553	13.65847
298	43.18754	18.9973	18.08183	13.60007
297	43.23153	18.91744	18.00901	13.54231
296	43.27399	18.83806	17.93684	13.4852
295	43.31503	18.75916	17.86532	13.42873
294	43.35456	18.68073	17.79445	13.37289
293	43.3924	18.60277	17.72424	13.3177
292	43.42827	18.52529	17.65468	13.26313
291	43.46188	18.44828	17.58577	13.20921
290	43.49299	18.37174	17.5175	13.15591
289	43.52149	18.29568	17.44989	13.10325
288	43.54737	18.22009	17.38293	13.05122
287	43.57077	18.14497	17.31661	12.99981
286	43.59186	18.07033	17.25094	12.94903
285	43.61086	17.99616	17.18592	12.89888
284	43.62805	17.92246	17.12154	12.84934
283	43.6437	17.84923	17.05781	12.80042
282	43.65803	17.77647	16.99472	12.75213
281	43.67111	17.70419	16.93227	12.70444
280	43.68321	17.63238	16.87047	12.65737

Fig 3 data:

2tata	intensity	intensity	intensity	intensity
(deg.)	(a.u)	(a.u)	(a.u)	(a.u)
20.024	4091.44735	1218	2067	1198
20.057	4096.4072	1269	1987	1206
20.09	4101.17414	1254	2074	1198
20.123	4105.75022	1262	2027	1225
20.156	4110.13746	1254	2066	1234
20.189	4114.33788	1350	2106	1299
20.222	4118.35347	1323	2056	1260
20.255	4122.18622	1281	2025	1252
20.288	4125.8381	1322	1987	1262
20.321	4129.31109	1335	2055	1278
20.354	4132.60712	1370	2058	1226
20.387	4135.72814	1335	2094	1277
20.42	4138.67607	1363	2063	1284
20.453	4141.45282	1312	2067	1242
20.486	4144.0603	1309	1949	1217
20.519	4146.5004	1381	2042	1260
20.552	4148.775	1296	2028	1247
20.585	4150.88595	1332	1981	1246
20.618	4152.83511	1352	2031	1274
20.651	4154.62433	1323	2086	1216
20.684	4156.25543	1297	2109	1237
20.717	4157.73023	1359	2042	1335
20.75	4159.05054	1361	2103	1318
20.783	4160.21815	1362	2110	1249
20.816	4161.23484	1323	2044	1279
20.849	4162.10238	1349	2003	1215
20.882	4162.82254	1395	2087	1223
20.915	4163.39705	1386	2059	1281
20.948	4163.82765	1323	2050	1318
20.981	4164.11608	1386	2046	1282
21.014	4164.26403	1433	2045	1280
21.047	4164.27321	1343	2129	1268
21.08	4164.14532	1332	2120	1299
21.113	4163.88202	1391	2104	1304
21.146	4163.48498	1377	2150	1301
21.179	4162.95586	1428	2130	1360
21.212	4162.29631	1460	2125	1337
21.245	4161.50794	1421	2154	1335

21.278	4160.5924	1438	2128	1377
21.311	4159.55128	1471	2108	1325
21.344	4158.38619	1454	2071	1307
21.377	4157.09871	1416	2118	1329
21.41	4155.69041	1444	2163	1327
21.443	4154.16288	1489	2112	1344
21.476	4152.51765	1466	2124	1343
21.509	4150.75627	1450	2164	1355
21.542	4148.88028	1459	2171	1407
21.575	4146.89119	1469	2153	1418
21.608	4144.79052	1461	2186	1376
21.641	4142.57977	1464	2222	1355
21.674	4140.26042	1513	2241	1379
21.707	4137.83396	1530	2184	1378
21.74	4135.30185	1513	2180	1331
21.773	4132.66554	1490	2223	1377
21.806	4129.92649	1479	2167	1415
21.839	4127.08613	1516	2122	1374
21.872	4124.14589	1560	2191	1395
21.905	4121.10718	1535	2257	1429
21.938	4117.97139	1571	2225	1433
21.971	4114.73994	1542	2218	1438
22.004	4111.41419	1503	2276	1432
22.037	4107.99553	1469	2212	1358
22.07	4104.48531	1583	2228	1447
22.103	4100.88489	1613	2190	1416
22.136	4097.19561	1562	2230	1426
22.169	4093.41879	1592	2199	1474
22.202	4089.55577	1557	2278	1396
22.235	4085.60784	1552	2267	1450
22.268	4081.57632	1568	2301	1457
22.301	4077.46249	1581	2278	1403
22.334	4073.26764	1640	2253	1469
22.367	4068.99303	1574	2269	1467
22.4	4064.63992	1582	2343	1444
22.433	4060.20957	1574	2321	1473
22.466	4055.70322	1565	2256	1521
22.499	4051.12209	1619	2283	1494
22.532	4046.46741	1578	2274	1478
22.565	4041.74039	1535	2197	1496
22.598	4036.94223	1525	2314	1526

22.631	4032.07413	1682	2381	1390
22.664	4027.13726	1652	2292	1537
22.697	4022.13279	1671	2374	1427
22.73	4017.06189	1616	2270	1447
22.763	4011.92572	1707	2288	1604
22.796	4006.72542	1691	2387	1501
22.829	4001.46211	1576	2369	1572
22.862	3996.13693	1676	2294	1482
22.895	3990.75099	1617	2392	1508
22.928	3985.30539	1655	2362	1545
22.961	3979.80124	1580	2359	1550
22.994	3974.23962	1669	2364	1535
23.027	3968.62161	1646	2356	1519
23.06	3962.94827	1662	2390	1552
23.093	3957.22066	1637	2349	1520
23.126	3951.43985	1679	2375	1516
23.159	3945.60685	1734	2381	1528
23.192	3939.72272	1690	2437	1580
23.225	3933.78847	1681	2378	1627
23.258	3927.80512	1670	2481	1633
23.291	3921.77366	1706	2439	1618
23.324	3915.6951	1715	2333	1565
23.357	3909.57042	1700	2393	1597
23.39	3903.40061	1714	2442	1570
23.423	3897.18662	1747	2352	1615
23.456	3890.92943	1777	2381	1569
23.489	3884.62998	1721	2407	1585
23.522	3878.28921	1749	2341	1631
23.555	3871.90807	1797	2323	1608
23.588	3865.48747	1755	2439	1630
23.621	3859.02833	1745	2464	1576
23.654	3852.53156	1767	2355	1566
23.687	3845.99807	1798	2369	1626
23.72	3839.42873	1843	2403	1655
23.753	3832.82444	1818	2406	1636
23.786	3826.18607	1790	2469	1597
23.819	3819.51449	1825	2492	1595
23.852	3812.81055	1806	2502	1634
23.885	3806.0751	1818	2473	1662
23.918	3799.30899	1832	2422	1664
23.951	3792.51304	1764	2424	1666

23.984	3785.68808	1747	2467	1679
24.017	3778.83493	1803	2449	1660
24.05	3771.9544	1824	2422	1636
24.083	3765.04728	1771	2489	1679
24.116	3758.11437	1769	2457	1683
24.149	3751.15645	1807	2447	1627
24.182	3744.1743	1796	2501	1625
24.215	3737.16869	1789	2428	1663
24.248	3730.14037	1806	2425	1712
24.281	3723.09011	1854	2490	1692
24.314	3716.01864	1832	2498	1635
24.347	3708.92669	1838	2481	1672
24.38	3701.81501	1856	2501	1745
24.413	3694.68431	1857	2485	1702
24.446	3687.53531	1859	2525	1710
24.479	3680.3687	1854	2467	1675
24.512	3673.18519	1801	2457	1640
24.545	3665.98547	1857	2502	1734
24.578	3658.77023	1851	2517	1724
24.611	3651.54013	1889	2533	1685
24.644	3644.29585	1933	2537	1703
24.677	3637.03804	1894	2589	1720
24.71	3629.76737	1907	2586	1711
24.743	3622.48447	1870	2607	1715
24.776	3615.18999	1841	2557	1710
24.809	3607.88455	1812	2543	1674
24.842	3600.56879	1880	2571	1728
24.875	3593.24331	1832	2569	1691
24.908	3585.90873	1862	2513	1804
24.941	3578.56564	1871	2532	1740
24.974	3571.21466	1827	2496	1725
25.007	3563.85636	1968	2575	1735
25.04	3556.49132	1864	2626	1787
25.073	3549.12012	1959	2566	1771
25.106	3541.74333	1908	2603	1766
25.139	3534.36151	1982	2588	1800
25.172	3526.97521	1902	2629	1714
25.205	3519.58498	1985	2665	1711
25.238	3512.19136	1914	2753	1682
25.271	3504.79487	1880	2649	1789
25.304	3497.39606	1938	2675	1879

25.337	3489.99544	1939	2897	1865
25.37	3482.59351	2037	2884	1733
25.403	3475.1908	1926	2902	1850
25.436	3467.78779	2009	2768	1801
25.469	3460.38498	1932	2670	1855
25.502	3452.98287	1877	2649	1790
25.535	3445.58192	1926	2598	1723
25.568	3438.18261	1851	2497	1680
25.601	3430.78542	1901	2498	1680
25.634	3423.3908	1890	2424	1698
25.667	3415.9992	1863	2584	1626
25.7	3408.61108	1766	2599	1678
25.733	3401.22688	1887	2499	1745
25.766	3393.84704	1892	2532	1647
25.799	3386.47198	1920	2496	1713
25.832	3379.10213	1949	2556	1733
25.865	3371.7379	1896	2473	1771
25.898	3364.37971	1853	2448	1771
25.931	3357.02797	1870	2489	1745
25.964	3349.68307	1820	2459	1697
25.997	3342.34541	1833	2420	1728
26.03	3335.01538	1836	2417	1654
26.063	3327.69335	1829	2378	1709
26.096	3320.37971	1929	2411	1721
26.129	3313.07482	1839	2351	1706
26.162	3305.77905	1887	2332	1672
26.195	3298.49276	1929	2316	1667
26.228	3291.2163	1890	2363	1635
26.261	3283.95002	1844	2390	1610
26.294	3276.69426	1845	2424	1638
26.327	3269.44936	1860	2417	1642
26.36	3262.21564	1841	2387	1648
26.393	3254.99344	1835	2306	1642
26.426	3247.78308	1800	2355	1589
26.459	3240.58486	1807	2400	1618
26.492	3233.3991	1816	2339	1637
26.525	3226.22609	1789	2259	1626
26.558	3219.06615	1822	2233	1638
26.591	3211.91956	1850	2271	1604
26.624	3204.78661	1814	2303	1635
26.657	3197.66759	1792	2274	1648

26.69	3190.56276	1792	2256	1556
26.723	3183.47241	1772	2280	1568
26.756	3176.39679	1763	2245	1631
26.789	3169.33618	1803	2217	1628
26.822	3162.29083	1794	2223	1619
26.855	3155.26098	1717	2269	1616
26.888	3148.2469	1745	2246	1588
26.921	3141.24882	1778	2186	1532
26.954	3134.26698	1727	2159	1535
26.987	3127.3016	1677	2144	1541
27.02	3120.35293	1692	2112	1559
27.053	3113.42118	1739	2109	1629
27.086	3106.50657	1708	2134	1604
27.119	3099.60931	1725	2134	1545
27.152	3092.72962	1687	2073	1522
27.185	3085.86769	1680	2011	1488
27.218	3079.02373	1703	2044	1508
27.251	3072.19794	1726	2102	1544
27.284	3065.39049	1695	2056	1533
27.317	3058.60159	1682	1991	1551
27.35	3051.83141	1698	2009	1543
27.383	3045.08012	1657	1980	1497
27.416	3038.34791	1601	2039	1581
27.449	3031.63494	1686	1967	1572
27.482	3024.94137	1697	1901	1457
27.515	3018.26737	1644	1975	1490
27.548	3011.6131	1598	1998	1533
27.581	3004.97869	1561	1992	1424
27.614	2998.36431	1593	1907	1531
27.647	2991.77009	1573	1885	1438
27.68	2985.19618	1578	1903	1448
27.713	2978.6427	1593	1936	1422
27.746	2972.1098	1579	1931	1414
27.779	2965.5976	1661	1989	1343
27.812	2959.10622	1571	1925	1405
27.845	2952.63579	1561	1923	1418
27.878	2946.18642	1551	1884	1438
27.911	2939.75821	1559	1889	1472
27.944	2933.35129	1544	1858	1389
27.977	2926.96575	1573	1901	1378
28.01	2920.6017	1605	1803	1342

28.043	2914.25923	1532	1823	1334
28.076	2907.93844	1493	1822	1384
28.109	2901.63941	1442	1822	1423
28.142	2895.36224	1546	1784	1361
28.175	2889.10701	1561	1753	1334
28.208	2882.8738	1391	1771	1268
28.241	2876.66268	1493	1709	1305
28.274	2870.47372	1533	1742	1330
28.307	2864.307	1502	1774	1274
28.34	2858.16259	1552	1759	1313
28.373	2852.04054	1468	1723	1318
28.406	2845.94091	1481	1765	1283
28.439	2839.86377	1417	1733	1265
28.472	2833.80916	1473	1733	1285
28.505	2827.77714	1444	1715	1345
28.538	2821.76775	1482	1751	1325
28.571	2815.78103	1452	1799	1229
28.604	2809.81704	1437	1690	1309
28.637	2803.87579	1459	1652	1256
28.67	2797.95734	1407	1634	1245
28.703	2792.06172	1388	1688	1310
28.736	2786.18894	1441	1653	1279
28.769	2780.33905	1462	1621	1306
28.802	2774.51206	1407	1606	1292
28.835	2768.70799	1418	1659	1243
28.868	2762.92686	1425	1628	1237
28.901	2757.16869	1398	1605	1242
28.934	2751.43349	1391	1603	1255
28.967	2745.72128	1374	1581	1210
29	2740.03205	1366	1551	1199
29.033	2734.36581	1327	1616	1188
29.066	2728.72258	1347	1619	1192
29.099	2723.10233	1377	1579	1197
29.132	2717.50509	1363	1594	1212
29.165	2711.93083	1343	1552	1201
29.198	2706.37956	1333	1543	1181
29.231	2700.85126	1334	1643	1173
29.264	2695.34592	1306	1661	1199
29.297	2689.86353	1299	1551	1225
29.33	2684.40408	1320	1492	1177
29.363	2678.96753	1285	1512	1144

29.396	2673.55389	1240	1528	1150
29.429	2668.16311	1272	1515	1169
29.462	2662.79519	1309	1547	1088
29.495	2657.45008	1286	1551	1089
29.528	2652.12777	1247	1520	1155
29.561	2646.82822	1255	1550	1130
29.594	2641.5514	1229	1500	1131
29.627	2636.29727	1261	1479	1197
29.66	2631.06581	1296	1449	1172
29.693	2625.85696	1288	1503	1133
29.726	2620.67071	1244	1492	1095
29.759	2615.50699	1231	1515	1069
29.792	2610.36577	1249	1498	1032
29.825	2605.24701	1270	1471	1067
29.858	2600.15065	1250	1371	1075
29.891	2595.07666	1244	1426	1085
29.924	2590.02497	1290	1458	1110
29.957	2584.44413	1269	1388	1091
29.99	2579.29413	1227	1440	1091
30.023	2573.82943	1226	1433	1154
30.056	2568.56944	1231	1469	1082
30.089	2563.04594	1190	1432	1029
30.122	2557.77329	1216	1401	1100
30.155	2552.72934	1209	1434	1015
30.188	2547.32002	1238	1434	1068
30.221	2542.08756	1163	1371	1090
30.254	2536.8918	1149	1418	1074
30.287	2531.939	1188	1406	1045
30.32	2526.84955	1145	1340	1026
30.353	2522.03195	1150	1393	1067
30.386	2517.29471	1149	1374	1088
30.419	2512.04376	1140	1303	1113
30.452	2506.97839	1142	1337	1073
30.485	2502.03225	1124	1350	1114
30.518	2497.0402	1178	1319	1040
30.551	2492.15294	1203	1410	1003
30.584	2487.93109	1167	1344	1041
30.617	2483.09823	1177	1362	1005
30.65	2478.48102	1130	1392	1063
30.683	2473.62877	1101	1316	1072
30.716	2468.87527	1143	1269	997

30.749	2464.2619	1089	1324	1013
30.782	2459.57409	1137	1311	999
30.815	2455.18004	1106	1328	1042
30.848	2450.66537	1121	1269	1036
30.881	2446.21496	1059	1250	958
30.914	2441.60726	1086	1296	1012
30.947	2436.75205	1064	1318	1040
30.98	2432.21887	1094	1238	1054
31.013	2427.88847	1123	1286	1065
31.046	2423.64329	1100	1340	1030
31.079	2419.46838	1121	1314	1047
31.112	2415.18893	1112	1365	1022
31.145	2410.8396	1108	1354	1038
31.178	2406.23031	1137	1345	1026
31.211	2401.6016	1160	1360	1002
31.244	2397.18925	1147	1372	1045
31.277	2393.23199	1168	1344	1053
31.31	2389.11681	1223	1325	1008
31.343	2384.5679	1223	1290	1034
31.376	2380.06417	1158	1271	1060
31.409	2376.3147	1185	1330	1030
31.442	2372.47674	1178	1352	1018
31.475	2368.59173	1152	1348	1030
31.508	2365.00127	1101	1315	991
31.541	2361.2445	1068	1294	971
31.574	2357.28361	1056	1311	957
31.607	2353.28852	1072	1297	990
31.64	2349.45227	1076	1262	989
31.673	2345.5383	1039	1245	957
31.706	2341.69761	1015	1220	981
31.739	2337.66946	1039	1194	987
31.772	2333.41251	1055	1219	986
31.805	2329.4911	1041	1239	951
31.838	2325.94024	1018	1245	915
31.871	2322.12717	996	1273	918
31.904	2318.08173	1010	1248	921
31.937	2314.30878	1051	1234	945
31.97	2310.27713	1055	1274	945
32.003	2306.31972	1034	1268	944
32.036	2302.60848	1033	1248	924
32.069	2298.7029	1026	1229	917

32.102	2294.73397	1017	1187	951
32.135	2291.20806	1012	1182	943
32.168	2287.6094	1035	1200	935
32.201	2283.76697	1016	1165	934
32.234	2280.46836	978	1134	961
32.267	2276.79236	995	1210	937
32.3	2272.96329	1005	1183	929
32.333	2269.30665	986	1143	933
32.366	2265.65432	1053	1144	917
32.399	2262.23014	994	1215	931
32.432	2258.66087	971	1198	921
32.465	2254.92491	998	1178	938
32.498	2251.19135	989	1173	949
32.531	2248.35527	996	1127	913
32.564	2245.34149	962	1184	893
32.597	2241.78865	987	1163	879
32.63	2238.36068	978	1152	898
32.663	2235.2759	993	1178	898
32.696	2232.31438	956	1167	891
32.729	2228.87118	1018	1159	904
32.762	2225.56648	946	1104	923
32.795	2222.15369	988	1155	915
32.828	2218.78751	985	1158	922
32.861	2215.52126	987	1242	849
32.894	2212.43635	948	1163	871
32.927	2209.09541	1002	1166	873
32.96	2205.59386	996	1130	873
32.993	2202.45447	892	1125	876
33.026	2199.22834	948	1147	834
33.059	2196.48533	965	1138	880
33.092	2193.35976	924	1161	862
33.125	2190.34914	935	1070	828
33.158	2187.24223	917	1093	865
33.191	2184.24981	908	1172	886
33.224	2181.47209	913	1102	832
33.257	2178.69394	909	1103	866
33.29	2175.97754	925	1161	867
33.323	2173.13895	955	1077	857
33.356	2170.52526	936	1066	895
33.389	2167.75385	931	1056	845
33.422	2165.23896	949	1085	868

33.455	2162.38407	924	1085	867
33.488	2159.48624	979	1168	880
33.521	2156.61075	927	1153	875
33.554	2153.88927	927	1128	823
33.587	2151.38598	897	1139	872
33.62	2148.57137	893	1157	854
33.653	2146.21603	881	1144	830
33.686	2143.39922	944	1074	830
33.719	2140.65208	958	1114	840
33.752	2138.02627	946	1102	881
33.785	2135.61575	936	1085	841
33.818	2133.22677	937	1102	873
33.851	2130.81225	936	1151	899
33.884	2128.31783	926	1089	857
33.917	2125.88435	884	1120	850
33.95	2123.38845	903	1105	843
33.983	2120.87698	923	1092	863
34.016	2118.6922	938	1092	860
34.049	2116.21551	895	1159	864
34.082	2113.82184	890	1165	870
34.115	2111.53574	882	1158	841
34.148	2109.1449	880	1129	818
34.181	2106.88319	852	1111	819
34.214	2104.8803	880	1098	856
34.247	2102.89825	935	1090	884
34.28	2100.7293	917	1092	851
34.313	2098.5034	862	1101	829
34.346	2096.31496	861	1121	863
34.379	2094.27164	869	1094	862
34.412	2092.29571	888	1066	842
34.445	2090.6395	886	1075	844
34.478	2088.81142	877	1078	833
34.511	2086.8027	913	1100	793
34.544	2085.302	922	1152	782
34.577	2083.5447	927	1144	833
34.61	2081.65435	906	1101	843
34.643	2079.68917	875	1090	794
34.676	2077.49579	886	1103	772
34.709	2075.28269	898	1137	809
34.742	2073.50444	900	1100	809
34.775	2071.6452	868	1081	760

34.808	2069.6776	831	1057	785
34.841	2067.71835	827	1097	813
34.874	2065.62498	840	1129	839
34.907	2063.37103	868	1079	848
34.94	2061.3797	875	1036	820
34.973	2058.85276	888	1078	826
35.006	2056.51796	855	1093	805
35.039	2054.68187	846	1058	778
35.072	2052.5655	875	1057	767
35.105	2050.31387	880	1094	803
35.138	2047.8015	862	1119	806
35.171	2046.03537	852	1117	795
35.204	2044.34634	861	1115	866
35.237	2042.3193	829	1062	812
35.27	2040.44516	840	1049	836
35.303	2038.64544	877	1104	846
35.336	2036.84105	857	1176	773
35.369	2034.72571	883	1087	781
35.402	2032.69682	848	1030	846
35.435	2030.82023	899	1053	801
35.468	2029.05758	864	1015	816
35.501	2027.25205	833	1054	774
35.534	2025.73321	865	1000	794
35.567	2024.15626	885	1091	864
35.6	2022.50923	838	1064	855
35.633	2020.84356	889	1077	752
35.666	2018.97408	895	1094	884
35.699	2017.31548	804	1124	860
35.732	2015.89007	863	1136	859
35.765	2014.49753	801	1151	859
35.798	2013.01357	837	1190	881
35.831	2011.66594	861	1152	857
35.864	2010.2322	924	1128	880
35.897	2008.76387	944	1175	888
35.93	2007.08051	887	1204	808
35.963	2005.35965	886	1135	829
35.996	2003.49197	948	1161	859
36.029	2001.97658	913	1136	875
36.062	2000.35304	923	1203	858
36.095	1998.80801	915	1216	869
36.128	1997.09268	875	1225	833

36.161	1995.69034	897	1176	817
36.194	1994.36551	896	1137	883
36.227	1993.06789	901	1145	892
36.26	1991.76224	912	1158	882
36.293	1990.69887	936	1196	862
36.326	1989.4078	942	1228	836
36.359	1988.47721	878	1258	863
36.392	1987.52879	907	1256	846
36.425	1986.57396	958	1280	880
36.458	1985.43789	978	1240	933
36.491	1984.58894	984	1249	910
36.524	1983.89501	977	1269	928
36.557	1983.03404	953	1266	918
36.59	1982.36335	920	1295	931
36.623	1981.74279	925	1282	949
36.656	1981.18726	973	1208	977
36.689	1980.25807	1040	1208	945
36.722	1979.13845	1024	1295	958
36.755	1978.09698	1014	1361	988
36.788	1976.90624	1051	1336	994
36.821	1975.56826	1067	1366	995
36.854	1974.08159	1041	1430	996
36.887	1972.47106	1090	1402	1012
36.92	1970.7808	1129	1395	1040
36.953	1969.51175	1138	1433	1039
36.986	1968.06793	1177	1483	1051
37.019	1966.76765	1180	1521	1088
37.052	1965.38326	1172	1514	1112
37.085	1963.73918	1191	1544	1163
37.118	1962.14376	1221	1582	1195
37.151	1960.45454	1241	1612	1177
37.184	1958.91272	1243	1661	1205
37.217	1957.55304	1269	1693	1255
37.25	1956.09838	1319	1737	1304
37.283	1954.54512	1347	1793	1373
37.316	1953.43657	1388	1878	1421
37.349	1952.02605	1462	1956	1497
37.382	1950.50594	1525	2050	1538
37.415	1949.41321	1628	2167	1586
37.448	1948.38728	1702	2245	1724
37.481	1947.06316	1795	2293	1804

37.514	1946.11026	1832	2568	1925
37.547	1944.64903	1950	2771	2040
37.58	1943.74033	2125	2918	2162
37.613	1942.95521	2308	3055	2341
37.646	1941.48938	2446	3285	2549
37.679	1940.26162	2643	3537	2728
37.712	1939.37017	2896	3831	2945
37.745	1938.16833	3152	4321	3184
37.778	1936.91843	3479	4692	3445
37.811	1935.58083	3938	5080	3679
37.844	1934.36219	4344	5557	3718
37.877	1932.82182	4719	5897	3899
37.91	1931.2413	5299	6514	4200
37.943	1929.58528	5978	6859	4374
37.976	1927.94483	6556	7336	4570
38.009	1926.29683	6916	7568	4593
38.042	1924.62094	7509	7796	4625
38.075	1922.71361	7686	7622	4562
38.108	1920.88918	7576	7329	4338
38.141	1919.20886	6957	6733	4166
38.174	1917.76302	6381	6323	3728
38.207	1916.31565	5808	5629	3454
38.24	1914.81964	5200	5138	3044
38.273	1913.49406	4625	4611	2842
38.306	1912.22653	4121	4168	2600
38.339	1911.05143	3553	3892	2625
38.372	1909.62201	3129	3596	2209
38.405	1908.05794	3089	3324	2081
38.438	1906.54965	2711	3093	2009
38.471	1905.07993	2569	2926	1917
38.504	1903.81393	2365	2810	1786
38.537	1902.50854	2171	2596	1694
38.57	1901.32089	2023	2456	1660
38.603	1899.99796	1970	2333	1596
38.636	1898.87508	1817	2201	1508
38.669	1897.88115	1778	2102	1421
38.702	1896.80315	1694	2087	1433
38.735	1895.49945	1651	1972	1376
38.768	1894.3051	1558	1892	1321
38.801	1893.15575	1499	1833	1305
38.834	1891.91772	1500	1739	1260

38.867	1890.54058	1353	1759	1205
38.9	1889.17484	1395	1673	1184
38.933	1888.01788	1363	1617	1204
38.966	1886.89943	1332	1683	1122
38.999	1885.89333	1294	1558	1128
39.032	1884.68637	1208	1574	1101
39.065	1883.59915	1194	1549	1135
39.098	1882.3065	1217	1467	1087
39.131	1881.09119	1181	1527	1047
39.164	1880.03813	1173	1507	1049
39.197	1878.65538	1139	1443	1033
39.23	1877.31479	1172	1428	1028
39.263	1875.91818	1130	1448	1058
39.296	1874.56378	1118	1417	1029
39.329	1873.32418	1093	1413	964
39.362	1872.14706	1043	1406	989
39.395	1871.10819	1040	1375	991
39.428	1870.16961	1038	1369	955
39.461	1869.54059	1063	1334	940
39.494	1868.53241	1054	1324	966
39.527	1867.5304	1040	1333	1003
39.56	1866.28627	1048	1322	969
39.593	1865.32994	1059	1293	904
39.626	1863.91666	1053	1274	911
39.659	1862.44551	1010	1271	911
39.692	1861.24092	978	1271	909
39.725	1859.77441	979	1282	921
39.758	1858.50153	975	1275	922
39.791	1857.10745	990	1224	960
39.824	1855.49707	1013	1187	928
39.857	1853.923	1000	1206	898
39.89	1852.46967	1010	1209	932
39.923	1851.1216	992	1207	902
39.956	1849.6485	977	1184	881
39.989	1848.31153	932	1201	925
40.022	1846.65028	938	1224	915
40.055	1845.34094	964	1241	894
40.088	1844.09343	975	1236	862
40.121	1842.72552	997	1191	911
40.154	1841.06369	957	1178	887
40.187	1839.59656	961	1182	892

40.22	1838.15676	954	1189	883
40.253	1836.62522	922	1172	850
40.286	1835.05896	926	1144	865
40.319	1833.63111	929	1166	867
40.352	1832.39647	954	1187	864
40.385	1830.92895	936	1136	868
40.418	1829.37521	919	1207	850
40.451	1827.97254	925	1219	857
40.484	1826.58661	948	1235	859
40.517	1825.38616	921	1161	836
40.55	1824.33281	933	1183	838
40.583	1823.33471	897	1151	869
40.616	1822.04883	932	1164	848
40.649	1820.67776	968	1169	915
40.682	1819.41772	934	1183	897
40.715	1817.76075	966	1159	880
40.748	1816.16449	957	1271	873
40.781	1814.66321	981	1204	902
40.814	1813.19498	880	1211	895
40.847	1811.86374	883	1196	886
40.88	1810.51492	939	1165	894
40.913	1809.23627	974	1131	868
40.946	1808.07898	923	1173	808
40.979	1806.9681	936	1202	855
41.012	1805.55482	946	1148	894
41.045	1803.89983	890	1140	889
41.078	1802.45559	941	1196	891
41.111	1801.3828	897	1208	886
41.144	1799.93396	989	1160	891
41.177	1798.40125	903	1147	877
41.21	1797.09678	945	1142	910
41.243	1795.5719	936	1146	882
41.276	1793.97124	1001	1190	861
41.309	1792.57821	985	1167	861
41.342	1790.92543	942	1152	843
41.375	1789.61274	907	1147	865
41.408	1788.28996	917	1203	886
41.441	1786.99492	983	1146	890
41.474	1785.65066	924	1158	901
41.507	1783.7594	934	1125	826
41.54	1782.28827	941	1176	889

41.573	1780.76694	954	1150	877
41.606	1779.36852	956	1163	828
41.639	1778.0323	965	1167	870
41.672	1776.69968	983	1170	893
41.705	1775.2066	979	1187	890
41.738	1773.61325	979	1201	903
41.771	1771.94141	935	1150	943
41.804	1770.41788	940	1168	944
41.837	1769.04743	981	1199	925
41.87	1767.76457	990	1173	888
41.903	1766.39984	1006	1186	905
41.936	1764.91271	991	1170	914
41.969	1763.2708	1010	1174	957
42.002	1761.618	1011	1165	936
42.035	1759.96602	955	1166	900
42.068	1758.38627	990	1162	939
42.101	1756.75116	1016	1114	925
42.134	1754.98746	1002	1140	900
42.167	1753.01345	984	1164	920
42.2	1750.90756	992	1178	960
42.233	1748.8913	1006	1193	950
42.266	1746.40243	999	1192	902
42.299	1743.79792	990	1220	924
42.332	1740.97219	984	1241	960
42.365	1737.81266	993	1260	960
42.398	1734.48031	1001	1244	954
42.431	1730.85255	1001	1199	951
42.464	1726.98954	1011	1186	924
42.497	1723.08306	1055	1216	904
42.53	1719.10237	1032	1244	925
42.563	1715.25534	1006	1223	946
42.596	1711.7377	1013	1201	965
42.629	1708.10633	1072	1218	934
42.662	1704.78188	1086	1278	973
42.695	1701.59879	1078	1250	1012
42.728	1698.67332	1074	1220	1011
42.761	1695.68873	1039	1204	999
42.794	1692.72587	1071	1268	992
42.827	1689.91539	1094	1249	993
42.86	1687.28596	1150	1265	1002
42.893	1684.81264	1164	1282	1014

42.926	1682.34994	1114	1352	1008
42.959	1679.88785	1142	1347	989
42.992	1677.36449	1175	1293	989
43.025	1674.62939	1200	1308	1027
43.058	1671.87405	1172	1361	1085
43.091	1669.23147	1195	1410	1047
43.124	1666.78787	1177	1344	1068
43.157	1664.14369	1222	1391	1128
43.19	1661.81952	1186	1418	1103
43.223	1659.05442	1190	1429	1146
43.256	1656.30618	1267	1486	1132
43.289	1653.68226	1264	1441	1108
43.322	1651.02703	1307	1510	1163
43.355	1648.26536	1340	1508	1190
43.388	1645.9286	1274	1499	1189
43.421	1643.44172	1348	1552	1190
43.454	1640.85886	1385	1644	1164
43.487	1638.44883	1265	1600	1285
43.52	1635.95109	1384	1623	1304
43.553	1633.66064	1475	1655	1242
43.586	1631.14766	1509	1700	1278
43.619	1628.36042	1531	1664	1383
43.652	1625.60159	1510	1767	1412
43.685	1622.94121	1604	1773	1385
43.718	1620.14349	1601	1840	1396
43.751	1617.56211	1639	1908	1475
43.784	1614.83216	1724	1952	1470
43.817	1612.16656	1780	2017	1479
43.85	1609.60216	1879	2077	1614
43.883	1606.98119	1920	2167	1635
43.916	1604.47556	1984	2240	1650
43.949	1601.97927	2121	2328	1704
43.982	1599.47614	2226	2384	1756
44.015	1596.88752	2341	2481	1886
44.048	1594.27416	2453	2622	1945
44.081	1591.68319	2561	2641	1895
44.114	1589.07095	2666	2762	1896
44.147	1586.1799	2739	2887	1945
44.18	1583.43833	2841	2846	1991
44.213	1580.76435	2861	2855	1949
44.246	1578.18337	2896	2785	1900

44.279	1575.95582	3022	2780	1891
44.312	1573.52478	2903	2756	1914
44.345	1571.01139	2716	2596	1809
44.378	1568.46094	2635	2440	1687
44.411	1565.95445	2523	2311	1633
44.444	1563.49383	2294	2198	1575
44.477	1561.21029	2161	2143	1531
44.51	1558.80157	2058	2049	1470
44.543	1556.32957	1949	1932	1405
44.576	1553.79767	1836	1786	1423
44.609	1551.34515	1732	1738	1351
44.642	1548.72467	1689	1759	1299
44.675	1546.21829	1673	1640	1269
44.708	1544.19653	1639	1573	1230
44.741	1541.94045	1562	1584	1187
44.774	1539.56326	1513	1555	1152
44.807	1537.12378	1460	1527	1137
44.84	1534.8256	1420	1511	1131
44.873	1532.49708	1393	1465	1108
44.906	1530.02448	1334	1416	1070
44.939	1527.83143	1298	1411	1047
44.972	1525.71403	1315	1378	1043
45.005	1523.2678	1316	1304	1057
45.038	1520.857	1228	1248	1033
45.071	1518.46185	1175	1227	998
45.104	1516.23082	1164	1228	988
45.137	1514.16347	1144	1220	998
45.17	1511.74134	1126	1200	985
45.203	1509.29968	1090	1179	939
45.236	1506.94711	1074	1144	963
45.269	1504.44158	1050	1169	977
45.302	1502.09214	1064	1174	930
45.335	1499.88882	1045	1131	893
45.368	1497.63787	1039	1123	884
45.401	1495.15328	1035	1142	865
45.434	1492.46686	1014	1132	899
45.467	1489.77507	985	1103	881
45.5	1487.45438	986	1092	847
45.533	1485.10761	986	1050	878
45.566	1482.88619	992	1070	830
45.599	1480.79265	941	1032	839

45.632	1478.48349	947	1066	849
45.665	1476.56082	937	1004	836
45.698	1474.55325	931	1030	834
45.731	1472.46633	886	983	837
45.764	1470.30612	894	1017	770
45.797	1468.13862	947	981	851
45.83	1465.72339	934	1027	850
45.863	1463.41646	960	992	779
45.896	1461.39227	909	943	783
45.929	1459.25448	878	1033	797
45.962	1457.26243	870	910	803
45.995	1455.13732	936	1014	745
46.028	1453.3059	865	906	758
46.061	1451.32061	878	931	759
46.094	1449.62003	874	983	778
46.127	1447.85284	849	963	773
46.16	1445.77584	830	995	764
46.193	1444.02834	841	933	791
46.226	1442.35378	811	922	756
46.259	1440.91086	885	962	752
46.292	1439.27343	853	928	815
46.325	1437.52414	825	904	714
46.358	1435.88897	835	885	734
46.391	1434.2037	804	852	779
46.424	1432.36036	880	921	778
46.457	1430.60502	800	908	744
46.49	1429.04602	844	927	746
46.523	1427.53008	849	913	745
46.556	1426.12715	797	871	747
46.589	1424.6208	841	903	750
46.622	1423.13214	854	936	729
46.655	1421.78529	843	926	797
46.688	1420.55545	805	867	763
46.721	1419.17642	794	885	732
46.754	1417.58053	799	952	750
46.787	1416.06265	790	907	738
46.82	1414.83518	800	897	726
46.853	1413.82234	792	931	739
46.886	1412.74384	806	878	702
46.919	1411.41831	792	859	696
46.952	1410.03403	792	827	700

46.985	1408.76909	782	839	733
47.018	1407.4026	770	872	742
47.051	1406.24063	804	923	714
47.084	1404.92916	802	919	689
47.117	1403.5997	770	880	716
47.15	1402.28541	751	895	739
47.183	1401.0579	749	891	701
47.216	1399.94556	748	841	670
47.249	1398.71567	739	826	701
47.282	1397.72824	753	893	709
47.315	1396.58217	746	916	684
47.348	1395.39312	756	875	652
47.381	1394.17582	752	845	685
47.414	1393.04775	742	847	718
47.447	1391.85015	744	855	705
47.48	1390.70766	740	850	690
47.513	1389.53086	754	844	691
47.546	1388.28993	763	821	709
47.579	1387.12527	722	798	710
47.612	1385.97596	691	811	695
47.645	1385.17592	729	813	667
47.678	1384.03113	724	791	679
47.711	1382.51215	713	790	712
47.744	1381.10151	729	798	697
47.777	1379.77961	713	819	659
47.81	1378.43607	747	851	675
47.843	1377.0302	753	855	687
47.876	1375.45689	706	842	669
47.909	1373.86349	730	841	663
47.942	1372.32533	732	807	674
47.975	1370.46659	693	785	658
48.008	1368.56768	671	811	651
48.041	1366.73199	696	832	627
48.074	1364.5841	729	824	646
48.107	1362.2171	726	828	633
48.14	1359.77042	722	829	590
48.173	1357.67788	679	861	646
48.206	1355.21009	698	868	640
48.239	1352.85916	696	842	632
48.272	1350.29347	666	818	659
48.305	1347.43796	689	814	649

48.338	1344.44094	697	816	634
48.371	1341.71962	693	792	625
48.404	1338.83955	721	788	660
48.437	1336.29103	656	785	684
48.47	1333.58239	690	825	653
48.503	1330.83459	672	814	640
48.536	1328.53488	687	847	673
48.569	1326.02271	682	845	686
48.602	1323.48862	674	838	646
48.635	1321.01561	762	877	636
48.668	1318.76348	655	838	644
48.701	1316.49407	672	810	625
48.734	1314.09377	684	830	633
48.767	1311.911	670	831	654
48.8	1309.581	684	827	602
48.833	1307.53634	698	827	628
48.866	1305.13964	634	759	626
48.899	1302.89626	704	793	651
48.932	1300.33257	665	785	626
48.965	1297.82967	670	736	648
48.998	1295.50158	747	767	642
49.031	1293.14384	682	810	655
49.064	1290.99584	667	818	658
49.097	1289.10769	671	786	655
49.13	1286.9325	667	786	620
49.163	1284.52794	674	756	638
49.196	1282.25031	660	743	649
49.229	1280.1158	638	776	631
49.262	1277.89042	649	743	632
49.295	1275.8184	667	778	640
49.328	1273.5142	657	753	582
49.361	1270.86429	640	778	643
49.394	1268.45041	628	736	690
49.427	1266.25846	671	770	659
49.46	1264.05275	665	745	612
49.493	1261.81597	686	746	634
49.526	1259.74189	650	771	618
49.559	1257.82232	643	764	609
49.592	1255.79487	618	827	604
49.625	1253.73859	635	810	601
49.658	1251.56401	659	767	604

49.691	1249.44831	663	756	602
49.724	1247.28649	665	715	622
49.757	1245.16393	638	735	625
49.79	1243.22998	639	741	611
49.823	1241.11979	630	721	613
49.856	1239.09104	612	722	585
49.889	1237.06562	632	719	567
49.922	1235.00005	648	762	604
49.955	1232.98304	653	767	614
49.988	1230.95864	660	741	601
50.021	1228.93075	658	748	587
50.054	1226.84164	663	741	558
50.087	1224.89641	650	723	571
50.12	1222.88873	625	712	607
50.153	1220.92418	618	712	586
50.186	1219.05003	622	730	576
50.219	1217.07044	634	751	600
50.252	1215.27824	631	747	593
50.285	1213.59172	618	733	579
50.318	1211.6995	623	717	582
50.351	1209.73586	629	731	589
50.384	1207.89689	626	770	562
50.417	1205.83518	634	724	574
50.45	1204.13926	635	691	585
50.483	1201.96938	626	715	585
50.516	1199.88547	596	705	558
50.549	1198.07111	592	683	545
50.582	1196.30598	599	717	582
50.615	1194.45998	646	713	615
50.648	1192.47247	614	706	600
50.681	1190.61517	623	702	615
50.714	1188.8021	594	700	605
50.747	1187.25317	638	734	563
50.78	1185.98226	615	732	606
50.813	1184.24441	589	723	579
50.846	1182.36183	602	693	596
50.879	1180.6984	626	728	600
50.912	1178.92015	567	709	577
50.945	1177.15692	563	723	586
50.978	1175.30508	572	669	579
51.011	1173.67595	623	717	516

51.044	1172.06371	641	711	508
51.077	1170.42561	607	761	568
51.11	1168.52988	632	663	577
51.143	1166.68936	586	678	574
51.176	1165.11202	610	746	583
51.209	1163.64919	617	743	572
51.242	1161.56093	610	674	555
51.275	1159.58297	604	647	600
51.308	1158.07755	604	653	543
51.341	1156.15776	613	699	541
51.374	1154.32088	575	736	536
51.407	1152.70521	557	668	563
51.44	1150.91146	637	726	565
51.473	1149.10188	594	690	537
51.506	1147.46048	598	678	604
51.539	1145.85483	615	723	550
51.572	1144.31404	594	680	573
51.605	1142.72456	602	686	528
51.638	1140.91779	596	700	553
51.671	1139.18599	575	689	545
51.704	1137.6147	590	697	561
51.737	1135.99954	616	719	558
51.77	1134.43246	585	739	586
51.803	1133.06283	596	698	569
51.836	1131.31149	592	699	548
51.869	1129.58399	581	724	533
51.902	1128.15059	608	728	530
51.935	1126.71508	579	679	554
51.968	1125.12099	595	675	596
52.001	1123.62004	604	727	544
52.034	1122.10518	565	668	550
52.067	1120.4874	575	671	599
52.1	1118.85902	589	690	573
52.133	1117.35904	571	680	545
52.166	1115.92988	573	674	582
52.199	1114.4128	578	710	591
52.232	1112.99483	590	741	561
52.265	1111.5466	616	754	561
52.298	1110.08397	603	744	577
52.331	1108.74037	591	694	575
52.364	1107.31619	581	681	558

52.397	1105.74335	578	713	552
52.43	1104.21136	565	701	570
52.463	1102.75477	572	685	552
52.496	1101.27617	588	697	545
52.529	1099.77539	609	726	530
52.562	1098.39277	599	697	546
52.595	1097.043	600	645	571
52.628	1095.71074	609	651	560
52.661	1094.29415	610	673	566
52.694	1092.83637	608	679	583
52.727	1091.43915	588	689	575
52.76	1090.28775	586	691	570
52.793	1089.21306	599	665	575
52.826	1088.02245	591	642	563
52.859	1086.88226	588	660	527
52.892	1085.63957	577	696	503
52.925	1084.32879	568	713	542
52.958	1083.03937	602	702	547
52.991	1081.69158	565	662	511
53.024	1080.41795	570	650	531
53.057	1079.17506	578	696	518
53.09	1078.00944	569	678	513
53.123	1076.67263	556	640	545
53.156	1075.22293	532	663	530
53.189	1073.88925	544	655	515
53.222	1072.88019	549	613	539
53.255	1071.80997	553	618	543
53.288	1070.70929	574	636	523
53.321	1069.52751	583	645	515
53.354	1068.41832	558	634	523
53.387	1067.10488	571	621	545
53.42	1065.81754	585	647	561
53.453	1064.78768	597	677	559
53.486	1063.42041	539	639	558
53.519	1062.34087	557	631	532
53.552	1061.29097	548	619	516
53.585	1060.26786	584	601	503
53.618	1059.11187	569	628	532
53.651	1058.15521	528	619	500
53.684	1057.07566	513	585	516
53.717	1055.90086	548	629	492

53.75	1054.72887	549	623	502
53.783	1053.71146	584	612	533
53.816	1052.76536	556	597	523
53.849	1051.59956	548	651	515
53.882	1050.6306	561	632	552
53.915	1049.73295	572	643	521
53.948	1048.73341	550	579	539
53.981	1047.80981	550	649	534
54.014	1046.45016	519	626	491
54.047	1045.08674	547	648	488
54.08	1044.01429	580	605	528
54.113	1042.99055	524	673	537
54.146	1041.89289	556	666	537
54.179	1040.81289	534	613	514
54.212	1039.74859	543	597	540
54.245	1038.68191	539	636	529
54.278	1037.32046	559	632	520
54.311	1036.31306	511	633	544
54.344	1035.37588	537	615	532
54.377	1034.25782	557	628	516
54.41	1032.92456	542	630	470
54.443	1031.61935	578	612	501
54.476	1030.40022	531	610	516
54.509	1029.35825	548	641	511
54.542	1028.1359	517	621	512
54.575	1026.66797	543	595	513
54.608	1025.35503	559	633	503
54.641	1024.16375	548	599	524
54.674	1023.03702	535	579	530
54.707	1021.7932	503	595	528
54.74	1020.48615	535	592	512
54.773	1019.48032	539	567	523
54.806	1018.62769	521	569	523
54.839	1017.80138	509	605	518
54.872	1016.79472	489	633	500
54.905	1015.82809	516	616	503
54.938	1014.89334	506	608	513
54.971	1013.95982	514	621	510
55.004	1012.88697	551	603	506
55.037	1011.73698	542	609	518
55.07	1010.63685	530	636	526

55.103	1009.55719	508	638	499
55.136	1008.45721	532	609	501
55.169	1007.46953	562	597	498
55.202	1006.55552	556	603	491
55.235	1005.59878	508	601	495
55.268	1004.35148	472	601	490
55.301	1003.1298	488	606	481
55.334	1001.89941	504	615	505
55.367	1000.60645	516	579	520
55.4	999.42068	538	563	487
55.433	998.24459	528	592	495
55.466	997.26983	514	604	497
55.499	996.32462	525	610	490
55.532	995.1382	516	608	519
55.565	994.00257	534	627	512
55.598	992.81326	545	594	488
55.631	991.72819	554	605	499
55.664	990.42371	561	632	530
55.697	989.38325	554	610	534
55.73	988.42144	543	598	563
55.763	987.42325	544	606	536
55.796	986.47469	569	625	520
55.829	985.45146	566	624	492
55.862	984.18033	532	587	503
55.895	983.20721	532	569	505
55.928	982.24568	533	622	509
55.961	981.38153	533	612	495
55.994	980.31675	524	598	489
56.027	979.11813	500	593	504
56.06	977.97738	519	564	557
56.093	976.92208	521	605	483
56.126	975.65608	523	606	459
56.159	974.67435	535	572	507
56.192	973.45137	502	588	448
56.225	972.16217	480	603	497
56.258	970.72428	502	586	486
56.291	969.45789	520	604	479
56.324	967.8871	524	600	461
56.357	966.02037	463	551	468
56.39	964.36017	510	568	487
56.423	962.54675	474	537	499

56.456	960.83258	506	642	485
56.489	958.90106	507	569	467
56.522	957.19744	508	580	497
56.555	955.61565	468	552	483
56.588	953.73186	465	594	477
56.621	952.0547	521	568	483
56.654	950.53103	508	594	469
56.687	949.2344	490	613	502
56.72	947.96449	466	638	483
56.753	946.69033	488	589	498
56.786	945.31513	517	551	492
56.819	944.15719	524	566	511
56.852	942.97248	484	544	462
56.885	941.69471	507	553	491
56.918	940.52349	509	559	501
56.951	939.4089	515	585	503
56.984	938.36783	496	580	477
57.017	937.25097	504	581	478
57.05	936.23465	499	549	467
57.083	935.30627	509	526	451
57.116	934.39379	485	566	474
57.149	933.42475	476	554	498
57.182	932.48721	516	564	471
57.215	931.58998	488	568	508
57.248	930.56933	506	569	495
57.281	929.49723	496	600	471
57.314	928.44715	480	564	490
57.347	927.5795	494	541	516
57.38	926.7388	495	552	502
57.413	925.76641	504	590	479
57.446	924.79376	460	561	458
57.479	923.95252	483	552	472
57.512	922.97918	494	538	492
57.545	921.84168	488	517	503
57.578	920.84469	490	517	487
57.611	920.0254	497	542	477
57.644	919.14811	501	563	471
57.677	918.34628	507	575	455
57.71	917.55836	515	591	438
57.743	916.81192	498	566	464
57.776	916.05753	467	524	519

57.809	915.20365	462	554	499
57.842	914.15601	492	572	460
57.875	913.10902	502	556	472
57.908	912.28196	496	554	506
57.941	911.59631	499	522	499
57.974	910.71816	474	527	467
58.007	909.74439	480	559	472
58.04	908.87677	497	545	467
58.073	907.93004	491	551	467
58.106	906.82512	485	557	494
58.139	905.92267	444	537	508
58.172	904.98159	458	519	492
58.205	904.1996	478	526	460
58.238	903.45925	493	538	454
58.271	902.67065	487	543	475
58.304	902.11839	524	548	485
58.337	901.37897	541	582	444
58.37	900.60247	514	570	447
58.403	899.53439	493	544	465
58.436	898.65011	509	545	464
58.469	897.72669	472	513	479
58.502	896.77296	503	527	497
58.535	895.89755	444	554	478
58.568	895.21201	459	546	463
58.601	894.54425	464	531	458
58.634	893.87025	501	533	488
58.667	893.18372	507	581	490
58.7	892.52753	463	569	476
58.733	891.72494	442	513	451
58.766	890.80135	479	555	452
58.799	890.02383	484	534	468
58.832	889.03647	464	566	477
58.865	888.08819	499	527	469
58.898	887.06175	488	571	439
58.931	886.28561	474	521	458
58.964	885.67685	476	540	478
58.997	884.8049	487	529	455
59.03	883.97171	481	525	454
59.063	883.2575	466	532	440
59.096	882.63289	456	522	501
59.129	882.0211	473	557	411

59.162	881.42574	449	560	472
59.195	880.75076	482	549	464
59.228	880.11077	470	537	436
59.261	879.33062	486	500	489
59.294	878.4241	476	539	471
59.327	877.64003	478	515	448
59.36	876.64676	487	507	455
59.393	875.84007	440	563	460
59.426	874.90283	460	523	465
59.459	874.06211	495	573	488
59.492	873.26269	432	550	443
59.525	872.37564	491	518	433
59.558	871.58161	471	495	493
59.591	870.83031	451	516	464
59.624	870.06288	430	544	434
59.657	869.20595	493	528	474
59.69	868.47755	497	519	476
59.723	867.75768	462	541	444
59.756	866.77684	445	534	454
59.789	865.92634	463	512	442
59.822	865.05047	446	538	458
59.855	864.3609	447	509	445
59.888	863.52999	445	531	426
59.921	862.66164	430	511	435
59.954	861.8855	462	509	454
59.987	861.2655	455	521	449
60.02	860.72004	494	495	441
60.053	860.14729	486	493	451
60.086	859.58122	456	499	438
60.119	859.03202	450	526	450
60.152	858.39773	463	507	456
60.185	857.61219	469	514	445
60.218	856.91772	438	509	471
60.251	856.39251	475	527	478
60.284	855.90867	488	547	495
60.317	855.28881	449	520	487
60.35	854.55538	428	511	428
60.383	853.8784	441	499	436
60.416	853.09635	452	476	478
60.449	852.43281	444	502	482
60.482	851.81979	456	513	470

60.515	851.25799	472	504	449
60.548	850.7595	445	521	459
60.581	850.15154	417	527	473
60.614	849.43085	444	535	452
60.647	848.82897	463	545	462
60.68	848.2943	440	527	466
60.713	847.65043	459	499	451
60.746	847.03275	473	496	456
60.779	846.35381	454	516	465
60.812	845.76647	494	523	485
60.845	845.162	476	514	479
60.878	844.6259	433	534	445
60.911	844.15936	463	533	445
60.944	843.59649	447	495	444
60.977	843.06908	435	532	443
61.01	842.41295	460	546	446
61.043	841.75157	463	520	462
61.076	841.02851	440	525	460
61.109	840.54443	475	493	463
61.142	839.87066	458	527	458
61.175	839.47038	438	541	457
61.208	838.95778	463	515	461
61.241	838.46055	454	508	449
61.274	837.83166	473	523	447
61.307	836.96464	460	508	446
61.34	836.60944	438	518	444
61.373	836.08649	443	497	452
61.406	835.62275	435	489	431
61.439	835.17274	445	528	475
61.472	834.7477	462	545	474
61.505	834.10262	483	506	472
61.538	833.69411	462	552	442
61.571	833.24814	426	519	475
61.604	832.69751	439	483	504
61.637	831.99697	462	526	450
61.67	831.36983	468	505	430
61.703	830.73891	510	466	447
61.736	830.16612	478	492	468
61.769	829.70849	455	512	477
61.802	829.41633	508	516	427
61.835	829.20107	471	527	466

61.868	828.89066	472	548	467
61.901	828.29662	486	548	486
61.934	827.82488	442	543	436
61.967	827.384	441	597	457
62	826.99546	476	526	449
62.033	826.55391	505	473	447
62.066	825.93597	452	552	470
62.099	825.46904	492	533	449
62.132	824.882	481	522	427
62.165	824.43875	469	505	474
62.198	824.04576	478	511	479
62.231	823.45064	467	537	502
62.264	822.94941	473	559	444
62.297	822.42673	436	587	500
62.33	821.84987	471	566	463
62.363	821.58956	496	545	473
62.396	820.93837	472	507	455
62.429	820.43566	473	498	453
62.462	820.05165	478	534	448
62.495	819.7114	489	550	471
62.528	819.2657	518	560	463
62.561	818.82312	489	534	465
62.594	818.36806	478	544	468
62.627	817.90472	521	552	451
62.66	817.23534	498	565	474
62.693	816.67735	476	566	501
62.726	816.16629	508	544	495
62.759	815.64954	513	562	493
62.792	815.26307	497	550	492
62.825	814.96646	495	577	488
62.858	814.51956	515	540	495
62.891	814.0687	505	543	481
62.924	813.6081	507	547	467
62.957	813.17302	506	545	491
62.99	812.71927	510	576	494
63.023	812.1303	520	607	511
63.056	811.78695	520	600	524
63.089	811.36342	528	588	504
63.122	810.86421	520	575	489
63.155	810.48394	497	572	487
63.188	810.09263	508	612	492

63.221	809.56607	535	641	507
63.254	809.04233	548	644	517
63.287	808.79713	557	642	532
63.32	808.48779	560	649	547
63.353	808.19051	544	642	545
63.386	807.73057	539	634	563
63.419	807.27046	598	655	557
63.452	806.84476	595	677	548
63.485	806.42366	581	683	541
63.518	806.13518	593	670	578
63.551	805.99978	621	699	579
63.584	805.68799	623	746	608
63.617	805.56417	623	748	629
63.65	805.26256	651	745	629
63.683	804.84784	651	744	609
63.716	804.41344	658	757	636
63.749	803.74206	682	811	672
63.782	803.22639	682	864	697
63.815	802.81065	708	855	710
63.848	802.40248	718	886	703
63.881	801.91874	750	936	733
63.914	801.5075	851	946	778
63.947	801.16064	867	973	805
63.98	800.9081	876	1017	796
64.013	800.6074	916	1026	818
64.046	800.32168	964	1124	876
64.079	800.05746	970	1219	949
64.112	799.49326	1079	1225	911
64.145	798.97065	1142	1299	994
64.178	798.64965	1241	1337	1021
64.211	798.35969	1322	1365	1021
64.244	797.80167	1385	1468	1142
64.277	797.2963	1503	1499	1139
64.31	796.80666	1563	1571	1179
64.343	796.12759	1700	1622	1164
64.376	795.50045	1654	1642	1253
64.409	795.01086	1628	1571	1159
64.442	794.44689	1652	1628	1147
64.475	794.0153	1645	1537	1136
64.508	793.54319	1625	1533	1070
64.541	792.82324	1473	1451	1056

64.574	792.29062	1426	1409	1060
64.607	791.88715	1368	1371	957
64.64	791.33208	1324	1284	930
64.673	790.61334	1208	1177	865
64.706	789.94529	1173	1150	869
64.739	789.4615	1067	1071	778
64.772	788.83133	1055	1074	788
64.805	788.15855	978	947	780
64.838	787.6097	952	1002	744
64.871	786.96763	925	962	716
64.904	786.28085	896	879	683
64.937	785.47043	792	857	680
64.97	784.67113	744	835	681
65.003	783.98397	760	791	652
65.036	783.27659	782	791	613
65.069	782.4793	761	776	620
65.102	781.69393	663	746	619
65.135	781.1132	617	775	604
65.168	780.45229	624	800	590
65.201	779.77738	610	717	585
65.234	779.30738	626	685	589
65.267	778.71669	630	683	576
65.3	778.13739	596	668	574
65.333	777.61603	586	657	539
65.366	777.35972	602	662	530
65.399	776.81131	598	626	548
65.432	776.24217	584	625	525
65.465	775.73001	558	621	531
65.498	775.07142	543	631	556
65.531	774.56445	565	641	528
65.564	773.99818	553	603	497
65.597	773.39737	528	570	487
65.63	772.97777	533	573	493
65.663	772.43383	524	576	506
65.696	771.86525	523	579	489
65.729	771.30691	532	577	503
65.762	770.86226	518	562	485
65.795	770.27596	503	544	462
65.828	769.87368	486	546	492
65.861	769.2714	487	558	474
65.894	768.78781	491	551	452

65.927	768.13836	470	534	449
65.96	767.58269	498	507	465
65.993	766.96927	529	502	471
66.026	766.46292	495	549	459
66.059	765.92287	467	575	461
66.092	765.1956	460	527	444
66.125	764.61489	452	520	462
66.158	763.97887	439	511	465
66.191	763.42676	431	502	462
66.224	762.87989	458	517	463
66.257	762.27204	462	532	436
66.29	761.72018	455	526	466
66.323	761.11008	458	498	452
66.356	760.60867	465	509	449
66.389	760.01468	455	531	471
66.422	759.43522	469	527	449
66.455	758.84451	450	532	480
66.488	758.31326	449	518	440
66.521	757.71224	449	522	423
66.554	757.05231	476	471	441
66.587	756.63887	418	482	436
66.62	756.33767	455	458	412
66.653	755.73497	438	495	429
66.686	755.1533	423	491	450
66.719	754.62985	423	532	435
66.752	754.10558	445	548	425
66.785	753.58818	448	476	423
66.818	753.00261	467	491	441
66.851	752.56664	450	508	422
66.884	752.1166	422	511	442
66.917	751.80231	456	499	399
66.95	751.31074	422	478	428
66.983	750.90287	443	508	431
67.016	750.65889	394	478	424
67.049	750.30581	434	490	444
67.082	749.78769	461	486	374
67.115	749.34811	436	487	405
67.148	749.04165	441	492	443
67.181	748.65188	408	493	395
67.214	748.00871	418	470	435
67.247	747.432	435	455	443

67.28	747.03306	410	497	413
67.313	746.58839	459	479	414
67.346	746.16152	440	482	420
67.379	745.40605	445	489	394
67.412	744.63344	383	515	405
67.445	743.82008	450	458	416
67.478	742.97402	405	479	410
67.511	742.34409	438	471	442
67.544	741.74694	462	500	435
67.577	740.94951	420	509	423
67.61	740.32572	432	483	421
67.643	739.65476	431	465	425
67.676	738.85608	397	478	414
67.709	737.79202	393	518	437
67.742	736.70638	394	491	403
67.775	735.78734	410	467	404
67.808	734.84258	418	468	399
67.841	734.01717	433	474	399
67.874	733.22557	411	473	386
67.907	732.30786	438	461	408
67.94	731.53673	457	453	394
67.973	730.65717	398	486	369
68.006	729.87967	381	475	362
68.039	728.97658	386	441	408
68.072	728.14184	384	447	426
68.105	727.39784	380	466	401
68.138	726.69171	386	459	401
68.171	725.99882	423	451	402
68.204	725.26611	412	460	392
68.237	724.54809	415	490	389
68.27	723.74202	424	478	396
68.303	722.81276	412	446	415
68.336	721.94199	390	457	408
68.369	721.28098	384	475	389
68.402	720.63117	399	458	397
68.435	719.77664	414	438	406
68.468	718.85023	433	464	390
68.501	718.11651	422	469	369
68.534	717.09578	412	454	382
68.567	716.15598	410	473	402
68.6	715.337	409	464	386

68.633	714.52763	394	462	372
68.666	713.67887	387	485	390
68.699	712.8044	404	486	407
68.732	711.98544	415	461	403
68.765	711.22931	410	466	387
68.798	710.42396	403	488	399
68.831	709.63733	399	489	401
68.864	708.99597	417	470	411
68.897	708.08902	433	468	384
68.93	707.16828	426	452	409
68.963	706.45317	381	436	411
68.996	705.7378	375	472	389
69.029	704.98566	404	450	392
69.062	704.0623	399	436	394
69.095	703.33319	427	464	409
69.128	702.4298	462	431	403
69.161	701.70134	419	433	390
69.194	701.17672	400	470	394
69.227	700.56971	379	444	375
69.26	699.84714	383	444	333
69.293	699.02728	414	476	388
69.326	698.41886	396	439	381
69.359	697.64746	406	397	392
69.392	696.84296	418	443	392
69.425	696.17736	413	464	367
69.458	695.41414	394	446	395
69.491	694.64246	401	477	414
69.524	694.00129	415	459	393
69.557	693.42518	437	415	384
69.59	692.92367	425	470	404
69.623	692.31807	398	441	405
69.656	691.81239	414	438	388
69.689	691.045	404	501	361
69.722	690.50252	368	477	418
69.755	690.03947	381	419	396
69.788	689.51763	387	415	392
69.821	688.86272	387	503	358
69.854	688.39602	415	441	358
69.887	688.16666	406	434	392
69.92	687.48855	351	448	372
69.953	686.91566	406	463	349

69.986	686.40851	450	443	413
70.019	685.8484	419	415	420
70.052	685.45815	380	435	388
70.085	690.59235	401	436	366
70.118	690.16099	368	463	375
70.151	689.73499	355	473	406
70.184	689.31438	411	491	382
70.217	688.89918	377	465	383
70.25	688.4894	404	446	413
70.283	688.08505	385	434	369
70.316	687.68615	386	415	395
70.349	687.29272	371	425	399
70.382	686.90477	378	451	364
70.415	686.52232	398	443	373
70.448	686.14536	403	438	361
70.481	685.77393	403	465	380
70.514	685.40803	384	473	403
70.547	685.04767	401	470	399
70.58	684.69286	417	476	410
70.613	684.34361	399	418	370
70.646	683.99993	404	435	353
70.679	683.66183	397	455	386
70.712	683.32932	386	458	392
70.745	683.0024	384	447	387
70.778	682.68109	380	440	388
70.811	682.36538	391	446	398
70.844	682.05528	377	441	384
70.877	681.7508	382	458	371
70.91	681.45194	396	465	396
70.943	681.15871	402	448	373
70.976	680.87111	398	425	366
71.009	680.58913	371	430	394
71.042	680.31279	370	441	385
71.075	680.04208	363	431	366
71.108	679.77701	369	458	373
71.141	679.51756	388	449	403
71.174	679.26375	377	435	397
71.207	679.01557	385	461	391
71.24	678.77302	378	448	382
71.273	678.5361	382	454	384
71.306	678.30479	401	428	376

71.339	678.07911	397	459	382
71.372	677.85904	408	471	404
71.405	677.64457	416	457	399
71.438	677.43571	401	469	411
71.471	677.23245	366	473	390
71.504	677.03477	371	470	398
71.537	676.84266	353	454	374
71.57	676.65613	348	455	376
71.603	676.47517	362	465	382
71.636	676.29975	393	444	389
71.669	676.12987	401	435	388
71.702	675.96552	385	419	380
71.735	675.80669	402	430	433
71.768	675.65336	381	443	402
71.801	675.50552	365	456	400
71.834	675.36315	391	437	378
71.867	675.22625	391	438	367
71.9	675.09479	404	424	362
71.933	674.96876	397	449	363
71.966	674.84815	416	440	371
71.999	674.73292	413	466	366
72.032	674.62308	353	445	357
72.065	674.51859	400	455	364
72.098	674.41944	363	439	376
72.131	674.3256	360	475	404
72.164	674.23706	387	443	400
72.197	674.1538	414	454	416
72.23	674.07578	384	475	317
72.263	674.00299	418	450	404
72.296	673.93541	399	465	368
72.329	673.87301	418	459	353
72.362	673.81575	387	486	401
72.395	673.76363	382	447	373
72.428	673.71661	393	430	392
72.461	673.67466	443	472	379
72.494	673.63775	414	452	373
72.527	673.60586	372	440	395
72.56	673.57896	371	464	363
72.593	673.55701	395	449	403
72.626	673.53999	346	420	384
72.659	673.52786	373	442	401

72.692	673.52058	412	454	396
72.725	673.51814	411	451	379
72.758	673.52048	383	468	373
72.791	673.52759	353	455	394
72.824	673.53941	375	448	405
72.857	673.55592	384	453	391
72.89	673.57707	389	454	363
72.923	673.60284	388	452	354
72.956	673.63318	361	433	369
72.989	673.66805	346	441	387
73.022	673.70741	396	468	408
73.055	673.75122	419	472	357
73.088	673.79944	424	447	360
73.121	673.85202	388	442	398
73.154	673.90893	380	452	380
73.187	673.97012	383	446	360
73.22	674.03554	389	450	362
73.253	674.10516	403	448	365
73.286	674.17891	390	440	374
73.319	674.25676	379	466	394
73.352	674.33866	379	447	381
73.385	674.42456	364	451	404
73.418	674.5144	370	469	395
73.451	674.60815	399	452	378
73.484	674.70575	394	443	372
73.517	674.80714	397	468	361
73.55	674.91228	407	474	382
73.583	675.02111	427	458	392
73.616	675.13357	439	440	375
73.649	675.24962	417	446	375
73.682	675.36919	415	455	390
73.715	675.49224	400	451	393
73.748	675.61869	399	457	409
73.781	675.7485	416	451	410
73.814	675.88161	398	463	367
73.847	676.01795	399	440	371
73.88	676.15747	384	444	398
73.913	676.3001	383	453	365
73.946	676.44579	384	467	368
73.979	676.59446	403	469	379
74.012	676.74606	415	484	378

74.045	676.90052	413	497	391
74.078	677.05777	400	477	403
74.111	677.21775	396	451	403
74.144	677.3804	400	476	372
74.177	677.54564	407	471	385
74.21	677.7134	424	454	391
74.243	677.88363	422	477	408
74.276	678.05623	432	458	433
74.309	678.23116	449	467	399
74.342	678.40832	430	499	413
74.375	678.58765	404	462	436
74.408	678.76908	408	484	397
74.441	678.95254	450	513	392
74.474	679.13793	448	460	432
74.507	679.3252	415	482	439
74.54	679.51426	423	466	416
74.573	679.70504	425	479	417
74.606	679.89745	429	452	400
74.639	680.09142	434	471	368
74.672	680.28686	469	480	400
74.705	680.4837	440	446	434
74.738	680.68185	468	489	396
74.771	680.88123	419	478	399
74.804	681.08176	470	460	420
74.837	681.28335	446	449	397
74.87	681.48591	409	485	425
74.903	681.68937	396	478	449
74.936	681.89362	406	447	386
74.969	682.09859	454	483	368
75.002	682.30418	372	489	406
75.035	682.5103	402	495	445
75.068	682.71687	417	466	439
75.101	682.92379	424	490	439
75.134	683.13097	438	467	397
75.167	683.33832	420	478	398
75.2	683.54573	447	504	429
75.233	683.75312	451	521	426
75.266	683.96039	417	497	402
75.299	684.16745	402	527	436
75.332	684.37419	427	512	448
75.365	684.58051	448	445	407

75.398	684.78632	419	482	415
75.431	684.99151	450	535	417
75.464	685.19599	475	523	394
75.497	685.39965	430	539	430
75.53	685.60239	430	495	416
75.563	685.8041	456	506	442
75.596	686.00468	483	512	475
75.629	686.20402	483	504	462
75.662	686.40202	459	498	457
75.695	686.59857	446	484	435
75.728	686.79355	478	528	444
75.761	686.98686	485	535	431
75.794	687.17839	479	550	449
75.827	687.36803	487	553	453
75.86	687.55566	479	528	467
75.893	687.74117	451	529	480
75.926	687.92445	472	566	485
75.959	688.10538	491	551	477
75.992	688.28385	488	540	486
76.025	688.45973	492	589	486
76.058	688.63291	535	598	483
76.091	688.80327	541	603	497
76.124	688.97069	531	612	526
76.157	689.13505	525	617	518
76.19	689.29623	541	623	506
76.223	689.45411	553	604	522
76.256	689.60855	530	595	519
76.289	689.75944	533	621	531
76.322	689.90665	558	639	556
76.355	690.05006	580	642	559
76.388	690.18953	544	680	546
76.421	690.32494	547	702	545
76.454	690.45616	597	700	572
76.487	690.58305	638	692	609
76.52	690.70549	692	716	610
76.553	690.82335	650	760	586
76.586	690.93649	656	769	594
76.619	691.04477	690	765	610
76.652	691.14806	732	831	638
76.685	691.24623	753	847	660
76.718	691.33913	747	864	675

76.751	691.42664	753	878	687
76.784	691.5086	749	903	753
76.817	691.58489	833	1008	719
76.85	691.65535	851	1003	778
76.883	691.71985	843	1022	825
76.916	691.77824	902	1036	821
76.949	691.83038	957	1055	830
76.982	691.87612	1022	1136	849
77.015	691.91533	1070	1184	887
77.048	691.94784	1157	1261	945
77.081	691.97351	1169	1303	1024
77.114	691.9922	1233	1352	1042
77.147	692.00375	1301	1423	1049
77.18	692.00802	1327	1438	1112
77.213	692.00484	1459	1523	1081
77.246	691.99407	1588	1559	1127
77.279	691.97555	1647	1591	1084
77.312	691.94913	1645	1577	1215
77.345	691.91465	1666	1577	1123
77.378	691.87195	1642	1529	1100
77.411	691.82088	1616	1493	1114
77.444	691.76128	1527	1568	1086
77.477	691.69298	1488	1529	1032
77.51	691.61583	1482	1455	983
77.543	691.52966	1337	1293	966
77.576	691.43431	1319	1263	919
77.609	691.32961	1274	1242	901
77.642	691.21541	1268	1213	897
77.675	691.09152	1149	1118	843
77.708	690.95779	1026	1060	841
77.741	690.81405	1045	1031	812
77.774	690.66013	1006	1041	762
77.807	690.49585	951	996	755
77.84	690.32105	923	902	776
77.873	690.13555	840	929	741
77.906	689.93918	793	871	653
77.939	689.73176	789	840	636
77.972	689.51312	757	836	653
78.005	689.28308	741	758	651
78.038	689.04147	696	731	623
78.071	688.7881	692	785	615

78.104	688.52279	689	709	604
78.137	688.24537	683	681	602
78.17	687.95565	690	727	586
78.203	687.65344	681	687	578
78.236	687.33857	636	687	541
78.269	687.01085	623	660	522
78.302	686.67009	622	643	520
78.335	686.3161	615	669	515
78.368	685.9487	580	686	523
78.401	685.56769	551	688	537
78.434	685.17289	554	646	501
78.467	684.7641	558	624	517
78.5	684.34113	537	613	557
78.533	683.90379	538	604	539
78.566	683.45188	556	586	507
78.599	682.9852	556	579	515
78.632	682.50356	535	579	502
78.665	682.00676	534	574	484
78.698	681.4946	538	560	482
78.731	680.96688	548	567	496
78.764	680.4234	542	593	508
78.797	679.86395	527	555	482
78.83	679.28833	520	502	470
78.863	678.69634	488	504	466
78.896	678.08777	477	537	453
78.929	677.46241	500	557	468
78.962	676.82005	516	544	483
78.995	676.16049	496	524	477
79.028	675.48352	469	500	453
79.061	674.78892	476	502	465
79.094	674.07647	488	531	471
79.127	673.34598	508	526	438
79.16	672.59721	482	521	432
79.193	671.82996	469	520	440
79.226	671.04401	485	519	448
79.259	670.23914	499	510	446
79.292	669.41512	464	488	444
79.325	668.57175	464	511	426
79.358	667.70879	476	503	424
79.391	666.82603	471	505	473
79.424	665.92324	495	549	448

79.457	665.0002	486	525	442
79.49	664.05667	464	502	417
79.523	663.09243	450	534	431
79.556	662.10726	465	492	447
79.589	661.10092	472	474	432
79.622	660.07319	466	499	436
79.655	659.02382	495	481	407
79.688	657.95259	465	517	429
79.721	656.85926	459	516	421
79.754	655.7436	471	490	458
79.787	654.60537	458	498	455
79.82	653.44434	426	490	425
79.853	652.26025	410	499	388
79.886	651.05288	447	476	420
79.919	649.82198	467	489	433
79.952	648.56731	431	462	426

Fig 4 data:

2%		3%		4%	
	size(nm)		size(nm)		size(nm)
**Mean**	4.617391	**Mean**	2.926402	**Mean**	2.494203
**Std. Dev.**	1.962319	**Std. Dev.**	1.079439	**Std. Dev.**	1.613043
	4.888406		2.247664		1.168116
	4.465217		4.233645		1.744928
	8.111594		3.292056		2.049275
	2.613043		1.523364		1.763768
	5.828986		2.231308		0.915942
	4.104348		1.077103		1.792754
	6.068116		1.558411		2.049275
	6.366667		6.32243		0.927536
	7.269565		5.129673		1.62029
	7.22029		3.658879		1.131884
	9.071014		1.518692		1.478261
	8.57971		2.920561		0.927536
	6.165217		1.174065		1.54058
	2.463768		1.901869		1.54058
	4.582609		3.213785		1.769565
	7.592754		1.755841		1.449275
	3.333333		1.684579		2.018841
	4.928986		1.203271		1.750725
	4.747826		2		1.336232
	4.202899		2.303738		1.295652
	1.971014		2.955607		1.62029
	3.191304		2.106308		1.63913
	6.724638		1.004673		2.255072
	7.598551		2.03972		2.173913
	6.611594		2.920561		1.54058
	3.015942		1.985981		2.173913
	4.798551		2.132009		1.652174
	4.304348		2.994159		2.492754
	4.86087		0.261682		1.744928
	4.817391		3.71028		1.304348
	1.168116		2.03972		1.237681
	3.505797		2.464953		1.375362
	2.468116		2.776869		1.744928
	5.124638		2.828271		1.336232
	3.178261		2.016355		1.024638
	6.384058		2.247664		1.56087

	5.771014		5.728972		1.478261
	1.336232		1.943925		1.73913
	1.195652		4.130841		1.815942
	6.088406		3.925234		3.588406
	0.927536		3.712617		1.54058
	1.230435		2.043224		1.295652
	0.647826		2.984813		1.131884
	0.844928		3.953271		0.844928
	1.195652		3.405374		1.449275
	3.894203		2.497664		1.104348
	6.489855		4.01986		1.024638
	6.231884		3.658879		1.366667
	5.507246		1.406542		1.750725
	5.942029		3.767523		2.155072
	4.582609		3.459112		1.844928
	3.081159		1.901869		1.652174
	5.73913		1.672897		1.237681
	6.191304		1.518692		1.492754
	3.75942		4.463785		1.237681
	4.4		4.390187		5.056522
	5.975362		2.348131		2.291304
	6.191304		3.03271		1.884058
	3.101449		3.419393		1.971014
	3.256522		3.28972		6.885507
	6.252174		2.219626		1.652174
	5.124638		4.264019		1.492754
	6.681159		5.998832		1.456522
	2.563768		3.396028		1.246377
	8.376812		4.336449		1.769565
	5.14058		2.464953		1.689855
	6.686957		2.75		7.043478
	0.724638		4.941589		4.826087
	3.828986		4.175234		3.081159
	5.73913		1.635514		1.492754
	7.072464		2.348131		2.988406
	1.055072		1.406542		4.262319
	0.972464		3.522196		4.86087
	1.375362		1.779206		2.788406
	3.657971		2.338785		5.307246
	2.988406		3.668224		1.594203
	4.407246		2.825935		0.927536

	7.471014		4.328271		1.336232
	2.663768		2.885514		4.817391
	5.644928		2.223131		5.637681
	5.975362		2.070093		2.898551
	1.246377		1.651869		5.885507
	7.47971		4.233645		2.089855
	5.469565		4.621495		1.856522
	4.213043		3.057243		6.471014
	6.17971		2.561916		5.030435
	5.252174		2.803738		4.986957
	6.353623		1.869159		6.691304
	1.750725		1.901869		2.411594
	1.044928		3.767523		3.384058
	1.131884		2.526869		1.695652
	1.336232		1.73715		1.336232
	1.246377		2.301402		1.689855
	4.144928		2.978972		3.75942
	4.550725		2.147196		2.268116
	7.971014		4.810748		3.304348
	5.771014		3.03972		8.426087
	5.991304		3.03972		1.949275
	3.97971		4.725467		1.15942
	6.005797		4.397196		5.030435
	5.8		2.483645		2.318841
	5.975362		3.207944		6.142029
	3.771014		6.649533		1.336232
	1.601449		3.973131		1.652174
	5.275362		2.69743		2.263768
	2.463768		3.084112		5.753623
	1.237681		2.625		2.263768
	1.230435		1.813084		1.513043
	7.791304		2.464953		1.304348
	6.095652		4.698598		1.246377
	3.128986		6.823598		1.769565
	5.849275		2.079439		5.005797
	5.718841		2.734813		3.502899
	1.884058		5.056075		5.16087
	6.223188		5.024533		5.869565
	5.030435		5.879673		4.150725
	4.605797		4.01986		3.942029
	6.25942		3.213785		5.447826

	3.634783		1.73715		3.436232
	5.65942		2.806075		1.434783
	5.753623		3.449766		1.949275
	3.285507		2.172897		1.744928
	2.613043		3.857477		1.237681
	6.971014		3.732477		3.902899
	7.294203		2.172897		2.973913
	3.489855		1.88785		5.455072
	5.14058		2.823598		1.884058
	2.049275		4.143692		2.468116
	4.369565		3.247664		1.944928
	6.436232		4.087617		6.756522
	6.152174		3.156542		2.530435
	2.898551		2.892523		3.285507
	5.828986		1.957944		3.986957
	3.986957		2.686916		1.295652
	0.881159		2.336449		1.856522
	4.104348		3.98715		1.313043
	4.06087		1.496495		1.456522
	5.321739		3.292056		2.075362
	5.975362		2.978972		1.54058
	4.927536		2.892523		1.449275
	1.449275		2.689252		1.54058
	7.911594		3.247664		1.750725
	4.73913		3.084112		1.336232
	2.217391		4.633178		1.195652
	7.328986		3.761682		2.049275
	6.871014		2.561916		1.295652
	1.427536		3.084112		1.427536
	0.82029		2.147196		1.594203
	1.844928		2.564252		3.77971
	2.857971		2.14486		1.844928
	3.623188		3.154206		1.949275
	5.669565		1.567757		5.185507
	4.547826		4.175234		
	3.762319		3.904206		
	4.791304		2.823598		
	6.027536		2.336449		
	3.478261		1.901869		
	1.54058		2.247664		
	6.027536		2.75		

	6.836232		3.046729			
	5.508696		3.297897			
	1.427536		3.363318			
	1.168116		3.531542			
	3.395652		3.17757			
	3.77971		5.016355			
	5.024638		3.651869			
	7.185507		5.573598			
	3.201449		5.705607			
	4.928986		2.561916			
	5.828986		2.561916			
	7.407246		1.915888			
	5.842029		3.63785			
	7.74058		4.561916			
	6.272464		2.365654			
	5.455072		1.78271			
	0.781159		5.550234			
	2.268116		2.313084			
	5.024638		3.17757			
	4.150725		3.207944			
	4.8		3.003505			
	3.695652		3.634346			
	2.723188		4.841121			
	5.081159		2.920561			
	4.386957		1.684579			
	5.769565		2.204439			
	5.669565		2.727804			
	5.226087		2.689252			
	7.702899		2.195093			
	3.57971		2.408879			
	7.114493		4.212617			
	1.54058		2.477804			
	7.22029		2.066589			
	5.127536		3.452103			
	7.798551		3.964953			
	1.62029		2.561916			
	5.314493		3.156542			
	5.469565		1.426402			
	8.505797		2.350467			
	1.246377		2.268692			
	1.313043		2.593458			

	0.927536		1.241822		
	1.168116		1.869159		
	1.594203		2.57243		
	4.872464		2.864486		
	6.681159		3.057243		
	5.627536		3.566589		
	5.82029		1.523364		
	2.973913		4.561916		
	3.75942		2.219626		
	2.530435		4.050234		
	3.537681		2.089953		
	3.42029		1.264019		
	6.610145		2.204439		
	4.927536		1.943925		
	5.753623		2.268692		
	3.046377		3.156542		
	2.613043		2.231308		
	3.955072		2.974299		
	3.74058		4.115654		
	5.124638		2.948598		
	5.030435		2.885514		
	3.165217		3.134346		
	8.669565		2.873832		
	7.324638		2.564252		
	5.426087		2.561916		
	5.314493		2.669393		
	8.017391		1.989486		
	2.45942		1.755841		
	7.101449		2.757009		
	4.12029		2.009346		
	4.182609		3.761682		
	5.63913		3.405374		
	4.714493		2.350467		
	4.308696		1.989486		
	1.54058		3.968458		
	6.197101		3.321262		
	5.771014		1.901869		
	6.968116		4.714953		
	6.191304		4.561916		
	8.898551		1.883178		
	7.885507		2.625		

	1.478261		2.663551		
	3.311594		3.066589		
	6		3.093458		
	4.624638		3.560748		
	5.518841		1.635514		
	6.376812		2.61215		
	5.913043		3.473131		
	4.53913		3.435748		
	4.718841		2.268692		
	5.555072		2.991822		
	5.457971		2.397196		
	2.35942		4.299065		
	3.792754		1.817757		
	4.630435		2.901869		
	6.946377		2.508178		
	5.627536		2.244159		
	4.826087		1.883178		
	6.927536		4.148364		
	6.813043		2.561916		
	1.652174		3.046729		
	3.711594		4.385514		
	3.336232		3.188084		
	5.968116		1.846963		
	3.942029		3.066589		
	7.542029		1.915888		
	1.889855		4.080607		
	4.785507		1.883178		
	4.785507		1.933411		
	4.465217		1.366822		
	2.784058		2.76986		
	3.97971		2.36215		
	6.805797		3.904206		
	5.681159		3.63785		
	6.476812		3.814252		
	3.346377		3.245327		
	7.391304		1.660047		
	1.449275		1.869159		
	3.395652		3.628505		
	4.12029		2.301402		
	5.252174		4.234813		
	4.86087		2.477804		

	5.162319		1.264019		
	5.769565		1.401869		
	4.547826		1.463785		
	7.37971		2.593458		
	4.64058		2.106308		
	3.304348		2.497664		
	5.862319		1.989486		
	4.888406				
	4.182609				
	7.652174				
	5.785507				
	3.955072				
	7.107246				
	4.613043				
	5.957971				
	4.718841				
	3.588406				
	4.308696				
	5.455072				
	4.8				
	4.843478				
	8.17971				
	4.202899				
	5.362319				
	6.763768				
	3.711594				
	4.057971				
	2.757971				
	4.824638				
	2.013043				
	3.942029				
	4.465217				
	4.308696				
	2.695652				
	7.981159				
	9.891304				
	6.152174				
	5.17971				
	6.202899				
	6.927536				
	7.102899				

	5.234783				
	3.565217				
	5.127536				
	5.5				
	3.957971				
	5.652174				
	4.8				
	5.071014				
	5.275362				
	6.637681				
	5.227536				
	5.314493				
	5.65942				
	8.908696				
	4.747826				
	2.66087				
	6.292754				
	3.9				
	4.843478				
	5.346377				
	4.091304				
	5.431884				
	4.85942				
	2.67971				
	5.447826				
	6.04058				
	4.018841				
	3.850725				
	1.449275				
	1.336232				
	1.246377				
	1.949275				
	1.56087				
	3.191304				
	2.798551				
	2.45942				
	5.508696				
	5.446377				
	6.649275				
	4.465217				
	1.492754				

	1.601449				
	3.346377				
	1.295652				
	1.168116				
	4.17971				
	6.872464				
	4.550725				
	5.913043				
	5.717391				
	5.549275				
	2.898551				
	0.915942				
	5.508696				
	8.726087				
	5.828986				
	5.127536				
	6.471014				
	2.723188				
	4.927536				
	2.35942				
	2.476812				
	8.081159				
	2.336232				
	3.436232				
	3.489855				
	3.804348				
	6.271014				
	5.771014				

Fig 5 data:

wavelength	absorbance	absorbance	absorbance
(nm)	(a.u)	(a.u)	(a.u)
	2%	3%	4%
355	0.15079	0.18944	0.27487
356	0.15342	0.19112	0.27766
357	0.15612	0.19264	0.28025
358	0.15865	0.19384	0.28264
359	0.16137	0.1948	0.28487
360	0.1639	0.19576	0.28695
361	0.16626	0.19696	0.2889
362	0.16883	0.19824	0.29073
363	0.17131	0.19952	0.29247
364	0.17399	0.20072	0.29412
365	0.1763	0.20216	0.2957
366	0.17852	0.20392	0.29723
367	0.18066	0.20576	0.29871
368	0.18334	0.20768	0.30015
369	0.18656	0.2096	0.30157
370	0.18989	0.21184	0.30297
371	0.19256	0.21408	0.30437
372	0.19509	0.21632	0.30576
373	0.19783	0.2188	0.30716
374	0.20092	0.2216	0.30858
375	0.20416	0.22472	0.31001
376	0.20682	0.228	0.31147
377	0.20974	0.23144	0.31295
378	0.21239	0.23472	0.31446
379	0.21563	0.23816	0.316
380	0.21853	0.2416	0.31757
381	0.22164	0.24504	0.31918
382	0.22475	0.24816	0.32083
383	0.22807	0.25168	0.32251
384	0.23147	0.2552	0.32422
385	0.23505	0.25896	0.32597
386	0.2389	0.26288	0.32776
387	0.2427	0.2668	0.32957
388	0.24654	0.27048	0.33141
389	0.25028	0.27384	0.33328
390	0.25404	0.27696	0.33517
391	0.2578	0.28008	0.33707

392	0.26181	0.28352	0.339
393	0.26585	0.28688	0.34093
394	0.26981	0.29016	0.34288
395	0.27363	0.29352	0.34482
396	0.27752	0.29712	0.34676
397	0.28123	0.30064	0.34869
398	0.28503	0.30384	0.35061
399	0.28851	0.30688	0.35252
400	0.29212	0.30992	0.35439
401	0.2954	0.31296	0.35624
402	0.2989	0.31592	0.35805
403	0.30227	0.31904	0.35982
404	0.30551	0.322	0.36154
405	0.30875	0.32472	0.36321
406	0.31199	0.32744	0.36438
407	0.31517	0.32992	0.3658
408	0.31808	0.33232	0.36725
409	0.32098	0.33464	0.3687
410	0.32378	0.33712	0.37
411	0.32664	0.33976	0.37112
412	0.32916	0.34216	0.37237
413	0.3316	0.34472	0.37337
414	0.334	0.34696	0.37401
415	0.33627	0.34904	0.37453
416	0.3386	0.35112	0.37495
417	0.34078	0.35328	0.37527
418	0.34294	0.35544	0.37576
419	0.34493	0.35768	0.37622
420	0.34683	0.35928	0.37662
421	0.34852	0.36072	0.37663
422	0.35003	0.36176	0.37651
423	0.35132	0.3628	0.37606
424	0.35251	0.36408	0.3758
425	0.35364	0.36528	0.3755
426	0.35487	0.36648	0.37531
427	0.35618	0.36752	0.37485
428	0.35711	0.36848	0.37432
429	0.35802	0.3688	0.37368
430	0.35891	0.36888	0.37269
431	0.35979	0.36856	0.37159
432	0.36055	0.36832	0.3702

433	0.3612	0.36784	0.36889
434	0.36171	0.36744	0.36762
435	0.36206	0.36672	0.36582
436	0.36232	0.36608	0.36387
437	0.36247	0.36544	0.36161
438	0.36253	0.36456	0.35942
439	0.36236	0.36344	0.35678
440	0.36208	0.36232	0.35412
441	0.36182	0.36104	0.35107
442	0.3615	0.35992	0.34801
443	0.361	0.3584	0.34493
444	0.36024	0.35656	0.34185
445	0.35947	0.35472	0.33859
446	0.35888	0.35296	0.33539
447	0.35839	0.3512	0.33203
448	0.35778	0.34936	0.32868
449	0.35713	0.34768	0.32549
450	0.35627	0.34576	0.32228
451	0.35523	0.34368	0.31889
452	0.35398	0.3416	0.31555
453	0.35251	0.3396	0.31189
454	0.351	0.33768	0.30862
455	0.34947	0.33584	0.30499
456	0.34787	0.33336	0.30161
457	0.34616	0.33032	0.29794
458	0.34424	0.32704	0.29457
459	0.3423	0.32408	0.2911
460	0.34009	0.32128	0.28752
461	0.33795	0.31848	0.2842
462	0.33569	0.31576	0.28095
463	0.33344	0.31296	0.27776
464	0.33102	0.31032	0.27464
465	0.32856	0.30752	0.27123
466	0.32603	0.30512	0.2679
467	0.32363	0.30232	0.26422
468	0.32113	0.29984	0.26107
469	0.31851	0.2972	0.25799
470	0.31575	0.29472	0.25507
471	0.31283	0.292	0.25199
472	0.31	0.28944	0.24912
473	0.30707	0.2872	0.24602

474	0.30408	0.28528	0.243
475	0.30093	0.28368	0.2404
476	0.29778	0.28192	0.23827
477	0.2946	0.28016	0.23645
478	0.29134	0.27824	0.23415
479	0.28795	0.27664	0.23145
480	0.28454	0.2748	0.22888
481	0.28119	0.27272	0.22629
482	0.27791	0.27096	0.22378
483	0.27456	0.26984	0.22137
484	0.27117	0.26872	0.2194
485	0.26775	0.26768	0.21724
486	0.26434	0.26624	0.21485
487	0.26093	0.26448	0.21264
488	0.25754	0.26248	0.21085
489	0.25419	0.26048	0.20914
490	0.25084	0.25896	0.20712
491	0.24747	0.25752	0.20551
492	0.24417	0.2564	0.20392
493	0.24086	0.25488	0.20256
494	0.23756	0.2532	0.20124
495	0.23438	0.25184	0.19978
496	0.23112	0.2504	0.19855
497	0.2279	0.24856	0.19714
498	0.22468	0.24648	0.19629
499	0.22146	0.24456	0.19486
500	0.21833	0.24272	0.19314
501	0.21522	0.24088	0.19116
502	0.21228	0.23896	0.18935
503	0.20946	0.23696	0.18772
504	0.20663	0.23448	0.18608
505	0.20375	0.23192	0.18476
506	0.20092	0.22936	0.18359
507	0.19818	0.2268	0.18259
508	0.1955	0.22456	0.18137
509	0.19282	0.2224	0.18007
510	0.1901	0.2204	0.17851
511	0.18744	0.21824	0.17708
512	0.18477	0.21584	0.17575
513	0.18226	0.21328	0.17423
514	0.17976	0.21064	0.17283

515	0.17736	0.20824	0.17142
516	0.17505	0.20632	0.16994
517	0.17276	0.20432	0.16846
518	0.17038	0.20248	0.16719
519	0.16803	0.20056	0.16555
520	0.16574	0.1984	0.16419
521	0.16353	0.19648	0.16253
522	0.16135	0.19456	0.16135
523	0.15921	0.19304	0.16016
524	0.15708	0.1916	0.15939
525	0.15496	0.19008	0.15846
526	0.15297	0.18848	0.15769
527	0.15103	0.18664	0.1565
528	0.14906	0.18496	0.15547
529	0.1472	0.18344	0.15445
530	0.14535	0.18224	0.15321
531	0.14351	0.18088	0.15196
532	0.14165	0.17992	0.15073
533	0.13977	0.17872	0.14974
534	0.13789	0.17768	0.14875
535	0.13608	0.17648	0.14734
536	0.13431	0.17544	0.14591
537	0.13258	0.17448	0.14465
538	0.13085	0.17336	0.14354
539	0.1291	0.17208	0.1422
540	0.12744	0.17112	0.14086
541	0.12578	0.17016	0.13978
542	0.12416	0.1692	0.1391
543	0.12258	0.16768	0.13844
544	0.12107	0.16664	0.13763
545	0.11949	0.1656	0.13671
546	0.11794	0.16456	0.13592
547	0.11634	0.16352	0.1353
548	0.11474	0.16232	0.13484
549	0.11316	0.16128	0.13467
550	0.11152	0.16024	0.13451
551	0.1099	0.15896	0.13447
552	0.10828	0.15784	0.1347
553	0.10668	0.15672	0.13449
554	0.10513	0.1556	0.13448
555	0.10357	0.15416	0.13408

556	0.10202	0.15272	0.13402
557	0.10042	0.15152	0.1339
558	0.09876	0.15024	0.13411
559	0.09703	0.14896	0.13411
560	0.09526	0.14784	0.13406
561	0.09351	0.14664	0.13416
562	0.09186	0.1456	0.13387
563	0.09031	0.14408	0.13341
564	0.08867	0.14256	0.13296
565	0.08703	0.14104	0.13267
566	0.08534	0.13976	0.13269
567	0.08366	0.13824	0.13247
568	0.08199	0.13664	0.13239
569	0.0804	0.13448	0.13201
570	0.0788	0.13224	0.13202
571	0.07713	0.13	0.13172
572	0.07556	0.12808	0.13176
573	0.07389	0.12648	0.13177
574	0.07225	0.12496	0.1316
575	0.07057	0.12312	0.13146
576	0.06901	0.12104	0.13081
577	0.06739	0.11904	0.13039
578	0.06577	0.1172	0.1296
579	0.06422	0.11536	0.12918
580	0.06288	0.1136	0.12856
581	0.06156	0.11184	0.12812
582	0.06022	0.11024	0.12767
583	0.05875	0.10864	0.1278
584	0.05737	0.10712	0.12759
585	0.05603	0.10592	0.12741
586	0.05478	0.10448	0.12652
587	0.05346	0.10296	0.1261
588	0.0521	0.10144	0.12577
589	0.0508	0.1	0.12582
590	0.04957	0.09912	0.12568
591	0.04836	0.09792	0.1259
592	0.04724	0.09672	0.12559
593	0.04616	0.09536	0.12564
594	0.04497	0.0944	0.12513
595	0.04383	0.0936	0.125
596	0.04257	0.09256	0.12486

597	0.04154	0.09136	0.12504
598	0.0405	0.09056	0.12477
599	0.03944	0.08952	0.12427
600	0.03838	0.08832	0.12373
601	0.03735	0.08736	0.12319
602	0.03629	0.08632	0.12289
603	0.03521	0.08528	0.12288
604	0.03415	0.08416	0.1231
605	0.03318	0.08328	0.12335
606	0.03227	0.08216	0.12342
607	0.03138	0.08136	0.12347
608	0.0305	0.0804	0.1235
609	0.02966	0.07944	0.1235
610	0.02886	0.07792	0.12365
611	0.02802	0.07656	0.12362
612	0.02719	0.07528	0.12368
613	0.0264	0.07384	0.12362
614	0.02557	0.07272	0.12354
615	0.02478	0.07176	0.12303
616	0.02406	0.0708	0.12264
617	0.02339	0.06984	0.1221
618	0.02264	0.06888	0.12188
619	0.0219	0.06768	0.12174
620	0.0211	0.0664	0.12186
621	0.02039	0.06504	0.1218
622	0.01979	0.0636	0.12173
623	0.01927	0.06232	0.12146
624	0.01868	0.06088	0.12137
625	0.01801	0.05976	0.12141
626	0.01724	0.0588	0.12146
627	0.01639	0.05776	0.1208
628	0.01562	0.05712	0.11988
629	0.01493	0.0564	0.11879
630	0.01421	0.056	0.11788
631	0.01337	0.0552	0.11694
632	0.01253	0.05464	0.11663
633	0.01177	0.0536	0.11616
634	0.01128	0.0528	0.11555
635	0.01074	0.05216	0.11459
636	0.01015	0.05144	0.11364
637	0.00944	0.05096	0.11294

638	0.00873	0.05024	0.11157
639	0.0081	0.0496	0.11014
640	0.00747	0.04888	0.10832
641	0.00683	0.04856	0.10679
642	0.00622	0.04784	0.10488
643	0.00559	0.04688	0.10313
644	0.00497	0.04608	0.10162
645	0.00428	0.04584	0.10081
646	0.00372	0.0456	0.09982
647	0.00309	0.04508	0.09943
648	0.00255	0.04446	0.09892
649	0.00195	0.04397	0.09885
650	0.00143	0.044	0.09828
651	9.22E-04	0.04379	0.09776
652	5.29E-04	0.04398	0.09654
653	1.20E-04	0.04408	0.0952
654	5.25E-04	0.0441	0.09415
655	0.00108	0.04419	0.0936
656	0.00172	0.04437	0.09312
657	0.00225	0.04507	0.09271
658	0.00279	0.0456	0.09247
659	0.0033	0.04574	0.092
660	0.00372	0.04648	0.09166
661	0.00419	0.04736	0.09141
662	0.00451	0.04768	0.09072
663	0.0049	0.048	0.08991
664	0.00527	0.0484	0.08929
665	0.00572	0.04864	0.08905
666	0.00616	0.04912	0.08895
667	0.00659	0.04944	0.08838
668	0.00698	0.0496	0.08797
669	0.00737	0.05	0.08786
670	0.00773	0.0504	0.08746
671	0.00816	0.05144	0.08674
672	0.00832	0.05344	0.08626
673	0.00838	0.05568	0.08615
674	0.00812	0.05736	0.08592
675	0.00804	0.05872	0.08528
676	0.00799	0.06	0.08435
677	0.00832	0.06096	0.08524
678	0.00879	0.06128	0.08589

679	0.00916	0.06208	0.08663
680	0.00948	0.06264	0.08561
681	0.00961	0.06288	0.08424
682	0.01013	0.06256	0.0828
683	0.01093	0.06224	0.08231
684	0.01166	0.06208	0.08263
685	0.0124	0.06216	0.08304
686	0.01294	0.06168	0.08236
687	0.01354	0.0608	0.08271
688	0.014	0.06008	0.08217
689	0.01439	0.0596	0.08256
690	0.01482	0.05904	0.08176
691	0.0151	0.05864	0.08146
692	0.01518	0.05808	0.08021
693	0.01534	0.0576	0.0791
694	0.01555	0.0572	0.07811
695	0.01575	0.05672	0.07759
696	0.01575	0.05608	0.07682
697	0.01566	0.05552	0.07648
698	0.01575	0.05552	0.07586
699	0.01583	0.0556	0.07646
700	0.01572	0.05552	0.07621
701	0.01542	0.0552	0.07663
702	0.01514	0.05448	0.07609
703	0.01495	0.05352	0.07614
704	0.01488	0.05328	0.07502
705	0.01462	0.05232	0.07389
706	0.01441	0.05136	0.07141
707	0.01395	0.05208	0.0694
708	0.01341	0.05128	0.06745
709	0.01268	0.05008	0.06703
710	0.0119	0.04928	0.06611
711	0.01119	0.04872	0.06458
712	0.01063	0.04792	0.06327
713	0.00998	0.04736	0.06237
714	0.00916	0.04792	0.06146
715	0.00827	0.04768	0.05998
716	0.00726	0.04736	0.0592
717	0.00667	0.04768	0.05914
718	0.00622	0.04736	0.05918
719	0.00581	0.0468	0.0592

720	0.00531	0.04648	0.05768
721	0.00456	0.0464	0.05692
722	0.00404	0.04656	0.05555
723	0.00348	0.04688	0.05471
724	0.00337	0.04752	0.053
725	0.0032	0.0488	0.0515
726	0.00294	0.0492	0.04992
727	0.0022	0.04976	0.04767
728	0.00138	0.05104	0.04528
729	6.13E-04	0.05248	0.04335
730	1.24E-05	0.05432	0.04295
731	6.42E-04	0.05488	0.04329
732	0.00151	0.05584	0.04383
733	0.00218	0.05688	0.04359
734	0.00274	0.05864	0.04357
735	0.00279	0.05976	0.04384
736	0.00281	0.06032	0.04394
737	0.00266	0.06064	0.04366
738	0.00264	0.0612	0.04283
739	0.00276	0.06176	0.04187
740	0.00287	0.06208	0.04037
741	0.00296	0.06248	0.03886
742	0.00276	0.06248	0.03748
743	0.00257	0.0632	0.03657
744	0.00222	0.0636	0.03597
745	0.00206	0.06512	0.03509
746	0.0018	0.06552	0.03484
747	0.00147	0.06584	0.03455
748	9.99E-04	0.06552	0.03421
749	6.55E-04	0.06616	0.0333
750	4.62E-04	0.06688	0.03239
751	2.95E-04	0.06704	0.03195
752	8.64E-05	0.0668	0.03091
753	4.87E-04	0.06648	0.03066
754	0.00107	0.06616	0.03032
755	0.00166	0.06584	0.03065
756	0.00225	0.06552	0.03011
757	0.00285	0.06512	0.02958
758	0.00343	0.0648	0.02908
759	0.004	0.06448	0.0286
760	0.00456	0.06416	0.02816

761	0.00508	0.06392	0.02774
762	0.00559	0.0636	0.02736
763	0.00609	0.06344	0.02702
764	0.00654	0.06328	0.02671
765	0.00698	0.06312	0.02645
766	0.00737	0.06312	0.02623
767	0.00773	0.06312	0.02605
768	0.00804	0.06312	0.02592
769	0.00834	0.06328	0.02584
770	0.00858	0.06352	0.02581
771	0.00877	0.06376	0.02583
772	0.00894	0.06408	0.0259
773	0.00907	0.06448	0.02602
774	0.00916	0.06496	0.02619
775	0.00922	0.06552	0.02642
776	0.00924	0.06608	0.0267
777	0.00924	0.06672	0.02702
778	0.00922	0.06744	0.0274
779	0.00918	0.06824	0.02783
780	0.00912	0.06904	0.0283
781	0.00905	0.06984	0.02882
782	0.00899	0.07072	0.02938
783	0.00892	0.07152	0.02997
784	0.00888	0.0724	0.03061
785	0.00886	0.0732	0.03127
786	0.00888	0.074	0.03195
787	0.00894	0.0748	0.03266
788	0.00907	0.07544	0.03338
789	0.00927	0.076	0.03411
790	0.00955	0.07648	0.03484
791	0.00996	0.0768	0.03557
792	0.01045	0.07696	0.03627
793	0.0111	0.07696	0.03696
794	0.01192	0.07664	0.03761
795	0.0129	0.07616	0.03822
796	0.01408	0.07528	0.03877
797	0.01547	0.07416	0.03926
798	0.01713	0.07264	0.03967
799	0.01903	0.07072	0.03998
800	0.02123	0.0684	0.04019
801	0.02376	0.06552	0.04028

Fig 6 data:

hv	$(\alpha hv)^2$	$(\alpha hv)^2$	$(\alpha hv)^2$
**eV**	**$(eV/cm)^2$**	**$(eV/cm)^2$**	**$(eV/cm)^2$**
	**2%**	**3%**	**4%**
5.64722	4851.35009	4950.72427	4123.55953
5.63441	4831.42739	4928.82449	4109.61265
5.62167	4811.29151	4906.75433	4095.43248
5.60898	4790.94026	4884.51396	4081.01017
5.59634	4770.37176	4862.10377	4066.33727
5.58377	4749.58447	4839.52444	4051.40584
5.57125	4728.57719	4816.77686	4036.2084
5.55878	4707.34904	4793.86217	4020.73804
5.54638	4685.8995	4770.78174	4004.98836
5.53402	4664.22838	4747.53721	3988.95357
5.52172	4642.33586	4724.1304	3972.62844
5.50948	4620.22245	4700.5634	3956.00838
5.49729	4597.88902	4676.83849	3939.08943
5.48516	4575.3368	4652.9582	3921.86827
5.47308	4552.56736	4628.92527	3904.34225
5.46105	4529.58262	4604.74262	3886.50938
5.44907	4506.38487	4580.41342	3868.36838
5.43715	4482.97674	4555.94103	3849.91866
5.42528	4459.36118	4531.32898	3831.16031
5.41346	4435.54153	4506.58103	3812.09414
5.40169	4411.52144	4481.70112	3792.72168
5.38997	4387.3049	4456.69335	3773.04513
5.3783	4362.89622	4431.56202	3753.06743
5.36669	4338.30006	4406.31159	3732.79221
5.35512	4313.52139	4380.94671	3712.22378
5.3436	4288.56547	4355.47216	3691.36715

5.33214	4263.4379	4329.8929	3670.228
5.32072	4238.14454	4304.21403	3648.81266
5.30935	4212.69156	4278.44081	3627.12813
5.29803	4187.08541	4252.57862	3605.18203
5.28676	4161.3328	4226.63298	3582.98257
5.27553	4135.4407	4200.60956	3560.53859
5.26436	4109.41634	4174.51412	3537.85945
5.25323	4083.26718	4148.35258	3514.9551
5.24214	4057.0009	4122.13092	3491.83599
5.23111	4030.62539	4095.85527	3468.51304
5.22012	4004.14878	4069.53185	3444.99768
5.20917	3977.57934	4043.16696	3421.30172
5.19828	3950.92555	4016.767	3397.43741
5.18742	3924.19605	3990.33846	3373.41735
5.17662	3897.39961	3963.88789	3349.25448
5.16585	3870.54515	3937.42192	3324.96204
5.15514	3843.64172	3910.94724	3300.55355
5.14446	3816.69847	3884.47061	3276.04273
5.13383	3789.72463	3857.99884	3251.44353
5.12325	3762.72953	3831.53876	3226.77003
5.11271	3735.72256	3805.09727	3202.03644
5.10221	3708.71315	3778.68128	3177.25707
5.09175	3681.71077	3752.29775	3152.44624
5.08134	3654.7249	3725.95363	3127.61831
5.07097	3627.76504	3699.65589	3102.7876
5.06064	3600.84068	3673.41152	3077.96838
5.05036	3573.96126	3647.22748	3053.1748
5.04011	3547.13621	3621.11075	3028.42089
5.02991	3520.37489	3595.06826	3003.72051
5.01975	3493.6866	3569.10694	2979.0873

5.00963	3467.08053	3543.23367	2954.53468
4.99955	3440.56581	3517.45529	2930.0758
4.98951	3414.15143	3491.7786	2905.72351
4.97951	3387.84628	3466.21033	2881.49033
4.96955	3361.65908	3440.75716	2857.38842
4.95963	3335.59843	3415.42567	2833.42957
4.94975	3309.67275	3390.22239	2809.62515
4.93991	3283.89029	3365.15373	2785.98611
4.93011	3258.2591	3340.22602	2762.52293
4.92035	3232.78704	3315.44547	2739.24563
4.91062	3207.48177	3290.81818	2716.16373
4.90094	3182.35071	3266.35012	2693.28625
4.89129	3157.40105	3242.04712	2670.62168
4.88168	3132.63975	3217.91489	2648.17797
4.87211	3108.07351	3193.95897	2625.96253
4.86258	3083.70879	3170.18474	2603.98221
4.85308	3059.55176	3146.59743	2582.24331
4.84362	3035.60832	3123.20207	2560.75153
4.83419	3011.88411	3100.00352	2539.51203
4.82481	2988.38445	3077.00644	2518.52937
4.81546	2965.11439	3054.21531	2497.80756
4.80614	2942.07868	3031.63439	2477.35003
4.79687	2919.28176	3009.26771	2457.15963
4.78762	2896.72777	2987.1191	2437.23867
4.77842	2874.42052	2965.19215	2417.58889
4.76924	2852.36354	2943.49023	2398.21151
4.76011	2830.56002	2922.01646	2379.10718
4.75101	2809.01285	2900.77369	2360.27608
4.74194	2787.72458	2879.76457	2341.71783
4.73291	2766.69747	2858.99145	2323.4316

4.72391	2745.93344	2838.45643	2305.41607
4.71495	2725.43411	2818.16136	2287.66947
4.70602	2705.20078	2798.1078	2270.18958
4.69712	2685.23443	2778.29705	2252.97379
4.68826	2665.53575	2758.73013	2236.01907
4.67943	2646.10511	2739.4078	2219.32203
4.67063	2626.94257	2720.33052	2202.87893
4.66187	2608.0479	2701.49849	2186.68571
4.65314	2589.4206	2682.91162	2170.738
4.64444	2571.05984	2664.56956	2155.03114
4.63578	2552.96455	2646.47166	2139.56025
4.62714	2535.13335	2628.61702	2124.32021
4.61854	2517.56462	2611.00445	2109.30568
4.60997	2500.25646	2593.6325	2094.51117
4.60144	2483.20673	2576.49945	2079.93104
4.59293	2466.41303	2559.60333	2065.55951
4.58446	2449.87276	2542.94191	2051.39071
4.57601	2433.58305	2526.5127	2037.4187
4.5676	2417.54083	2510.31298	2023.63748
4.55922	2401.74283	2494.33979	2010.04103
4.55087	2386.18558	2478.58995	1996.62332
4.54255	2370.86541	2463.06004	1983.37836
4.53426	2355.77848	2447.74646	1970.30019
4.526	2340.92079	2432.64537	1957.3829
4.51777	2326.28818	2417.75276	1944.62068
4.50958	2311.87633	2403.06446	1932.00783
4.50141	2297.68081	2388.57609	1919.53875
4.49327	2283.69705	2374.28313	1907.208
4.48516	2269.92037	2360.18093	1895.01026
4.47707	2256.34601	2346.26467	1882.94042

4.46902	2242.9691	2332.52945	1870.99353
4.461	2229.78469	2318.97024	1859.16483
4.453	2216.78779	2305.58192	1847.44979
4.44504	2203.97332	2292.35928	1835.84408
4.4371	2191.33617	2279.29705	1824.34359
4.42919	2178.87122	2266.38993	1812.94445
4.42131	2166.5733	2253.63256	1801.64304
4.41346	2154.43725	2241.01954	1790.43597
4.40563	2142.45788	2228.54551	1779.32009
4.39783	2130.63004	2216.20506	1768.29253
4.39006	2118.94859	2203.99285	1757.35064
4.38232	2107.40842	2191.90354	1746.49204
4.37461	2096.00446	2179.93186	1735.71458
4.36692	2084.73169	2168.07258	1725.01638
4.35926	2073.58515	2156.32057	1714.3958
4.35162	2062.55995	2144.67078	1703.85142
4.34401	2051.65128	2133.11826	1693.38207
4.33643	2040.8544	2121.65817	1682.98681
4.32888	2030.16467	2110.28583	1672.66492
4.32135	2019.57756	2098.99666	1662.41588
4.31385	2009.08863	2087.78626	1652.23938
4.30637	1998.69357	2076.65037	1642.1353
4.29892	1988.38818	2065.58493	1632.10371
4.2915	1978.16838	2054.58605	1622.14485
4.2841	1968.03023	2043.65001	1612.25912
4.27672	1957.96993	2032.77333	1602.44705
4.26937	1947.98381	2021.95271	1592.70934
4.26205	1938.06836	2011.18507	1583.04678
4.25475	1928.2202	2000.46756	1573.4603
4.24748	1918.43612	1989.79755	1563.9509

4.24023	1908.71305	1979.17264	1554.51968
4.23301	1899.04807	1968.59067	1545.16781
4.22581	1889.43845	1958.0497	1535.89651
4.21864	1879.88159	1947.54805	1526.70704
4.21148	1870.37507	1937.08428	1517.60072
4.20436	1860.91662	1926.65718	1508.57884
4.19726	1851.50414	1916.26579	1499.64275
4.19018	1842.13568	1905.90937	1490.79375
4.18312	1832.80946	1895.58745	1482.03314
4.17609	1823.52386	1885.29977	1473.36218
4.16909	1814.27743	1875.04631	1464.78211
4.1621	1805.06885	1864.82726	1456.2941
4.15514	1795.89696	1854.64306	1447.89926
4.14821	1786.76078	1844.49433	1439.59864
4.14129	1777.65945	1834.38193	1431.3932
4.1344	1768.59226	1824.30689	1423.28381
4.12754	1759.55864	1814.27045	1415.27125
4.12069	1750.55818	1804.27402	1407.3562
4.11387	1741.59058	1794.3192	1399.53924
4.10707	1732.65566	1784.40773	1391.82081
4.10029	1723.75341	1774.54152	1384.20127
4.09354	1714.88389	1764.72261	1376.68083
4.0868	1706.0473	1754.95316	1369.25957
4.08009	1697.24394	1745.23546	1361.93748
4.0734	1688.47422	1735.57191	1354.71439
4.06674	1679.73864	1725.96498	1347.59
4.06009	1671.0378	1716.41723	1340.5639
4.05347	1662.37239	1706.93129	1333.63554
4.04687	1653.74315	1697.50983	1326.80424
4.04029	1645.15093	1688.15556	1320.06919

4.03373	1636.59662	1678.87122	1313.42948
4.02719	1628.08119	1669.65955	1306.88405
4.02067	1619.60565	1660.5233	1300.43175
4.01418	1611.17107	1651.46519	1294.07129
4.0077	1602.77855	1642.48792	1287.80128
4.00125	1594.42923	1633.59415	1281.62025
3.99482	1586.12428	1624.78647	1275.52661
3.9884	1577.8649	1616.06741	1269.51869
3.98201	1569.65228	1607.43942	1263.59471
3.97564	1561.48764	1598.90485	1257.75285
3.96929	1553.37222	1590.46597	1251.99119
3.96296	1545.30722	1582.1249	1246.30776
3.95665	1537.29386	1573.88367	1240.70053
3.95036	1529.33334	1565.74414	1235.16741
3.94409	1521.42684	1557.70806	1229.70628
3.93784	1513.57551	1549.77701	1224.31497
3.93161	1505.78049	1541.95242	1218.99131
3.9254	1498.04286	1534.23552	1213.73309
3.91921	1490.36369	1526.62742	1208.53808
3.91303	1482.74398	1519.129	1203.40406
3.90688	1475.1847	1511.74099	1198.32882
3.90075	1467.68677	1504.46392	1193.31013
3.89463	1460.25105	1497.29811	1188.34581
3.88854	1452.87833	1490.24372	1183.43368
3.88246	1445.56936	1483.30068	1178.57159
3.87641	1438.32481	1476.46874	1173.75743
3.87037	1431.14529	1469.74745	1168.98913
3.86435	1424.03134	1463.13617	1164.26467
3.85835	1416.98343	1456.63404	1159.58208
3.85237	1410.00195	1450.24002	1154.93943

3.8464	1403.08723	1443.95289	1150.33486
3.84046	1396.2395	1437.77123	1145.76659
3.83453	1389.45894	1431.69344	1141.23288
3.82862	1382.74562	1425.71773	1136.73206
3.82273	1376.09957	1419.84215	1132.26256
3.81686	1369.52071	1414.0646	1127.82286
3.81101	1363.00889	1408.38278	1123.41152
3.80517	1356.5639	1402.79428	1119.02718
3.79935	1350.18542	1397.29653	1114.66855
3.79355	1343.87308	1391.88681	1110.33443
3.78777	1337.62642	1386.5623	1106.02369
3.782	1331.44492	1381.32005	1101.73527
3.77626	1325.32798	1376.15701	1097.46821
3.77053	1319.27491	1371.07004	1093.2216
3.76481	1313.28498	1366.05591	1088.99461
3.75912	1307.35738	1361.11131	1084.78651
3.75344	1301.49125	1356.23287	1080.59659
3.74778	1295.68564	1351.41719	1076.42424
3.74213	1289.93956	1346.6608	1072.26891
3.73651	1284.25198	1341.96022	1068.13012
3.7309	1278.62177	1337.31195	1064.00742
3.7253	1273.0478	1332.7125	1059.90045
3.71972	1267.52886	1328.15835	1055.80887
3.71416	1262.06372	1323.64605	1051.73241
3.70862	1256.6511	1319.17213	1047.67083
3.70309	1251.28967	1314.73318	1043.62395
3.69758	1245.9781	1310.32587	1039.5916
3.69209	1240.71501	1305.94689	1035.57366
3.68661	1235.49899	1301.59301	1031.57004
3.68115	1230.32863	1297.26112	1027.58067

3.6757	1225.20248	1292.94815	1023.60548
3.67027	1220.1191	1288.65117	1019.64446
3.66486	1215.07703	1284.36733	1015.69758
3.65946	1210.07479	1280.09393	1011.76483
3.65408	1205.11093	1275.82836	1007.84621
3.64872	1200.18398	1271.56818	1003.94171
3.64337	1195.29248	1267.31105	1000.05134
3.63803	1190.43498	1263.05481	996.17509
3.63271	1185.61005	1258.79741	992.31295
3.62741	1180.81627	1254.537	988.46491
3.62212	1176.05225	1250.27185	984.63093
3.61685	1171.31661	1246.00041	980.81098
3.61159	1166.60799	1241.7213	977.005
3.60635	1161.9251	1237.43328	973.21291
3.60112	1157.26664	1233.13531	969.43464
3.59591	1152.63137	1228.82649	965.67008
3.59072	1148.01808	1224.5061	961.9191
3.58554	1143.4256	1220.17359	958.18156
3.58037	1138.85281	1215.82858	954.4573
3.57522	1134.29864	1211.47084	950.74613
3.57008	1129.76205	1207.10031	947.04786
3.56496	1125.24206	1202.71708	943.36226
3.55985	1120.73776	1198.3214	939.6891
3.55476	1116.24826	1193.91368	936.02813
3.54968	1111.77274	1189.49447	932.37907
3.54462	1107.31045	1185.06444	928.74165
3.53957	1102.86068	1180.62442	925.11556
3.53453	1098.42278	1176.17535	921.50051
3.52951	1093.99615	1171.71831	917.89618
3.52451	1089.58028	1167.25448	914.30225

3.51951	1085.17469	1162.78514	910.71839
3.51453	1080.77897	1158.31169	907.14428
3.50957	1076.39277	1153.8356	903.57959
3.50462	1072.01578	1149.35843	900.02401
3.49968	1067.64779	1144.88182	896.4772
3.49476	1063.28862	1140.40746	892.93887
3.48985	1058.93814	1135.93712	889.40871
3.48496	1054.59629	1131.4726	885.88644
3.48008	1050.26307	1127.01574	882.37179
3.47521	1045.93853	1122.56841	878.8645
3.47036	1041.62277	1118.1325	875.36436
3.46552	1037.31593	1113.70991	871.87114
3.46069	1033.01823	1109.30256	868.38467
3.45588	1028.72992	1104.91233	864.9048
3.45108	1024.45128	1100.54113	861.43139
3.44629	1020.18266	1096.1908	857.96435
3.44152	1015.92445	1091.86319	854.50363
3.43676	1011.67707	1087.56008	851.04919
3.43201	1007.44097	1083.28322	847.60105
3.42728	1003.21666	1079.03431	844.15926
3.42256	999.00466	1074.81497	840.72389
3.41785	994.80554	1070.62678	837.29507
3.41315	990.61987	1066.47122	833.87296
3.40847	986.44829	1062.3497	830.45777
3.4038	982.29141	1058.26357	827.04975
3.39915	978.1499	1054.21405	823.64916
3.3945	974.02443	1050.2023	820.25634
3.38987	969.91568	1046.22936	816.87164
3.38525	965.82437	1042.29618	813.49547
3.38065	961.75119	1038.40361	810.12826

3.37605	957.69686	1034.55239	806.77048
3.37147	953.66211	1030.74314	803.42264
3.36691	949.64766	1026.97639	800.08527
3.36235	945.65422	1023.25255	796.75895
3.35781	941.68252	1019.57191	793.44428
3.35327	937.73326	1015.93467	790.14189
3.34875	933.80715	1012.34091	786.85241
3.34425	929.90488	1008.79058	783.57654
3.33975	926.02713	1005.28356	780.31496
3.33527	922.17455	1001.81959	777.06837
3.3308	918.34781	998.39833	773.83751
3.32634	914.54752	995.01932	770.62311
3.32189	910.77429	991.68203	767.4259
3.31746	907.0287	988.38579	764.24665
3.31303	903.3113	985.12989	761.08609
3.30862	899.62263	981.9135	757.94498
3.30422	895.96318	978.73572	754.82405
3.29984	892.33344	975.59557	751.72406
3.29546	888.73383	972.49201	748.64571
3.29109	885.16477	969.42391	745.58973
3.28674	881.62664	966.3901	742.55681
3.2824	878.11977	963.38935	739.54762
3.27807	874.64447	960.42037	736.56282
3.27375	871.20101	957.48184	733.60303
3.26944	867.78963	954.5724	730.66886
3.26515	864.41052	951.69066	727.76087
3.26086	861.06385	948.8352	724.8796
3.25659	857.74973	946.00457	722.02555
3.25232	854.46826	943.19734	719.19918
3.24807	851.21949	940.41205	716.40091

3.24383	848.00341	937.64724	713.63113
3.2396	844.82001	934.90147	710.89018
3.23539	841.66923	932.17329	708.17836
3.23118	838.55097	929.46129	705.49591
3.22698	835.46509	926.76407	702.84303
3.2228	832.41142	924.08027	700.21989
3.21862	829.38977	921.40855	697.6266
3.21446	826.39988	918.74761	695.06321
3.21031	823.44151	916.09621	692.52974
3.20616	820.51434	913.45314	690.02616
3.20203	817.61804	910.81725	687.55237
3.19791	814.75225	908.18744	685.10827
3.1938	811.91659	905.56267	682.69365
3.1897	809.11065	902.94199	680.30832
3.18561	806.33397	900.32446	677.95198
3.18153	803.58611	897.70927	675.62435
3.17746	800.86657	895.09562	673.32506
3.1734	798.17484	892.48284	671.05372
3.16936	795.51041	889.8703	668.80991
3.16532	792.87272	887.25744	666.59314
3.16129	790.26123	884.6438	664.40291
3.15728	787.67534	882.02898	662.2387
3.15327	785.11448	879.41265	660.09993
3.14927	782.57805	876.79458	657.98601
3.14529	780.06544	874.1746	655.89631
3.14131	777.57603	871.55262	653.83019
3.13734	775.10921	868.92861	651.78698
3.13339	772.66433	866.30262	649.76601
3.12944	770.24078	863.67479	647.76656
3.1255	767.83792	861.04529	645.78794

3.12158	765.45512	858.41439	643.82941
3.11766	763.09174	855.7824	641.89024
3.11375	760.74716	853.1497	639.9697
3.10986	758.42075	850.51674	638.06705
3.10597	756.1119	847.88399	636.18155
3.10209	753.81998	845.252	634.31247
3.09822	751.54441	842.62136	632.45907
3.09437	749.28459	839.99269	630.62064
3.09052	747.03992	837.36666	628.79645
3.08668	744.80985	834.74397	626.98581
3.08285	742.5938	832.12536	625.18802
3.07903	740.39124	829.51158	623.40242
3.07522	738.20163	826.90342	621.62835
3.07142	736.02446	824.30167	619.86517
3.06762	733.85923	821.70715	618.11226
3.06384	731.70545	819.12067	616.36904
3.06007	729.56267	816.54305	614.63493
3.05631	727.43043	813.97513	612.90939
3.05255	725.3083	811.41772	611.19189
3.04881	723.19587	808.87163	609.48195
3.04507	721.09276	806.33767	607.77909
3.04134	718.99859	803.81661	606.08288
3.03762	716.91301	801.30923	604.39291
3.03391	714.83569	798.81625	602.70879
3.03021	712.76632	796.33839	601.03016
3.02652	710.7046	793.87634	599.3567
3.02284	708.65027	791.43074	597.68811
3.01917	706.60306	789.0022	596.02411
3.0155	704.56275	786.5913	594.36447
3.01185	702.52911	784.19856	592.70896

3.0082	700.50196	781.82448	591.05739
3.00457	698.48112	779.46949	589.4096
3.00094	696.46642	777.13398	587.76543
2.99732	694.45773	774.81831	586.12478
2.99371	692.45491	772.52275	584.48754
2.9901	690.45786	770.24755	582.85363
2.98651	688.46649	767.9929	581.223
2.98292	686.48071	765.75894	579.59561
2.97935	684.50046	763.54573	577.97144
2.97578	682.5257	761.35331	576.35047
2.97222	680.55638	759.18166	574.73273
2.96867	678.59247	757.03069	573.11823
2.96513	676.63397	754.90026	571.50701
2.96159	674.68087	752.79021	569.8991
2.95807	672.73317	750.70028	568.29457
2.95455	670.79089	748.63021	566.69347
2.95104	668.85406	746.57966	565.09587
2.94754	666.9227	744.54825	563.50184
2.94405	664.99685	742.53556	561.91146
2.94056	663.07655	740.54113	560.32479
2.93709	661.16186	738.56447	558.74194
2.93362	659.25281	736.60503	557.16296
2.93016	657.34947	734.66223	555.58794
2.92671	655.4519	732.73549	554.01695
2.92327	653.56015	730.82415	552.45007
2.91983	651.67429	728.92757	550.88737
2.9164	649.79437	727.04507	549.32891
2.91298	647.92046	725.17593	547.77475
2.90957	646.05261	723.31945	546.22495
2.90617	644.19089	721.47489	544.67955

2.90278	642.33536	719.6415	543.13859
2.89939	640.48605	717.81854	541.60212
2.89601	638.64304	716.00527	540.07015
2.89264	636.80635	714.20091	538.54271
2.88927	634.97605	712.40472	537.01982
2.88592	633.15216	710.61596	535.50147
2.88257	631.33473	708.83389	533.98766
2.87923	629.52377	707.05778	532.4784
2.8759	627.71933	705.28692	530.97366
2.87257	625.92141	703.52062	529.47342
2.86926	624.13003	701.75822	527.97766
2.86595	622.3452	699.99905	526.48634
2.86265	620.56693	698.24249	524.99942
2.85935	618.7952	696.48796	523.51687
2.85606	617.03001	694.73488	522.03864
2.85279	615.27135	692.98272	520.56468
2.84951	613.5192	691.23098	519.09494
2.84625	611.77353	689.4792	517.62937
2.84299	610.03431	687.72694	516.16791
2.83974	608.30151	685.97383	514.71051
2.8365	606.57507	684.21952	513.25711
2.83327	604.85497	682.4637	511.80767
2.83004	603.14115	680.7061	510.36213
2.82682	601.43355	678.94651	508.92045
2.82361	599.73212	677.18475	507.48259
2.8204	598.03679	675.42068	506.04849
2.81721	596.34751	673.65421	504.61814
2.81402	594.6642	671.88529	503.19149
2.81083	592.9868	670.11392	501.76853
2.80766	591.31523	668.34014	500.34924

2.80449	589.64942	666.56403	498.93361
2.80133	587.98931	664.7857	497.52163
2.79817	586.33481	663.00533	496.11332
2.79502	584.68585	661.2231	494.70868
2.79188	583.04237	659.43927	493.30775
2.78875	581.40428	657.6541	491.91055
2.78562	579.77151	655.8679	490.51712
2.7825	578.144	654.08102	489.12752
2.77939	576.52169	652.29383	487.7418
2.77629	574.90449	650.50673	486.36003
2.77319	573.29237	648.72015	484.98228
2.7701	571.68524	646.93456	483.60866
2.76701	570.08308	645.15041	482.23924
2.76393	568.48581	643.36822	480.87413
2.76086	566.8934	641.58849	479.51345
2.7578	565.30582	639.81176	478.15731
2.75474	563.72301	638.03857	476.80584
2.75169	562.14496	636.26947	475.45917
2.74865	560.57165	634.50502	474.11744
2.74561	559.00304	632.74579	472.78079
2.74258	557.43914	630.99234	471.44938
2.73955	555.87994	629.24525	470.12335
2.73654	554.32544	627.50506	468.80286
2.73353	552.77565	625.77235	467.48808
2.73052	551.23058	624.04767	466.17916
2.72753	549.69025	622.33154	464.87626
2.72454	548.1547	620.62451	463.57955
2.72155	546.62396	618.92708	462.28919
2.71857	545.09807	617.23974	461.00534
2.7156	543.57709	615.56297	459.72815

2.71264	542.06105	613.89722	458.45778
2.70968	540.55004	612.24292	457.19439
2.70673	539.0441	610.60047	455.93812
2.70378	537.54333	608.97025	454.68911
2.70084	536.04779	607.3526	453.44749
2.69791	534.55758	605.74784	452.2134
2.69498	533.07277	604.15626	450.98696
2.69207	531.59347	602.5781	449.76826
2.68915	530.11977	601.01357	448.55743
2.68624	528.65178	599.46288	447.35456
2.68334	527.1896	597.92615	446.15972
2.68045	525.73335	596.4035	444.97299
2.67756	524.28312	594.895	443.79443
2.67468	522.83905	593.40068	442.62411
2.6718	521.40123	591.92055	441.46204
2.66893	519.9698	590.45456	440.30827
2.66607	518.54487	589.00265	439.16281
2.66321	517.12655	587.56469	438.02566
2.66036	515.71497	586.14054	436.89682
2.65751	514.31025	584.73001	435.77625
2.65468	512.91248	583.33288	434.66394
2.65184	511.5218	581.9489	433.55984
2.64902	510.13832	580.57778	432.46388
2.64619	508.76213	579.21921	431.376
2.64338	507.39335	577.87284	430.29611
2.64057	506.03207	576.53828	429.22413
2.63777	504.6784	575.21514	428.15996
2.63497	503.33242	573.90298	427.10347
2.63218	501.99422	572.60134	426.05454
2.62939	500.66388	571.30976	425.01305

2.62661	499.34147	570.02774	423.97884
2.62384	498.02705	568.75475	422.95176
2.62107	496.72069	567.49028	421.93166
2.61831	495.42244	566.23376	420.91837
2.61555	494.13234	564.98465	419.91171
2.6128	492.85043	563.74238	418.91151
2.61006	491.57672	562.50638	417.91758
2.60732	490.31124	561.27606	416.92972
2.60459	489.054	560.05084	415.94774
2.60186	487.80499	558.83014	414.97145
2.59914	486.5642	557.61339	414.00064
2.59642	485.33161	556.39999	413.0351
2.59371	484.10719	555.18939	412.07464
2.59101	482.89089	553.98102	411.11905
2.58831	481.68267	552.77432	410.16812
2.58562	480.48247	551.56876	409.22165
2.58293	479.2902	550.36381	408.27942
2.58025	478.10579	549.15897	407.34125
2.57757	476.92915	547.95374	406.40692
2.5749	475.76017	546.74765	405.47624
2.57223	474.59875	545.54026	404.54901
2.56957	473.44477	544.33115	403.62504
2.56692	472.29808	543.11991	402.70416
2.56427	471.15856	541.90617	401.78616
2.56162	470.02606	540.68959	400.87088
2.55899	468.90041	539.46986	399.95815
2.55635	467.78147	538.24668	399.04779
2.55373	466.66905	537.01979	398.13965
2.5511	465.56297	535.78899	397.23357
2.54849	464.46306	534.55408	396.3294

2.54588	463.36912	533.31489	395.427
2.54327	462.28096	532.0713	394.52625
2.54067	461.19836	530.82323	393.627
2.53808	460.12114	529.57061	392.72913
2.53549	459.04906	528.31341	391.83254
2.5329	457.98193	527.05166	390.93711
2.53032	456.91952	525.78537	390.04275
2.52775	455.86162	524.51464	389.14935
2.52518	454.808	523.23957	388.25683
2.52262	453.75844	521.96028	387.36511
2.52006	452.71273	520.67696	386.47412
2.5175	451.67064	519.38979	385.58378
2.51496	450.63194	518.09899	384.69403
2.51241	449.59643	516.80483	383.80482
2.50987	448.56388	515.50757	382.9161
2.50734	447.53409	514.20753	382.02782
2.50481	446.50683	512.90501	381.13994
2.50229	445.48192	511.60037	380.25243
2.49977	444.45914	510.29398	379.36526
2.49726	443.4383	508.98621	378.4784
2.49476	442.41922	507.67748	377.59185
2.49225	441.4017	506.36818	376.70557
2.48976	440.38558	505.05875	375.81957
2.48726	439.37069	503.74963	374.93383
2.48478	438.35688	502.44126	374.04836
2.48229	437.34398	501.1341	373.16316
2.47982	436.33186	499.82859	372.27823
2.47734	435.32039	498.52522	371.39359
2.47488	434.30944	497.22443	370.50924
2.47241	433.29892	495.92668	369.6252

2.46996	432.28871	494.63244	368.7415
2.4675	431.27873	493.34216	367.85816
2.46506	430.2689	492.05628	366.9752
2.46261	429.25916	490.77524	366.09266
2.46017	428.24945	489.49948	365.21057
2.45774	427.23973	488.22939	364.32897
2.45531	426.22997	486.96539	363.4479
2.45289	425.22016	485.70787	362.56739
2.45047	424.21029	484.45719	361.68751
2.44806	423.20036	483.21371	360.80829
2.44565	422.19041	481.97775	359.9298
2.44324	421.18045	480.74964	359.05208
2.44084	420.17053	479.52967	358.17521
2.43845	419.16071	478.3181	357.29925
2.43605	418.15106	477.11518	356.42426
2.43367	417.14166	475.92113	355.55032
2.43129	416.13259	474.73615	354.6775
2.42891	415.12395	473.56041	353.80588
2.42654	414.11587	472.39406	352.93555
2.42417	413.10846	471.23722	352.0666
2.42181	412.10186	470.08998	351.19911
2.41945	411.0962	468.95242	350.33318
2.4171	410.09164	467.82457	349.4689
2.41475	409.08834	466.70646	348.6064
2.4124	408.08647	465.59808	347.74576
2.41006	407.08619	464.49939	346.8871
2.40773	406.0877	463.41034	346.03054
2.4054	405.09118	462.33085	345.17619
2.40307	404.09682	461.26081	344.32417
2.40075	403.10482	460.2001	343.47461

2.39843	402.11539	459.14858	342.62764
2.39612	401.12874	458.10607	341.78338
2.39381	400.14507	457.07239	340.94197
2.39151	399.16459	456.04735	340.10355
2.38921	398.18753	455.03072	339.26825
2.38691	397.21409	454.02226	338.43621
2.38462	396.2445	453.02173	337.60757
2.38234	395.27896	452.02887	336.78247
2.38005	394.3177	451.0434	335.96105
2.37778	393.36092	450.06506	335.14346
2.3755	392.40884	449.09354	334.32984
2.37323	391.46166	448.12856	333.52032
2.37097	390.51958	447.16982	332.71505
2.36871	389.58281	446.21701	331.91416
2.36645	388.65153	445.26984	331.11779
2.3642	387.72594	444.32799	330.32607
2.36195	386.80621	443.39117	329.53913
2.35971	385.89252	442.45908	328.75709
2.35747	384.98504	441.53143	327.98007
2.35524	384.08392	440.60792	327.20819
2.35301	383.18932	439.68827	326.44156
2.35078	382.30137	438.77222	325.68028
2.34856	381.42021	437.8595	324.92445
2.34634	380.54596	436.94985	324.17415
2.34413	379.67872	436.04305	323.42947
2.34192	378.81859	435.13885	322.69049
2.33971	377.96568	434.23705	321.95725
2.33751	377.12004	433.33745	321.22983
2.33532	376.28175	432.43987	320.50827
2.33312	375.45086	431.54414	319.7926

2.33093	374.62741	430.6501	319.08285
2.32875	373.81143	429.75763	318.37904
2.32657	373.00293	428.86661	317.68117
2.32439	372.20191	427.97695	316.98923
2.32222	371.40838	427.08855	316.3032
2.32005	370.6223	426.20137	315.62307
2.31789	369.84363	425.31535	314.94878
2.31573	369.07234	424.43046	314.28029
2.31357	368.30836	423.54671	313.61753
2.31142	367.55163	422.66409	312.96043
2.30927	366.80204	421.78262	312.30891
2.30713	366.05952	420.90236	311.66286
2.30499	365.32394	420.02334	311.02218
2.30285	364.5952	419.14565	310.38676
2.30072	363.87316	418.26936	309.75646
2.29859	363.15767	417.39457	309.13115
2.29647	362.4486	416.52138	308.51069
2.29435	361.74577	415.64992	307.8949
2.29223	361.04902	414.78031	307.28365
2.29012	360.35816	413.91269	306.67674
2.28801	359.67301	413.0472	306.07401
2.2859	358.99337	412.184	305.47526
2.2838	358.31904	411.32324	304.88031
2.2817	357.64981	410.46507	304.28896
2.27961	356.98546	409.60966	303.70102
2.27752	356.32577	408.75718	303.11627
2.27544	355.67051	407.90778	302.53451
2.27335	355.01945	407.06163	301.95554
2.27128	354.37237	406.21889	301.37914
2.2692	353.72901	405.37971	300.80511

2.26713	353.08914	404.54425	300.23323
2.26506	352.45252	403.71265	299.66331
2.263	351.8189	402.88505	299.09513
2.26094	351.18805	402.06157	298.52849
2.25889	350.55972	401.24234	297.9632
2.25684	349.93367	400.42748	297.39906
2.25479	349.30966	399.61707	296.83589
2.25274	348.68745	398.81121	296.27351
2.2507	348.06681	398.00997	295.71173
2.24867	347.44752	397.21342	295.15041
2.24663	346.82934	396.42161	294.58937
2.2446	346.21205	395.63456	294.02847
2.24258	345.59545	394.8523	293.46757
2.24056	344.97932	394.07484	292.90655
2.23854	344.36346	393.30216	292.34528
2.23652	343.74768	392.53424	291.78366
2.23451	343.13179	391.77105	291.22159
2.2325	342.51562	391.01252	290.65899
2.2305	341.89899	390.2586	290.09578
2.2285	341.28175	389.50919	289.53191
2.2265	340.66375	388.7642	288.96733
2.22451	340.04484	388.02352	288.402
2.22252	339.42491	387.28702	287.8359
2.22053	338.80383	386.55457	287.26902
2.21855	338.18149	385.82603	286.70135
2.21657	337.5578	385.10123	286.13292
2.2146	336.93269	384.38	285.56374
2.21262	336.30606	383.66218	284.99386
2.21065	335.67787	382.94757	284.42331
2.20869	335.04807	382.23599	283.85216

2.20673	334.41662	381.52723	283.28046
2.20477	333.7835	380.82111	282.70831
2.20282	333.1487	380.1174	282.13578
2.20086	332.51221	379.41591	281.56296
2.19892	331.87406	378.71644	280.98996
2.19697	331.23427	378.01876	280.41689
2.19503	330.59287	377.32268	279.84385
2.19309	329.94991	376.628	279.27097
2.19116	329.30546	375.93452	278.69838
2.18923	328.65959	375.24205	278.12619
2.1873	328.01237	374.55039	277.55455
2.18538	327.36391	373.85939	276.98358
2.18346	326.7143	373.16886	276.41341
2.18154	326.06366	372.47864	275.84419
2.17963	325.41211	371.78861	275.27605
2.17772	324.75979	371.0986	274.70913
2.17581	324.10683	370.40852	274.14354
2.17391	323.45339	369.71825	273.57944
2.17201	322.79962	369.02769	273.01693
2.17011	322.14568	368.33678	272.45615
2.16822	321.49175	367.64545	271.89721
2.16633	320.83801	366.95365	271.34022
2.16444	320.18463	366.26137	270.7853
2.16256	319.5318	365.56858	270.23253
2.16067	318.87972	364.87531	269.68203
2.1588	318.22857	364.18157	269.13387
2.15692	317.57856	363.48741	268.58813
2.15505	316.92989	362.79289	268.04489
2.15319	316.28276	362.09809	267.5042
2.15132	315.63737	361.4031	266.96612

2.14946	314.99392	360.70804	266.43071
2.1476	314.35262	360.01304	265.89798
2.14575	313.71367	359.31823	265.36799
2.1439	313.07728	358.62379	264.84074
2.14205	312.44363	357.92989	264.31624
2.1402	311.81292	357.2367	263.79451
2.13836	311.18535	356.54444	263.27553
2.13652	310.56111	355.8533	262.75929
2.13469	309.94037	355.16352	262.24578
2.13286	309.32332	354.47532	261.73497
2.13103	308.71012	353.78894	261.22681
2.1292	308.10094	353.10462	260.72128
2.12738	307.49595	352.42261	260.21833
2.12556	306.89528	351.74316	259.7179
2.12374	306.29908	351.06653	259.21994
2.12193	305.70749	350.39297	258.72438
2.12012	305.12062	349.72273	258.23118
2.11831	304.53861	349.05606	257.74025
2.1165	303.96154	348.39321	257.25153
2.1147	303.38952	347.73443	256.76495
2.1129	302.82263	347.07994	256.28044
2.11111	302.26094	346.42998	255.79793
2.10932	301.70451	345.78475	255.31735
2.10753	301.15341	345.14447	254.83862
2.10574	300.60766	344.50933	254.36169
2.10396	300.06729	343.87949	253.88648
2.10218	299.53232	343.25514	253.41294
2.1004	299.00276	342.63641	252.941
2.09863	298.47859	342.02343	252.47061
2.09686	297.95979	341.41632	252.00172

2.09509	297.44633	340.81517	251.53428
2.09332	296.93816	340.22004	251.06826
2.09156	296.43523	339.631	250.60362
2.0898	295.93747	339.04806	250.14033
2.08805	295.44479	338.47124	249.67838
2.08629	294.9571	337.90052	249.21774
2.08454	294.4743	337.33586	248.7584
2.0828	293.99627	336.77721	248.30036
2.08105	293.52289	336.22448	247.84364
2.07931	293.05401	335.67757	247.38823
2.07757	292.58949	335.13634	246.93415
2.07584	292.12917	334.60065	246.48143
2.0741	291.67288	334.07033	246.0301
2.07237	291.22044	333.54518	245.58019
2.07065	290.77168	333.02499	245.13173
2.06892	290.32638	332.50953	244.68478
2.0672	289.88436	331.99855	244.23938
2.06548	289.44541	331.49177	243.79558
2.06377	289.0093	330.98892	243.35344
2.06205	288.57582	330.48969	242.91302
2.06035	288.14473	329.99376	242.47437
2.05864	287.71582	329.50081	242.03756
2.05693	287.28883	329.01051	241.60265
2.05523	286.86353	328.52249	241.16969
2.05353	286.43968	328.03642	240.73875
2.05184	286.01703	327.55191	240.30989
2.05015	285.59533	327.06861	239.88315
2.04846	285.17434	326.58615	239.45859
2.04677	284.75381	326.10415	239.03627
2.04508	284.33348	325.62224	238.61621

2.0434	283.91312	325.14005	238.19846
2.04172	283.49248	324.65722	237.78304
2.04005	283.07131	324.17339	237.36998
2.03837	282.64938	323.68821	236.9593
2.0367	282.22645	323.20133	236.55099
2.03503	281.80229	322.71242	236.14506
2.03337	281.37668	322.22117	235.74149
2.03171	280.9494	321.72726	235.34025
2.03005	280.52022	321.23041	234.94133
2.02839	280.08896	320.73034	234.54466
2.02673	279.65541	320.2268	234.1502
2.02508	279.21938	319.71956	233.75788
2.02343	278.78069	319.20839	233.36762
2.02179	278.33917	318.69311	232.97933
2.02014	277.89466	318.17354	232.5929
2.0185	277.44701	317.64954	232.20823
2.01686	276.99608	317.12098	231.82519
2.01523	276.54175	316.58777	231.44365
2.01359	276.08389	316.04983	231.06345
2.01196	275.62242	315.50712	230.68445
2.01034	275.15723	314.9596	230.30648
2.00871	274.68824	314.40729	229.92936
2.00709	274.21541	313.85022	229.55292
2.00547	273.73867	313.28843	229.17695
2.00385	273.25798	312.722	228.80128
2.00224	272.77334	312.15105	228.42569
2.00062	272.28473	311.5757	228.04998
1.99902	271.79215	310.99609	227.67394
1.99741	271.29564	310.41241	227.29735
1.9958	270.79521	309.82484	226.92001

1.9942	270.29093	309.23361	226.5417
1.9926	269.78285	308.63895	226.1622
1.99101	269.27107	308.04111	225.78132
1.98941	268.75566	307.44036	225.39883
1.98782	268.23674	306.83699	225.01455
1.98623	267.71442	306.23129	224.62828
1.98465	267.18885	305.62357	224.23982
1.98306	266.66017	305.01416	223.849
1.98148	266.12853	304.40339	223.45566
1.9799	265.59412	303.79159	223.05963
1.97832	265.05712	303.1791	222.66078
1.97675	264.51773	302.56628	222.25897
1.97518	263.97615	301.95346	221.8541
1.97361	263.43261	301.341	221.44605
1.97204	262.88733	300.72924	221.03474
1.97048	262.34055	300.11853	220.62013
1.96892	261.79253	299.50919	220.20215
1.96736	261.24352	298.90156	219.78079
1.9658	260.69379	298.29596	219.35603
1.96425	260.14361	297.69271	218.9279
1.9627	259.59325	297.09208	218.49642
1.96115	259.043	296.49439	218.06166
1.9596	258.49316	295.89988	217.6237
1.95806	257.94401	295.30882	217.18263
1.95652	257.39585	294.72145	216.73858
1.95498	256.84898	294.13797	216.29169
1.95344	256.3037	293.5586	215.84212
1.95191	255.76032	292.98351	215.39007
1.95037	255.21913	292.41286	214.93573
1.94884	254.68044	291.84679	214.47933

1.94732	254.14454	291.2854	214.02111
1.94579	253.61174	290.7288	213.56134
1.94427	253.08232	290.17705	213.10029
1.94275	252.55658	289.63019	212.63825
1.94123	252.03479	289.08826	212.17554
1.93972	251.51724	288.55126	211.71247
1.9382	251.00419	288.01916	211.24936
1.93669	250.49591	287.49193	210.78658
1.93518	249.99266	286.96949	210.32445
1.93368	249.49467	286.45176	209.86334
1.93217	249.00218	285.93865	209.40361
1.93067	248.51542	285.43003	208.94562
1.92917	248.03459	284.92576	208.48973
1.92768	247.55991	284.42567	208.0363
1.92618	247.09154	283.92961	207.5857
1.92469	246.62966	283.43737	207.13827
1.9232	246.17444	282.94877	206.69437
1.92171	245.72602	282.4636	206.25433
1.92023	245.28451	281.98162	205.81847
1.91875	244.85004	281.50262	205.38711
1.91727	244.42269	281.02635	204.96055
1.91579	244.00255	280.55258	204.53907
1.91431	243.58967	280.08107	204.12293
1.91284	243.18409	279.61156	203.71238
1.91137	242.78584	279.14382	203.30764
1.9099	242.39493	278.6776	202.90889
1.90843	242.01133	278.21265	202.51633
1.90697	241.63502	277.74876	202.13008
1.9055	241.26595	277.28568	201.75029
1.90404	240.90404	276.82319	201.37702

1.90259	240.54921	276.3611	201.01035
1.90113	240.20134	275.89919	200.65031
1.89968	239.86031	275.43729	200.29691
1.89822	239.52598	274.97521	199.9501
1.89678	239.19818	274.5128	199.60984
1.89533	238.87672	274.04992	199.27604
1.89388	238.56141	273.58644	198.94856
1.89244	238.25203	273.12225	198.62727
1.891	237.94834	272.65727	198.31197
1.88956	237.65009	272.19142	198.00246
1.88813	237.35702	271.72465	197.6985
1.88669	237.06884	271.25693	197.39981
1.88526	236.78526	270.78825	197.10611
1.88383	236.50595	270.31861	196.81707
1.88241	236.23061	269.84805	196.53236
1.88098	235.95888	269.37662	196.2516
1.87956	235.69042	268.90437	195.97441
1.87814	235.42488	268.4314	195.7004
1.87672	235.16187	267.9578	195.42913
1.8753	234.90103	267.48371	195.16018
1.87389	234.64196	267.00925	194.8931
1.87248	234.38428	266.53458	194.62744
1.87107	234.12757	266.05987	194.36274
1.86966	233.87145	265.5853	194.09853
1.86825	233.6155	265.11106	193.83435
1.86685	233.35931	264.63735	193.56974
1.86545	233.10247	264.16438	193.30423
1.86405	232.84458	263.69238	193.03737
1.86265	232.58521	263.22157	192.76872
1.86126	232.32397	262.75216	192.49784

1.85986	232.06045	262.2844	192.22433
1.85847	231.79426	261.81852	191.94777
1.85708	231.525	261.35474	191.66779
1.8557	231.25228	260.89329	191.38403
1.85431	230.97574	260.43439	191.09616
1.85293	230.69501	259.97825	190.80388
1.85155	230.40972	259.52507	190.50691
1.85017	230.11955	259.07506	190.20501
1.84879	229.82416	258.62839	189.89796
1.84742	229.52324	258.18523	189.58559
1.84604	229.21648	257.74573	189.26777
1.84467	228.90362	257.31003	188.94439
1.84331	228.58439	256.87826	188.61539
1.84194	228.25853	256.45049	188.28075
1.84057	227.92584	256.02682	187.94049
1.83921	227.5861	255.6073	187.59467
1.83785	227.23914	255.19195	187.24339
1.83649	226.88479	254.7808	186.88679
1.83514	226.52293	254.37383	186.52507
1.83378	226.15343	253.97099	186.15844
1.83243	225.77623	253.57223	185.78718
1.83108	225.39124	253.17745	185.41158
1.82973	224.99845	252.78654	185.03201
1.82839	224.59784	252.39936	184.64883
1.82704	224.18943	252.01574	184.26247
1.8257	223.77326	251.63551	183.87338
1.82436	223.34942	251.25844	183.48204
1.82302	222.91799	250.88431	183.08897
1.82168	222.47911	250.51285	182.69471
1.82035	222.03293	250.1438	182.29982

1.81902	221.57963	249.77685	181.90488
1.81769	221.11943	249.4117	181.51052
1.81636	220.65256	249.04801	181.11734
1.81503	220.17928	248.68544	180.72598
1.81371	219.69988	248.32363	180.3371
1.81238	219.21468	247.96223	179.95133
1.81106	218.72401	247.60084	179.56933
1.80974	218.22824	247.2391	179.19177
1.80843	217.72776	246.87661	178.81927
1.80711	217.22297	246.51299	178.4525
1.8058	216.71431	246.14785	178.09207
1.80449	216.20223	245.78081	177.7386
1.80318	215.68719	245.41149	177.39268
1.80187	215.16969	245.03951	177.0549
1.80056	214.65024	244.66453	176.72581
1.79926	214.12935	244.2862	176.40592
1.79796	213.60756	243.90417	176.09572
1.79666	213.08541	243.51815	175.79567
1.79536	212.56348	243.12784	175.5062
1.79406	212.04232	242.73296	175.22768
1.79277	211.52252	242.33328	174.96044
1.79148	211.00465	241.92856	174.70479
1.79018	210.48931	241.51864	174.46097
1.7889	209.97708	241.10333	174.22917
1.78761	209.46855	240.68253	174.00955
1.78632	208.96432	240.25612	173.80221
1.78504	208.46497	239.82406	173.6072
1.78376	207.97107	239.38633	173.42451
1.78248	207.48322	238.94293	173.25408
1.7812	207.00196	238.49393	173.09581

1.77993	206.52786	238.03941	172.94953
1.77865	206.06146	237.5795	172.81504
1.77738	205.60328	237.11438	172.69205
1.77611	205.15384	236.64425	172.58027
1.77484	204.71363	236.16937	172.47933
1.77357	204.28312	235.69003	172.38881
1.77231	203.86276	235.20653	172.30827
1.77104	203.45297	234.71925	172.23721
1.76978	203.05416	234.22859	172.17508
1.76852	202.66669	233.73497	172.12132
1.76727	202.29091	233.23886	172.07532
1.76601	201.92714	232.74074	172.03644
1.76476	201.57564	232.24115	172.004
1.7635	201.23666	231.74062	171.97733
1.76225	200.91042	231.23973	171.95571
1.761	200.59708	230.73908	171.93841
1.75976	200.29679	230.23926	171.9247
1.75851	200.00964	229.7409	171.91383
1.75727	199.73568	229.24464	171.90504
1.75603	199.47493	228.75112	171.89759
1.75479	199.22737	228.26099	171.89073
1.75355	198.99293	227.7749	171.88373
1.75231	198.77149	227.2935	171.87587
1.75108	198.5629	226.81743	171.86643
1.74984	198.36696	226.34732	171.85475
1.74861	198.18344	225.8838	171.84016
1.74738	198.01204	225.42746	171.82204
1.74615	197.85244	224.97888	171.7998
1.74493	197.70427	224.53863	171.77288
1.7437	197.5671	224.10722	171.74077

1.74248	197.44048	223.68517	171.703
1.74126	197.32391	223.27293	171.65915
1.74004	197.21686	222.87091	171.60885
1.73882	197.11873	222.47952	171.55178
1.73761	197.02891	222.09908	171.48767
1.73639	196.94674	221.72989	171.41631
1.73518	196.87153	221.37217	171.33755
1.73397	196.80254	221.02613	171.2513
1.73276	196.73903	220.6919	171.15752
1.73155	196.68018	220.36955	171.05624
1.73035	196.62519	220.05909	170.94754
1.72914	196.5732	219.76049	170.83157
1.72794	196.52334	219.47365	170.70853
1.72674	196.47471	219.1984	170.57868
1.72554	196.4264	218.93451	170.44233
1.72434	196.37746	218.68169	170.29986
1.72315	196.32696	218.4396	170.15168
1.72195	196.27392	218.20782	169.99825
1.72076	196.21738	217.98588	169.8401
1.71957	196.15636	217.77324	169.67778
1.71838	196.08988	217.56931	169.51187
1.71719	196.01696	217.37345	169.34301
1.71601	195.93663	217.18494	169.17186
1.71482	195.8479	217.00305	168.99909
1.71364	195.74983	216.82696	168.8254
1.71246	195.64145	216.65583	168.65152
1.71128	195.52185	216.48876	168.47817
1.7101	195.39011	216.32482	168.30607
1.70892	195.24533	216.16306	168.13597
1.70775	195.08666	216.00247	167.96857

1.70658	194.91327	215.84204	167.80459
1.70541	194.72435	215.68072	167.64472
1.70424	194.51915	215.51745	167.48962
1.70307	194.29694	215.35117	167.33993
1.7019	194.05704	215.1808	167.19625
1.70074	193.79882	215.00527	167.05914
1.69957	193.5217	214.82351	166.9291
1.69841	193.22516	214.63445	166.80659
1.69725	192.9087	214.43707	166.69203
1.69609	192.57193	214.23033	166.58573
1.69494	192.21449	214.01324	166.48799
1.69378	191.83607	213.78486	166.399
1.69263	191.43648	213.54426	166.31888
1.69147	191.01553	213.29057	166.24769
1.69032	190.57317	213.02297	166.1854
1.68917	190.10936	212.74069	166.13188
1.68803	189.62417	212.44303	166.08695
1.68688	189.11775	212.12935	166.05032
1.68574	188.59032	211.79909	166.0216
1.68459	188.04215	211.45176	166.00034
1.68345	187.47365	211.08694	165.98598
1.68231	186.88525	210.7043	165.97789
1.68117	186.2775	210.30362	165.97533
1.68004	185.65103	209.88473	165.97749
1.6789	185.00652	209.44759	165.98348
1.67777	184.34478	208.99224	165.99232
1.67664	183.66665	208.51882	166.00296
1.67551	182.9731	208.02756	166.01428
1.67438	182.26513	207.51882	166.02508
1.67325	181.54387	206.99303	166.03412

1.67212	180.81049	206.45074	166.04008
1.671	180.06624	205.89259	166.0416
1.66988	179.31247	205.31935	166.03728
1.66875	178.55056	204.73185	166.02568
1.66763	177.782	204.13105	166.00533
1.66652	177.00832	203.51799	165.97474
1.6654	176.23113	202.8938	165.93242
1.66428	175.45207	202.25973	165.87685
1.66317	174.67287	201.61707	165.80654
1.66206	173.8953	200.96724	165.72001
1.66095	173.12118	200.3117	165.61581
1.65984	172.35236	199.652	165.49251
1.65873	171.59075	198.98976	165.34874
1.65762	170.83828	198.32665	165.18317
1.65652	170.09692	197.6644	164.99455
1.65541	169.36866	197.00479	164.78171
1.65431	168.65551	196.34964	164.54354
1.65321	167.95948	195.70078	164.27904
1.65211	167.28261	195.06011	163.98732
1.65101	166.62694	194.42949	163.6676
1.64992	165.99448	193.81084	163.3192
1.64882	165.38726	193.20604	162.9416
1.64773	164.80726	192.61698	162.53441
1.64664	164.25648	192.04552	162.09738
1.64555	163.73684	191.4935	161.63041
1.64446	163.25026	190.96271	161.13357
1.64337	162.7986	190.45489	160.6071
1.64228	162.38367	189.97175	160.05139
1.6412	162.00723	189.51488	159.46703
1.64012	161.67095	189.08585	158.85475

1.63903	161.37646	188.68611	158.21551
1.63795	161.12529	188.317	157.55041
1.63687	160.91889	187.9798	156.86076
1.6358	160.75862	187.67563	156.14803
1.63472	160.64573	187.4055	155.41391
1.63365	160.58137	187.17031	154.66022
1.63257	160.56658	186.97078	153.88899
1.6315	160.60226	186.80751	153.10242
1.63043	160.68922	186.68093	152.30287
1.62936	160.82809	186.59131	151.49286
1.62829	161.0194	186.53874	150.67508
1.62723	161.26352	186.52315	149.85234
1.62616	161.56065	186.54426	149.02762
1.6251	161.91087	186.60163	148.204
1.62404	162.31406	186.69461	147.38468
1.62298	162.76997	186.82234	146.57298
1.62192	163.27814	186.98378	145.77229
1.62086	163.83796	187.17766	144.98609
1.6198	164.44862	187.40253	144.21791
1.61875	165.10915	187.65671	143.47134
1.61769	165.81837	187.9383	142.74999
1.61664	166.57491	188.24521	142.05748
1.61559	167.37722	188.57512	141.39744
1.61454	168.22353	188.9255	140.77345
1.61349	169.11189	189.29362	140.18907
1.61244	170.04014	189.67652	139.6478
1.6114	171.00592	190.07104	139.15304
1.61035	172.00667	190.47384	138.70811
1.60931	173.03962	190.88133	138.31618
1.60827	174.1018	191.28977	137.98031

1.60723	175.19003	191.6952	137.70338
1.60619	176.30095	192.0935	137.4881
1.60515	177.43098	192.48034	137.33695
1.60412	178.57633	192.85124	137.25222
1.60308	179.73304	193.20155	137.23595
1.60205	180.89695	193.52645	137.28991
1.60102	182.06368	193.82098	137.41558
1.59998	183.22871	194.08005	137.61418
1.59896	184.38731	194.29842	137.88658
1.59793	185.53456	194.47073	138.23333
1.5969	186.66539	194.59152	138.65463
1.59587	187.77456	194.65522	139.15032
1.59485	188.85666	194.65617	139.71986
1.59383	189.90613	194.58863	140.36233
1.59281	190.91725	194.44679	141.07639
1.59178	191.88418	194.22478	141.86031
1.59077	192.80093	193.91671	142.71192
1.58975	193.66139	193.51661	143.62864
1.58873	194.45934	193.01852	144.60744
1.58772	195.18844	192.41646	145.64485
1.5867	195.84226	191.70446	146.73697
1.58569	196.41428	190.87656	147.87945
1.58468	196.89791	189.92682	149.06749
1.58367	197.28647	188.84934	150.29585
1.58266	197.57325	187.63827	151.55886
1.58165	197.75146	186.28783	152.85038
1.58065	197.81431	184.7923	154.16389
1.57964	197.75497	183.14605	155.49242
1.57864	197.56659	181.34354	156.82859
1.57764	197.24232	179.37935	158.16463

1.57663	196.77533	177.24816	159.49237
1.57563	196.15882	174.94479	160.8033
1.57464	195.386	172.4642	162.08852
1.57364	194.45016	169.80147	163.33881
1.57264	193.34462	166.95187	164.54465
1.57165	192.0628	163.91082	165.69619
1.57065	190.59818	160.6739	166.78335
1.56966	188.94435	157.2369	167.79576
1.56867	187.09502	153.59577	168.72286
1.56768	185.04401	149.74668	169.55387
1.56669	182.78528	145.68598	170.27786
1.56571	180.31293	141.41026	170.88375
1.56472	177.62123	136.91629	171.36036
1.56374	174.70461	132.20108	171.69642
1.56275	171.5577	127.26187	171.88062
1.56177	168.17531	122.09611	171.90163
1.56079	164.55245	116.7015	171.74815
1.55981	160.68438	111.07597	171.4089
1.55883	156.56654	105.2177	170.87272
1.55785	152.19465	99.12508	170.12855
1.55688	147.56466	92.79679	169.16548
1.5559	142.67278	86.23172	167.9728
